# Pyramidalization/twisting of the amide functional group *via* remote steric congestion triggered by metal coordination†
†Electronic supplementary information (ESI) available. CCDC 1494998–1495005. For ESI and crystallographic data in CIF or other electronic format see DOI: 10.1039/c6sc03669d



**DOI:** 10.1039/c6sc03669d

**Published:** 2016-09-23

**Authors:** Shinya Adachi, Naoya Kumagai, Masakatsu Shibasaki

**Affiliations:** a Institute of Microbial Chemistry (BIKAKEN) , 3-14-23 Kamiosaki , Shinagawa-ku , Tokyo 141-0021 , Japan . Email: nkumagai@bikaken.or.jp ; Email: mshibasa@bikaken.or.jp

## Abstract

For decades, the planarity of the amide functional group has garnered sustained interest in organic chemistry, enticing chemists to deform its usually characteristic high-fidelity plane. As opposed to the construction of amides that are distorted by imposing rigid covalent bond assemblies, we demonstrate herein the deformation of the amide plane through increased steric bulk in the periphery of the amide moiety, which is induced by coordination to metal cations. A crystallographic analysis revealed that the thus obtained amides exhibit significant pyramidalization and twisting upon coordination to the metals, while the amide functional group remained intact. The observed deformation, which should be attributed to through-space interactions, substantially enhanced the solvolytic cleavage of the amide, providing compelling evidence that temporary crowding in the periphery of the amide functional group may be used to control the reactivity of amides.

## Abstract 2

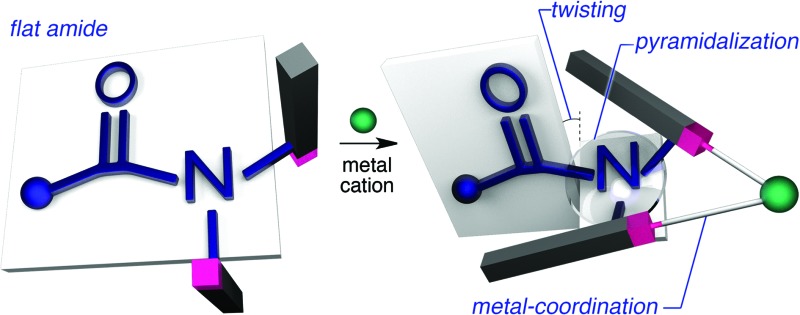
The distortion of the planar structure of amides is manifested by taking advantage of the temporary increase of the peripheral steric factor.

## Introduction

**Fig. 1:**
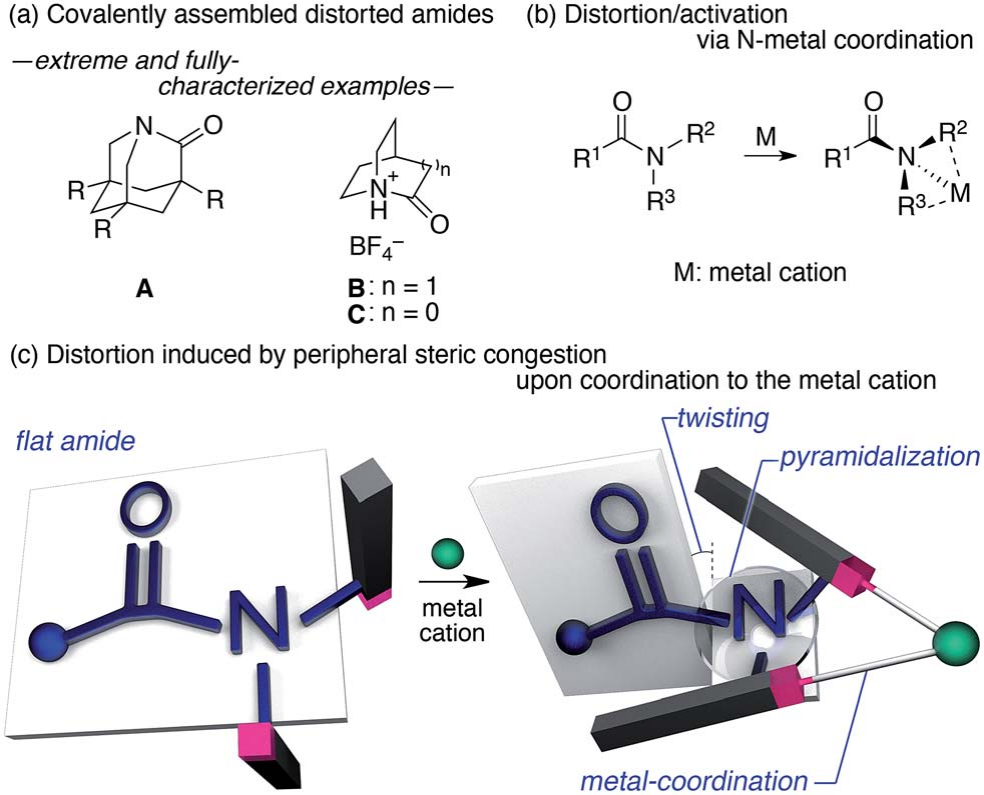
(a) Extreme examples of highly distorted amides in a covalent framework, (b) deformation/activation of amides *via* the coordination of the amide nitrogen to metals, and (c) the distortion of the amide induced by peripheral steric constraints upon the coordination to metal cations.

The amide bond is characterized by its thermodynamic stability and kinetic tolerance toward hydrolytic cleavage, which arises from conjugation *via* the planar O–C–N array.^1^ Under neutral conditions in aqueous solution at ambient temperature, non-activated amide bonds have a half-life of *ca.* 100 years.^2^ This high stability is usually attributed to the resonance interaction between the n_N_ and π*_C=O_ orbitals, which occurs most efficiently in a planar geometry with a shortened and stronger C–N bond. Therefore, amide bonds are commonly used as a robust structural motif (*e.g.* in synthetic polymers), and the hydrolysis of the amide bond in a practical timescale requires general harsh conditions (*e.g.* high or low pH at elevated temperatures). The deformation of the co-planarity of the amide bond represents an intuitive strategy to lower its robustness, which was initially proposed by Lukeš in 1938, who presented a model of strained “twisted amides” with a nitrogen atom at the bridgehead position.^3^ The longstanding pursuit toward twisted amides led to the identification of extreme examples of fully-characterized and highly distorted amides (**A–C**),^4,5^ which exhibited twist angles (*τ*) and pyramidalization (*χ*_N_) values at the nitrogen, defined by Winkler and Dunitz,^6^ of *ca.* 90° and 60°, respectively (Fig. 1a). The prime importance of the resonance interaction for stabilizing the amide bond manifests in the case of **B**, which lacks an amide resonance, and exhibits a remarkably short half-life of <15 s in water.^5*d*^ Besides these extreme examples, a number of amides that exhibit unusual *τ* and *χ*_N_ values have been reported, typically using covalently distorted bridged lactam architectures.^1,7,8^ These structurally intriguing bonds may not only be of fundamental academic interest, but also of significant applied importance in life science, considering that amides constitute the primary backbone of proteins. Indeed, the involvement of distorted amides has been invoked for enzymatic transformations.^1,9^ In this context, we were interested in distorting the amide planarity using an external trigger; more specifically, instead of constructing covalently assembled distorted amides, we aimed at inducing the amide deformation *via* temporary non-covalent interactions. Although several reports link the deformation and activation (*e.g.* hydrolysis and *E*/*Z* isomerization) of amides to the coordination of the amide nitrogen to metal cations (Fig. 1b),^10,11^ substantial amide deformation without direct coordination of the amide nitrogen or oxygen has, to the best of our knowledge, not yet been reported. Herein, we show that it is possible to induce significant pyramidalization and twisting of the amide functional group by remote steric congestion upon the coordination of the substituents attached to the amide nitrogen to a metal cation (Fig. 1c). A crystallographic analysis revealed that the substantial deformation of the amide planarity occurs without direct coordination of the amide. Peripheral crowding being a viable strategy to weaken the amide linkage is supported by the observed rapid solvolysis of the thus obtained distorted amides.^12^

## Results and discussion

**Fig. 2:**
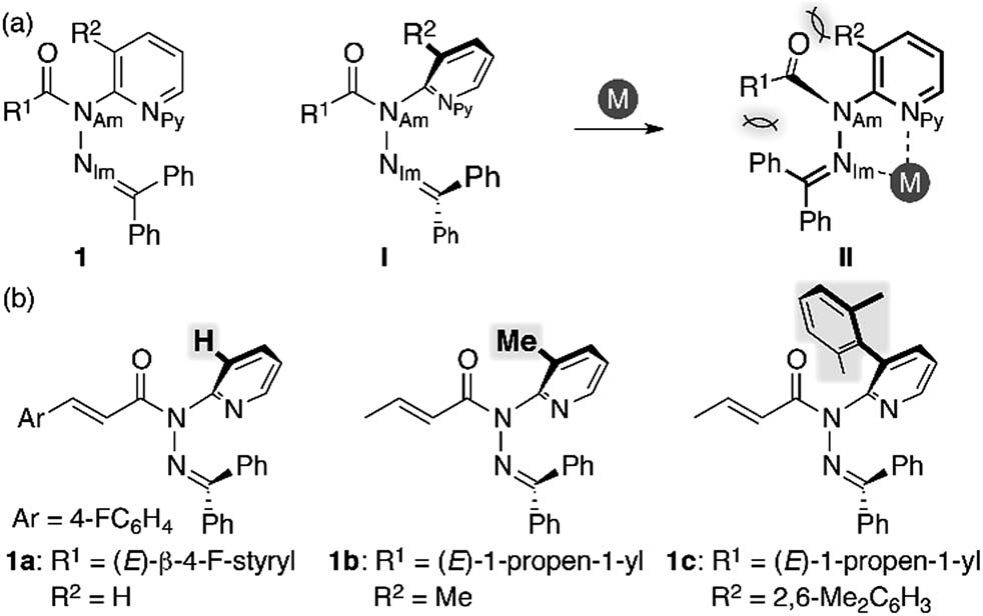
(a) Amide **1** with a 3-substituted-2-hydrazonopyridine moiety as a model compound and (b) amides **1a–c** with R^2^ substituents of different sizes.

We began our study by designing a suitable amide with metal-coordination sites that may be able to create a steric bias upon the addition of appropriate metal cations. As the metal coordination should be orthogonal to the amide functional group, we selected a combination of azophilic metals and nitrogen-based bidentate coordination sites. Fig. 2a shows the generic structure of amide **1** with a 3-substituted-2-hydrazonopyridine moiety, which contains an amide (N_Am_) and an adjacent imine (hydrazone of benzophenone; N_Im_) functional group. It should be noted that **1** prefers a planar amide structure, which is achieved by tilting the pyridine ring and the imine along the C(pyridine)–N_Am_ and N_Am_–N_Im_ single bonds, respectively (Fig. 2a, **I**). Conversely, we anticipated that the addition of azophilic cations (**M**) should induce a bidentate chelation through N_Im_ and N_Py_, thus affording a rigid and planar 5-membered cycle. This conformational change should provoke both the bulky benzophenone imine and the R^2^ group on the pyridine ring to swing close to the amide, thus compromising the amide planarity *via* through-space steric bias (Fig. 2a, **II**). With this blueprint in hand, we set out to synthesize three derivatives, which contain R^2^ groups of varying steric bulk: H (**1a**), Me (**1b**), and 2,6-dimethylphenyl (**1c**) (Fig. 2b).^13^ While **1b** and **1c** were synthesized as (*E*)-crotonyl amides, **1a** was based on a *p*-fluorocinnamoyl amide in order to increase its crystallinity.

**Fig. 3:**
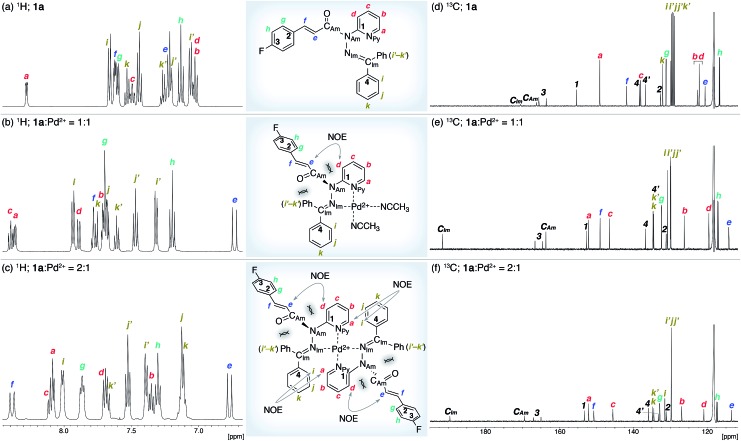
NMR analysis of **1a** and its Pd^2+^ complexes in CD_3_CN. (a and d) ^1^H and ^13^C NMR spectra of **1a**. (b and e) ^1^H and ^13^C NMR spectra of a 1 : 1 mixture of **1a** and [Pd(CH_3_CN)_4_](BF_4_)_2_. (c and f) ^1^H and ^13^C NMR spectra of a 2 : 1 mixture of **1a** and [Pd(CH_3_CN)_4_](BF_4_)_2_.

**Fig. 4:**
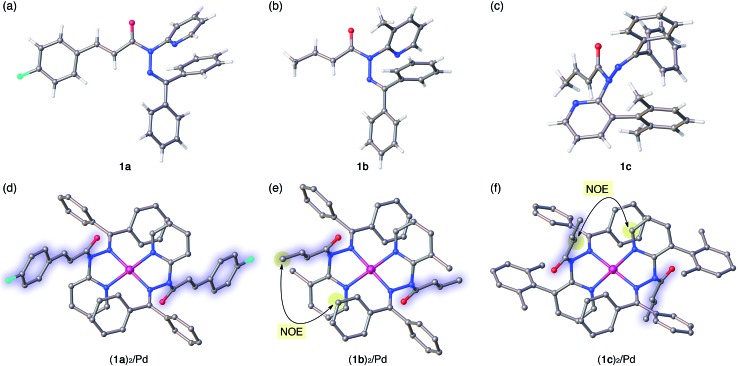
Crystal structures of amides **1a–c** (a–c) and of their Pd complexes (**1a**)_2_/Pd, (**1b**)_2_/Pd, and (**1c**)_2_/Pd (d–f). For the Pd complexes, hydrogen atoms and BF_4_^–^ anions are omitted for clarity. In (d)–(f), the amide fragments are highlighted. Color code: white = hydrogen, gray = carbon, blue = nitrogen, red = oxygen, sky blue = fluorine, and pink = palladium. In (e) and (f), characteristic NOE signals between protons observed in the NMR analysis in solution are highlighted.

The coordination of **1a** to azophilic Pd^2+^ cations, which favor a square-planar coordination mode, afforded in aprotic solvents the corresponding complexes. The formation of these complexes, which are thermodynamically stable under anhydrous conditions at ambient temperature, was monitored using ^1^H and ^13^C NMR spectroscopy. The NMR analysis revealed that **1a**/Pd (**1a** : Pd = 1 : 1) and (**1a**)_2_/Pd (**1a** : Pd = 2 : 1) complexes were formed depending on the ratio of **1a** and [Pd(CH_3_CN)_4_](BF_4_)_2_ (Fig. 3). In CD_3_CN, a 1 : 1 mixture of **1a** and [Pd(CH_3_CN)_4_(BF_4_)_2_ favored the formation of **1a**/Pd. The observed NOE signals between H_e_ and H_d_ are consistent with the anticipated coordination mode *via* N_py_ and N_Im_, in which the amide nitrogen N_Am_ is left uncoordinated (Fig. 3a and b).^14^ The characteristic downfield shift of the β-olefinic proton H_f_ upon complexation implied an increased polarization of the C=O bond *via* distortion of the amide moiety. The formation of the homoleptic complex (**1a**)_2_/Pd from bidentate coordination *via* N_py_ and N_Im_ induced similar spectral changes in the ^1^H and ^13^C NMR spectra, together with diagnostic NOE signals between the two **1a** fragments (H_a_ and H_i_; Fig. 3c). For (**1a**)_2_/Pd, the observed downfield shift of H_f_ was even more pronounced, which was tentatively ascribed to the deshielding effect of the phenyl group on the opposite **1a**/Pd fragment (Fig. 3b and c and 4d; *vide infra*). Unfortunately, the chemical shifts in the ^13^C NMR spectra were not straightforward to interpret (Fig. 3d–f); in contrast to the rather subtle changes to the resonances for the amide carbonyl (C_Am_) moiety, the signal for the imino carbonyl (C_Im_) fragment experienced a substantial downfield shift. In the Ph group-rich environment of these complexes, the downfield shift of the amide carbonyl *via* distortion and the imino carbonyl *via* direct coordination might be increased and decreased, respectively, by shielding and deshielding effects from nearby multiple bonds (*vide infra*).

The distortion of the amide plane in **1a** by peripheral crowding was further examined *via* single-crystal X-ray diffraction analysis (Fig. 4a and d). Single crystals of amide **1a** and its Pd complex (**1a**)_2_/Pd were obtained from acetone/hexane, and their solid-state structures are shown in Fig. 4a and d, while selected bond lengths and distortion parameters are summarized in Table 1. In **1a**, the pyridyl and the hydrazine group occupy the far side of the amide group in order to minimize steric repulsion. The amide group exhibited a negligible twist angle (*τ* = 3.0°), whereas partial pyramidalization was observed for the amide nitrogen (*χ*_N_ = 19.6°). In stark contrast, the complex (**1a**)_2_/Pd exhibited a significant pyramidalization (*χ*_N_ = 54.1°) of the amide nitrogen, and the expected bidentate coordination *via* N_py_ and N_Im_ was confirmed. The pyramidalization of the amide nitrogen, in combination with a twisting of the amide (*τ* = 12.1°) resulted in a diminished amide conjugation, which is consistent with the observed decreased C_Am_ =O and increased N–C_Am_(=O) bond lengths (Table 1). It should be noted here that the pyramidalization and twisting do not originate from the direct coordination to the amide functional group, but from the steric congestion in the periphery of the amide functional group.^10b,d^ Similar to other covalently assembled distorted amides, the pyramidalization of the carbonyl carbon (*χ*_C_) was negligible for both **1a** and (**1a**)_2_/Pd. The unusual downfield shift of the imino carbonyl (C_Im_) resonance in the ^13^C NMR spectrum may arise from the deshielding effects of the amide C=O bond in close proximity (Fig. 3d and f, and Table 1). Similarly, the orientation of the Ph groups in (**1a**)_2_/Pd most likely influences the chemical shift of the amide carbonyl carbon (C_Am_) and the β-olefinic hydrogen H_f_ (Fig. 3c, e and f and Table 1).

**Table 1:** Selected ^13^C NMR shifts and structural parameters for the crystal structures of **1a–c** and their Pd complexes (**1a**)_2_/Pd–(**1c**)_2_/Pd

	^13^C NMR^*a*^ [ppm]		Bond length [Å]				Distortion parameters^*b*^		
	C_Am_	C_Im_	N–C_Am_(=O)	C_Am_ =O	Pd–N_Im_	Pd–N_Py_	*χ*_N_	*χ*_C_	*τ*
**1a**	166.2	170.5	1.386(1)	1.227(1)			19.6°	1.8°	1.8°
(**1a**)_2_/Pd	169.4	189.6	1.425(6)	1.212(6)	2.003(5)	2.024(4)	54.1°	1.4°	12.2°
**1b**	168.1	161.9	1.394(3)	1.224(3)			28.3°	2.7°	4.1°
(**1b**)_2_/Pd	169.0	189.8	1.421(7)	1.216(6)	1.996(3)	2.022(4)	56.3°	3.1°	19.3°
**1c**	163.8	169.8	1.407(3)	1.213(4)			43.8°	4.3°	19.2°
(**1c**)_2_/Pd	171.0	187.9	1.444(9)	1.211(9)	2.019(5)	2.018(5)	51.9°	0.5°	4.3°

^*a*^Chemical shifts in CD_3_CN.

^*b*^ *χ*_N_, *χ*_C_, and *τ* denote the pyramidalization of the amide nitrogen atom, the carbonyl carbon atom, and the twist angle, respectively.^6^

The crystallographic analysis of amides **1b** and **1c** as well as of their Pd^2+^ complexes revealed an identical coordination mode *via* N_py_ and N_Im_, as well as distortion of the amide moiety (Fig. 4b, c, e and f).^15^ Although the free amides **1b** and **1c** showed only a moderate pyramidalization of the amide nitrogen (*χ*_N_), presumably due to the increased steric demand of R^2^ (**1a**: R^2^ = H, **1b**: R^2^ = Me, **1c**: R^2^ = 2,6-dimethylphenyl), the pyramidalization was significantly enhanced upon coordination to Pd^2+^. The elongated C_Am_ =O and compressed N–C_Am_(=O) bonds suggest a diminished amide conjugation (Table 1). It should also be noted that all three complexes show *χ*_N_ values beyond 50°. Similar spectral (^1^H and ^13^C NMR) patterns were observed upon complexation of **1b** and **1c**. More importantly, characteristic NOE signals were observed between the two amide fragments in (**1b**)_2_/Pd and (**1c**)_2_/Pd, which provides compelling evidence for significant N-pyramidalization in solution (Fig. 4e and f).^16^ For **1c**, some anomalies were observed: while **1a** and **1b** are *E*-amides in their free form and afford homoleptic complexes (**1**)_2_/Pd with *Z*-amides, the structural behavior of **1c** is antipodal. Moreover, the twist angle (*τ*) decreased upon the coordination of **1c** to Pd^2+^, whereas the planarity of the amide moiety was substantially disrupted by considerable pyramidalization. For amide **1c**, a 1 : 1 Pd complex (**1c**/Pd) could be isolated, which exhibited an identical coordination mode with amide distortion (*χ*_N_ = 57.5°),^17^ thus supporting the structure that was assigned to **1a**/Pd (Fig. 3b and e) on the basis of the NMR analysis.

**Fig. 5:**
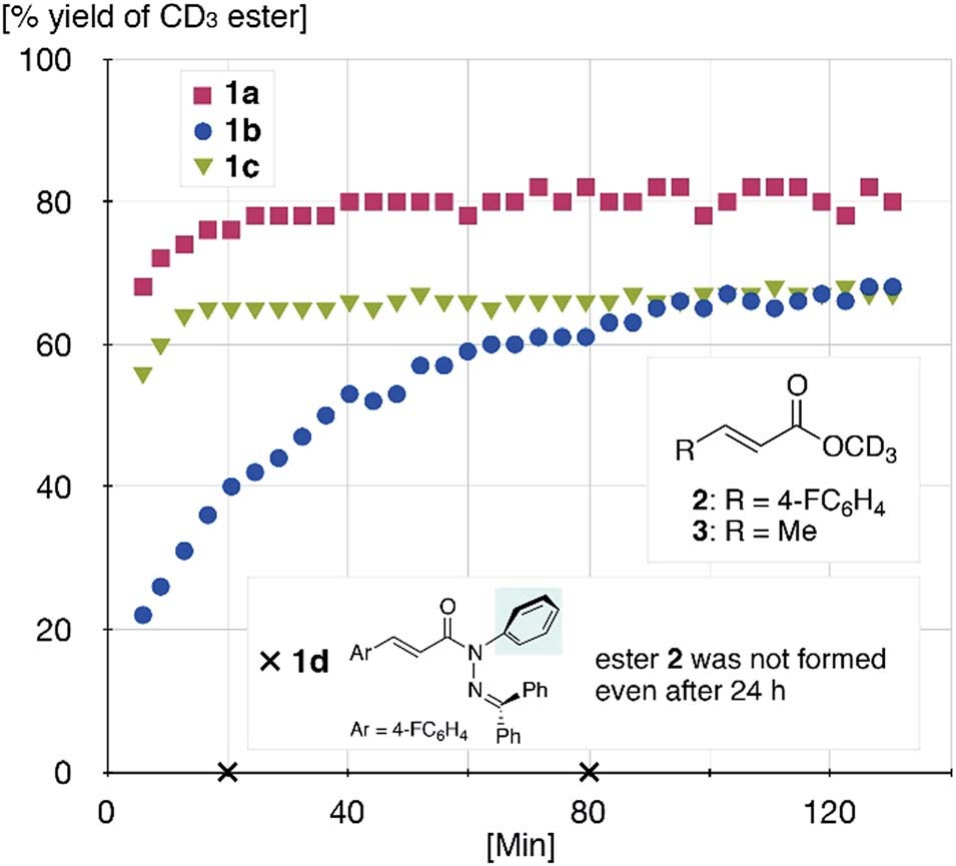
Methanolysis of **1a–d** in the presence of 1 equiv. of [Pd(CH_3_CN)_4_](BF_4_)_2_ at 27 °C monitored using ^1^H NMR spectroscopy.

**Fig. 6:**
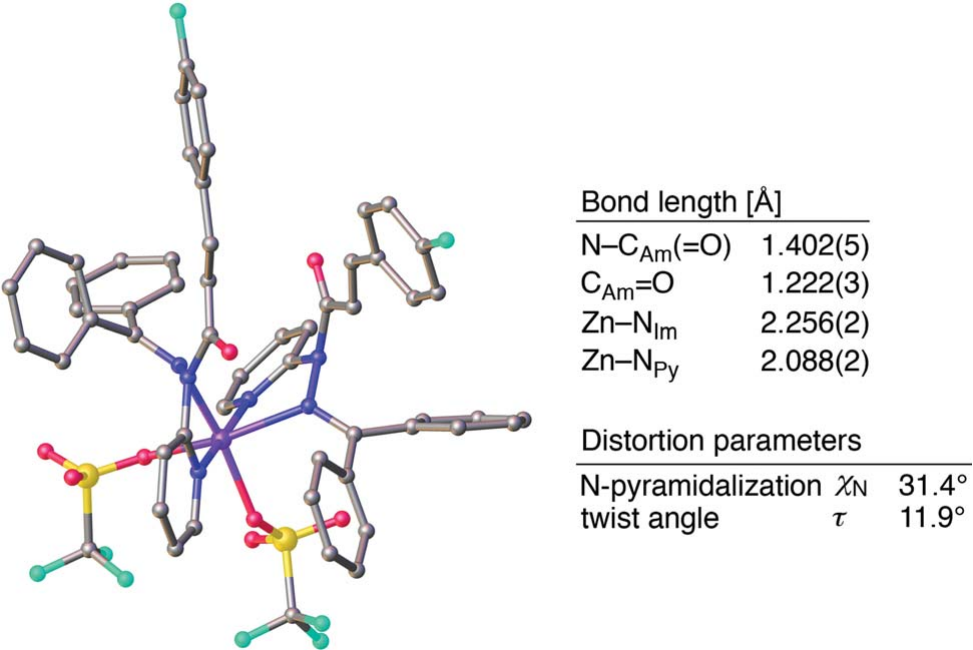
Crystal structure of (**1a**)_2_/Zn with selected structural parameters. Hydrogen atoms are omitted for clarity. Color code: gray = carbon, blue = nitrogen, red = oxygen, sky blue = fluorine, yellow = sulfur, and purple = zinc.

The sterically driven distortion of the amide moieties led to a substantially enhanced rate of solvolytic cleavage under neutral and ambient conditions.^10a,c,d,f–i^ Amides **1a–c** exhibited high levels of stability toward hydrolytic cleavage under such conditions, and no methanolysis was detected in CD_3_OD. However, in the presence of [Pd(CH_3_CN)_4_](BF_4_)_2_, **1a–c** were rapidly converted to the corresponding CD_3_ esters **2** and **3** under otherwise identical conditions (Fig. 5). Considering the aforementioned NMR and crystallographic analyses, this reactivity should be interpreted in terms of an electrophilic activation of the amide moiety in solution, which is most likely caused by a diminished amide conjugation in response to peripheral steric congestion.^18^ In contrast, amide **1d**, which lacks the pyridine unit for chelating complexation with Pd^2+^, did not produce any detectable quantity of the corresponding ester, thus further supporting the importance of the peripheral congestion. While (**1c**)_2_/Pd exhibited a small twist angle (*τ* = 4.3°), the methanolysis rate for **1c** was significantly increased, suggesting that the pyramidalization of the amide nitrogen atom is predominantly responsible for the electrophilic activation. The chelating unit liberated by the methanolysis was found to strongly bind to Pd^2+^, and the slow association/dissociation kinetics of these amides and Pd^2+^ prevented a catalytic methanolysis.^17,19^ As is evident from the averaged ^1^H NMR spectrum, Zn^2+^ showed faster complexation kinetics in comparison,^17^ which allows the methanolysis to proceed catalytically, converting amide **1a** to CD_3_ ester **2** in a 92% yield using 5 mol% of Zn(OTf)_2_ (eqn (1)).^20^ The crystallographic analysis of (**1a**)_2_/Zn revealed a coordination *via* N_Py_ and N_Im_, which corroborates that a sterically driven amide activation should also be operative in the case of complexation with Zn^2+^ (Fig. 6).^21^

1

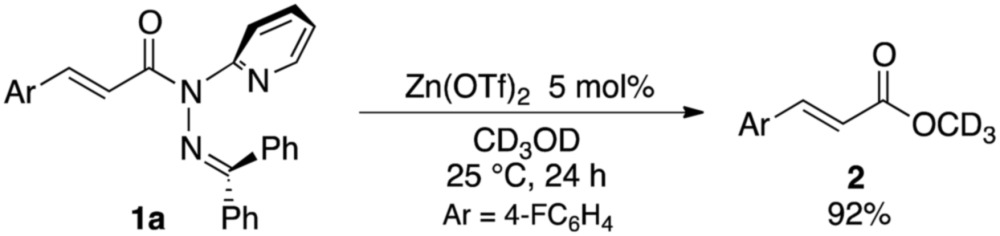




## Conclusions

In conclusion, we demonstrated that peripheral steric constraint may induce the pyramidalization and twisting of the amide functional group. The results summarized herein represent proof-of-concept that the distortion of the amide moiety can be induced in an indirect and temporary fashion, which stands in contrast to the previously reported permanent distortion by covalent bonds or to the temporary distortion *via* the direct coordination of the amide functional group. The crystallographic analysis of the Pd^2+^ and Zn^2+^ complexes of **1** revealed the presence of distorted amides in the solid state, and the observed coordination patterns, as well as the pyramidalization of the amide nitrogen were confirmed in solution *via* NMR analysis. The distortion of the amide moiety in solution was further supported by the enhanced solvolysis rates in the presence of metal cations. Further studies to transfer the present results to the chemoselective activation of simple amides *via* peripheral crowding are currently under way and will be reported in due course.
