# Supplementary material for: Pyramidalization/twisting of the amide functional group via remote steric congestion triggered by metal coordination
Source: Chem Sci. 2016 Sep 23;8(1):85–90. doi: 10.1039/c6sc03669d (PMC5304688; doi:10.1039/c6sc03669d)
Supplement: Supplementary file 1 [file SC-008-C6SC03669D-s001.pdf]

Electronic Supplementary Material (ESI) for Chemical Science.
This journal is © The Royal Society of Chemistry 2016

Pyramidalization/Twisting of the Amide Functionality via Remote Steric Congestion Triggered by Metal–Coordination

Shinya Adachi, Naoya Kumagai,* Masakatsu Shibasaki*

Institute of Microbial Chemistry (BIKAKEN), Tokyo, Japan

nkumagai@bikaken.or.jp, mshibasa@bikaken.or.jp

1. General Methods **2**

1–1. Reactions and purifications *2*
1–2. Characterizations *2*
1–3. Solvents and reagents *2*

2. General Procedures **3**

*2–1. Synthesis of **1a**.* *3*
*2–2. Synthesis of **1b**.* *3*
*2–3. Synthesis of **1c**.* *4*
*2–4. Synthesis of **1d**.* *5*
*2–5. Preparation and characterization of metal complexes with amides **1a**–**c*** *6*
*2–6. Synthesis of ester **2*** *7*

3. NMR Analyses of Amide–Metal Complexes **8**

*3–1. **1a**–Pd Complexes* *8*
*3–1–1. Detailed NMR of **1a** for peak assignments* *8*
*3–1–2. Detailed NMR of **1a**/Pd for peak assignments* *10*
*3–1–3. Detailed NMR of (**1a**)$_2$/Pd for peak assignments* *13*
*3–1–4. Stacked ^{1}H and ^{13}C NMR of **1a**, **1a**/Pd, and (**1a**)$_2$/Pd* *17*
*3–2. **1b**–Pd Complex* *19*
*3–2–1. Detailed NMR of **1b** for peak assignments* *19*
*3–2–2. Detailed NMR of (**1b**)$_2$/Pd for peak assignments* *23*
*3–2–3. Stacked ^{1}H and ^{13}C NMR of **1b** and (**1b**)$_2$/Pd* *30*
*3–1. **1c**–Pd Complex* *31*
*3–3–1. Detailed NMR of **1c** for peak assignments* *31*
*3–3–2. Detailed NMR of (**1c**)$_2$/Pd for peak assignments* *34*
*3–3–3. Stacked ^{1}H and ^{13}C NMR of **1c** and (**1c**)$_2$/Pd* *44*
*3–4–1. ^{1}H NMR of a 1:1 mixture of amide **1b** or **1c** with [Pd(CH_3CN)$_4$](BF_4)$_2$* *45*
*3–5–1. ^{1}H NMR of **1a**–metal complexes with variable stoichiometry* *46*

4. Crystal Structures of Amides 1a–c and Their Pd Complexes **47**

*4–1. Crystal structures of amides **1a**–**c*** *47*
*4–2. Crystal structures of metal complexes with amides **1a**–**c*** *50*

5. References **55**

6. NMR Spectra of New Compounds (^{1}H, ^{13}C, and ^{19}F NMR spectra) **56**

1. General Methods

1-1. Reactions and purifications

Unless otherwise noted, all reactions were carried out in an oven-dried flask fitted with a septum under an argon atmosphere with magnetically stirred chips. All work-up and purification procedures were carried out with reagent-grade solvents under ambient atmosphere. Thin layer chromatography (TLC) was performed on Merck TLC plates (0.25 mm) with silica gel 60 F254 and visualized by UV quenching and staining with $KMnO_4$. Flash column chromatography was performed using SiO_2 [Kanto Chemical 60N (neutral, spherical, 50–60 μm)] or CombiFlash systems with a Redisep column..

1-2. Characterizations

Infrared (IR) spectra were recorded on a HORIBA FT210 Fourier transform infrared spectrophotometer. NMR spectra were recorded on a Bruker AVANCE III HD400, Bruker AVANCE III 500, or a JEOL ECZ-600R. Chemical shifts (δ) are given in ppm relative to residual solvent peaks.[1] Data for ^{1}H NMR are reported as follows: chemical shift (multiplicity, coupling constants where applicable, number of hydrogens). Abbreviations are as follows: s (singlet), d (doublet), t (triplet), dd (doublet of doublet), dt (doublet of triplet), ddd (doublet of doublet of doublet), q (quartet), m (multiplet), br (broad). For ^{19}F NMR, chemical shifts were reported in the scale relative to $PhCF_3$ (δ –62.7680 ppm in $CDCl_3$) as an external reference. High-resolution mass spectra were measured on a Thermo Fisher Scientific LTQ Orbitrap XL.

1-3. Solvents and reagents

THF, toluene, hexane, and CH_3CN were purified by passing through a solvent purification system (Glass Contour). All other materials were used as supplied by commercial vendors or prepared by the method described in the corresponding references.

2. General Procedures

2-1. Synthesis of **1a**.

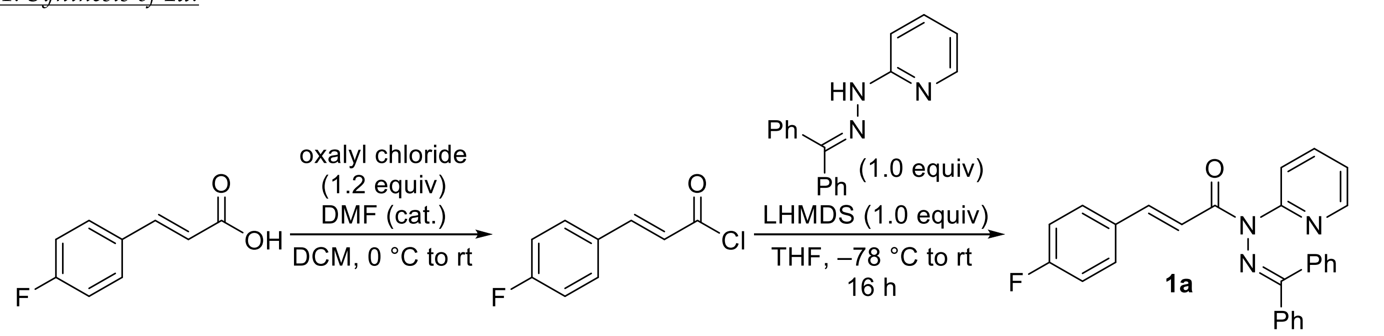

(*E*)-*N*'-(Diphenylmethylene)-3-(4-fluorophenyl)-*N*-(pyridin-2-yl)acrylohydrazide (1a)

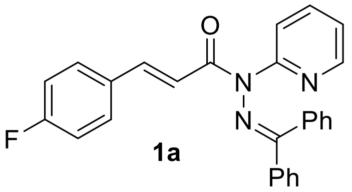

To a solution of *p*-fluorocinnamic acid (166 mg, 1.0 mmol, 1.0 equiv) in 2 mL of dichloromethane at 0 °C, under argon, was added oxalyl chloride (152 mg, 1.2 mmol, 1.2 equiv) followed by one drop of DMF. After 10 minutes the solution was allowed to warm to room temperature and stirred for 2 h. The solvent and excess oxalyl chloride was removed *in vacuo* to yield the corresponding crude acid chloride, which was used without further purification.

To a stirred solution of LHMDS (1.0 M in THF, 1.0 mmol) at –78 °C was slowly added 2-(2-(diphenylmethylene)hydrazinyl)pyridine[2] (273.3 mg, 1.0 mmol, 1.0 equiv) in anhydrous THF (2.0 mL). After 1 h, the crude acid chloride (1.0 mmol, 1.0 equiv) in anhydrous THF (2.5 mL) was then added dropwise and the mixture was allowed to stir for 30 minitues at –78 °C and then allowed to warm to room temperature over 4 h and stirred for 12 h. The reaction mixture was diluted with EtOAc and washed with saturated aqueous $NaHCO_3$. The organic layer was then dried over Na_2SO_4, filtered, and concentrated under reduced pressure. Purification of the residue by silica gel flash column chromatography (hexane/EtOAc, 100:0 –4:1 –2:1) gave title compound **1a** (307 mg, 73% yield).

Yellow solid, m.p.: 127–129 °C; ^{1}H NMR (500 MHz, 298 K, CD_3CN): δ 8.29–8.27 (m, 1H), 7.67–7.65 (m, 2H), 7.63–7.59 (m, 3H), 7.55–7.48 (m, 2H), 7.46–7.42 (m, 2H), 7.28–7.20 (m, 4H), 7.15–7.11 (m, 2H), 7.07–7.01 (m, 4H); ^{13}C NMR (125 MHz, 298 K, CD_3CN): δ 170.5, 166.2, 164.6 (d, *J* = 248.3 Hz), 155.4, 149.1, 141.8, 138.4, 138.0, 136.7, 132.7 (d, *J* = 3.2 Hz), 132.1, 131.2 (d, *J* = 8.8 Hz), 129.9, 129.7, 129.4, 129.1, 129.0, 122.8, 122.3, 120.6 (d, *J* = 2.5 Hz), 116.8 (d, *J* = 22.1 Hz); ^{19}F NMR (376 MHz, 343 K, $CDCl_3$): –112.4 (m); IR (KBr): $\tilde{\nu}$ = 3049, 1662, 1615, 1599, 1508, 1357, 1230, 994, 828, 699 cm^{-1}; HRMS (ESI) *m*/*z* calculated for $C_{27}H_{21}ON_3F$ $[M+H]^+$: 422.1663, found: 422.1661.

2-2. Synthesis of **1b**.

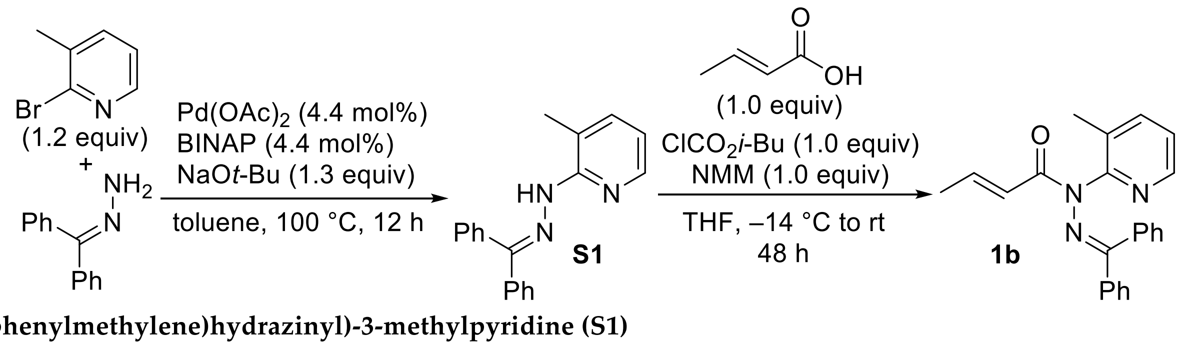

2-(2-(Diphenylmethylene)hydrazinyl)-3-methylpyridine (S1)

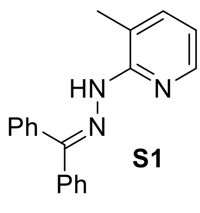

Following the procedure of J. B. Arterburn, et al.,[2] a flask was charged with benzophenone hydrazone (762 mg, 3.9 mmol, 1.0 equiv), $Pd(OAc)_2$ (38.3 mg, 0.17 mmol, 4.4 mol%), racemic 2,2′-bis(diphenylphosphanyl)-1,1′-binaphtyl (BINAP) (106.4 mg, 0.17 mmol, 4.4 mol%), and NaO*t*-Bu (653 mg, 6.8 mmol, 1.75 equiv) in a glove box. The flask was purged with argon, then anhydrous toluene (10 mL) and 2-bromo-3-methylpyridine (828 mg, 4.8 mmol, 1.24 equiv) were added. The reaction mixture was heated at 100 °C and stirred for 12 h. The reaction mixture was cooled, diluted with EtOAc, washed with water and concentrated under vacuum. The residue was purified by flash column chromatography (hexane/EtOAc, 100:0 – 6:1 – 4:1) to yield title compound **S1** (706 mg, 63% yield).

Brown solid, m.p.: 79–80 °C; ^{1}H NMR (400 MHz, 298 K, $CDCl_3$): δ 8.11 (dd, *J* = 5.0, 1.7 Hz, 1H), 7.94 (s, 1H), 7.67–7.56 (m, 4H), 7.55–7.48 (m, 1H), 7.39–7.28 (m, 6H), 6.72 (dd, *J* = 7.2, 4.9 Hz, 1H), 2.20 (s, 3H); ^{13}C NMR (100 MHz, 298 K, $CDCl_3$): δ 153.5, 147.4, 145.9, 139.4, 138.0, 132.9, 129.9, 129.5, 129.0, 128.5, 128.2, 127.0, 117.9, 116.1, 18.4; IR (KBr): $\tilde{\nu}$ = 3314, 3042,

2965, 1586, 1556, 1488, 1452, 1292, 1116, 785, 690 cm^{-1}; HRMS (ESI) *m/z* calculated for $C_{19}H_{18}N_3$ [M+H]$^+$: 288.1495, found: 288.1497.

(*E*)-*N*'-(Diphenylmethylene)-*N*-(3-methylpyridin-2-yl)but-2-enehydrazide (1b)

A solution of crotonic acid (86.1 mg, 1.0 mmol, 1.0 equiv) and *N*-methylmorpholine (202 mg, 2.0 mmol, 2.0 equiv) in anhydrous THF (7.0 mL) was cooled to –14 °C and treated dropwise with isobutyl chloroformate (137 mg, 1.0 mmol, 1.0 equiv). After 1 h, a solution of 2-(2-(diphenylmethylene)hydrazinyl)-3-methylpyridine **S1** (287 mg, 1.0 mmol, 1.0 equiv) in anhydrous THF (7.0 mL) was added dropwise. The reaction mixture was warmed to room temperature over 2 h and stirred for 46 h. The resulting mixture was diluted with EtOAc and washed with saturated aqueous $NaHCO_3$. The organic layer was dried over Na_2SO_4, filtered, and concentrated. The residue was purified by flash column chromatography (hexane/EtOAc, 100:0 – 4:1 – 2:1) to yield title compound **1b** (89.8 mg, 25% yield).

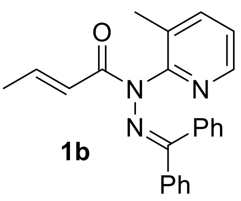

1b

*Note that the NMR spectra of title compound **1a** was recorded at elevated temperature (50 °C), as peaks were broadened at room temperature.*

Brown solid, m.p.: 154–156 °C; ^{1}H NMR (500 MHz, 323 K, CD_3CN): δ 8.14 (br d, *J* = *ca.* 4 Hz, 1H), 7.57–7.51 (m, 2H), 7.48–7.42 (m, 1H), 7.39–7.36 (m, 2H), 7.24–7.09 (m, 4H), 7.04–6.80 (m, 5H), 1.94 (s, 3H), 1.84 (s, 3H); ^{13}C NMR (125 MHz, 323 K, CD_3CN): δ 168.1, 161.9, 155.2, 147.3, 143.9, 139.7, 139.6, 137.4, 133.3, 131.3, 129.4, 129.3, 129.2, 128.9, 128.8, 124.2, 124.1, 18.5, 18.3; IR (KBr): $\tilde{\nu}$ = 2962, 2910, 1676, 1638, 1445, 1343, 1204, 967, 784, 698 cm^{-1}; HRMS (ESI) *m/z* calculated for $C_{23}H_{22}ON_3$ [M+H]$^+$: 356.1757, found: 356.1760.

*2-3. Synthesis of **1c**.*

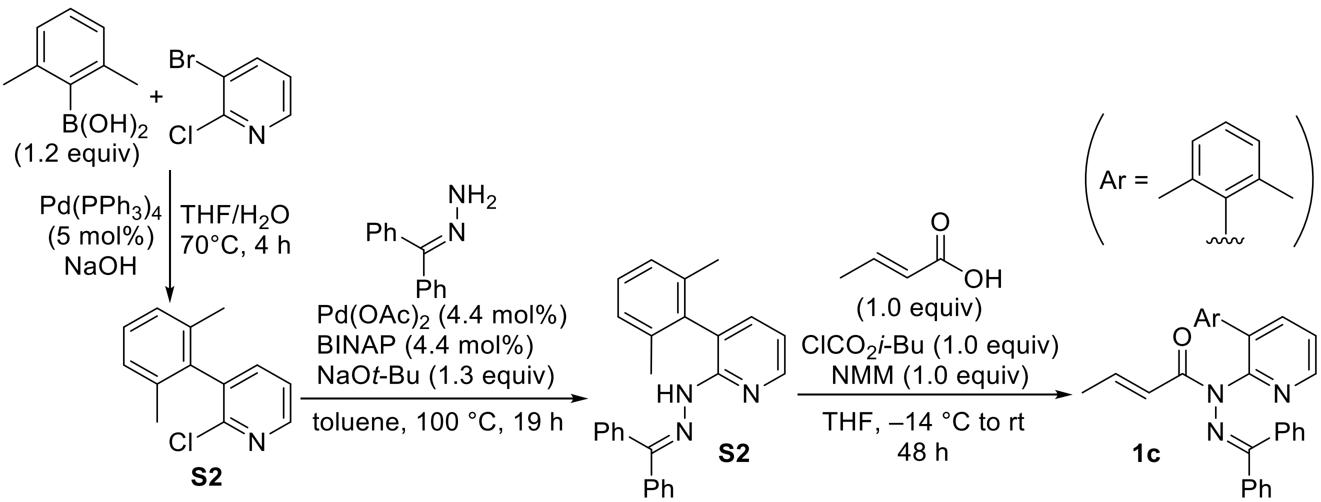

2-Chloro-3-(2,6-dimethylphenyl)pyridine (S2)

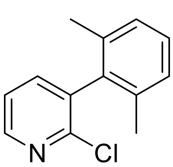

S2

Following the procedure of P. Langer et al.,[3] the flask charged with 3-bromo-2-chloropyridine (1.89 g, 9.8 mmol, 1.0 equiv), 2,6-dimethylphenyl boronic acid (1.77 g, 11.8 mmol, 1.2 equiv), $Pd(PPh_3)_4$ (568 mg, 0.49 mmol, 5.0 mol%), and NaOH (1.18 g, 29.6 mmol, 3.0 equiv) were evacuated and back filled with argon. To the mixture, THF (132 mL) and water (19 mL) were added, and then the reaction flask was evacuated and back filed several times with argon. The reaction was heated at 70 °C and stirred for 4 h. The solvent was evaporated *in vacuo*. The residue was extracted with EtOAc and water. The organic layer was dried over Na_2SO_4 and filtered and the solvent was evaporated *in vacuo*. The residue was purified by flash column chromatography (hexane/EtOAc, 100:0 – 19:1) to yield title compound **S2** (1.79 g, 84% yield).

White solid, m.p.: 94–96 °C; ^{1}H NMR (400 MHz, 298 K, $CDCl_3$): δ 8.36 (dd, *J* = 4.8, 2.0 Hz, 1H), 7.44 (dd, *J* = 7.5, 2.0 Hz, 1H), 7.26 (dd, *J* = 7.5, 4.8 Hz, 1H), 7.20–7.13 (m, 1H), 7.11–7.03 (m, 2H), 1.93 (s, 6H); ^{13}C NMR (100 MHz, 298 K, $CDCl_3$): δ 151.0, 148.8, 139.8, 136.9, 136.12, 136.09, 128.4, 127.6, 122.8, 20.4; IR (KBr): $\tilde{\nu}$ = 3047, 2911, 1554, 1446, 1386, 1085, 998, 778, 725 cm^{-1}; HRMS (ESI) *m/z* calculated for $C_{13}H_{13}NCl$ [M+H]$^+$: 218.0731, found: 218.0730.

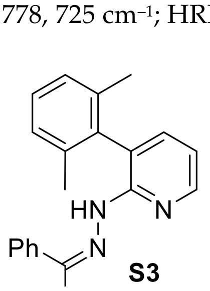

S3

2-(2-(Diphenylmethylene)hydrazinyl)-3-(2,6-dimethylphenyl)pyridine (S3)

Following the procedure of J. B. Arterburn, et al.,[2] a flask was charged with benzophenone

hydrazone (295 mg, 1.5 mmol, 1.0 equiv), Pd(OAc)$_2$ (14.9 mg, 0.066 mmol, 4.4 mol%), racemic 2,2′-bis(diphenylphosphanyl)-1,1′-binaphtyl (BINAP) (41.2 mg, 0.066 mmol, 4.4 mol%), and NaO*t*-Bu (253 mg, 2.6 mmol, 1.75 equiv) in a glove box. The flask was purged with argon, then anhydrous toluene (3.8 mL), and 2-chloro-3-(2,6-dimethylphenyl)pyridine **S2** (410 mg, 1.9 mmol, 1.25 equiv) were added. The reaction mixture was heated at 100 °C and stirred for 19 h. The reaction mixture was cooled, diluted with EtOAc, washed with water and concentrated under vacuum. The residue was purified by flash column chromatography (hexane/EtOAc, 100:0 – 6:1– 4:1) to yield title compound **S3** (461 mg, 81% yield).
Brown solid, m.p.: 119–121 °C; ^{1}H NMR (400 MHz, 298 K, CD$_3$CN): δ 8.26 (dd, *J* = 4.9, 1.8 Hz, 1H), 7.51 (s, 1H), 7.43–7.33 (m, 5H), 7.32–7.27 (m, 3H), 7.25 (dd, *J* = 7.3, 1.8 Hz, 1H), 7.08 (dd, *J* = 8.3, 6.9 Hz, 1H), 7.05–7.00 (m, 2H), 7.00–6.96 (m, 2H), 6.91 (dd, *J* = 7.3, 4.9 Hz, 1H), 1.86 (s, 6H); ^{13}C NMR (100 MHz, 298 K, CD$_3$CN): δ 152.5, 148.5, 148.4, 139.0, 138.8, 137.2, 135.8, 133.1, 130.5, 130.1, 129.4, 129.2, 129.1, 128.9, 128.8, 127.4, 121.5, 116.8, 20.3; IR (KBr): $\tilde{\nu}$ = 3319, 3021, 2913, 1575, 1503, 1488, 1395, 1235, 1217, 1128, 769, 705, 691 cm^{-1}; HRMS (ESI) *m/z* calculated for $C_{26}H_{24}N_3$ [M+H]$^+$: 378.1965, found: 378.1961.

(*E*)-*N*'-(Diphenylmethylene)-*N*-[3-(2,6-dimethylphenyl)pyridin-2-yl]but-2-enehydrazide (1c)
Following the above described reaction for the preparation of amide **1b**, amide **1c** was obtained in 28% yield (72.3 mg) from 2-(2-(diphenylmethylene)hydrazinyl)-3-(2,6-dimethylphenyl)pyridine **S3** (218 mg, 0.58 mmol) after purification by flash column chromatography (hexane/EtOAc, 100:0 – 4:1 – 2:1).

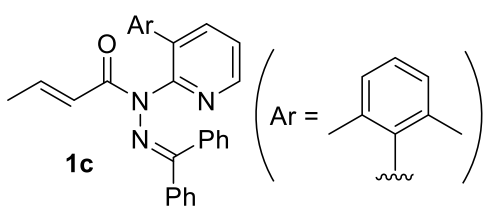

White solid, m.p.: 139–141 °C; ^{1}H NMR (400 MHz, 298 K, CD$_3$CN): δ 8.49 (dd, *J* = 4.8, 1.9 Hz, 1H), 7.62 (dd, *J* = 7.6, 1.9 Hz, 1H), 7.52–7.38 (m, 2H), 7.36–7.24 (m, 5H), 7.22–717 (m, 3H), 7.09 (br s, 2H), 6.60 (br s, 2H), 6.44 (dq, *J* = 14.1, 6.9 Hz, 1H), 5.65 (dq, *J* = 15.1, 1.8 Hz, 1H), 1.92 (s, 6H), 1.62 (dd, *J* = 6.9, 1.7 Hz, 3H); ^{13}C NMR (100 MHz, 298 K, CD$_3$CN): δ 169.8, 163.8, 155.4, 148.9, 142.0, 141.0, 139.0, 137.93, 137.92, 137.0, 135.4, 131.5, 129.6, 129.1, 129.0 (3C), 128.43, 128.38, 125.8, 124.5, 21.2, 17.9; ^{1}H NMR (400 MHz, 298 K, *d*$_6$-acetone): δ 8.52 (dd, *J* = 4.8, 1.9 Hz, 1H), 7.67 (dd, *J* = 7.6, 1.9 Hz, 1H), 7.53 (dd, *J* = 7.6, 4.7 Hz, 1H), 7.47–7.39 (m, 1H), 7.36–7.28 (m, 4H), 7.27–7.14 (m, 4H), 7.08 (br s, 2H), 6.62 (br s, 2H), 6.40 (dq, *J* = 14.1, 6.9 Hz, 1H), 5.67 (dq, *J* = 15.0, 1.7 Hz, 1H), 1.96 (s, 6H), 1.61 (dd, *J* = 6.9, 1.7 Hz, 3H); ^{13}C NMR (100 MHz, 298 K, *d*$_6$-acetone): δ 169.0, 163.4, 155.8, 148.7, 141.6, 139.8, 139.2, 138.1, 137.7, 137.2, 135.3, 131.1, 129.5, 129.1, 129.0, 128.9, 128.7, 128.3, 128.1, 126.2, 124.2, 21.2, 17.8; IR (KBr): $\tilde{\nu}$ = 3051, 2912, 1683, 1645, 1561, 1415, 1319, 1176, 965, 771, 695 cm^{-1}; HRMS (ESI) *m/z* calculated for $C_{30}H_{28}ON_3$ [M+H]$^+$: 446.2227, found: 446.2222.

<u>*2-4. Synthesis of* ***1d**.*</u>

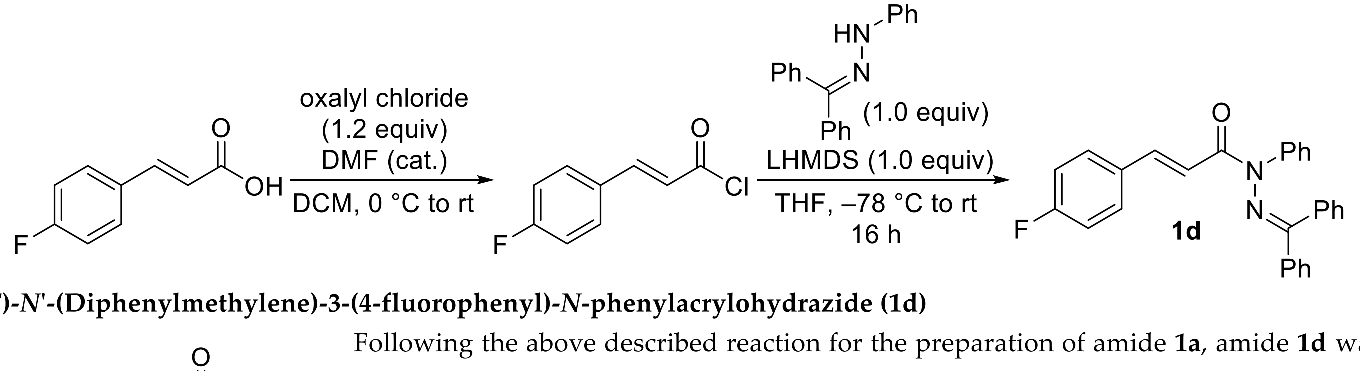

(*E*)-*N*'-(Diphenylmethylene)-3-(4-fluorophenyl)-*N*-phenylacrylohydrazide (1d)

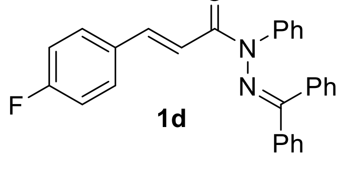

Following the above described reaction for the preparation of amide **1a**, amide **1d** was obtained in 29% yield (122 mg) from 1-(diphenylmethylene)-2-phenylhydrazine[4] (272 mg, 1.0 mmol) after purification by flash column chromatography (hexane/EtOAc, 100:0 – 4:1).
*Note that the NMR spectra of title compound **1d** was recorded at elevated temperature (70 °C), as peaks were broadened at room temperature.*
Yellow solid, m.p.: 134–136 °C; ^{1}H NMR (500 MHz, 343 K, DMSO-*d*$_6$): δ 7.79–7.50 (m, 6H), 7.47–7.44 (m, 2H), 7.39–7.25 (m, 3H), 7.22–7.17 (m, 4H), 7.13 (t, *J* = 7.3 Hz, 1H), 7.07–6.77 (m, 5H); ^{13}C NMR (125 MHz, 343 K, DMSO-*d*$_6$): δ 170.2, 163.5, 162.7 (d, *J* = 248.2 Hz), 141.7, 140.2, 136.7, 134.9, 131.1 (d, *J* = 3.3 Hz), 130.8, 129.8 (d, *J* = 8.5 Hz), 128.7, 128.3, 128.14, 128.09, 127.7, 127.4, 126.0, 125.7, 118.8 (d, *J* = 2.3 Hz), 115.5 (d, *J* = 21.9 Hz); ^{19}F NMR (376 MHz, 343 K, DMSO-*d*$_6$): –110.9 (m); IR (KBr): $\tilde{\nu}$ = 3056, 3036, 1652, 1615, 1600, 1591, 1507, 1489, 1372, 1225, 1157, 1003, 991, 824, 698 cm^{-1}; HRMS (ESI)

m/z calculated for $C_{28}H_{22}ON_2F$ [M+H]+: 421.1711 found: 421.1708.

*2-5. Preparation and characterization of metal complexes with amides **1a–c***

(*E*)-*N*'-(Diphenylmethylene)-3-(4-fluorophenyl)-*N*-(pyridin-2-yl)acrylohydrazide·*Pd* (1:1) Complex (1a/Pd)

Complex **1a**/Pd was formed immediately after the addition of CD_3CN (550 μL) into an NMR tube containing (*E*)-*N*'-(diphenylmethylene)-3-(4-fluorophenyl)-*N*-(pyridin-2-yl)acrylohydrazide (**1a**) (4.9 mg, 0.0117 mmol, 1.0 equiv) and $[Pd(CH_3CN)_4](BF_4)_2$ (5.2 mg, 0.0117 mmol, 1 equiv).

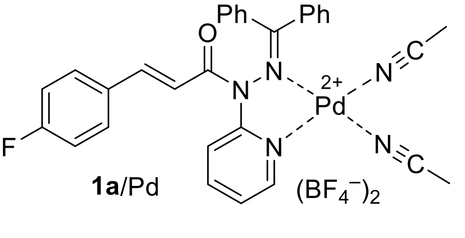

1a/Pd

^{1}H NMR (500 MHz, 298 K, CD_3CN): δ 8.40 (td, J = 7.9, 1.5 Hz, 1H), 8.36 (dd, *J* = 6.1, 1.4 Hz, 1H), 7.98–7.92 (m, 2H), 7.91–7.86 (m, 1H), 7.82–7.74 (m, 2H), 7.73–7.65 (m, 5H), 7.63–7.58 (m, 1H), 7.51–7.43 (m, 2H), 7.35–7.27 (m, 2H), 7.23–7.14 (m, 2H), 6.73 (d, *J* = 15.4 Hz, 1H); ^{13}C NMR (125 MHz, 298 K, CD_3CN): δ 191.6, 165.60 (d, *J* = 251.2 Hz), 163.6, 152.7, 152.1, 149.0, 146.4, 136.7, 134.7, 134.54, 134.50, 132.4 (d, *J* = 8.9 Hz), 131.2 (d, *J* = 3.2 Hz), 130.8, 130.00, 129.97, 129.9, 126.3, 119.6, 117.2 (d, *J* = 22.3 Hz), 114.2 (d, *J* = 2.5 Hz); ^{19}F NMR (376 MHz, 298 K, CD_3CN): –109.1 (m), –151.5 (s, $^{10}BF_4$), –151.6 (s, $^{11}BF_4$); HRMS (ESI) *m/z* calculated for $C_{27}H_{20}ON_3F_2Pd$ [M–$(CH_3CN)_2$– BF_4^-– BF_3]+: 546.0604 found: 546.0620.

(*E*)-*N*'-(Diphenylmethylene)-3-(4-fluorophenyl)-*N*-(pyridin-2-yl)acrylohydrazide·*Pd* (2:1) Complex [(1a)2/Pd]

Complex (**1a**)2/Pd was formed immediately after the addition of CD_3CN (550 μL) into an NMR tube containing (*E*)-*N*'-(diphenylmethylene)-3-(4-fluorophenyl)-*N*-(pyridin-2-yl)acrylohydrazide (**1a**) (4.9 mg, 0.0117 mmol, 1.0 equiv) and $[Pd(CH_3CN)_4](BF_4)_2$ (2.6 mg, 0.00585 mmol, 0.5 equiv).

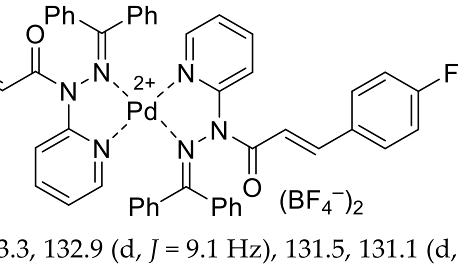

(**1a**)2/Pd

^{1}H NMR (500 MHz, 298 K, CD_3CN): δ 8.39 (d, *J* = 15.4 Hz, 2H), 8.16–8.06 (m, 4H), 8.01 (d, J = 7.1 Hz, 4H), 7.87 (m, 4H), 7.74–7.64 (m, 4H), 7.53 (t, *J* = 7.9 Hz, 4H), 7.39 (d, *J* = 7.8 Hz, 4H), 7.35 (m, 2H), 7.30 (t, *J* = 8.6 Hz, 4H), 7.15–7.09 (m, 6H), 6.77 (d, *J* = 15.4 Hz, 2H); ^{13}C NMR (125 MHz, 298 K, CD_3CN): δ 189.6, 169.4, 166.0 (d, *J* = 252.4 Hz), 153.3, 152.1, 150.7, 145.6, 136.0, 134.8, 134.5, 133.3, 132.9 (d, *J* = 9.1 Hz), 131.5, 131.1 (d, *J* = 3.2 Hz), 129.8 (3Cx2), 127.0, 121.0, 117.4 (d, *J* = 22.3 Hz), 113.6; ^{19}F NMR (376 MHz, 343 K, CD_3CN): –108.2 (m), –151.7 (s, $^{10}BF_4$), –151.8 (s, $^{11}BF_4$); HRMS (ESI) *m/z* calculated for $C_{54}H_{40}O_2N_6F_2Pd$ [M–$(BF_4^-)_2$]$^{2+}$: 474.1102 found: 474.1113.

(*E*)-*N*'-(Diphenylmethylene)-*N*-(3-methylpyridin-2-yl)but-2-enehydrazide·*Pd* (2:1) Complex [(1b)2/Pd]

Complex (**1b**)2/Pd was formed immediately after the addition of CD_3CN (550 μL) into an NMR tube containing (*E*)-*N*'-(diphenylmethylene)-*N*-(3-methylpyridin-2-yl)but-2-enehydrazide (**1b**) (4.2 mg, 0.0117 mmol, 1.0 equiv) and $[Pd(CH_3CN)_4](BF_4)_2$ (2.6 mg, 0.00585 mmol, 0.5 equiv).

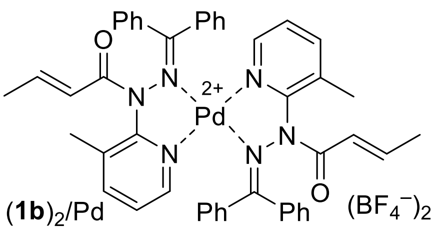

(**1b**)2/Pd

^{1}H NMR (500 MHz, 298 K, CD_3CN): δ 8.04–7.94 (m, 4H), 7.89–7.88 (m, 2H), 7.84–7.83 (m, 2H), 7.81–7.72 (m, 2H), 7.72–7.65 (m, 2H), 7.57–7.51 (m, 4H), 7.44–7.37 (m, 6H), 7.25–7.20 (m, 6H), 5.99 (d, *J* = 14.9 Hz, 2H), 2.15 (dd, *J* = 7.0, 1.7 Hz, 6H), 1.82 (s, 6H); ^{13}C NMR (125 MHz, 298 K, CD_3CN): δ; 189.8, 169.0, 155.2, 152.1, 149.8, 147.1, 135.2, 135.0, 134.4, 133.7, 132.4, 131.4, 129.8, 129.7, 129.6, 127.3, 119.3, 19.0, 18.4; HRMS (ESI) *m/z* calculated for $C_{46}H_{42}O_2N_6Pd$ [M–$(BF_4^-)_2$]$^{2+}$: 408.1197 found: 408.1206.

(*E*)-*N*'-(Diphenylmethylene)-*N*-[3-(2,6-dimethylphenyl)pyridin-2-yl]but-2-enehydrazide·*Pd* (2:1) Complex [(1c)2/Pd]

Complex (**1c**)2/Pd was formed immediately after the addition of CD_3CN (550 μL) into an NMR tube containing (*E*)-*N*'-(diphenylmethylene)-*N*-[3-(2,6-dimethylphenyl)pyridin-2-yl]but-2-enehydrazide (**1c**) (5.2 mg, 0.0117 mmol, 1.0 equiv) and $[Pd(CH_3CN)_4](BF_4)_2$ (2.6 mg, 0.00585 mmol, 0.5 equiv).

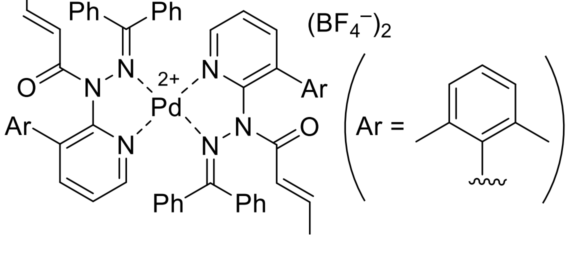

^{1}H NMR (500 MHz, 298 K, CD_3CN): δ 8.88 (br s, 2H), 7.98 (dd, *J* = 5.7, 1.5 Hz, 2H), 7.88–7.74 (m, 6H), 7.51–7.41 (m, 6H), 7.39–7.27 (m, 8H), 7.28–7.23 (m, 4H), 7.22–7.15 (m, 4H), 6.59 (d, *J* = 7.0 Hz, 2H), 6.36 (d, *J*

= 15.0 Hz, 2H), 2.16 (dd, *J* = 7.0, 1.6 Hz, 6H), 2.14 (s, 6H), 0.79 (s, 6H); ^{13}C NMR (125 MHz, 298 K, CD_3CN): δ 187.9, 171.0, 156.6, 153.2, 151.5, 147.8, 137.8, 137.1, 136.6, 136.4, 134.9, 134.5, 134.1 (br), 133.8, 133.5, 132.7, 130.3 (br), 130.2, 130.05, 130.02, 129.0, 128.5, 127.0, 119.8, 21.3, 20.3, 19.3; HRMS (ESI) *m*/*z* calculated for $C_{60}H_{54}O_2N_6Pd$ $[M-(BF_4^-)_2]^{2+}$: 498.1666 found: 498.1670.

(*E*)-*N*'-(Diphenylmethylene)-3-(4-fluorophenyl)-*N*-(pyridin-2-yl)acrylohydrazide·*Zn* (2:1) Complex [(1a)$_2$/Zn]

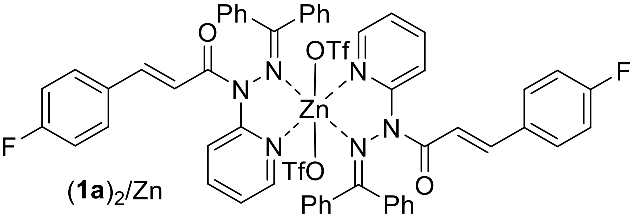

(**1a**)$_2$/Zn

Complex (**1a**)$_2$/Zn was formed immediately after the addition of CD_3CN (550 μL) into an NMR tube containing (*E*)-*N*'-(diphenylmethylene)-3-(4-fluorophenyl)-*N*-(pyridin-2-y l)acrylohydrazide (**1a**) (4.9 mg, 0.0117 mmol, 1.0 equiv) and $Zn(OTf)_2$ (2.1 mg, 0.00585 mmol, 0.5 equiv).

^{1}H NMR (500 MHz, 298 K, CD_3CN): δ 8.24 (br s, 2H), 7.99 (s, 2H), 7.62–7.35 (m, 22H), 7.29 (t, *J* = 7.7 Hz, 4H), 7.11 (t, *J* = 8.7 Hz, 4H), 7.09–6.94 (m, 6H); ^{13}C NMR (125 MHz, 298 K, CD_3CN): δ 181.1, 166.4, 165.2 (d, *J* = 248.8 Hz), 154.1, 148.2, 146.5, 142.6, 136.6, 135.4, 134.0, 132.0 (d, *J* = 8.8 Hz), 131.8, 131.6 (d, *J* = 3.8 Hz), 131.0, 129.6, 129.4, 129.3, 123.8, 121.9 (d, *J* = 318.8 Hz), 121.1, 117.3, 117.0 (d, *J* = 22.5 Hz); ^{19}F NMR (376 MHz, 343 K, CD_3CN):–78.3 (s), –110.2 (br s); HRMS (ESI) *m*/*z* calculated for $C_{55}H_{40}O_5N_6F_5SZn$ $[M-(OTf^-)]^+$: 1055.1987 found: 1055.1993.

2-6. Synthesis of ester **2**

Methyl (*E*)-3-(4-fluorophenyl)acrylate (2)

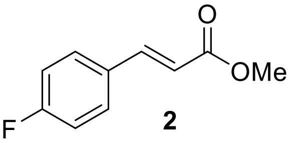

2

Note that ester **2** *is known compound.*[5]

To a mixture of (*E*)-*N*'-((*E*)-benzylidene)-3-(4-fluorophenyl)-*N*-(pyridin-2-yl)acrylohydrazide (**1a**) (42.1 mg, 0.10 mmol 1.0 equiv) and $Zn(OTf)_2$ (1.8 mg, 0.005 mmol, 5 mol%) was added MeOH (9.4 mL) at room temperature. After stirred for 24 h, the reaction mixture was concentrated under reduced pressure. Purification of the residue by flash column chromatography (hexane/EtOAc, 19:1) gave title compound **2** (16.5 mg, 92% yield).

^{1}H NMR (400 MHz, 298 K, $CDCl_3$): δ 7.66 (d, *J* = 16.0 Hz, 1H), 7.57–7.42 (m, 2H), 7.16–6.96 (m, 2H), 6.36 (d, *J* = 16.0 Hz, 1H), 3.81 (s, 3H); ^{13}C NMR (100 MHz, 298 K, $CDCl_3$): δ .167.5, 164.05 (d, *J* = 251.5 Hz), 143.7, 130.8 (d, J = 3.3 Hz), 130.08 (d, *J* = 8.5 Hz), 117.69 (d, *J* = 2.4 Hz), 116.20 (d, *J* = 22.0 Hz), 51.9; ^{19}F NMR (376 MHz, 298 K, $CDCl_3$): –109.6 (m).

3. NMR Analyses of Amide–Metal Complexes

*3-1. **1a**–Pd Complexes*

*3-1-1. Detailed NMR of **1a** for peak assignments*

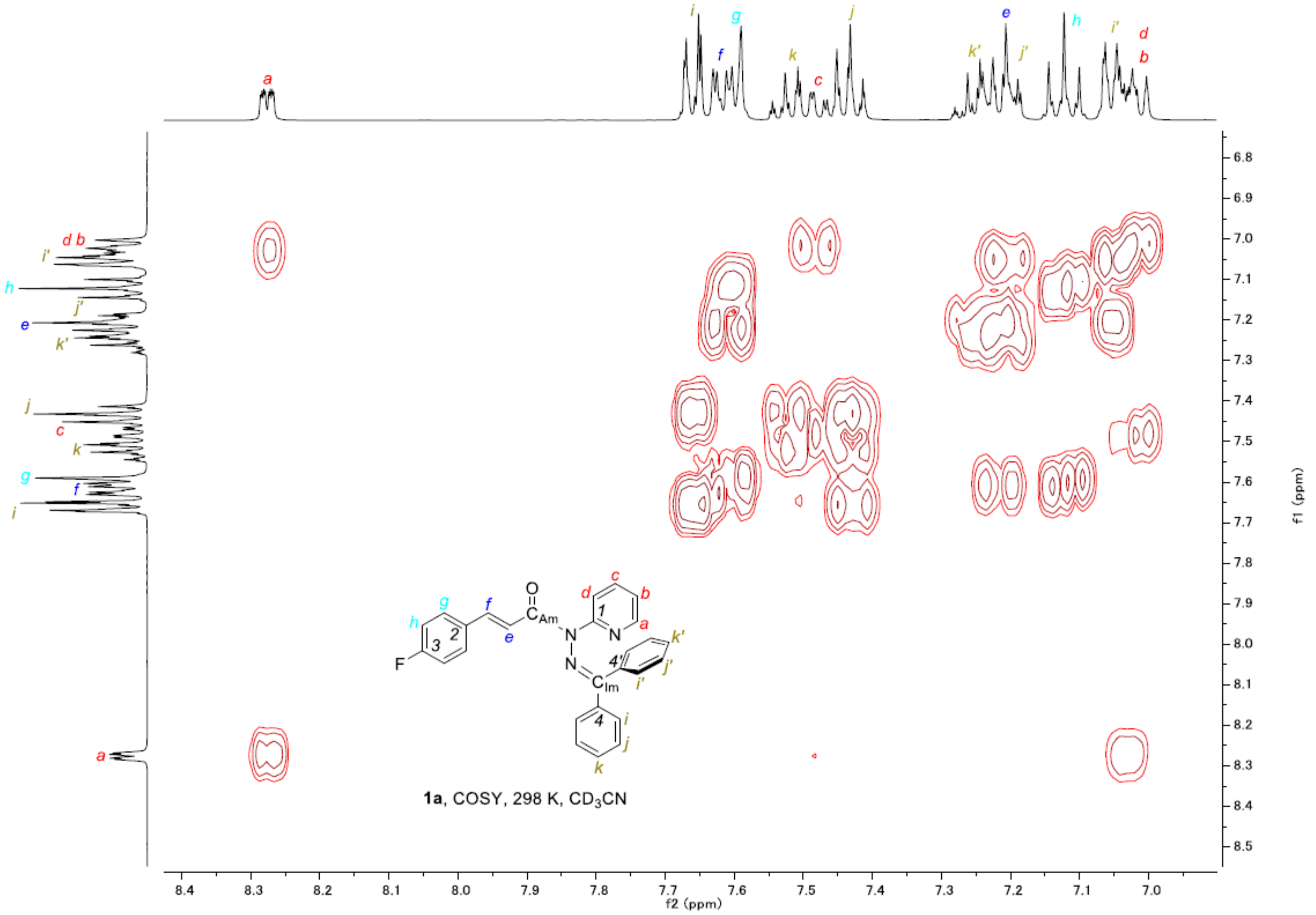

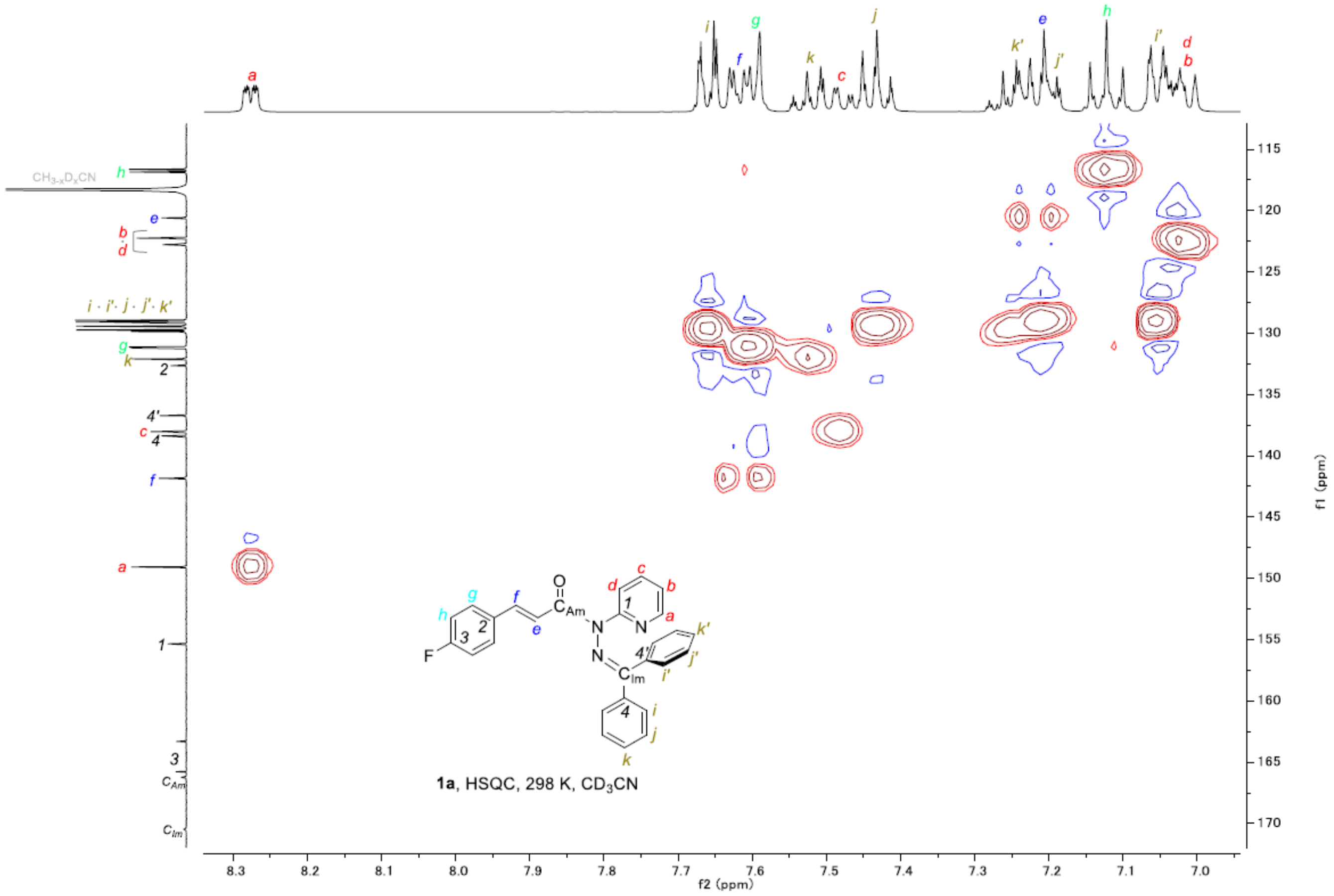

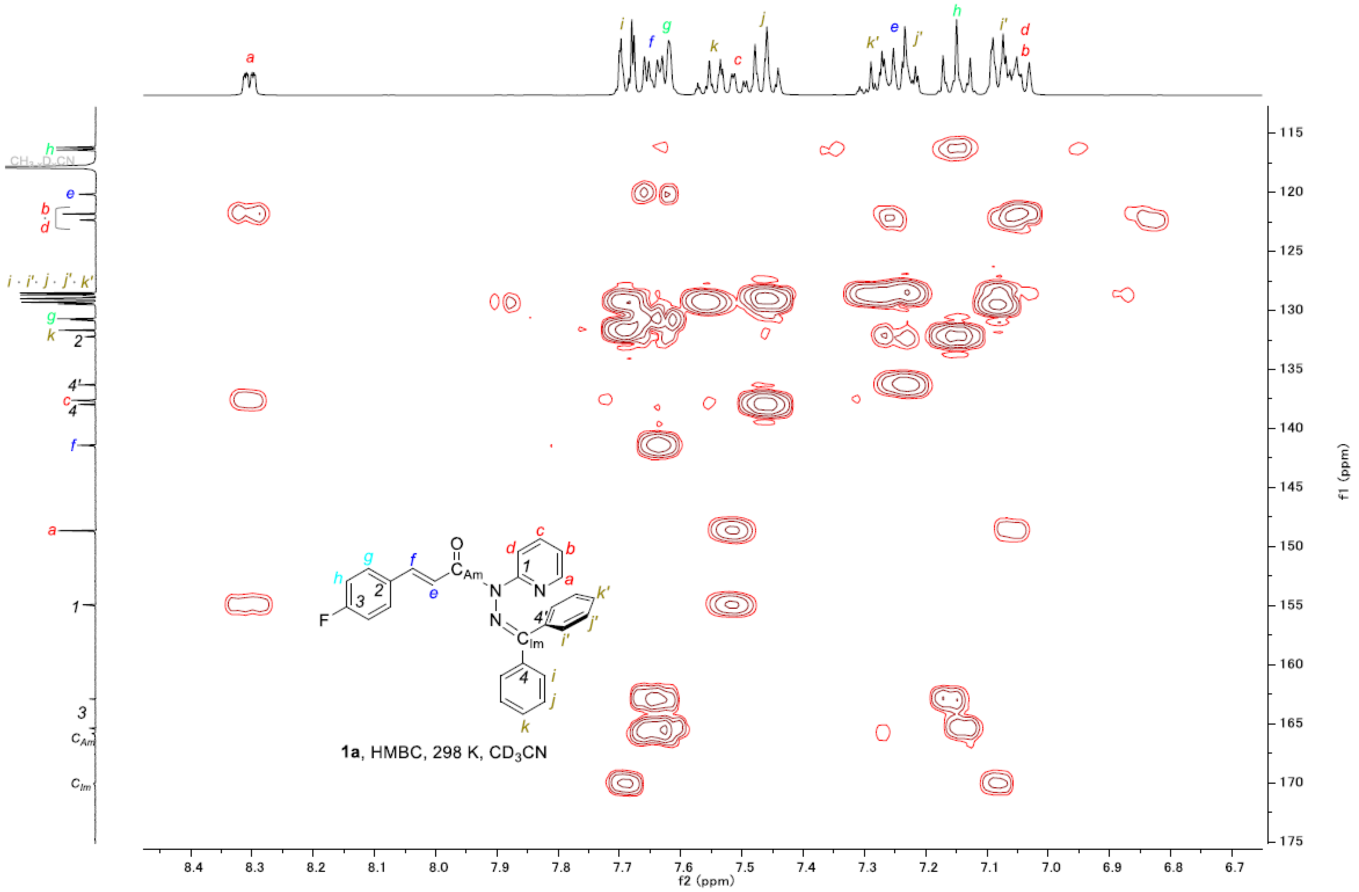
1a, HMBC, 298 K, CD3CN
f2 (ppm)
f1 (ppm)

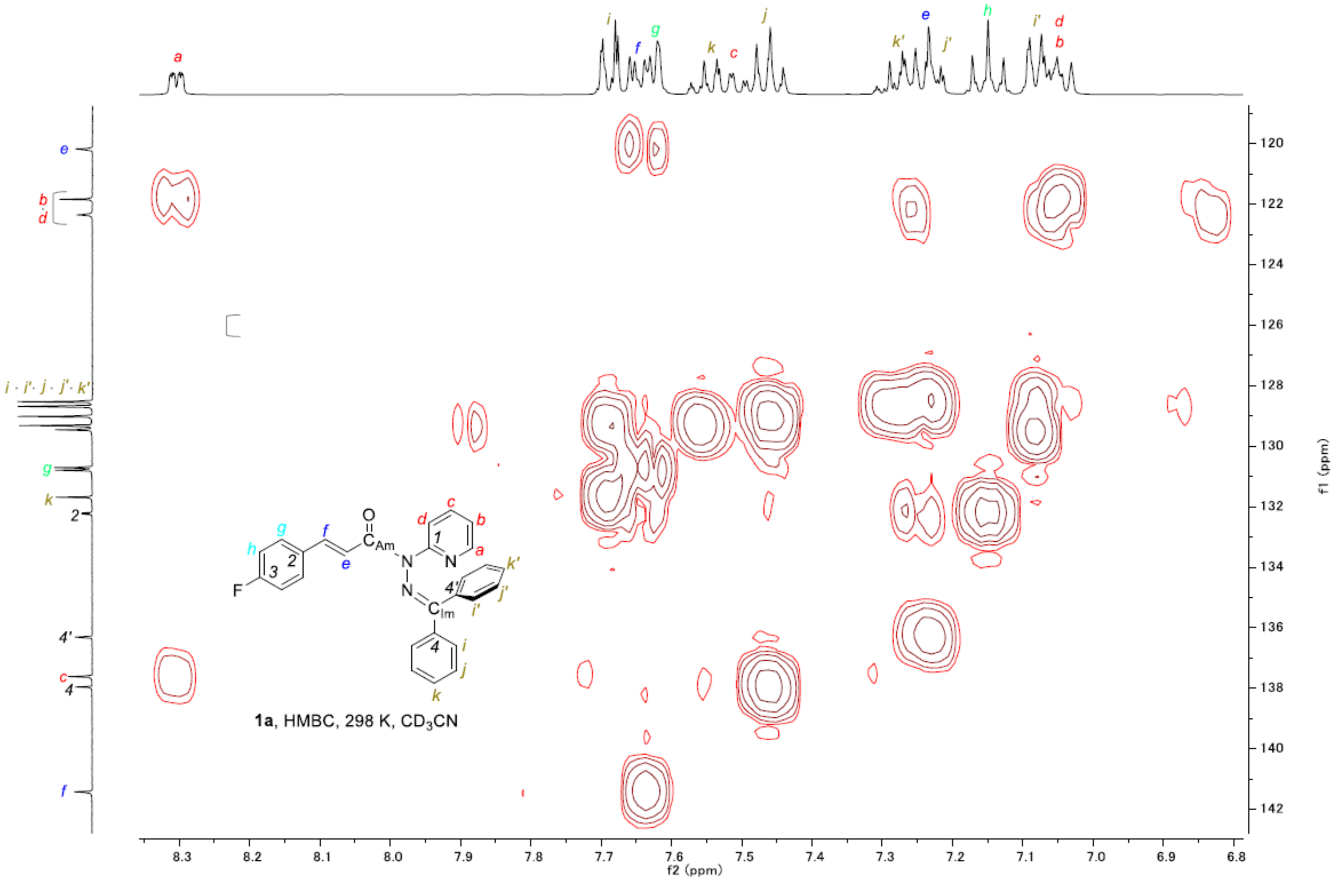
1a, HMBC, 298 K, CD3CN
f2 (ppm)
f1 (ppm)

3-1-2. Detailed NMR of **1a**/Pd for peak assignments

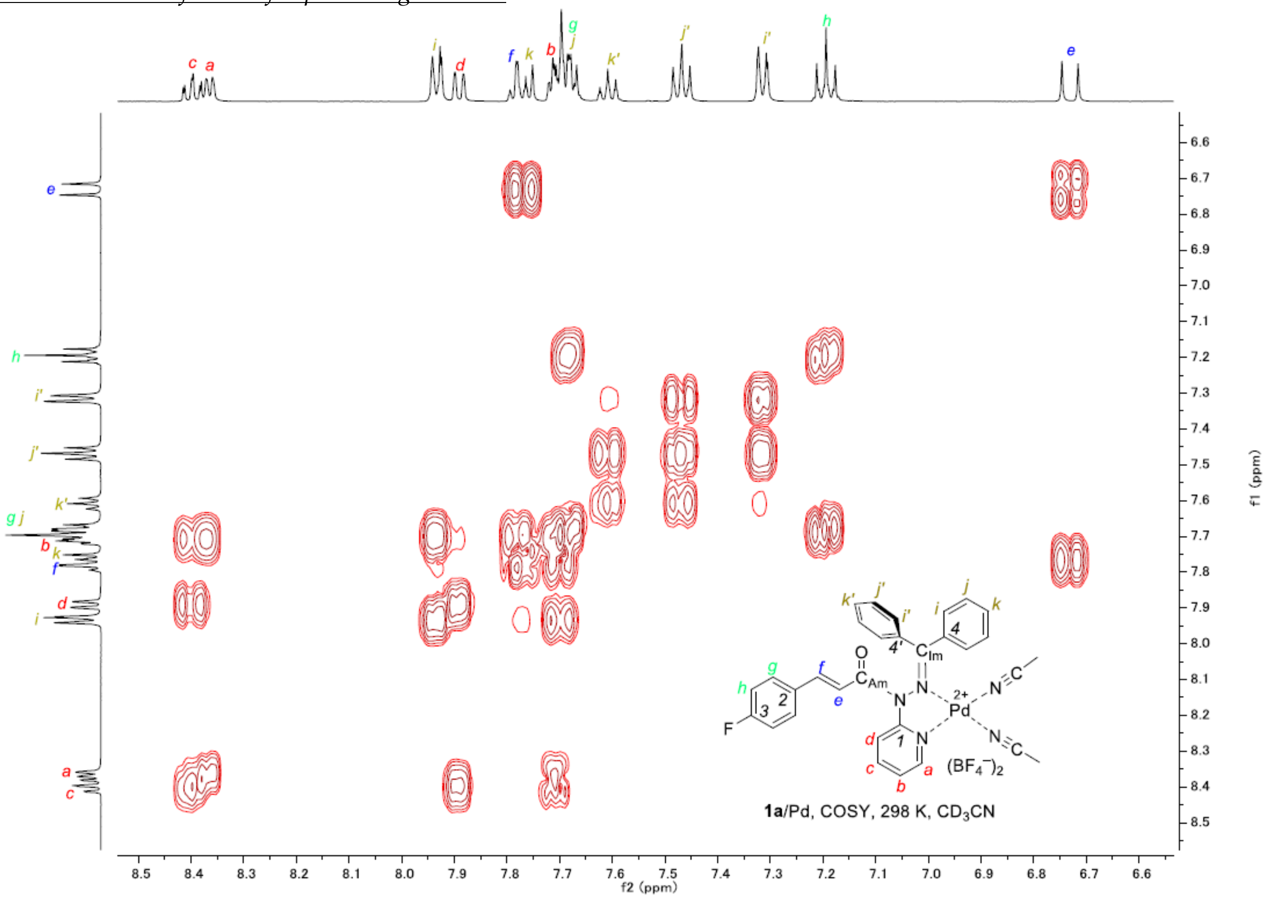

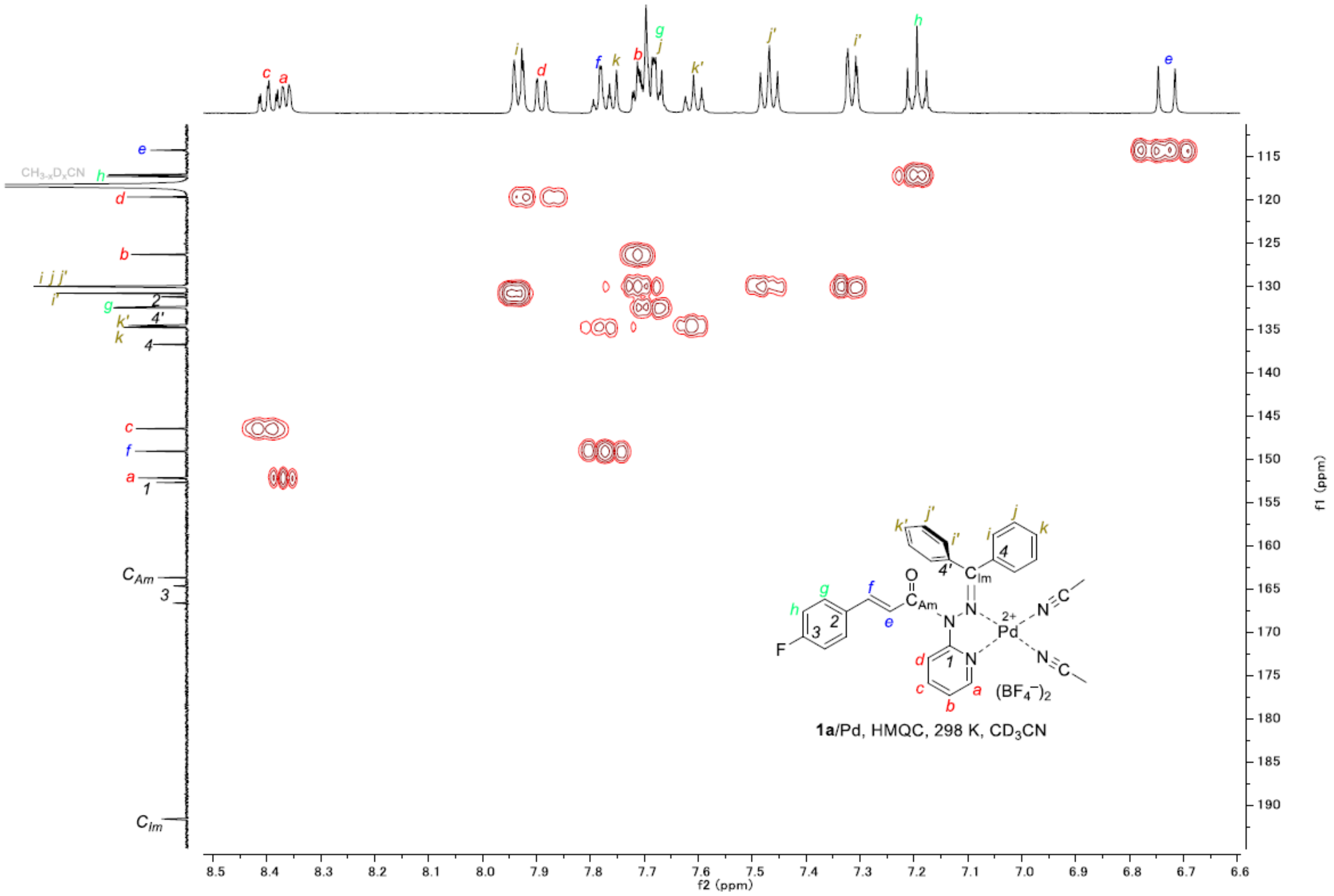

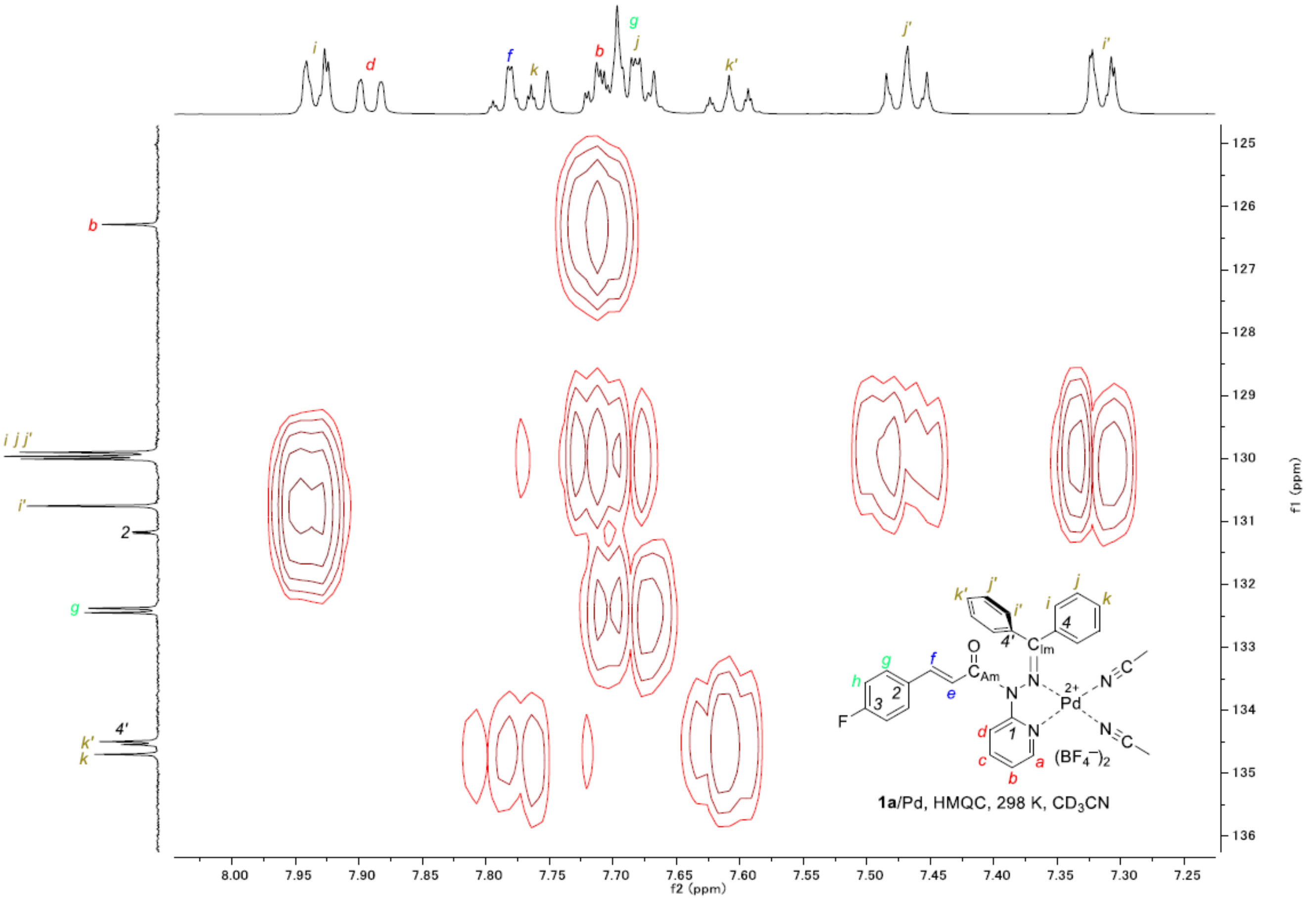
1a/Pd, HMQC, 298 K, CD3CN
f2 (ppm)
f1 (ppm)

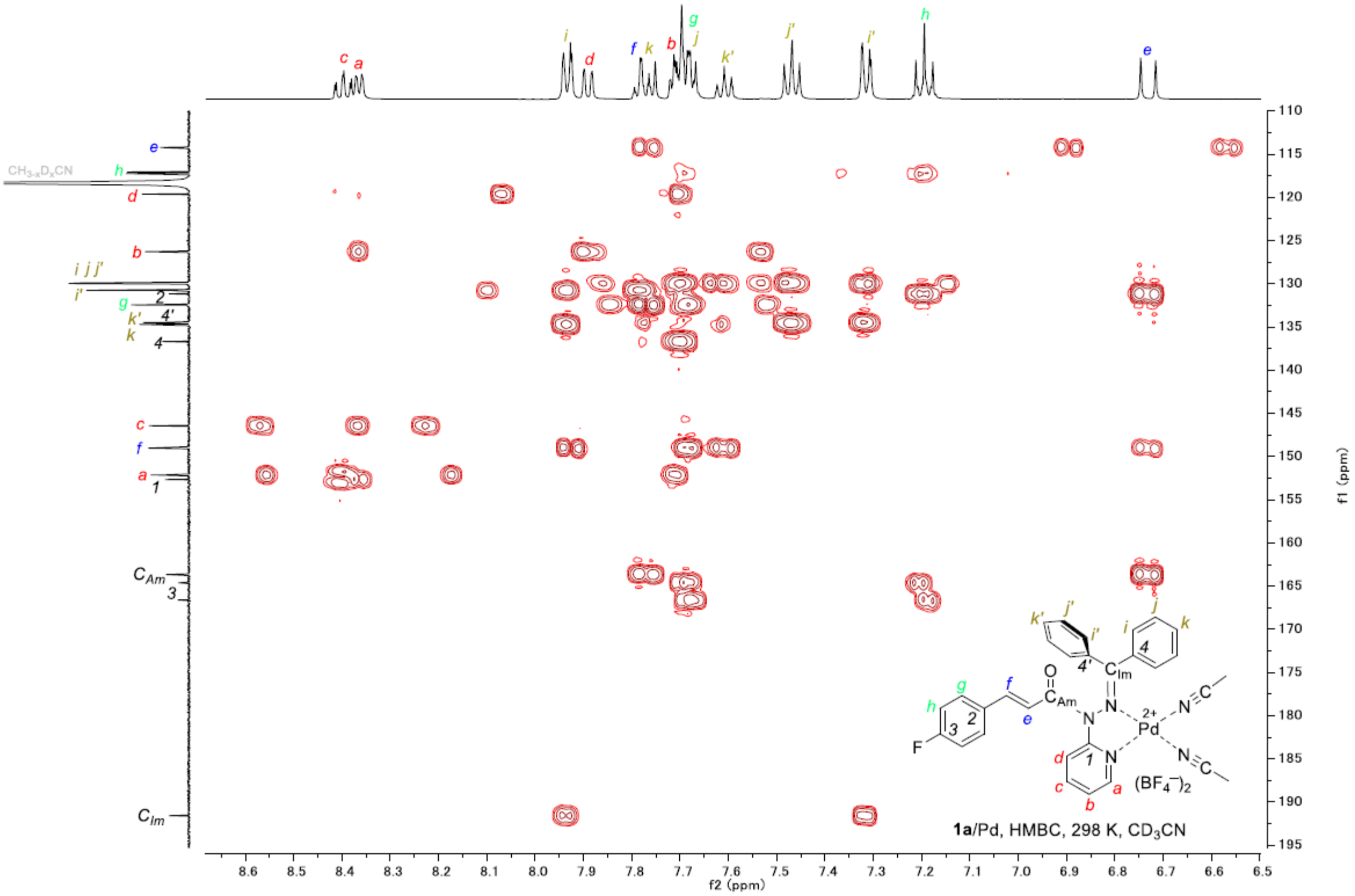
1a/Pd, HMBC, 298 K, CD3CN
f2 (ppm)
f1 (ppm)

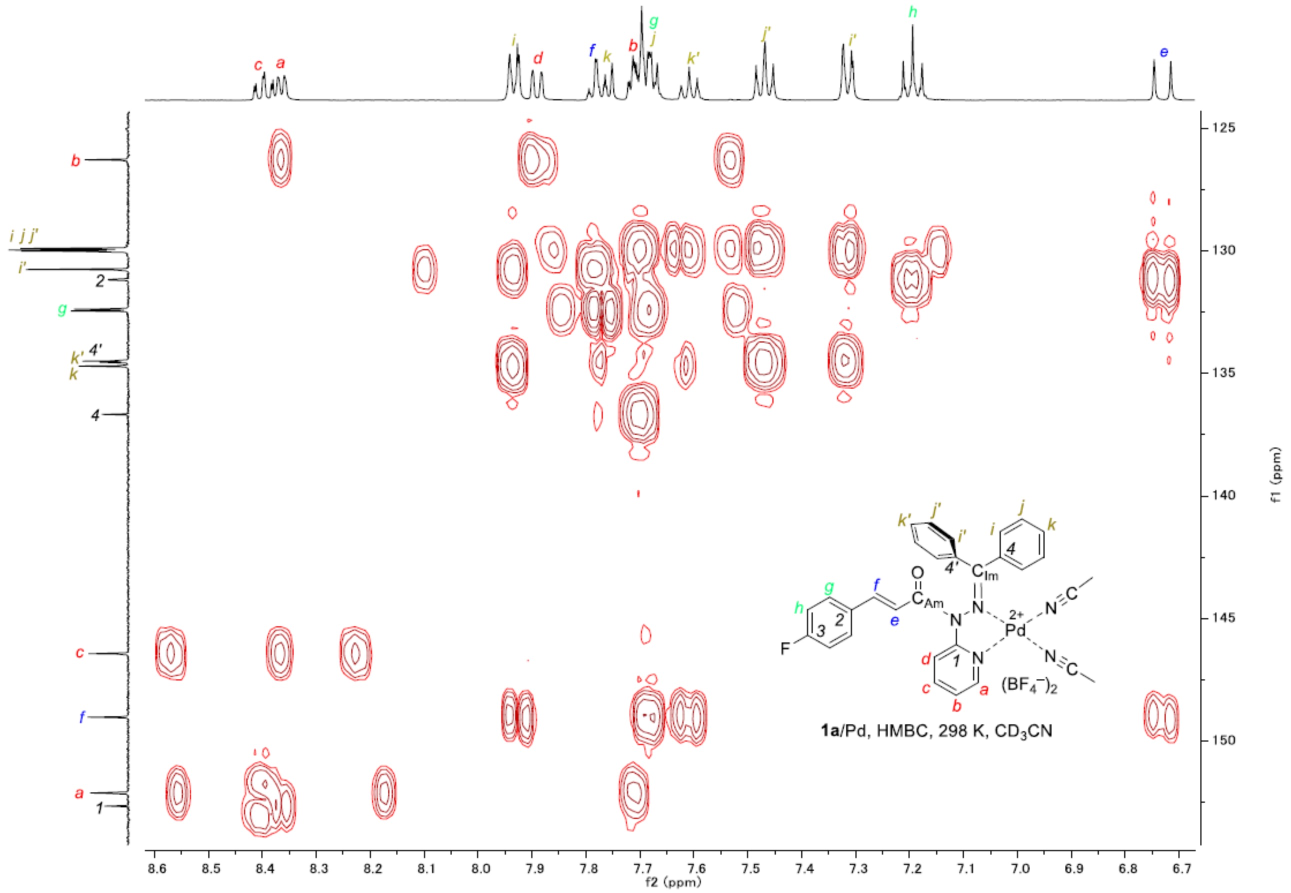
1a/Pd, HMBC, 298 K, CD3CN
f2 (ppm)
f1 (ppm)

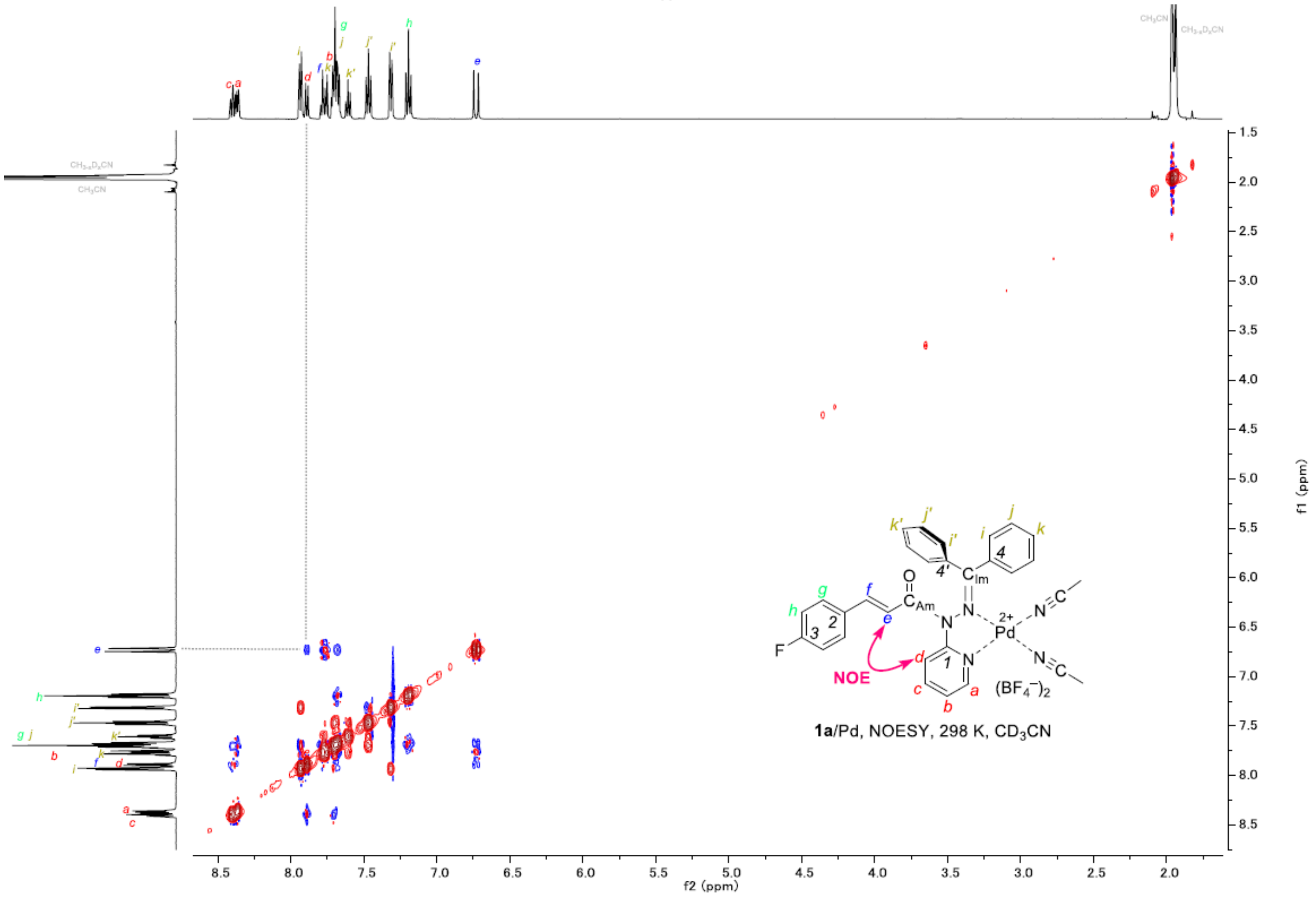
NOE
1a/Pd, NOESY, 298 K, CD3CN
f2 (ppm)
f1 (ppm)

3-1-3. Detailed NMR of (**1a**)$_2$/Pd for peak assignments

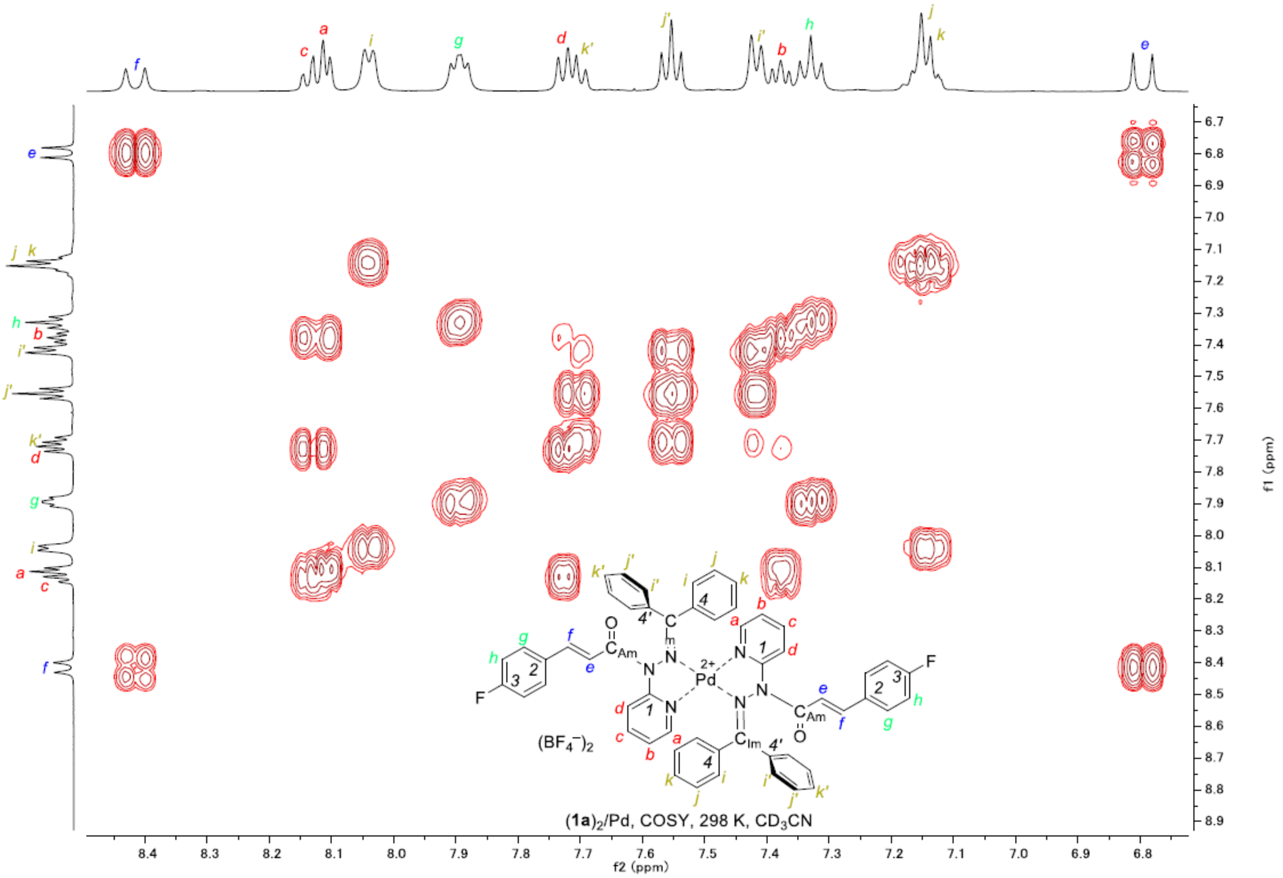

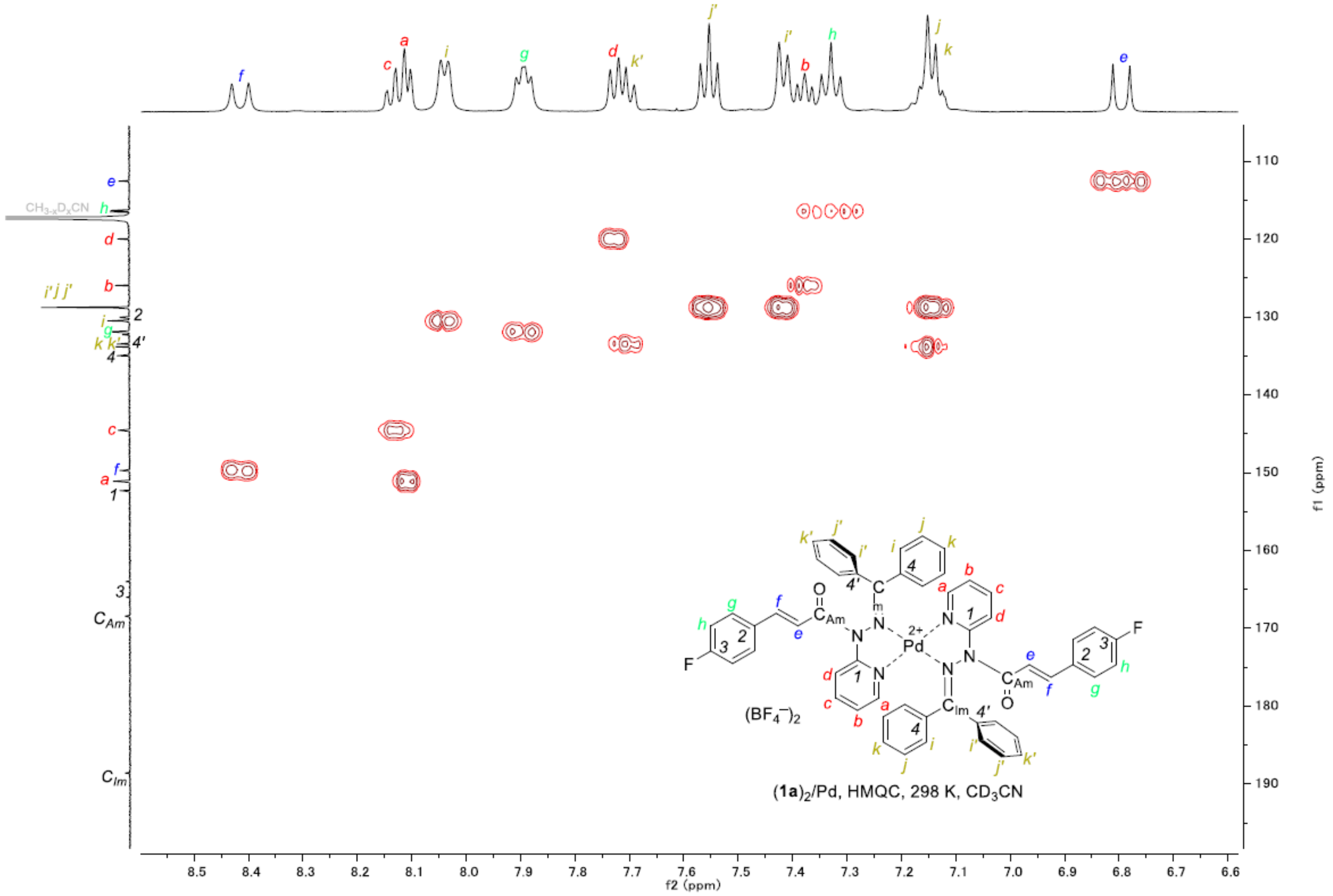

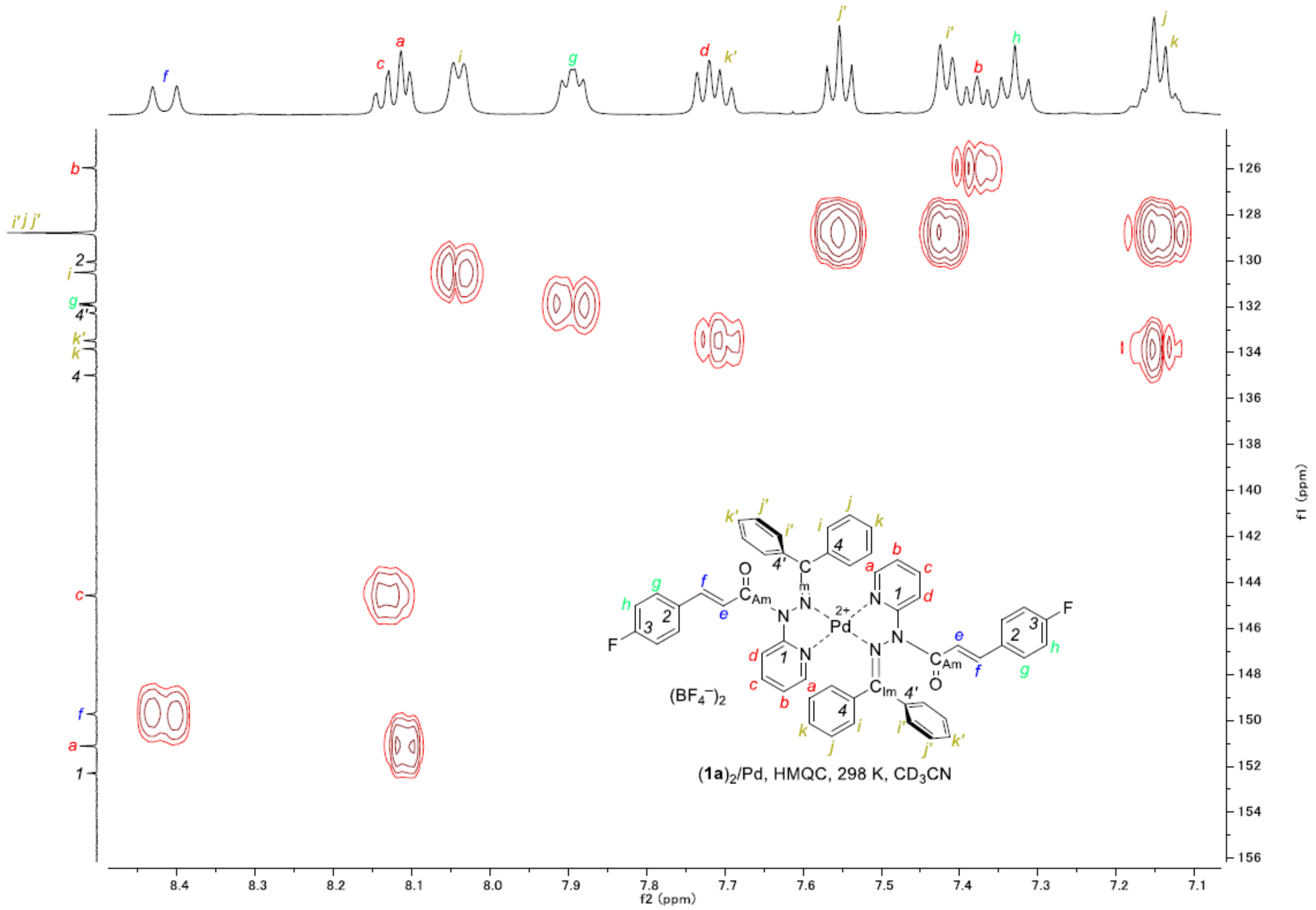
(1a)2/Pd, HMQC, 298 K, CD3CN
(BF4−)2
f2 (ppm)
f1 (ppm)

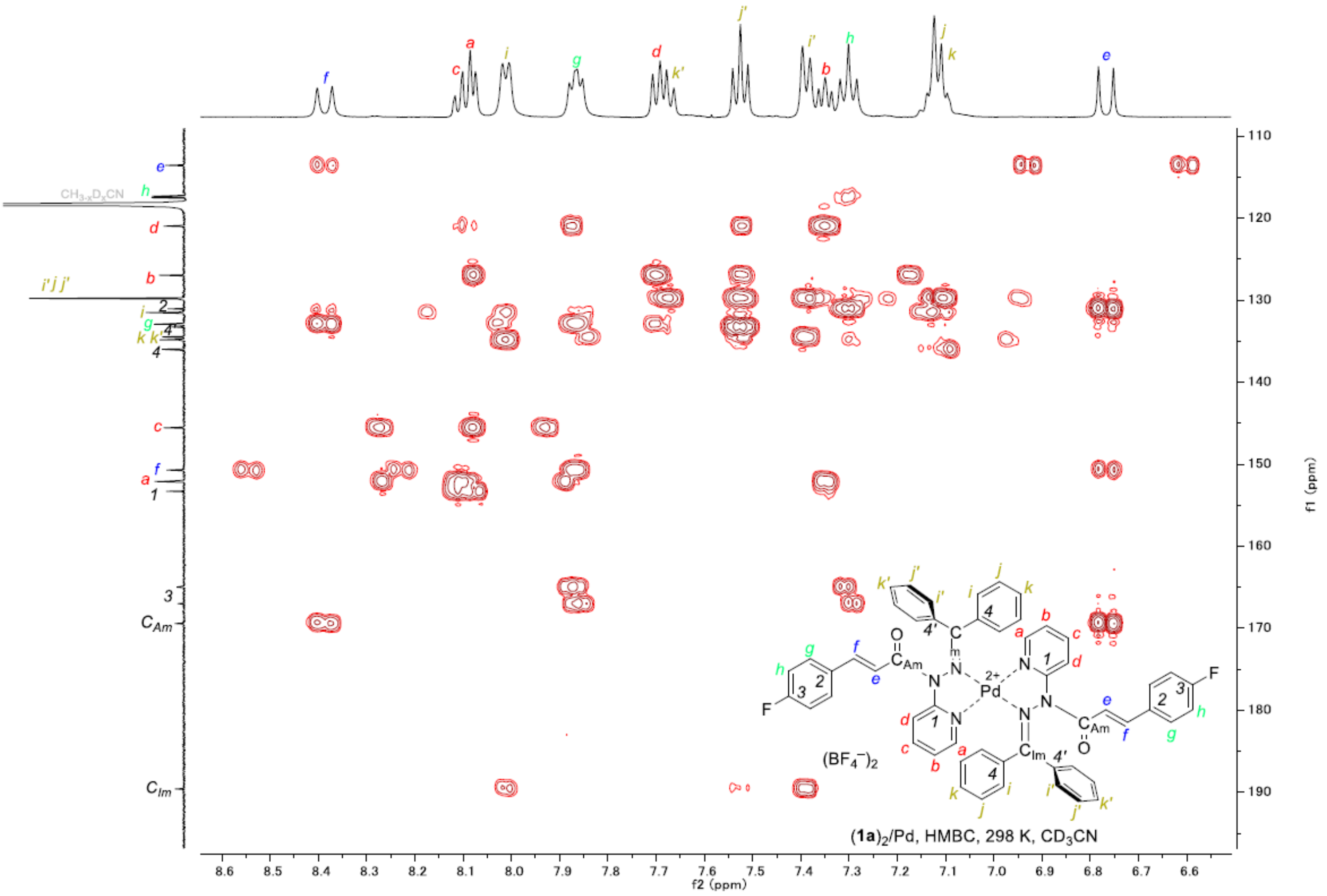
(1a)2/Pd, HMBC, 298 K, CD3CN
(BF4−)2
CH3-xDxCN
f2 (ppm)
f1 (ppm)

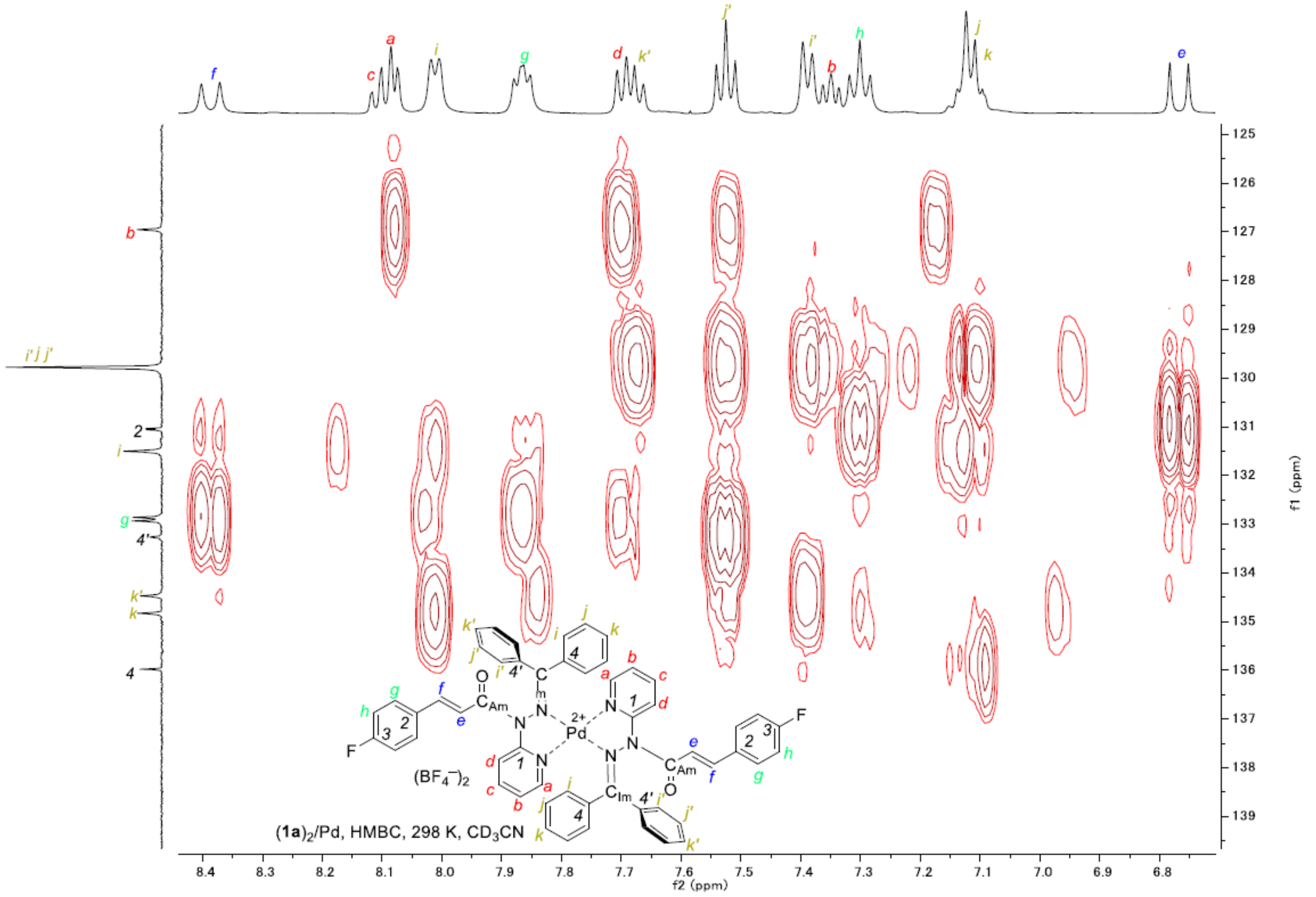
(1a)2/Pd, HMBC, 298 K, CD3CN
$(BF_4^-)_2$
f2 (ppm)
f1 (ppm)

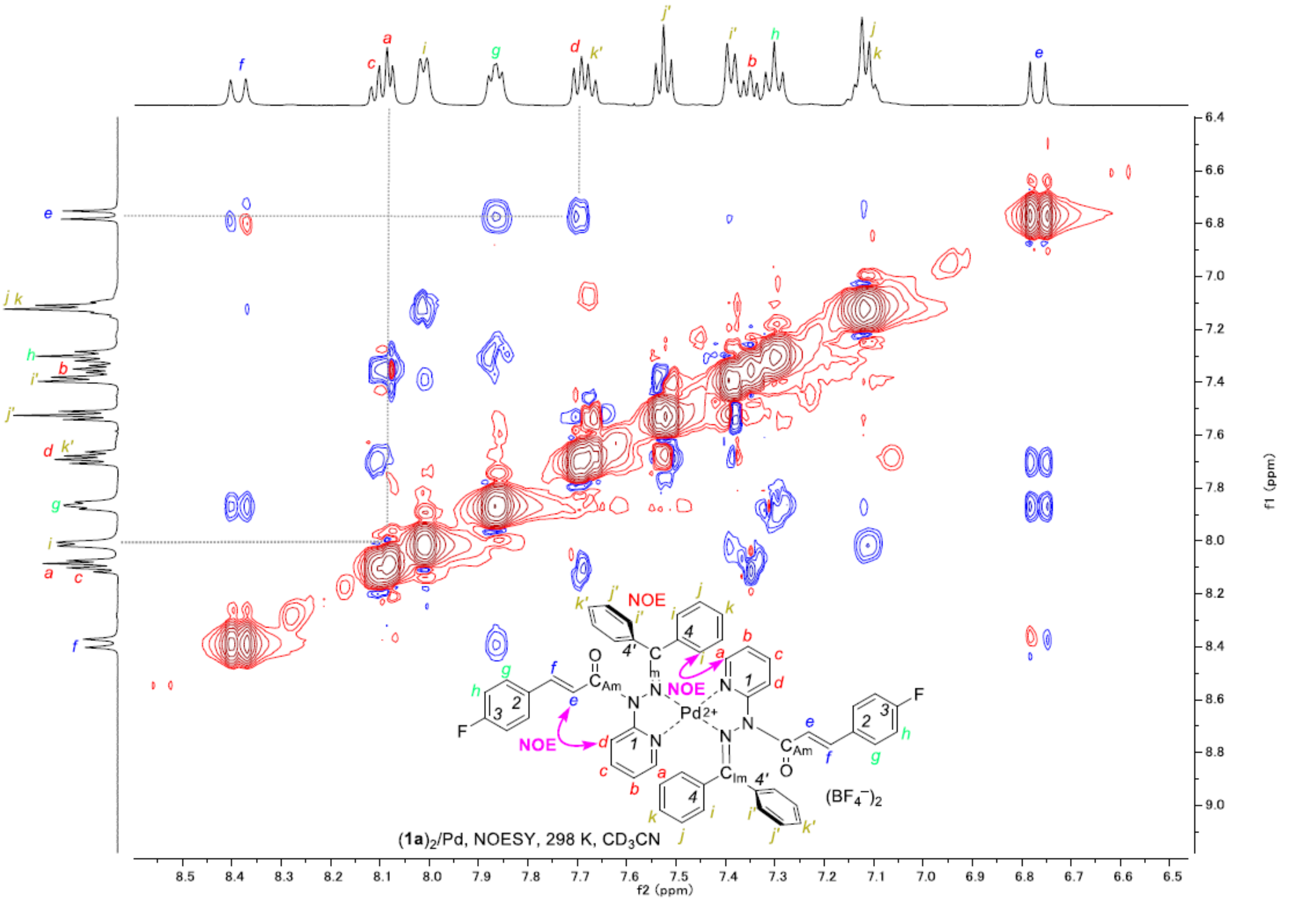
(1a)2/Pd, NOESY, 298 K, CD3CN
NOE
$(BF_4^-)_2$
f2 (ppm)
f1 (ppm)

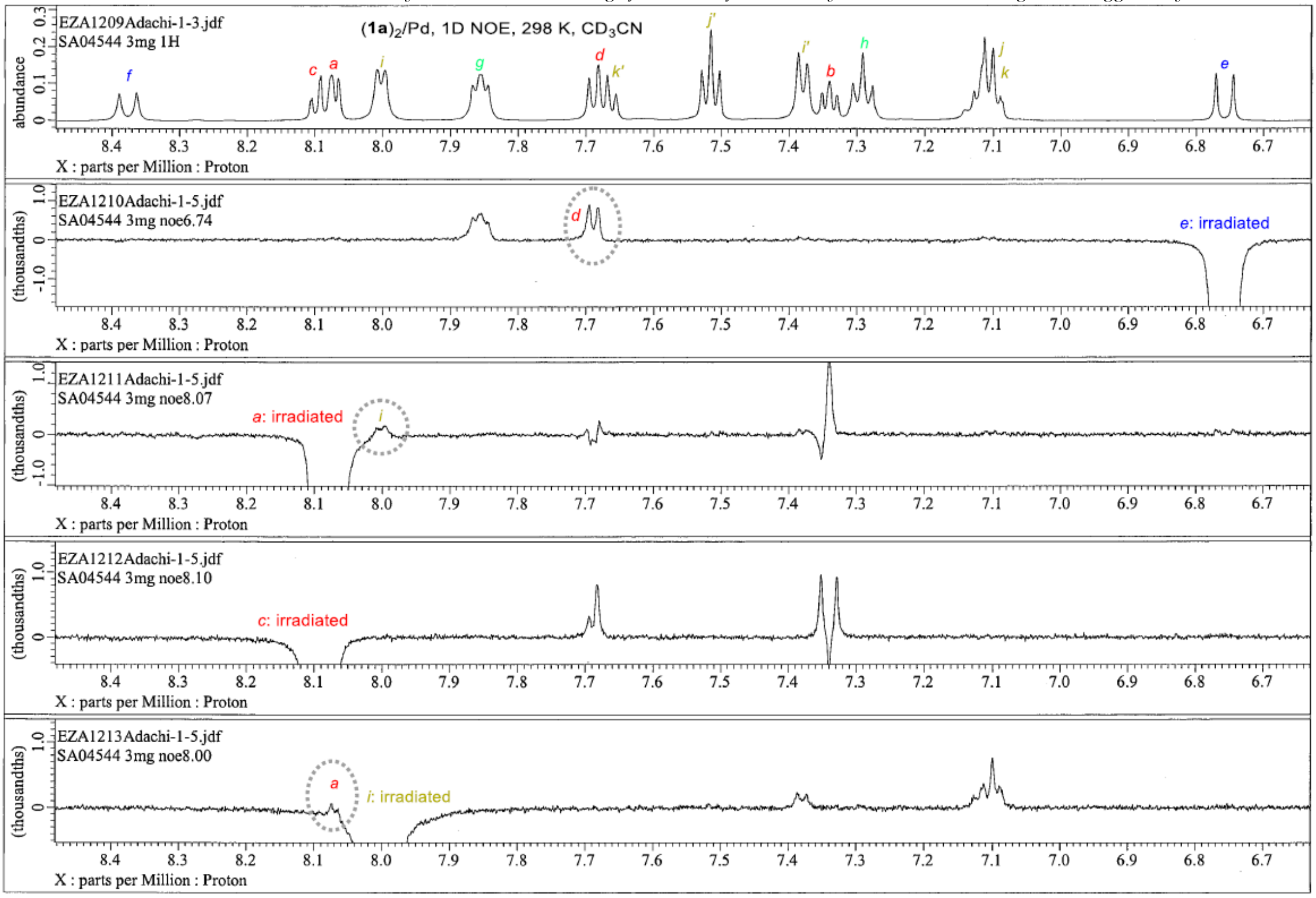
EZA1209Adachi-1-3.jdf
SA04544 3mg 1H
(1a)2/Pd, 1D NOE, 298 K, CD3CN
abundance
X : parts per Million : Proton
EZA1210Adachi-1-5.jdf
SA04544 3mg noe6.74
e: irradiated
(thousandths)
X : parts per Million : Proton
EZA1211Adachi-1-5.jdf
SA04544 3mg noe8.07
a: irradiated
(thousandths)
X : parts per Million : Proton
EZA1212Adachi-1-5.jdf
SA04544 3mg noe8.10
c: irradiated
(thousandths)
X : parts per Million : Proton
EZA1213Adachi-1-5.jdf
SA04544 3mg noe8.00
i: irradiated
(thousandths)
X : parts per Million : Proton

3-1-4. Stacked ^{1}H and ^{13}C NMR of **1a**, **1a**/Pd, and (**1a**)$_2$/Pd

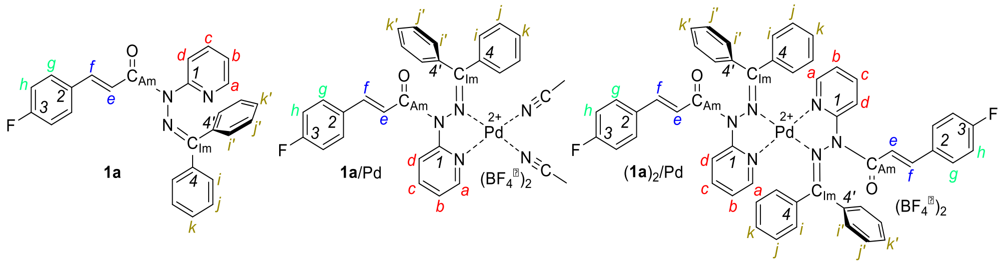

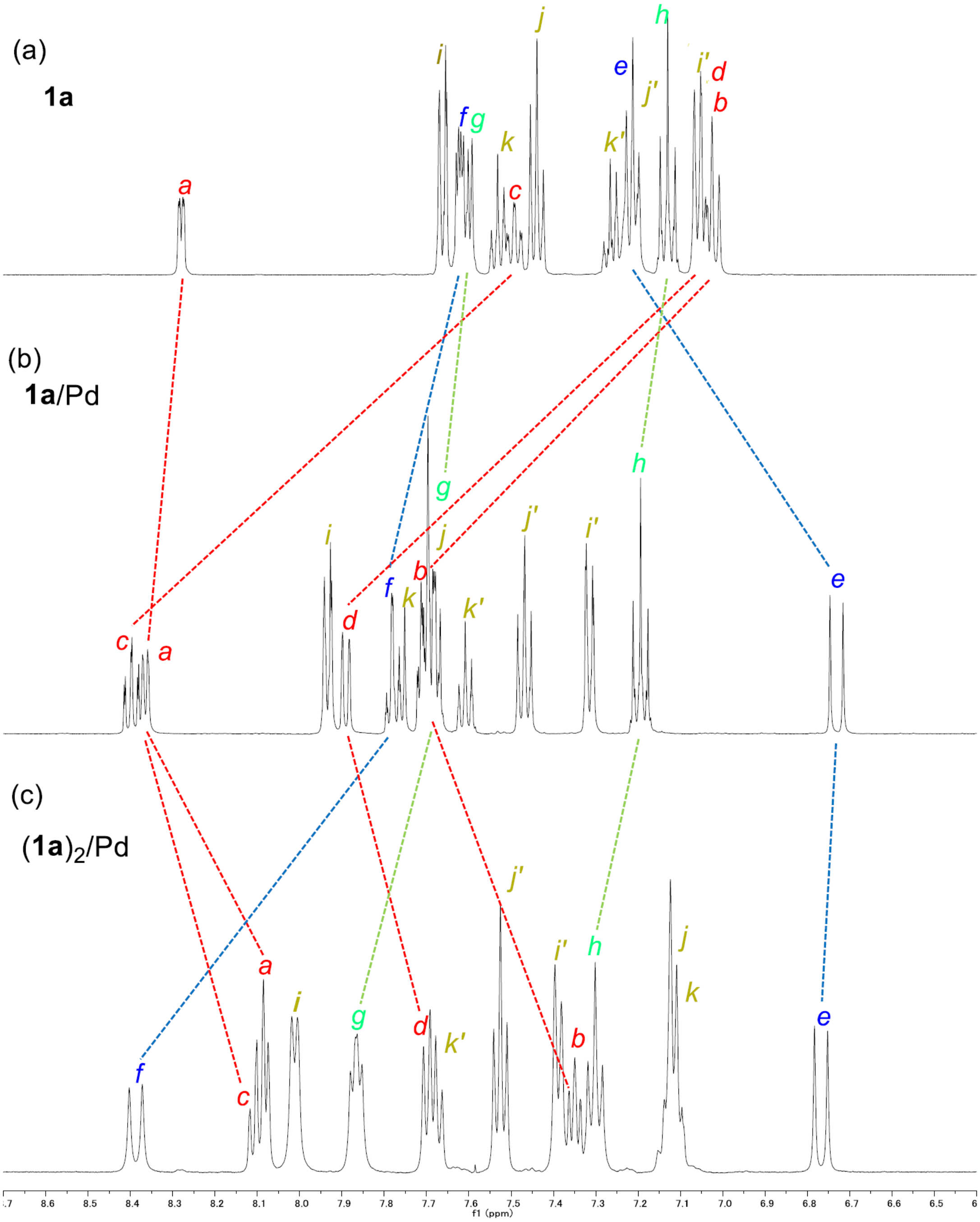

Figure S1. (a) ^{1}H NMR of **1a** in CD_3CN. (b) ^{1}H NMR of **1a**/[Pd(CH_3CN)$_4$](BF_4)$_2$ = 1/1 in CD_3CN. (c) ^{1}H NMR of **1a**/[Pd(CH_3CN)$_4$](BF_4)$_2$ = 2/1 in CD_3CN. *i*•*j*•*k* and *i'*•*j'*•*k'* are not distinguished in (a) and (b).

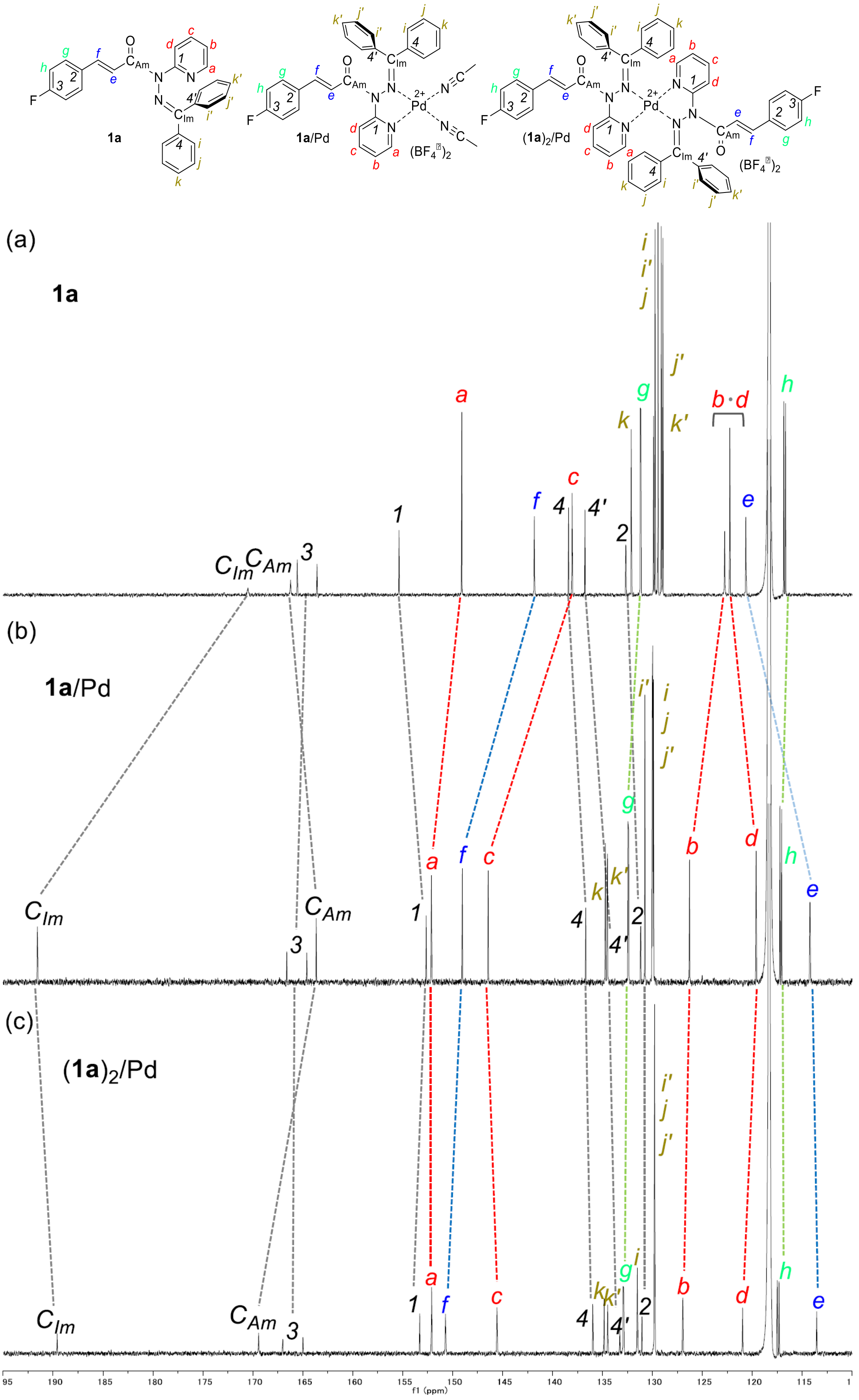

Figure S2. (a) ^{13}C NMR of **1a** in CD_3CN. (b) ^{13}C NMR of **1a**/[Pd(CH_3CN)$_4$](BF_4)$_2$ = 1/1 in CD_3CN. (c) ^{13}C NMR of **1a**/[Pd(CH_3CN)$_4$](BF_4)$_2$ = 2/1 in CD_3CN. *i*▪*j*▪*k*▪*4* and *i′*▪*j′*▪*k′*▪*4′* are not distinguished in (a) and (b).

3-2. ***1b***–Pd Complex

3-2-1. Detailed NMR of **1b** for peak assignments

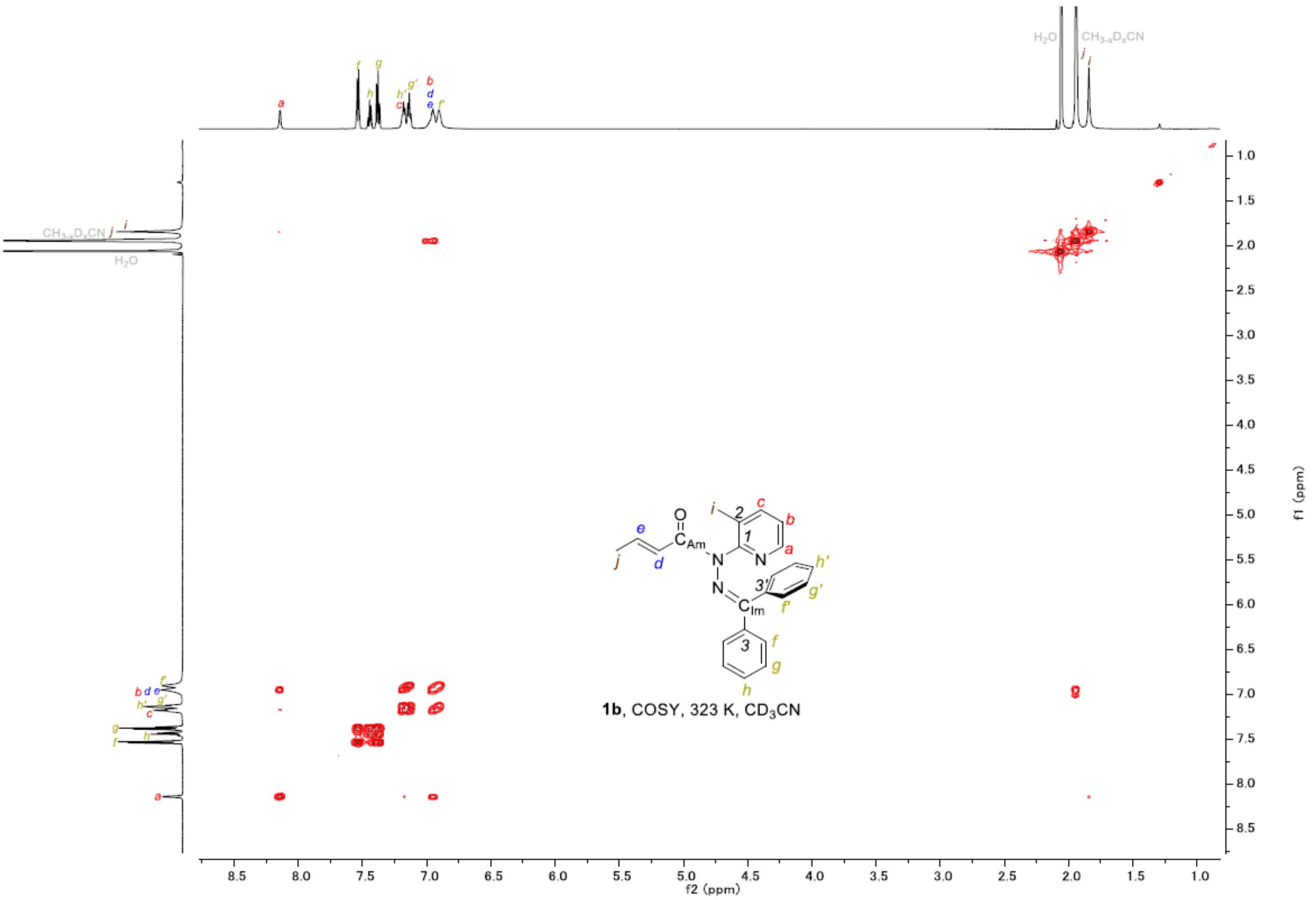

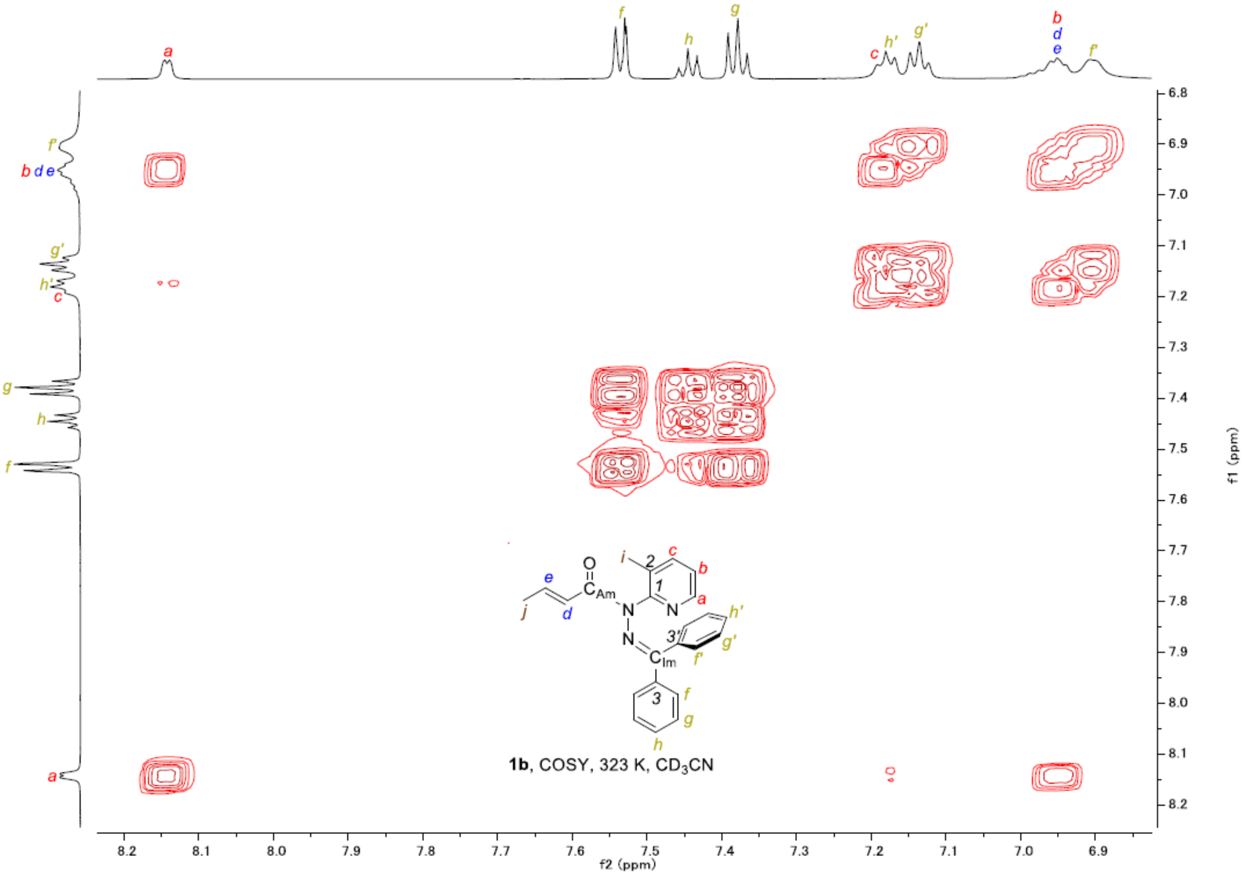

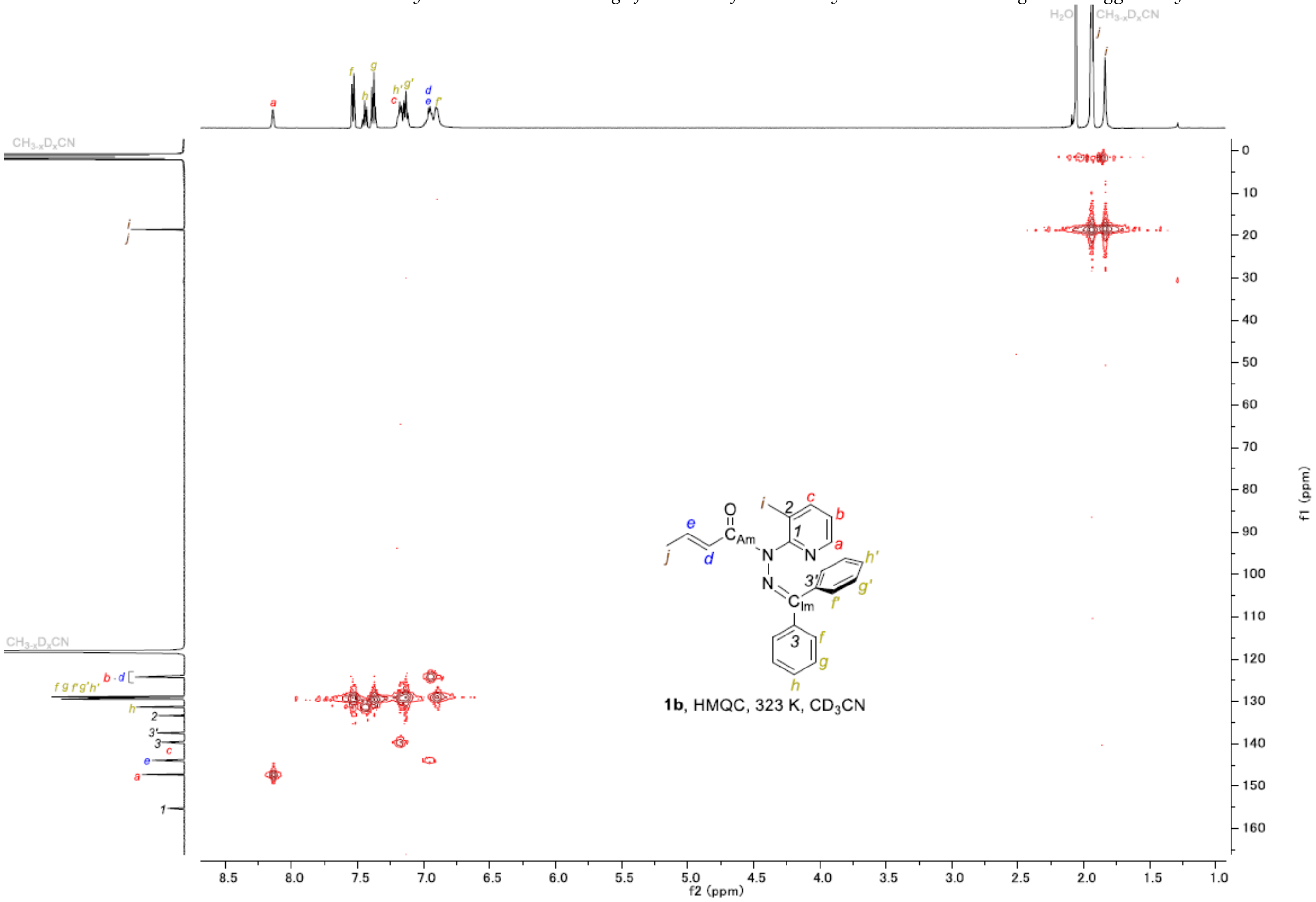
1b, HMQC, 323 K, CD_3CN
f2 (ppm)
f1 (ppm)
H2O
CH3-xDxCN

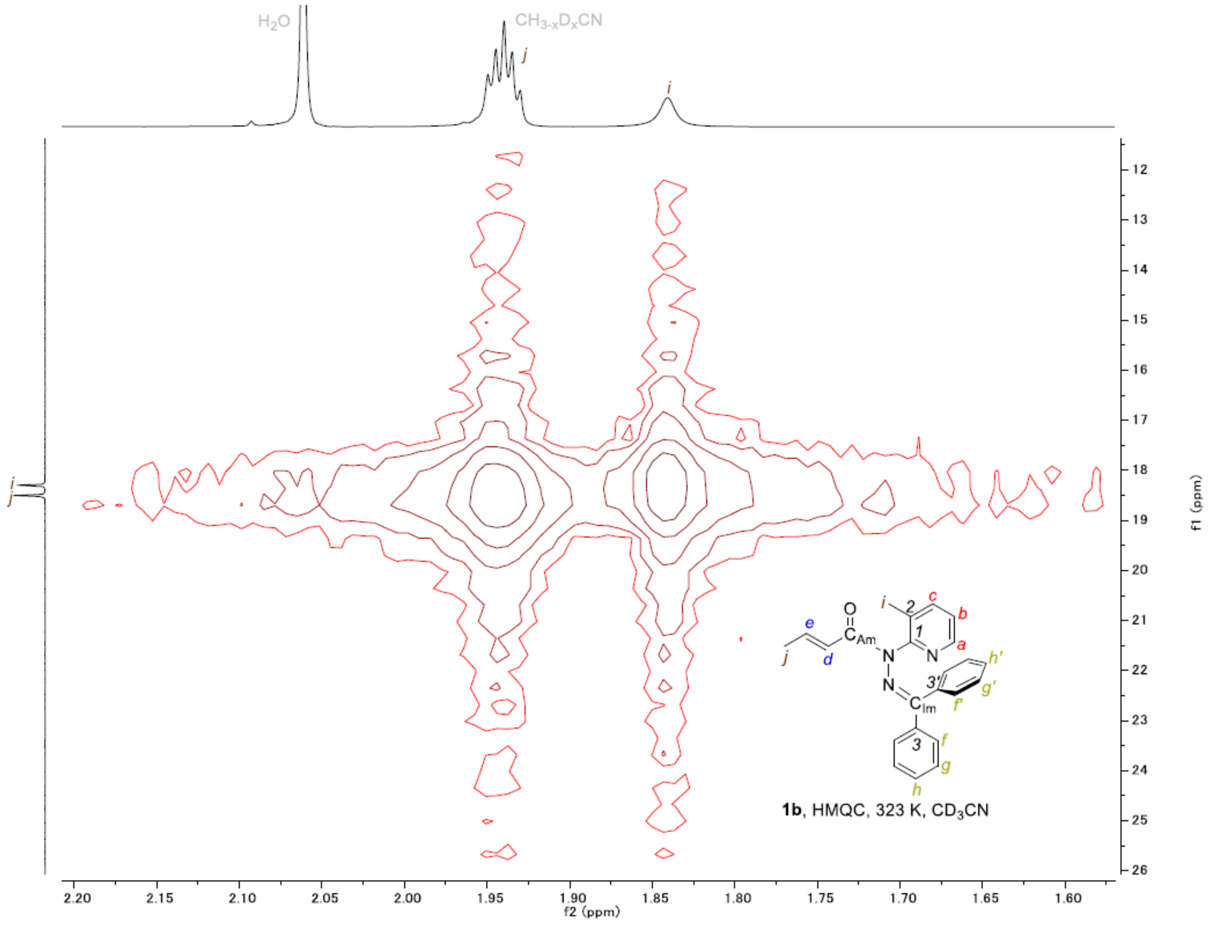
1b, HMQC, 323 K, CD_3CN
f2 (ppm)
f1 (ppm)
H2O
CH3-xDxCN

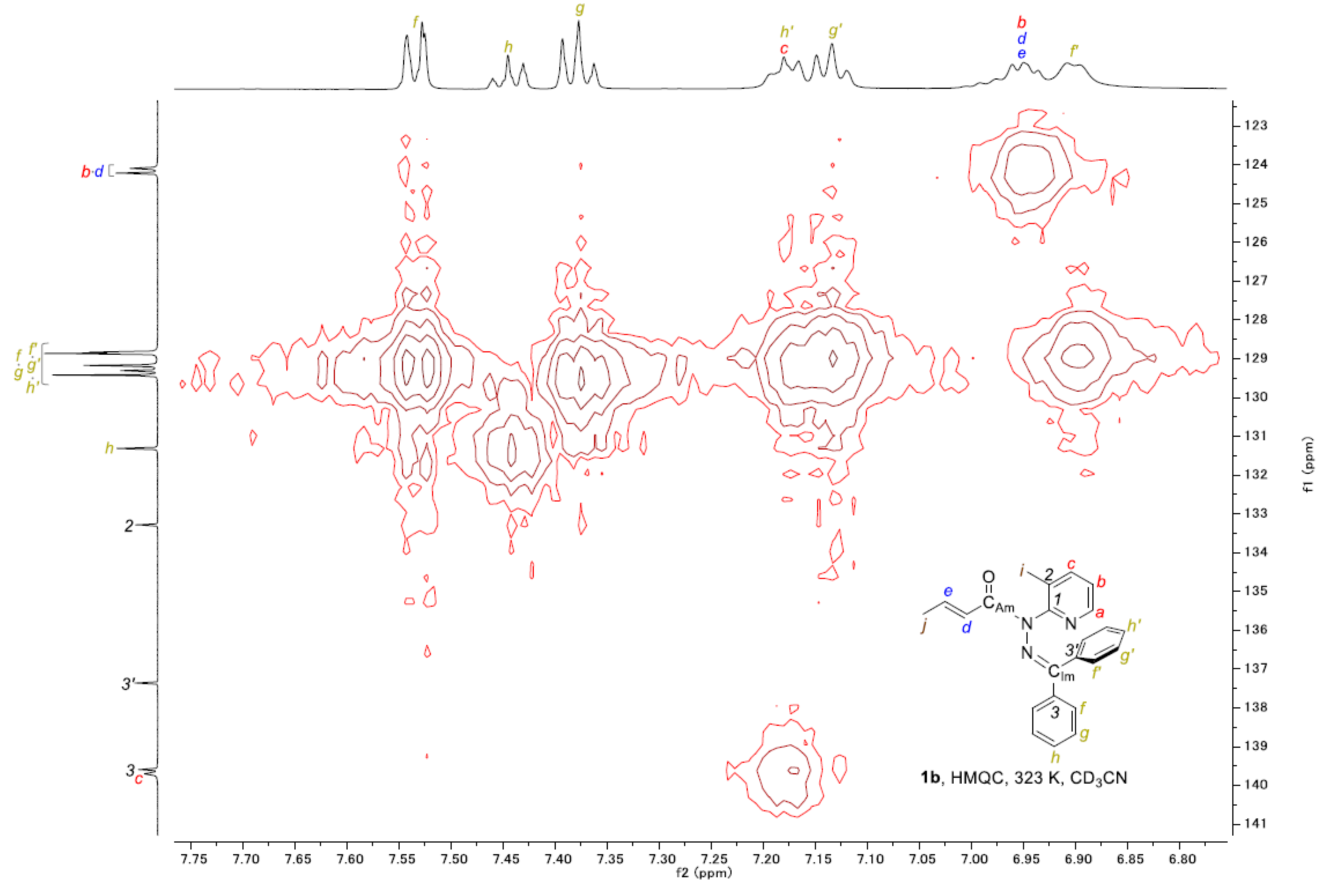
1b, HMQC, 323 K, CD3CN
f2 (ppm)
f1 (ppm)

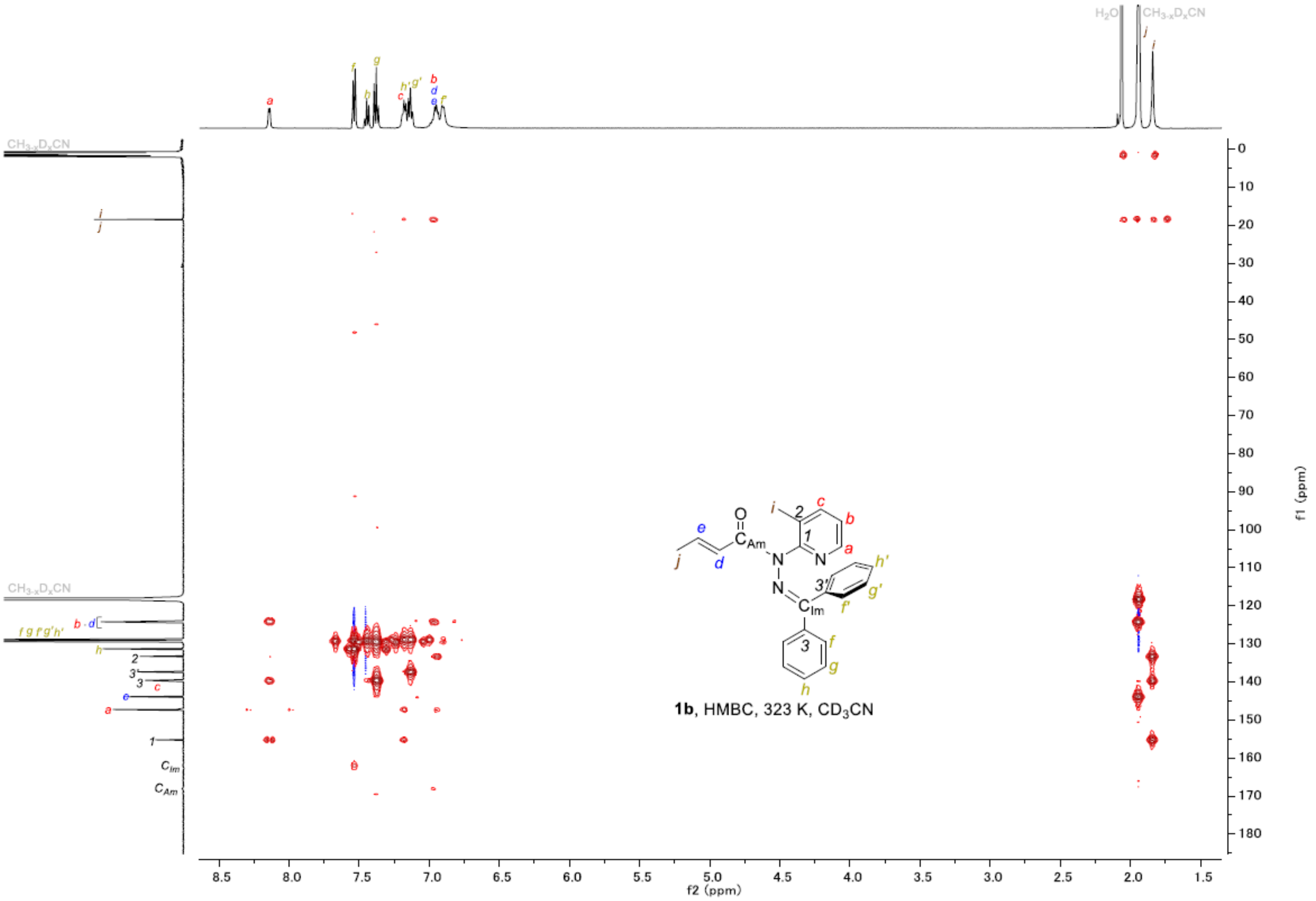
1b, HMBC, 323 K, CD3CN
f2 (ppm)
f1 (ppm)

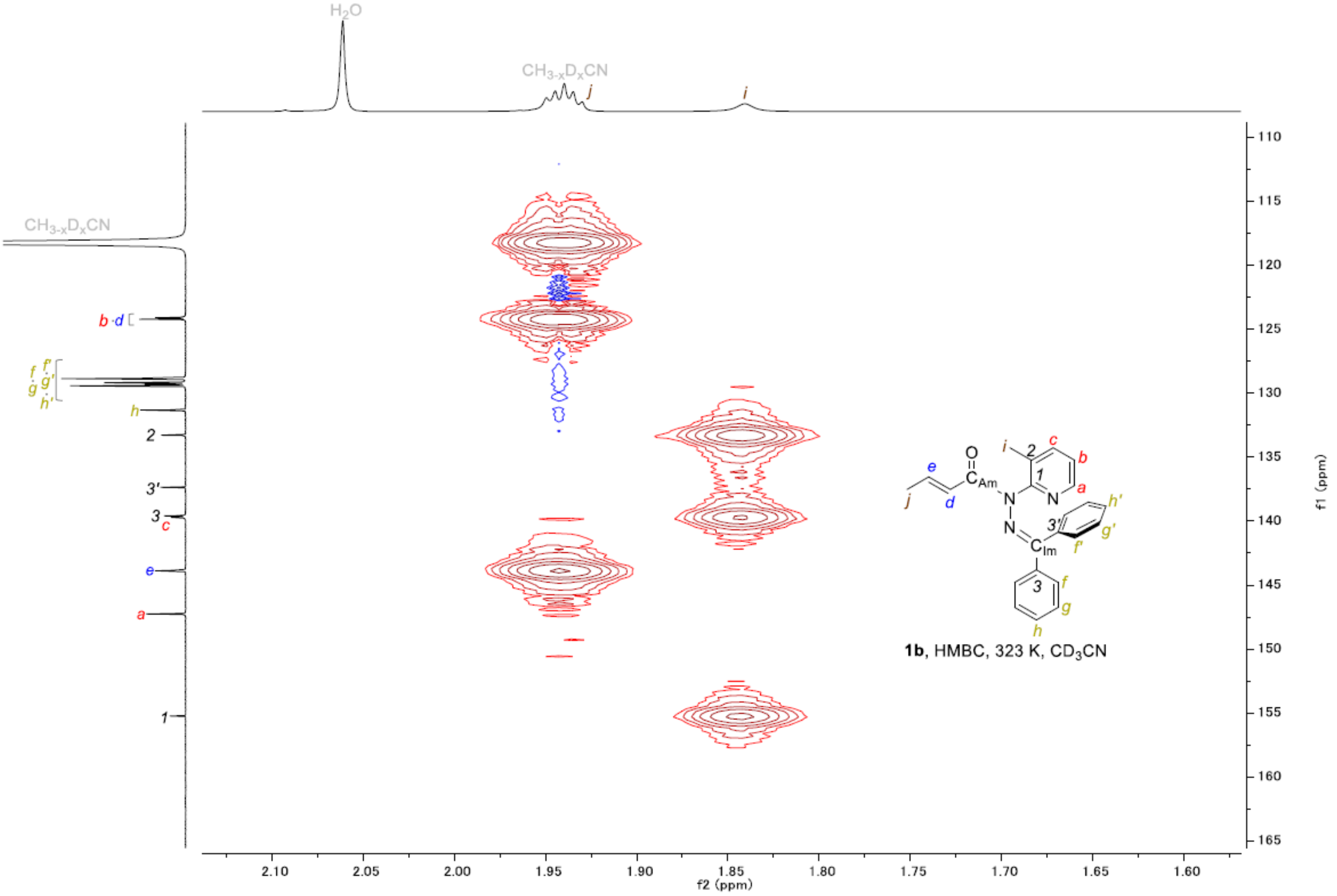
1b, HMBC, 323 K, CD3CN
f2 (ppm)
f1 (ppm)

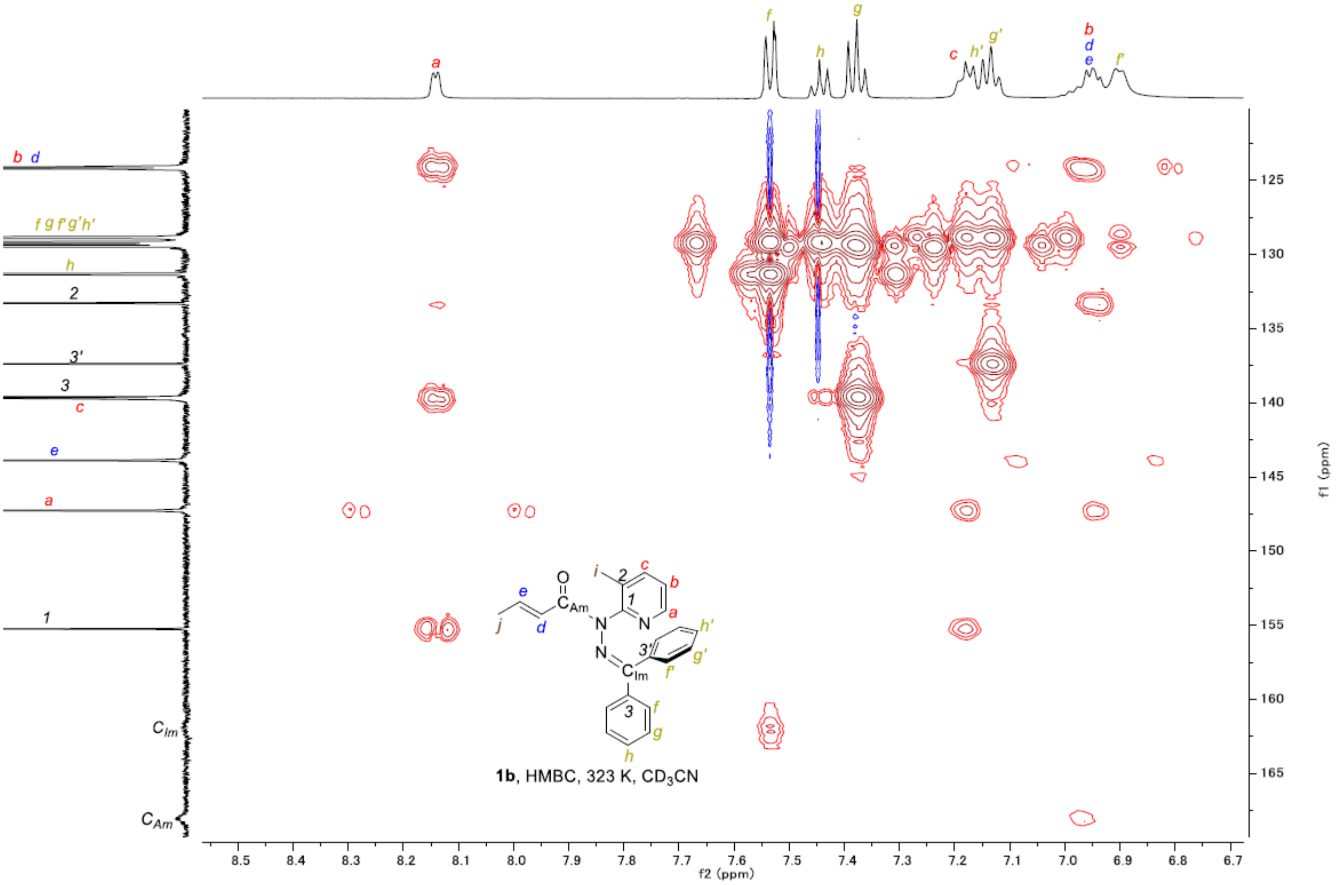
1b, HMBC, 323 K, CD3CN
f2 (ppm)
f1 (ppm)

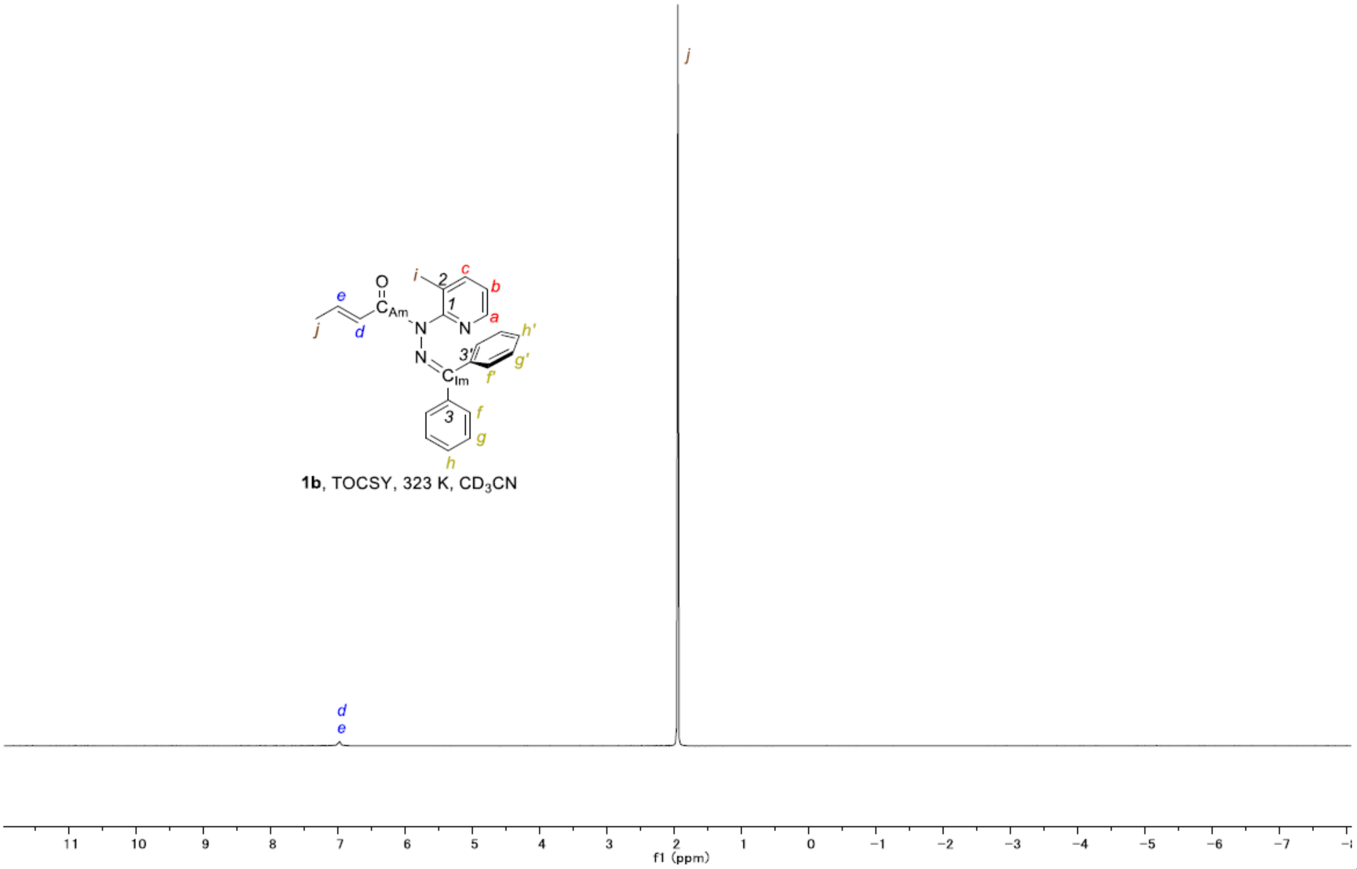

3-

3-2-2. Detailed NMR of (**1b**)$_2$/Pd for peak assignments

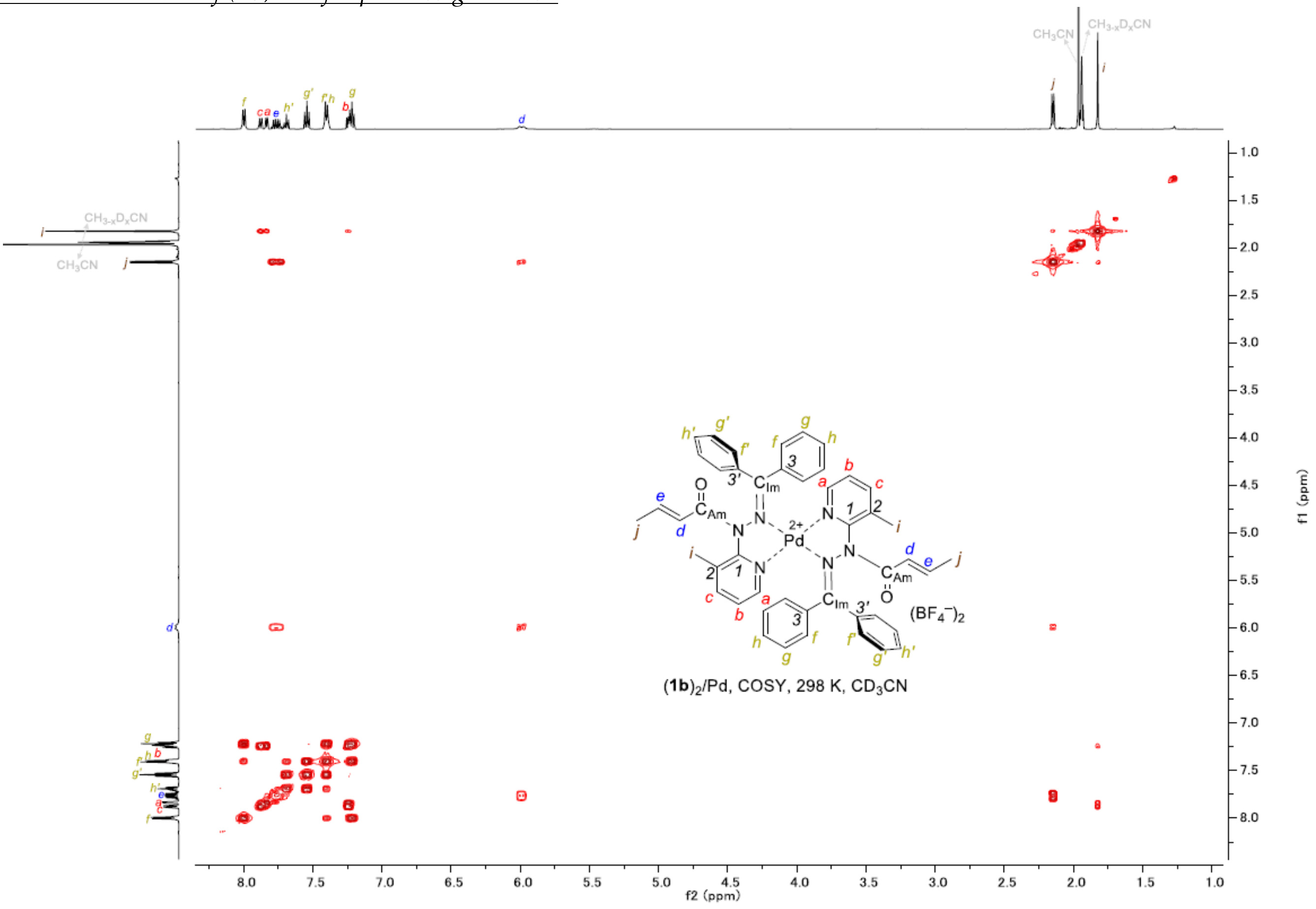

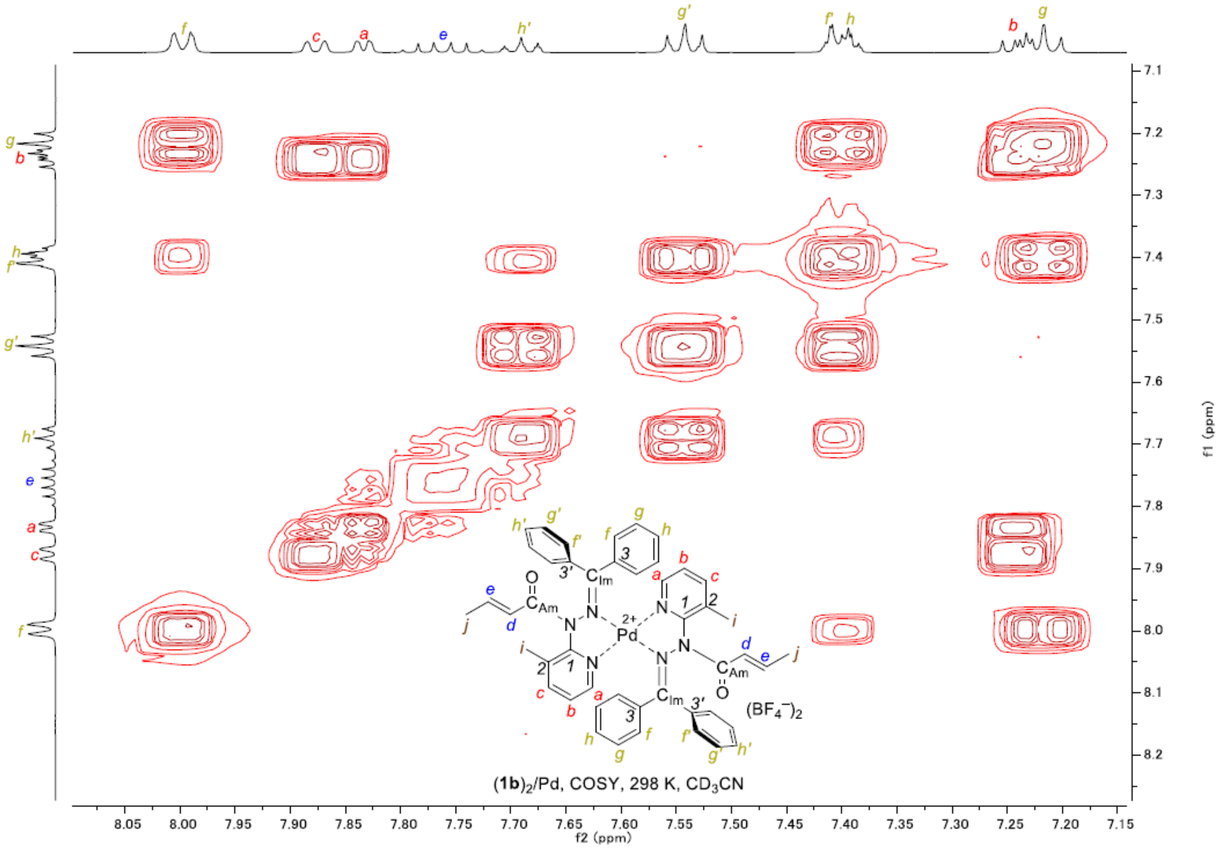
(1b)$_2$/Pd, COSY, 298 K, CD_3CN
f2 (ppm)
f1 (ppm)
$(BF_4^-)_2$

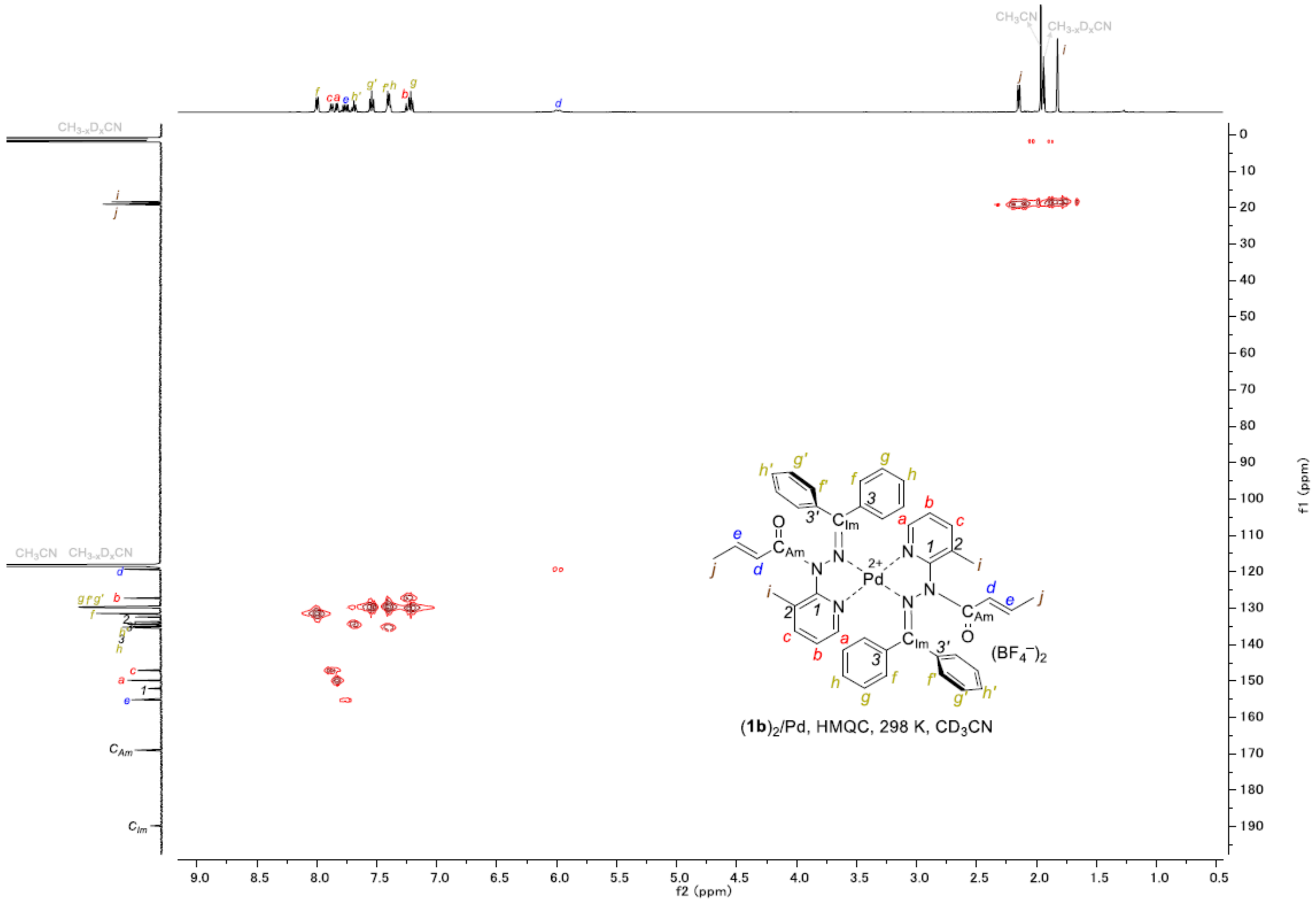
(1b)$_2$/Pd, HMQC, 298 K, CD_3CN
f2 (ppm)
f1 (ppm)
CH_3CN
$CH_{3-x}D_xCN$
$(BF_4^-)_2$

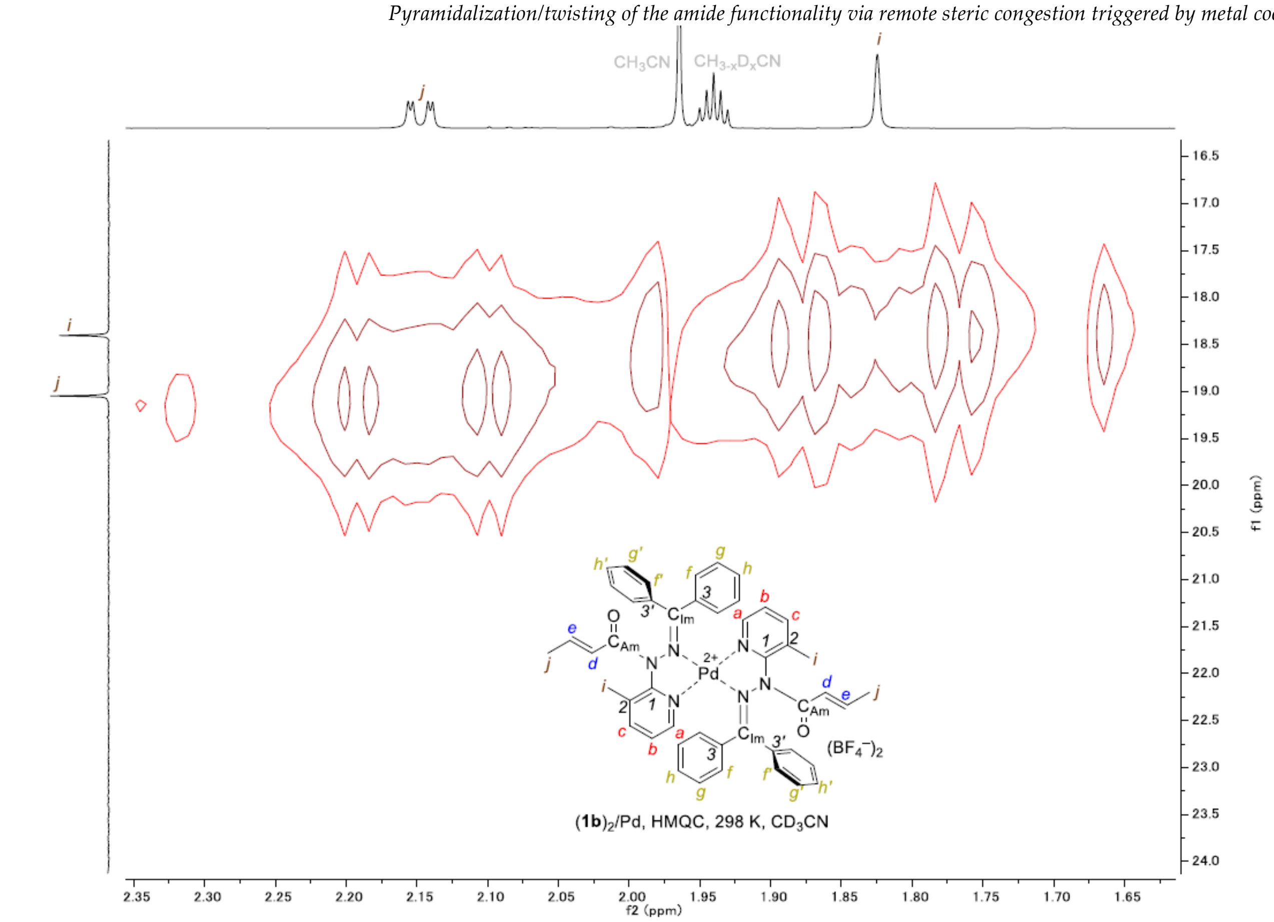
CH3CN
CH3-xDxCN
(1b)2/Pd, HMQC, 298 K, CD3CN
(BF4−)2
f2 (ppm)
f1 (ppm)

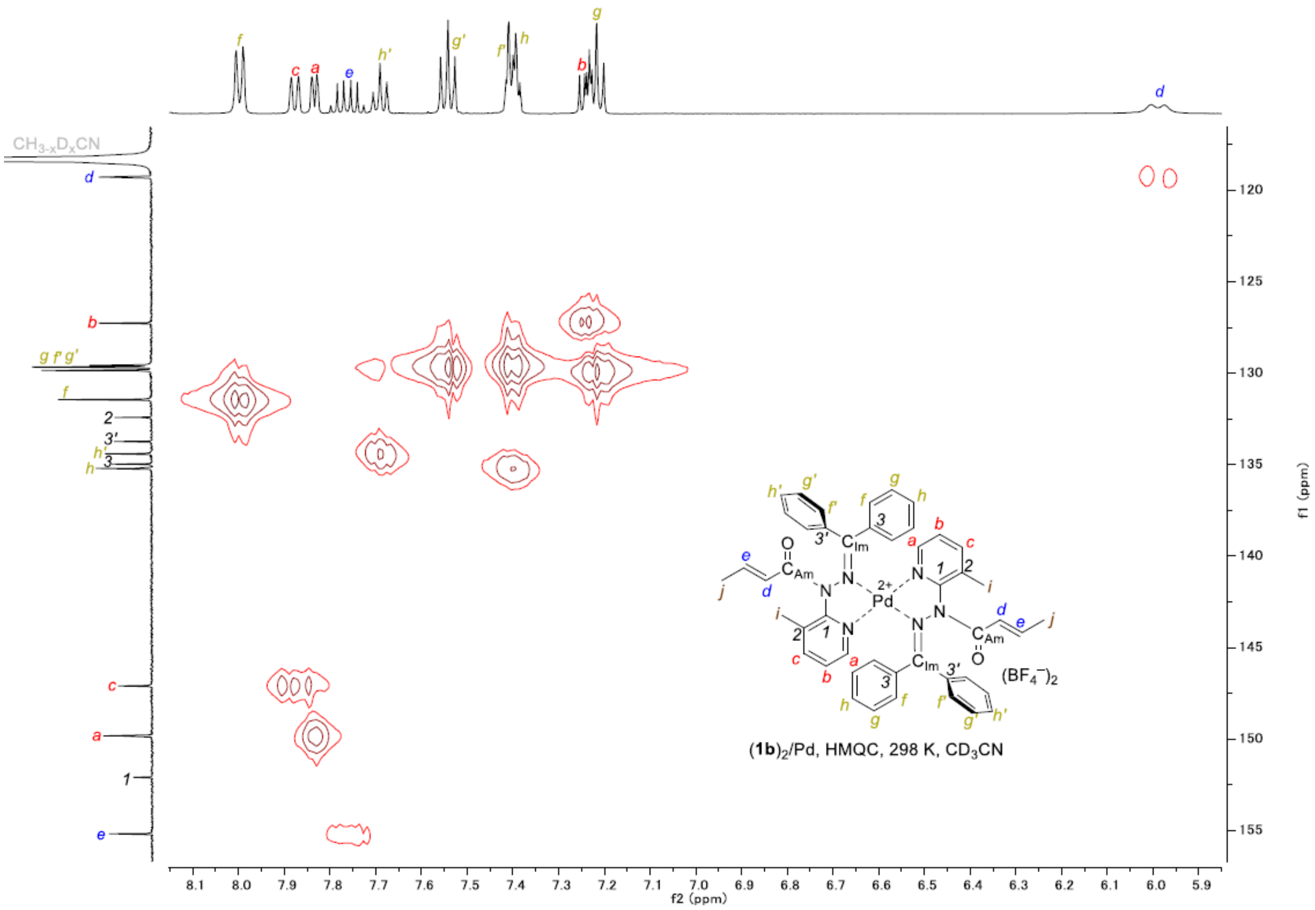
CH3-xDxCN
(1b)2/Pd, HMQC, 298 K, CD3CN
(BF4−)2
f2 (ppm)
f1 (ppm)

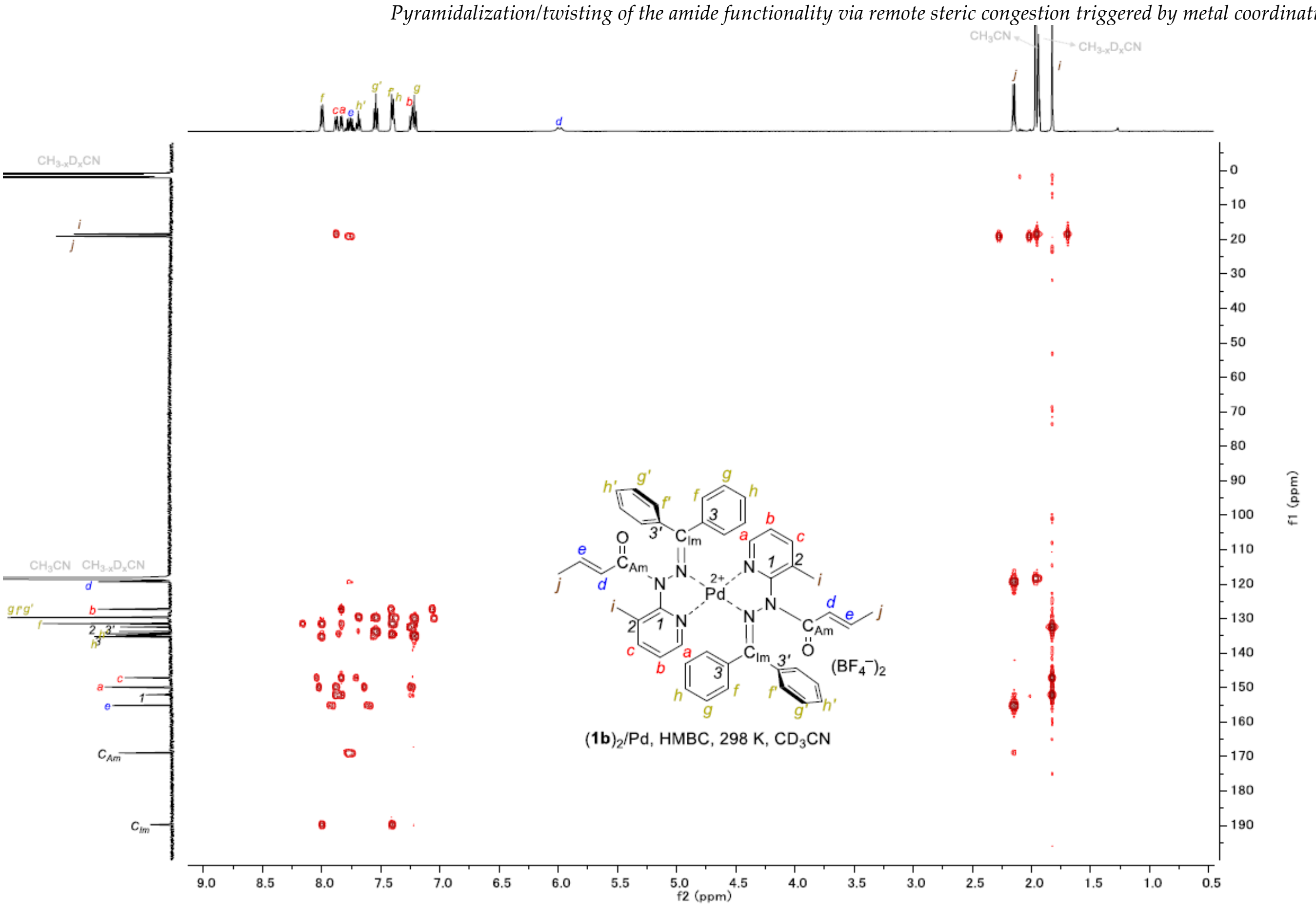
(1b)2/Pd, HMBC, 298 K, CD3CN
(BF4−)2
f2 (ppm)
f1 (ppm)
CH3CN
CH3-xDxCN

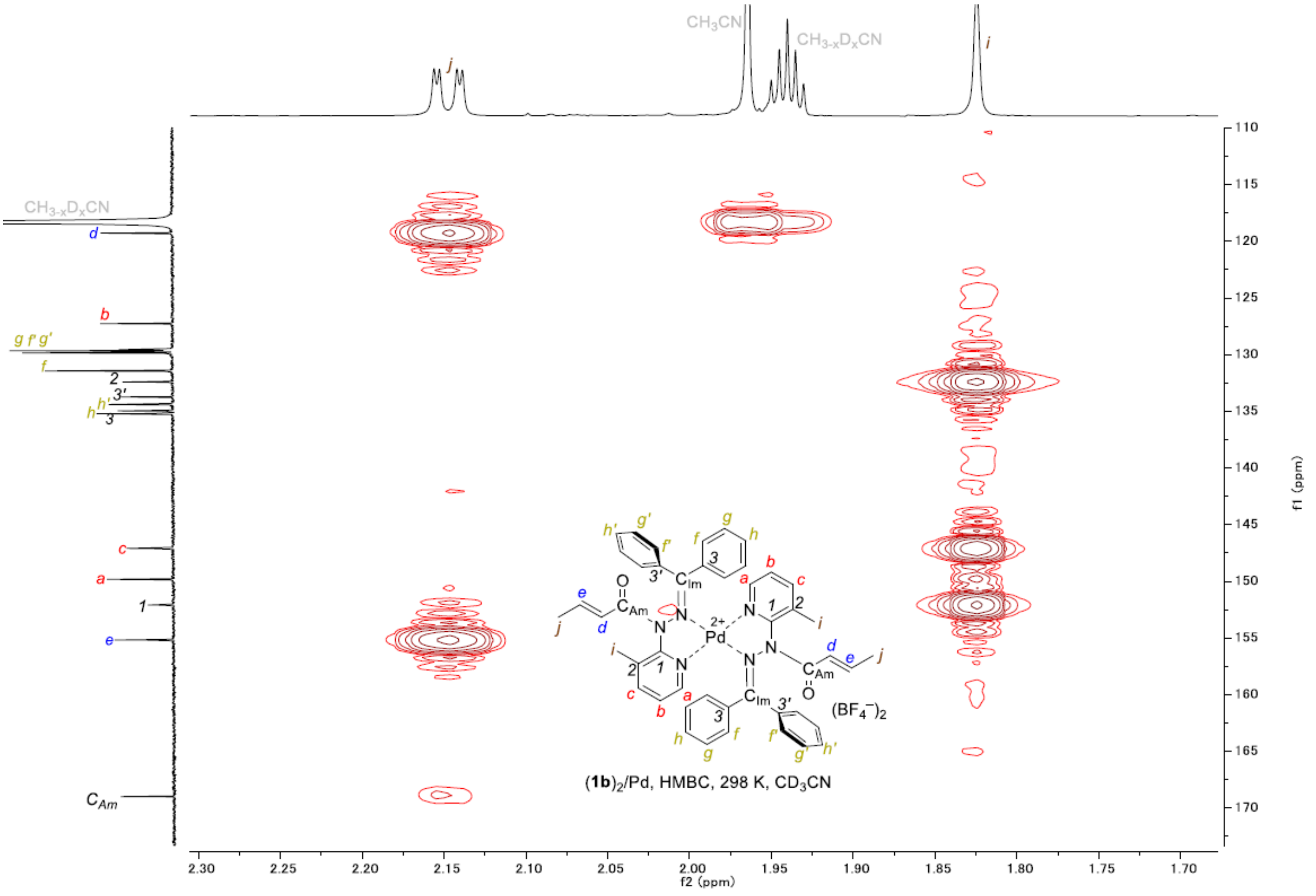
(1b)2/Pd, HMBC, 298 K, CD3CN
(BF4−)2
f2 (ppm)
f1 (ppm)
CH3CN
CH3-xDxCN

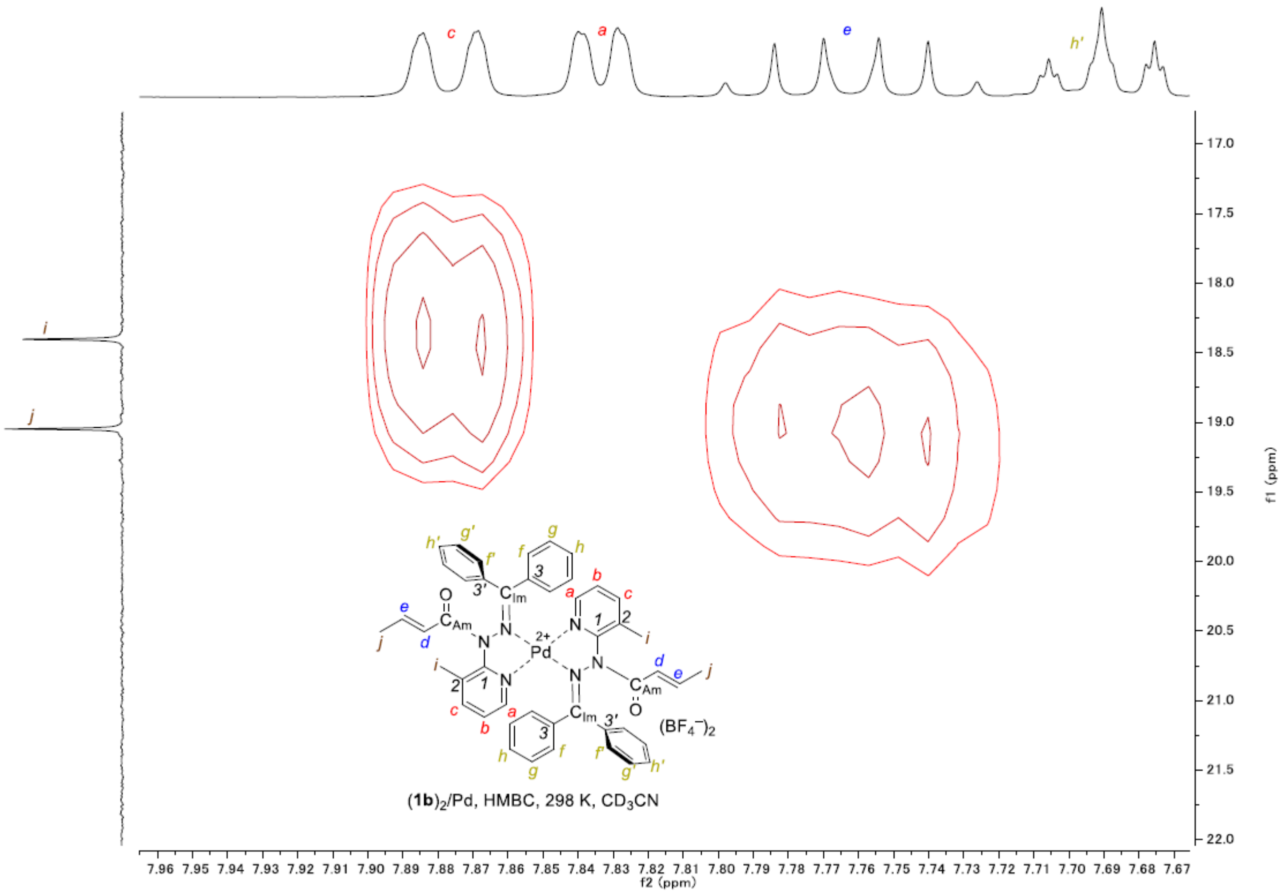
(1b)2/Pd, HMBC, 298 K, CD3CN
(BF4−)2
f2 (ppm)
f1 (ppm)

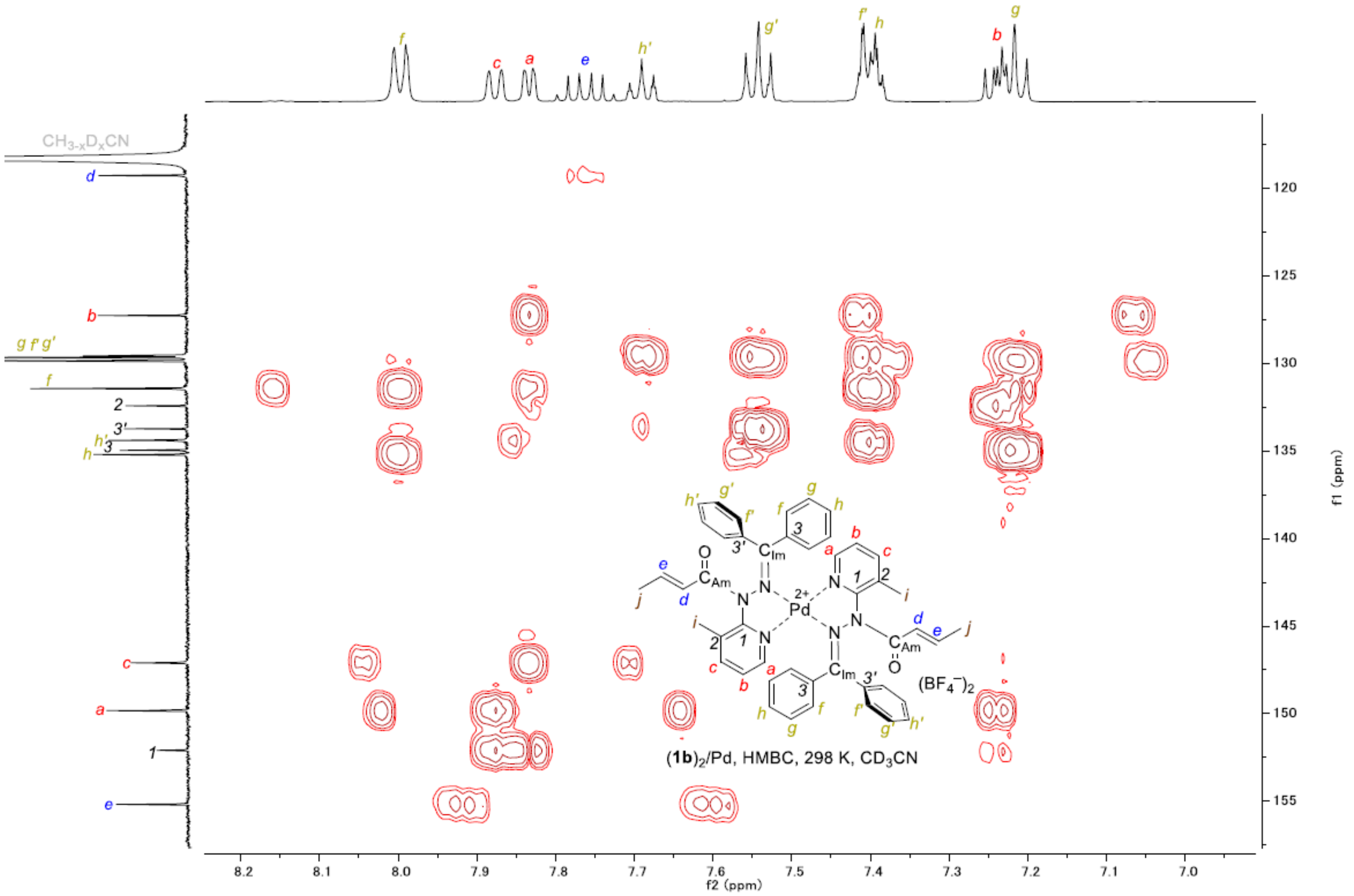
CH3-xDxCN
(1b)2/Pd, HMBC, 298 K, CD3CN
(BF4−)2
f2 (ppm)
f1 (ppm)

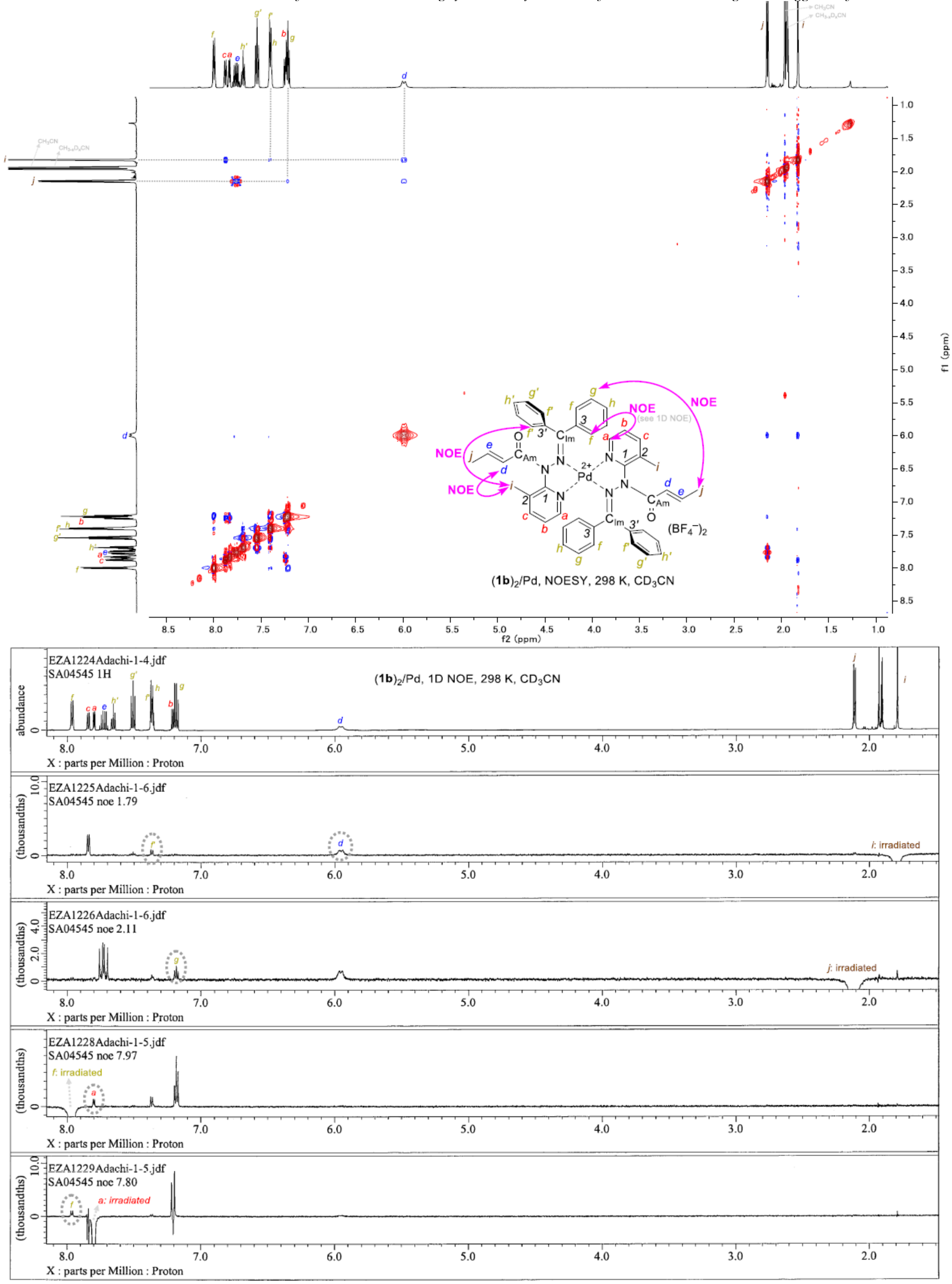
(1b)2/Pd, NOESY, 298 K, CD3CN
NOE
(see 1D NOE)
(BF4−)2
f2 (ppm)
f1 (ppm)
EZA1224Adachi-1-4.jdf
SA04545 1H
(1b)2/Pd, 1D NOE, 298 K, CD3CN
abundance
X : parts per Million : Proton
EZA1225Adachi-1-6.jdf
SA04545 noe 1.79
i: irradiated
(thousandths)
EZA1226Adachi-1-6.jdf
SA04545 noe 2.11
j: irradiated
EZA1228Adachi-1-5.jdf
SA04545 noe 7.97
f: irradiated
EZA1229Adachi-1-5.jdf
SA04545 noe 7.80
a: irradiated

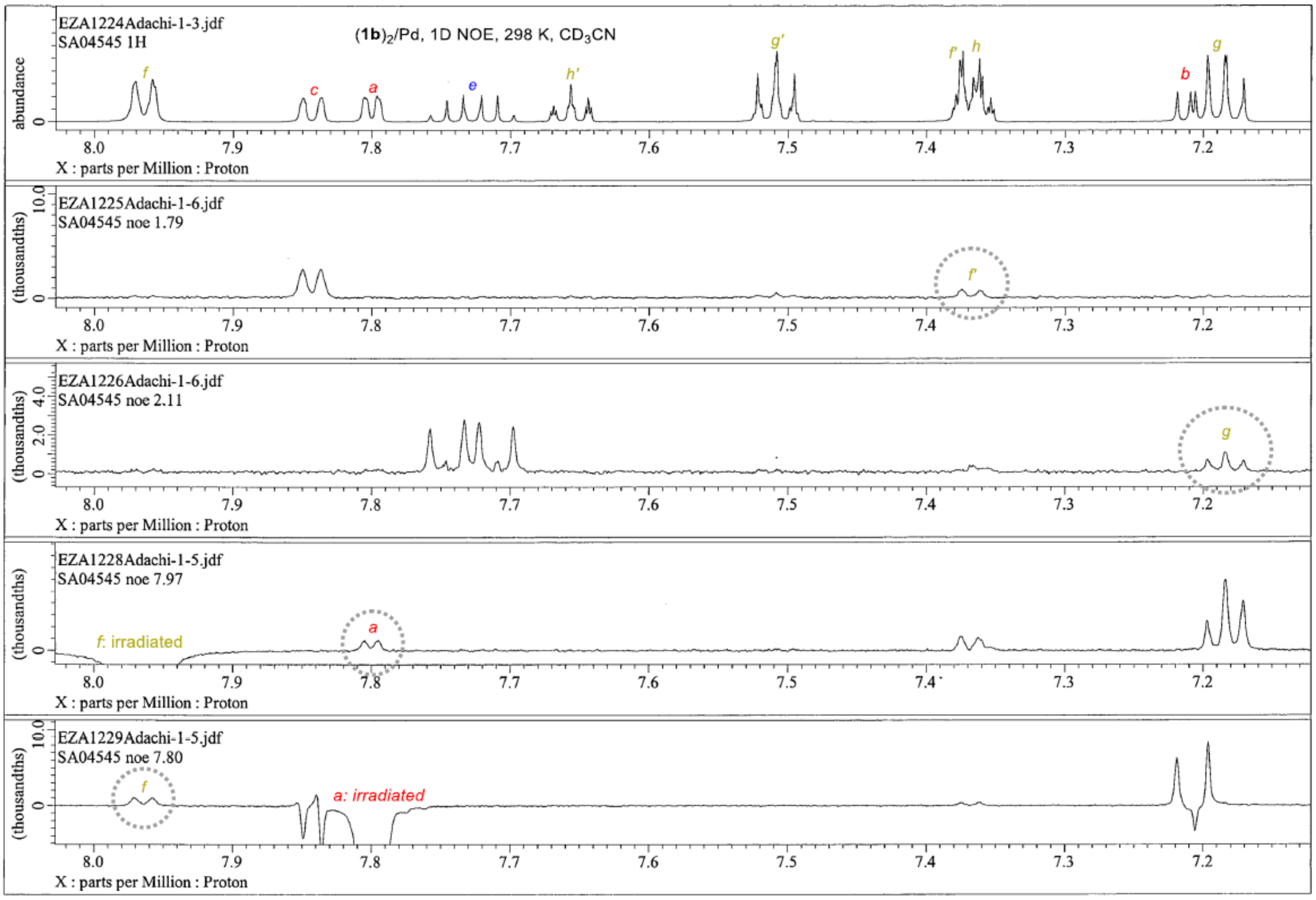
EZA1224Adachi-1-3.jdf
SA04545 1H
(1b)2/Pd, 1D NOE, 298 K, CD3CN
EZA1225Adachi-1-6.jdf
SA04545 noe 1.79
EZA1226Adachi-1-6.jdf
SA04545 noe 2.11
EZA1228Adachi-1-5.jdf
SA04545 noe 7.97
f: irradiated
EZA1229Adachi-1-5.jdf
SA04545 noe 7.80
a: irradiated
X : parts per Million : Proton

*3-2-3. Stacked ^{1}H and ^{13}C NMR of **1b** and (**1b**)$_2$/Pd*

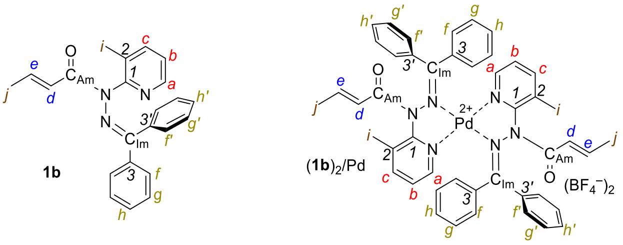

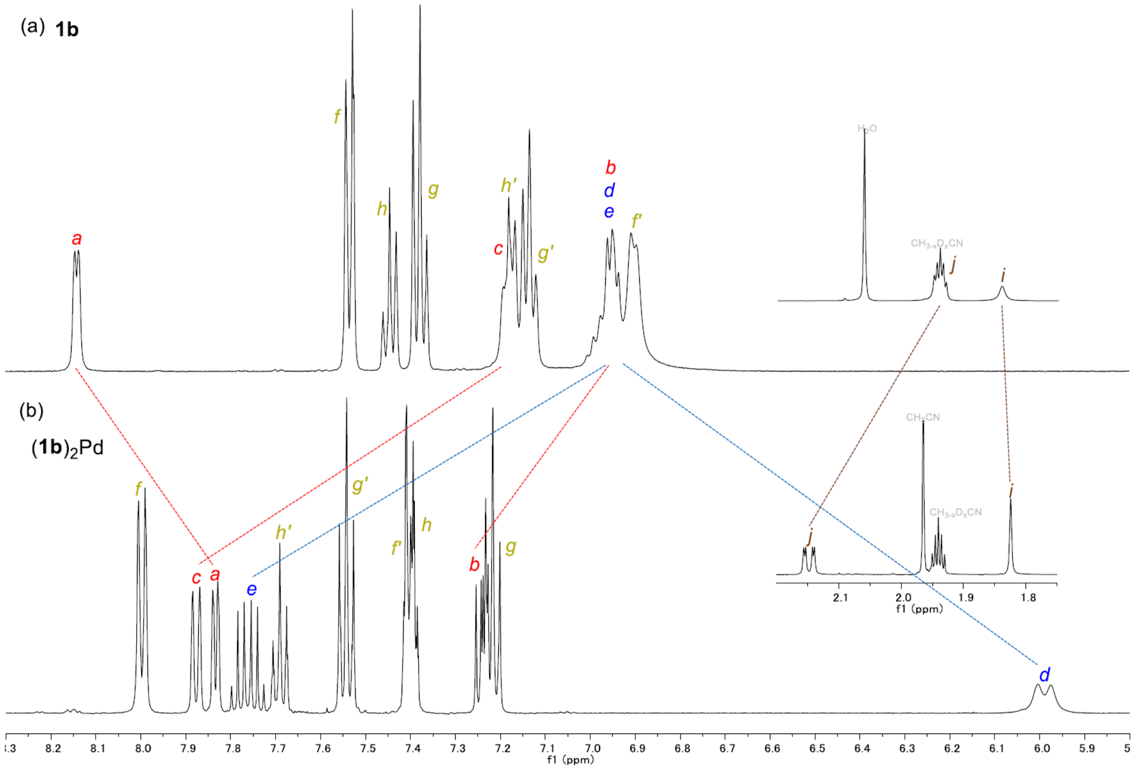

Figure S3. (a) ^{1}H NMR of **1b** in CD_3CN. (b) ^{1}H NMR of **1b**/[Pd(CH_3CN)$_4$](BF_4)$_2$ = 2/1 in CD_3CN. *f*▪*g*▪*h* and *f′*▪*g′*▪*h′* are not distinguished in (a).

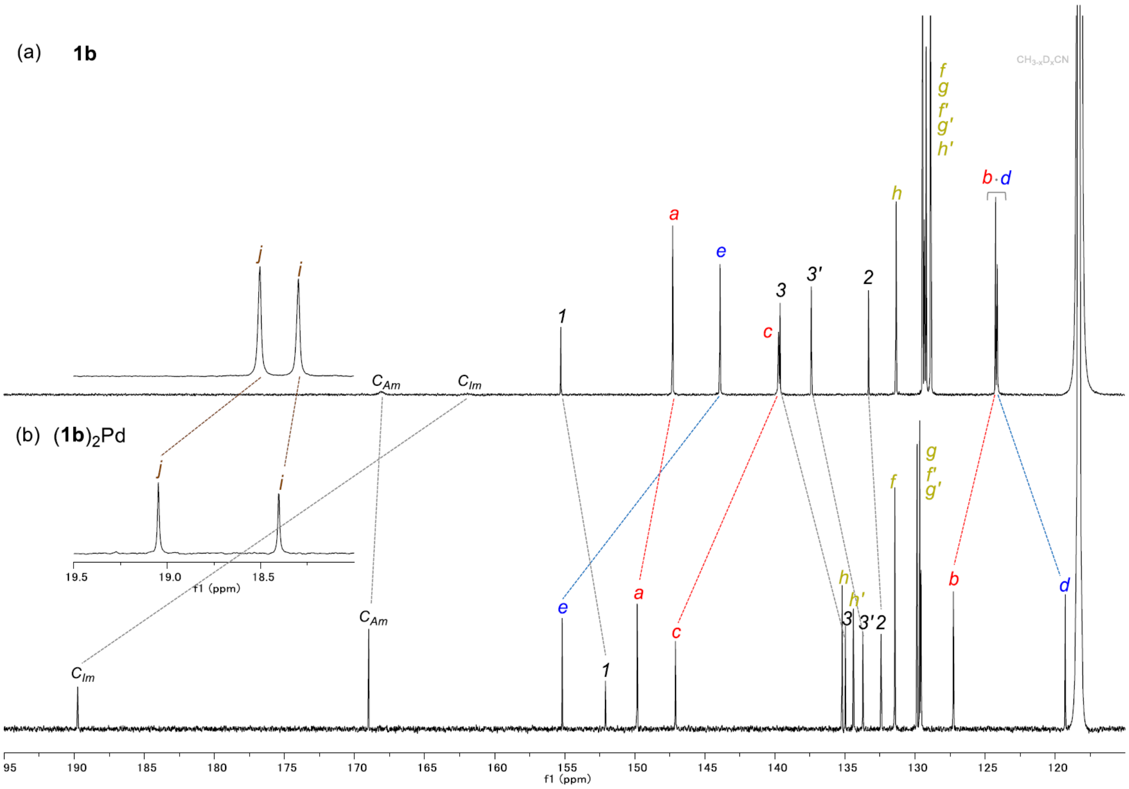

Figure S4. (a) ^{13}C NMR of **1b** in CD_3CN. (b) ^{13}C NMR of **1b**/[Pd(CH_3CN)$_4$](BF_4)$_2$ = 2/1 in CD_3CN. *f*▪*g*▪*h*▪3 and *f′*▪*g′*▪*h′*▪3′ are not distinguished in (a).

3-1. **1c**–Pd Complex

3-3-1. Detailed NMR of **1c** for peak assignments

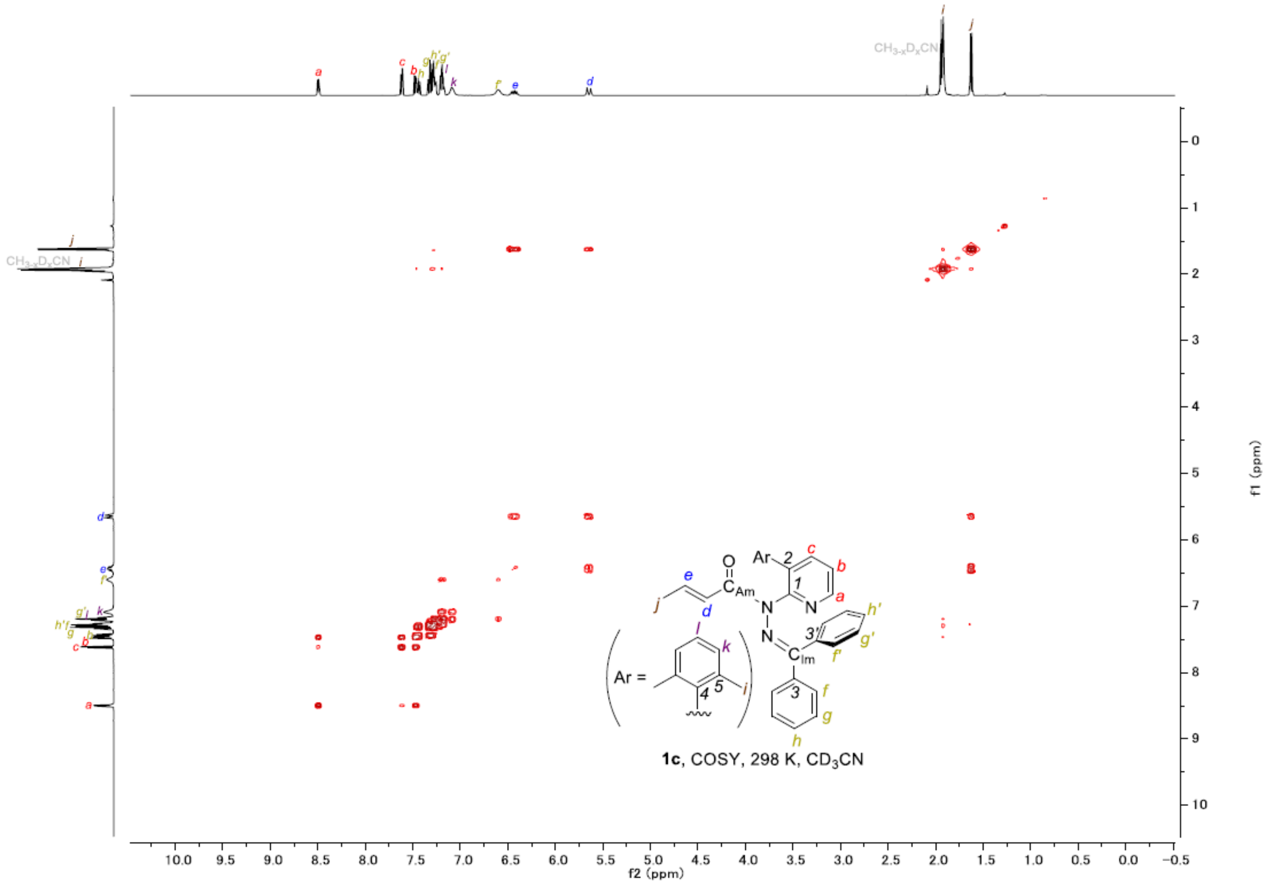

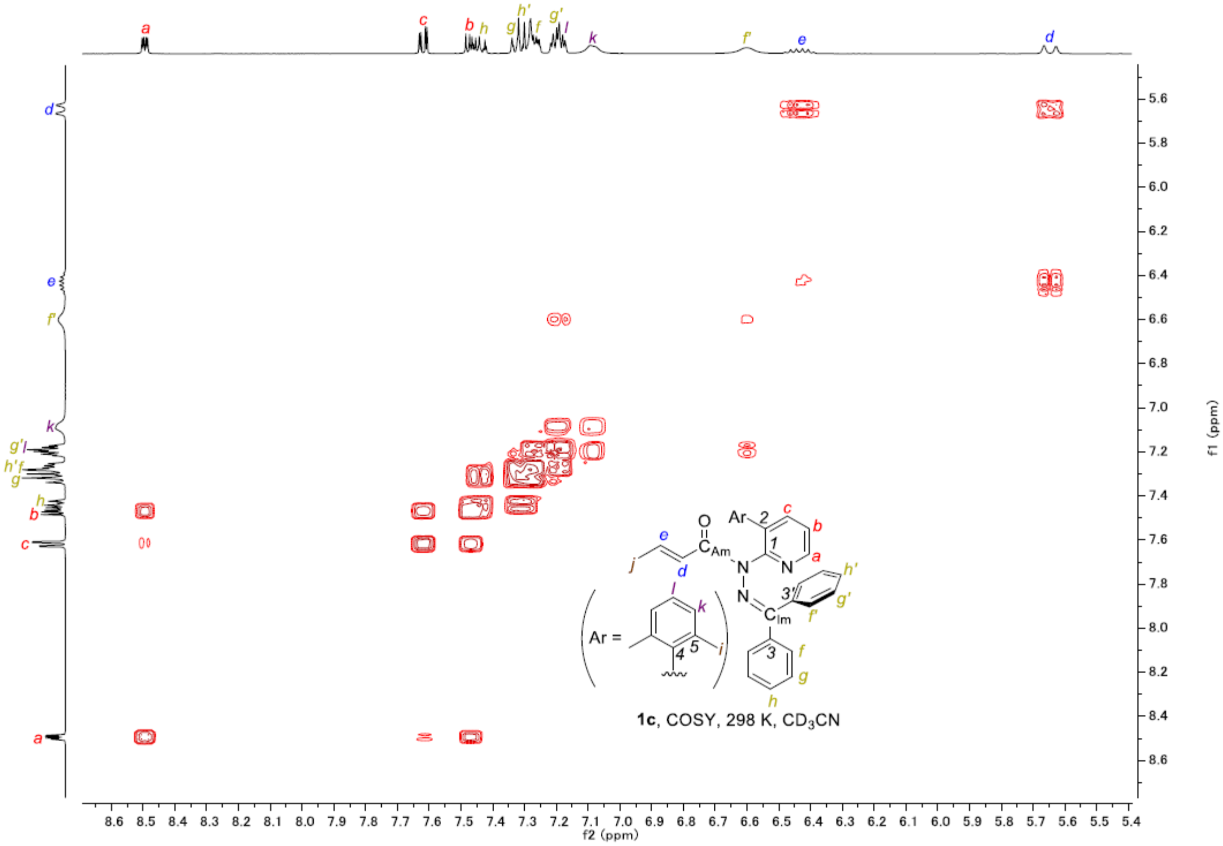

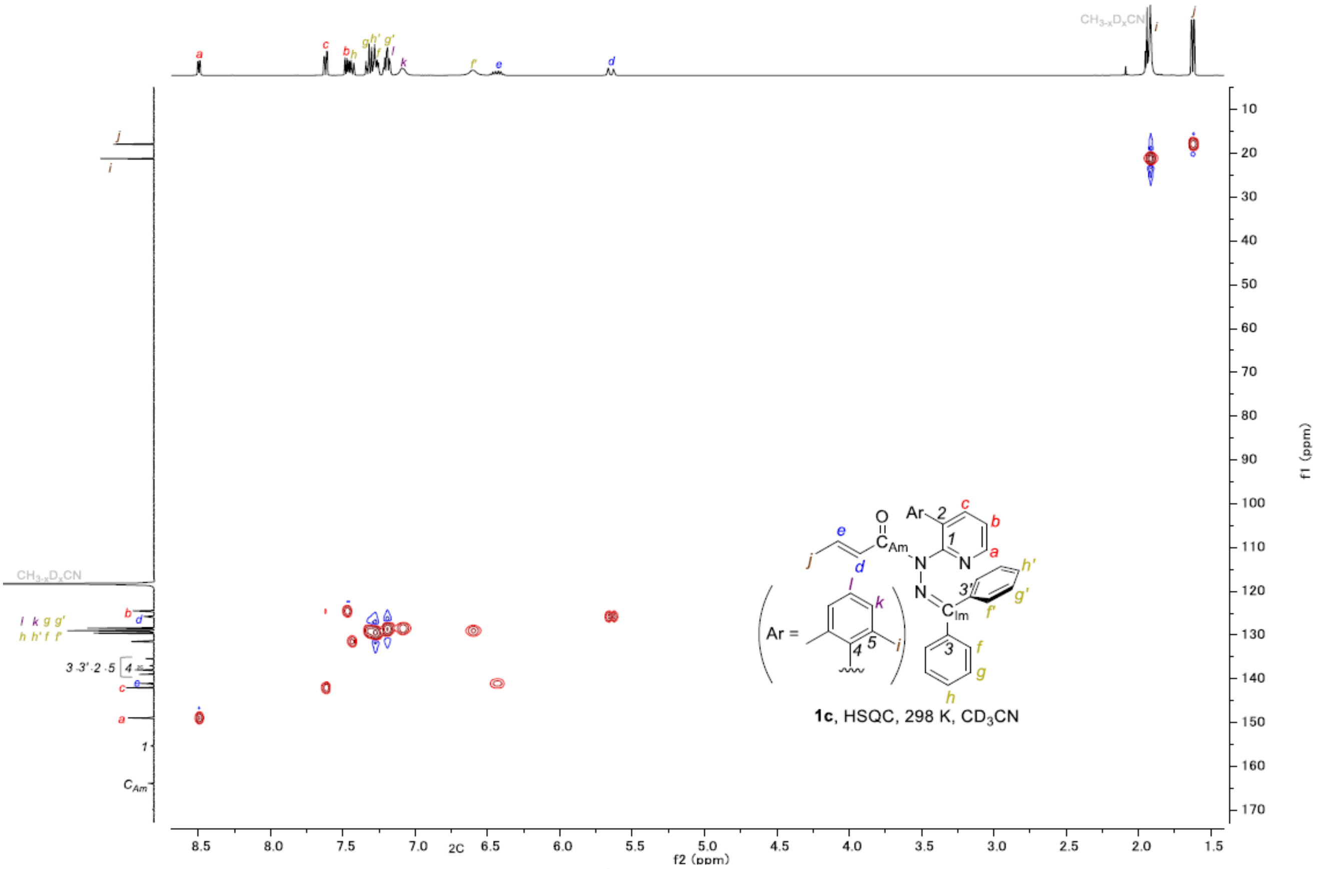
1c, HSQC, 298 K, CD3CN
f2 (ppm)
f1 (ppm)
CH3-xDxCN

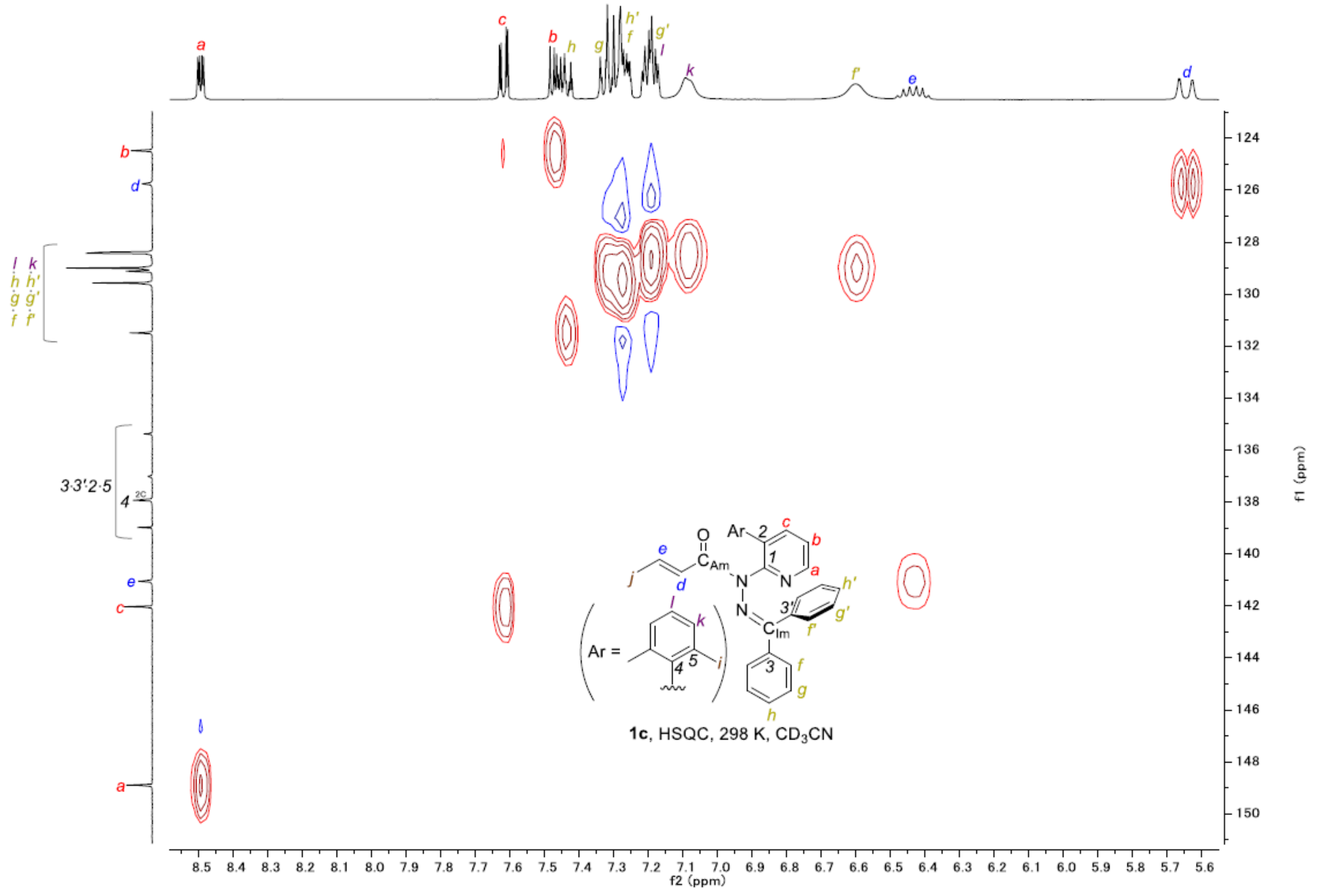
1c, HSQC, 298 K, CD3CN
f2 (ppm)
f1 (ppm)

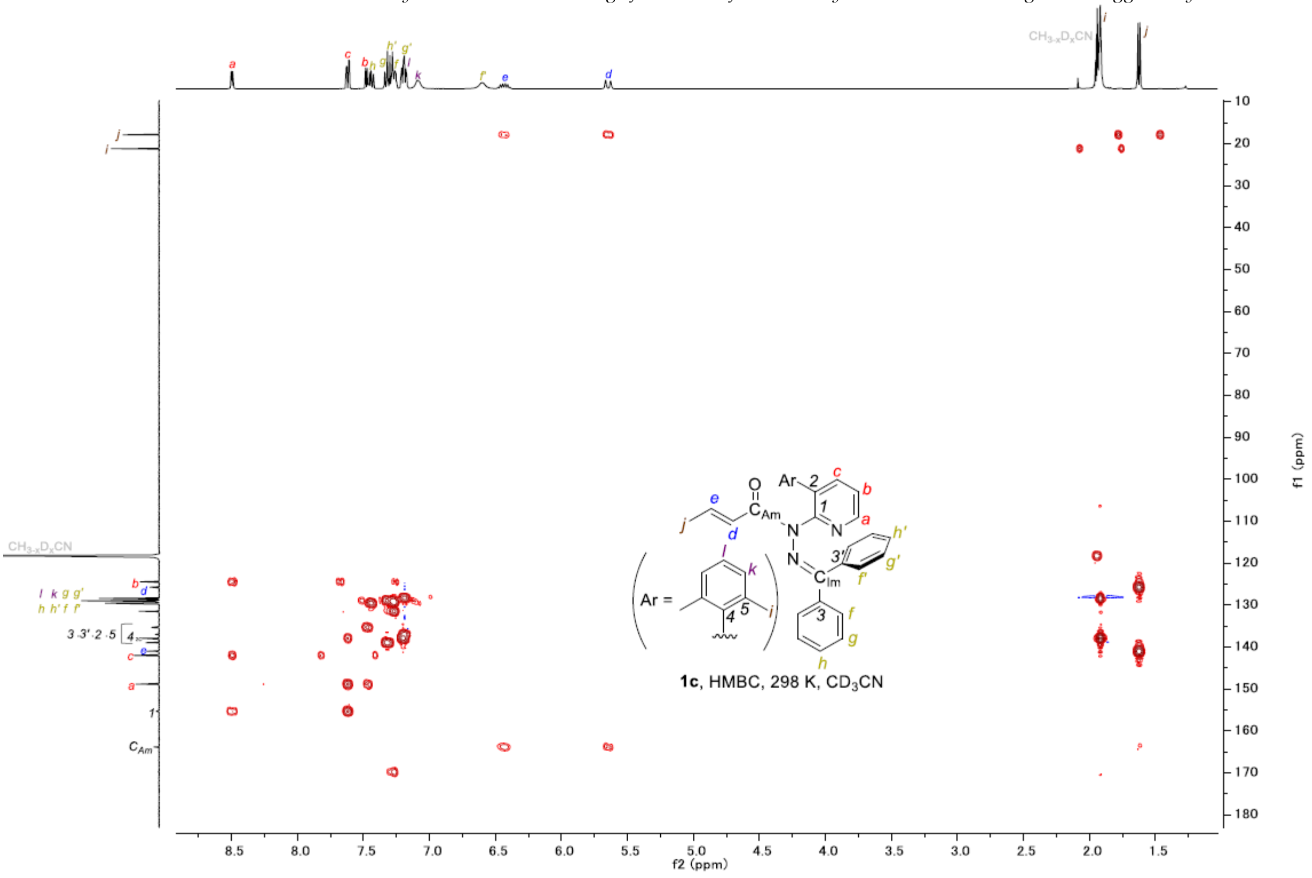
1c, HMBC, 298 K, CD3CN
f2 (ppm)
f1 (ppm)
CH3-xDxCN

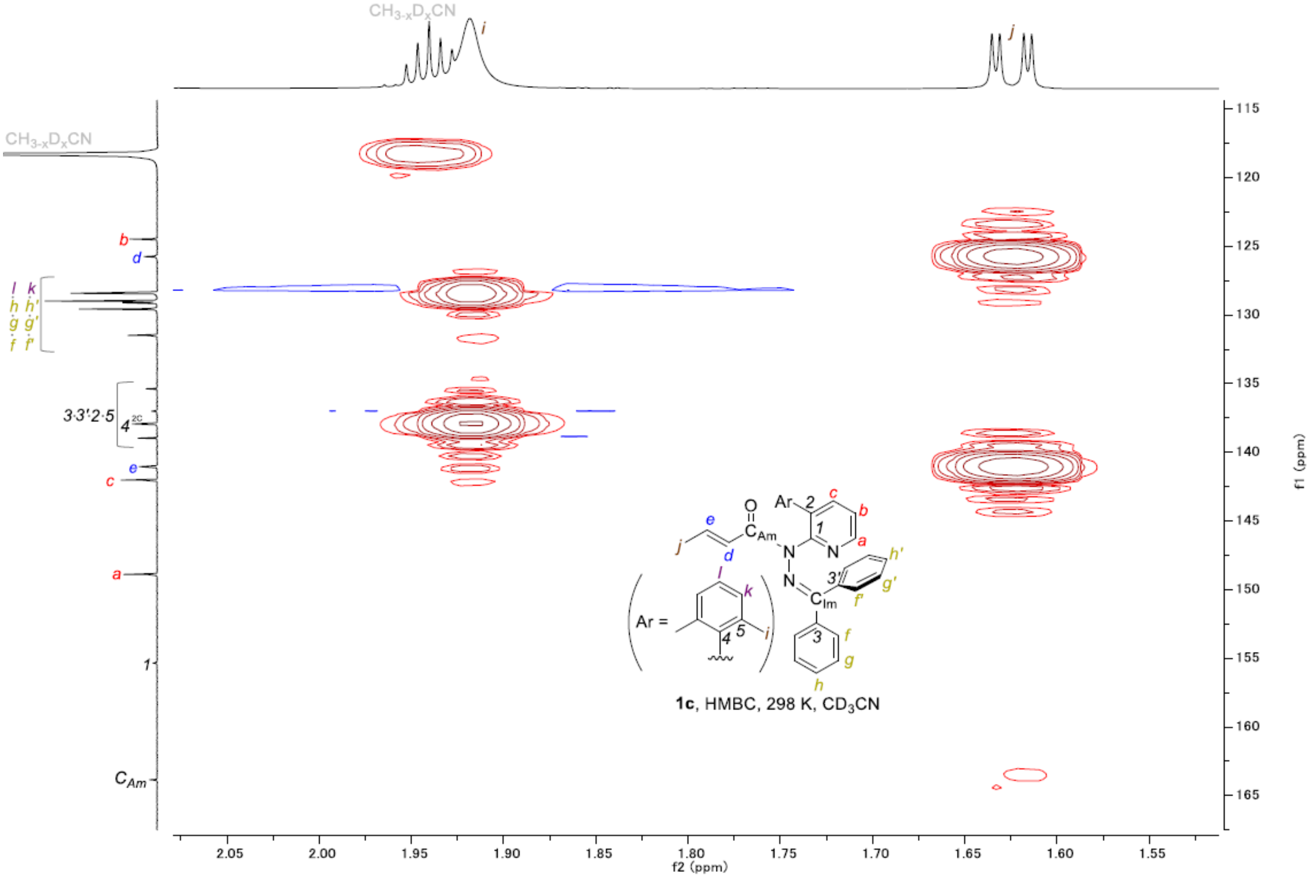
1c, HMBC, 298 K, CD3CN
f2 (ppm)
f1 (ppm)
CH3-xDxCN

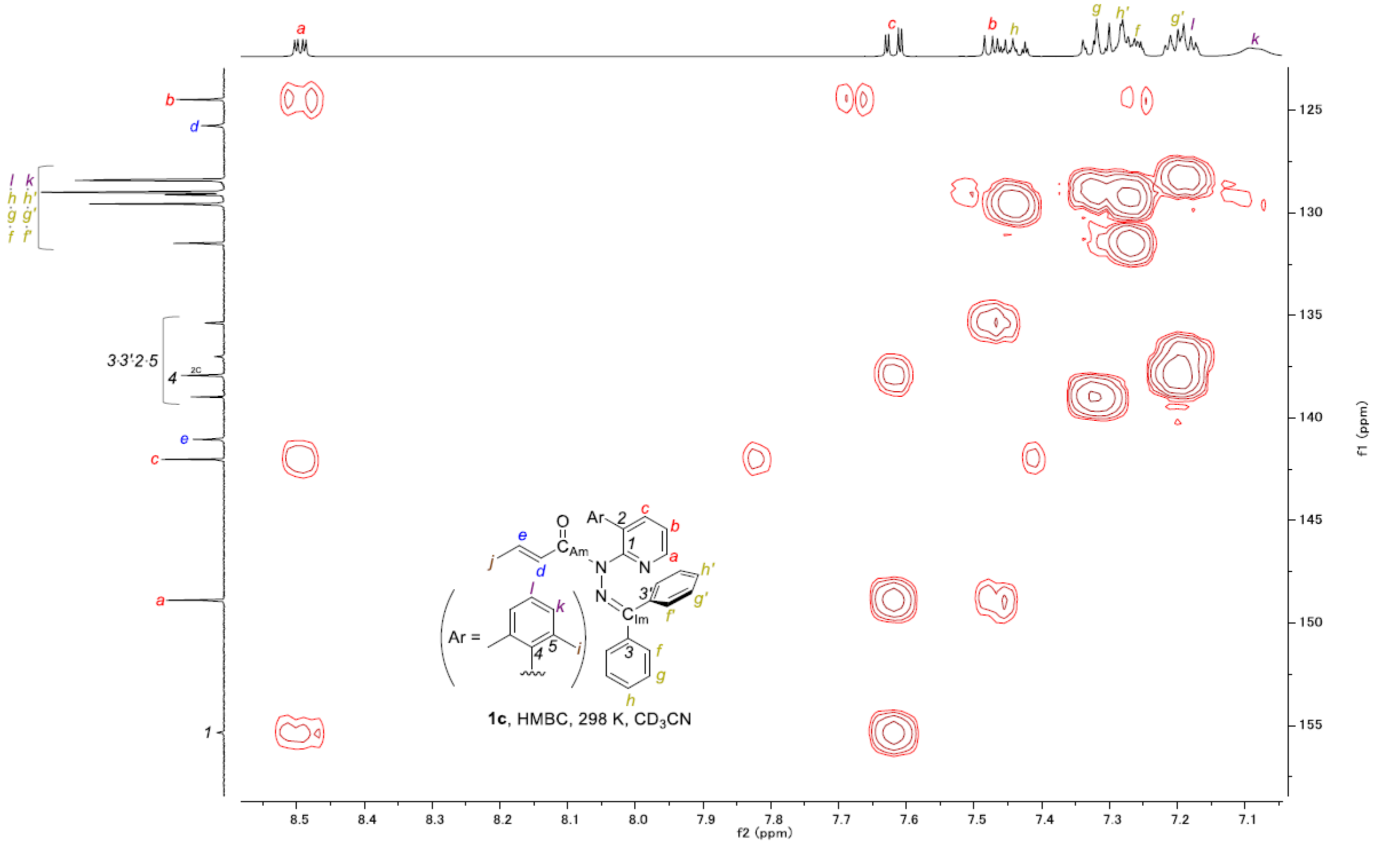

3-3-2. Detailed NMR of (**1c**)2/Pd for peak assignments

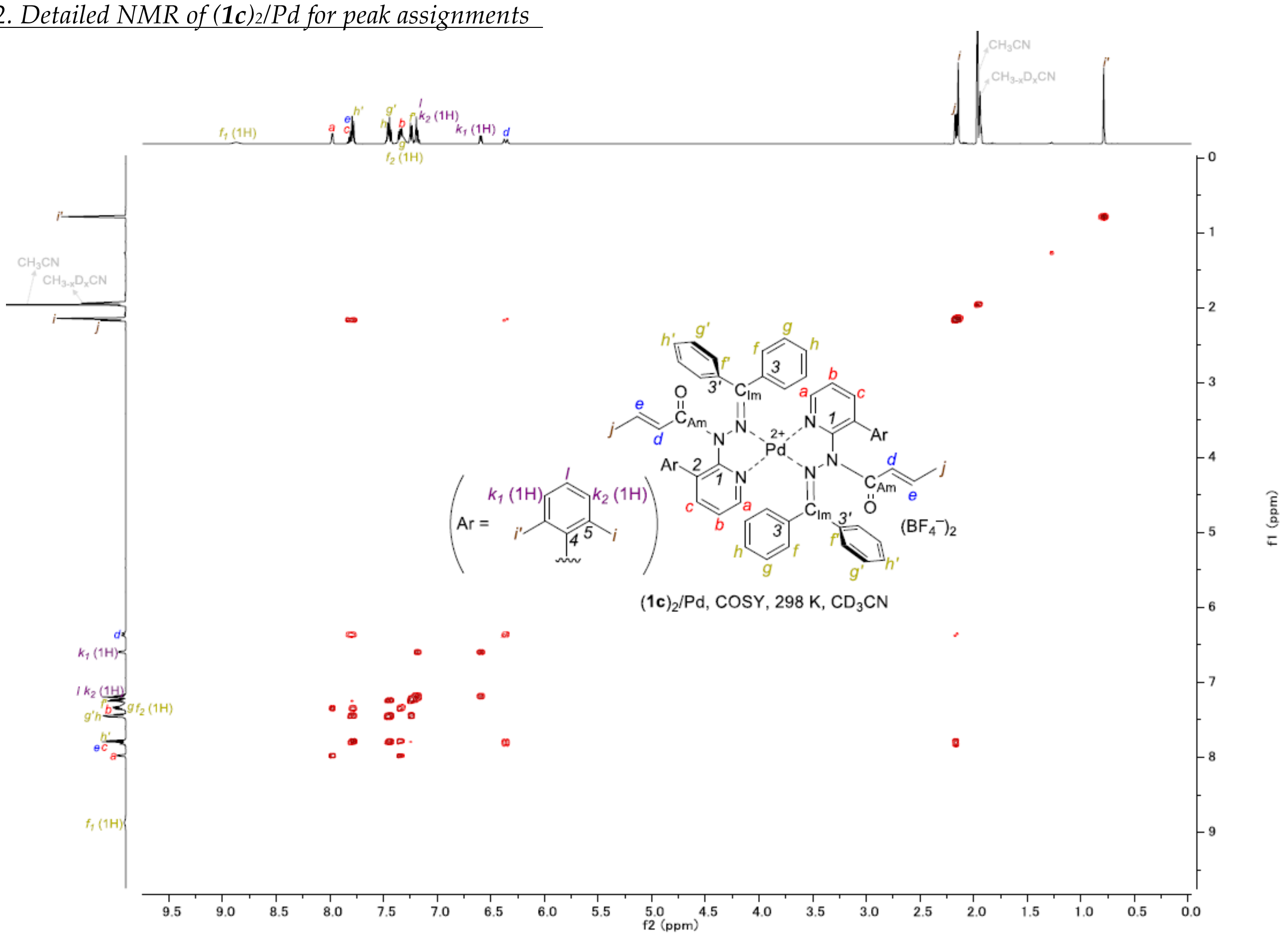

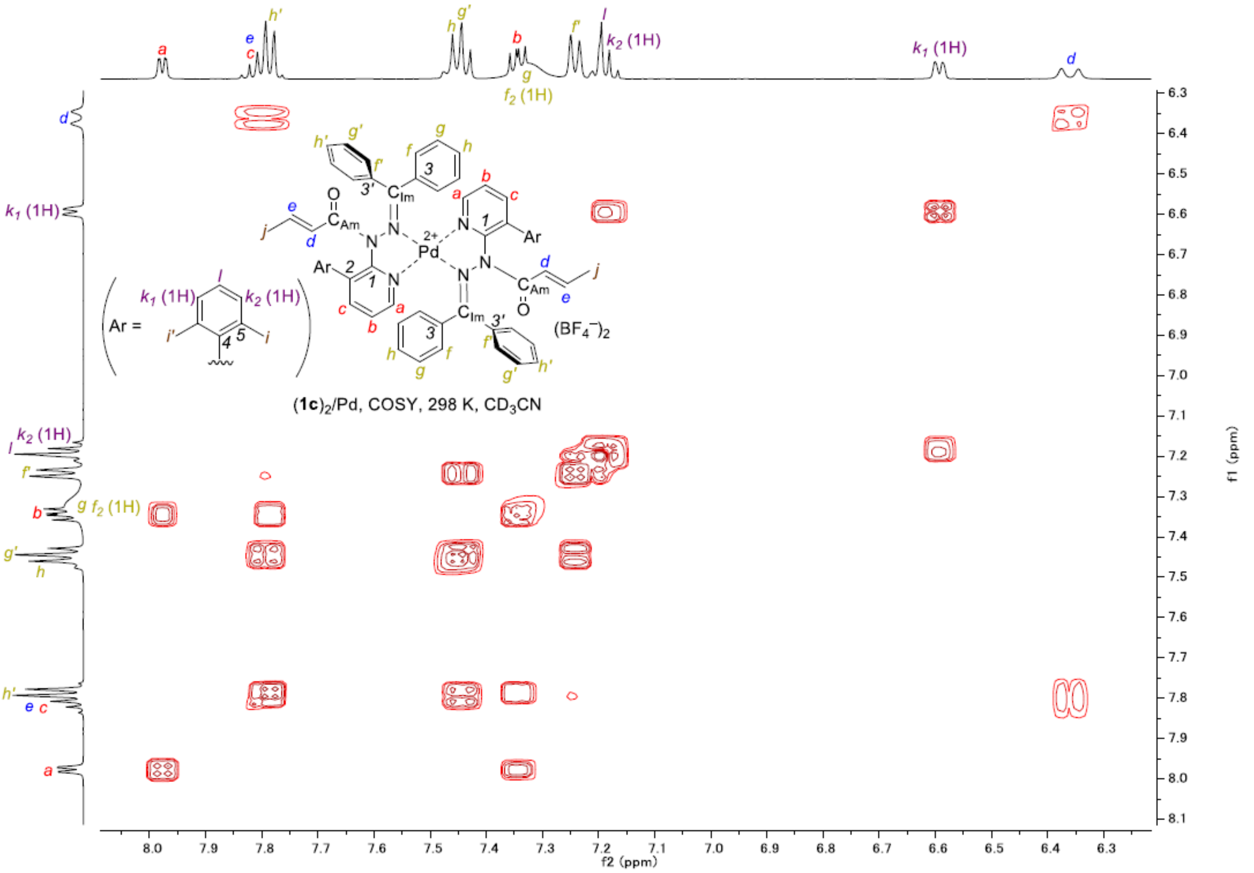
(1c)2/Pd, COSY, 298 K, CD3CN
f2 (ppm)
f1 (ppm)

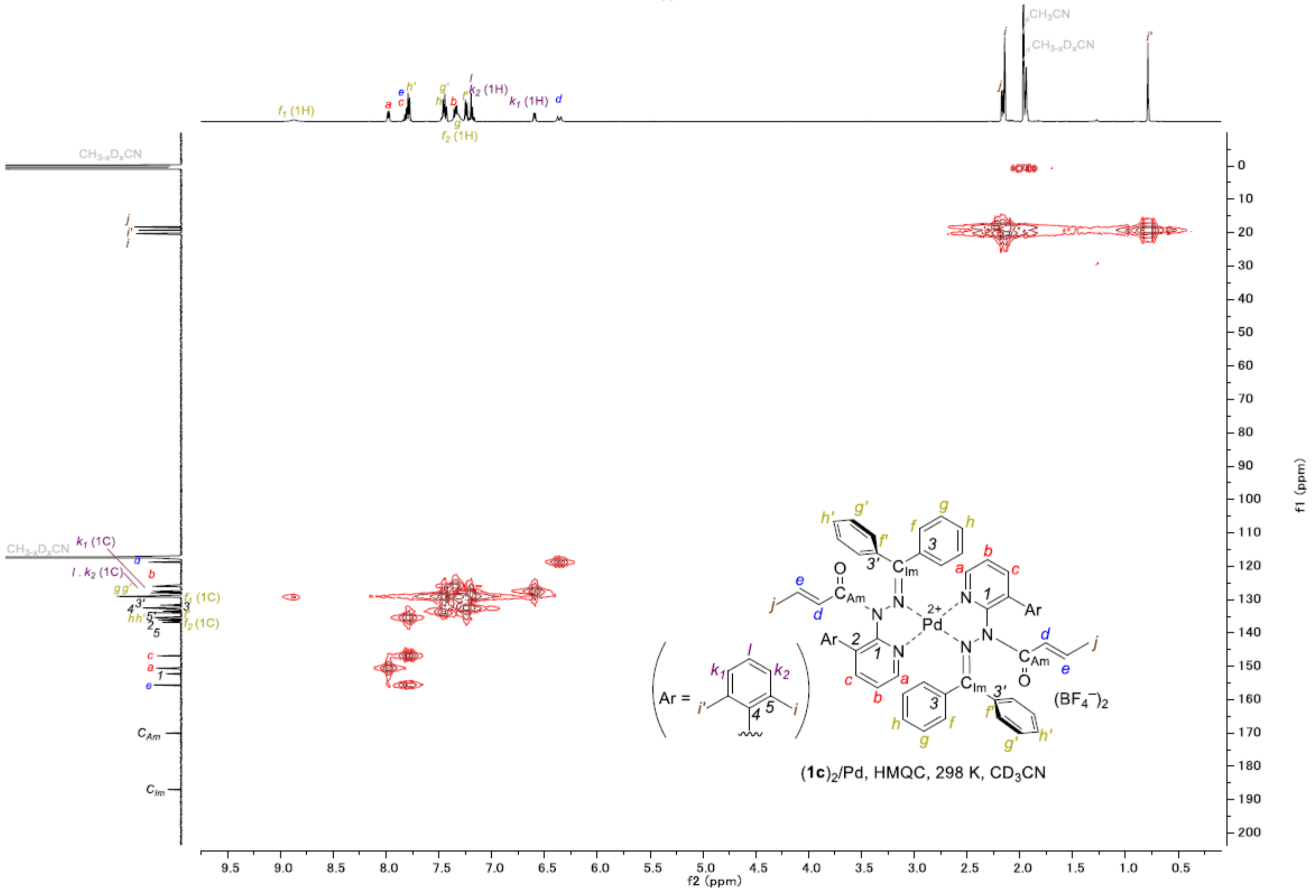
(1c)2/Pd, HMQC, 298 K, CD3CN
f2 (ppm)
f1 (ppm)

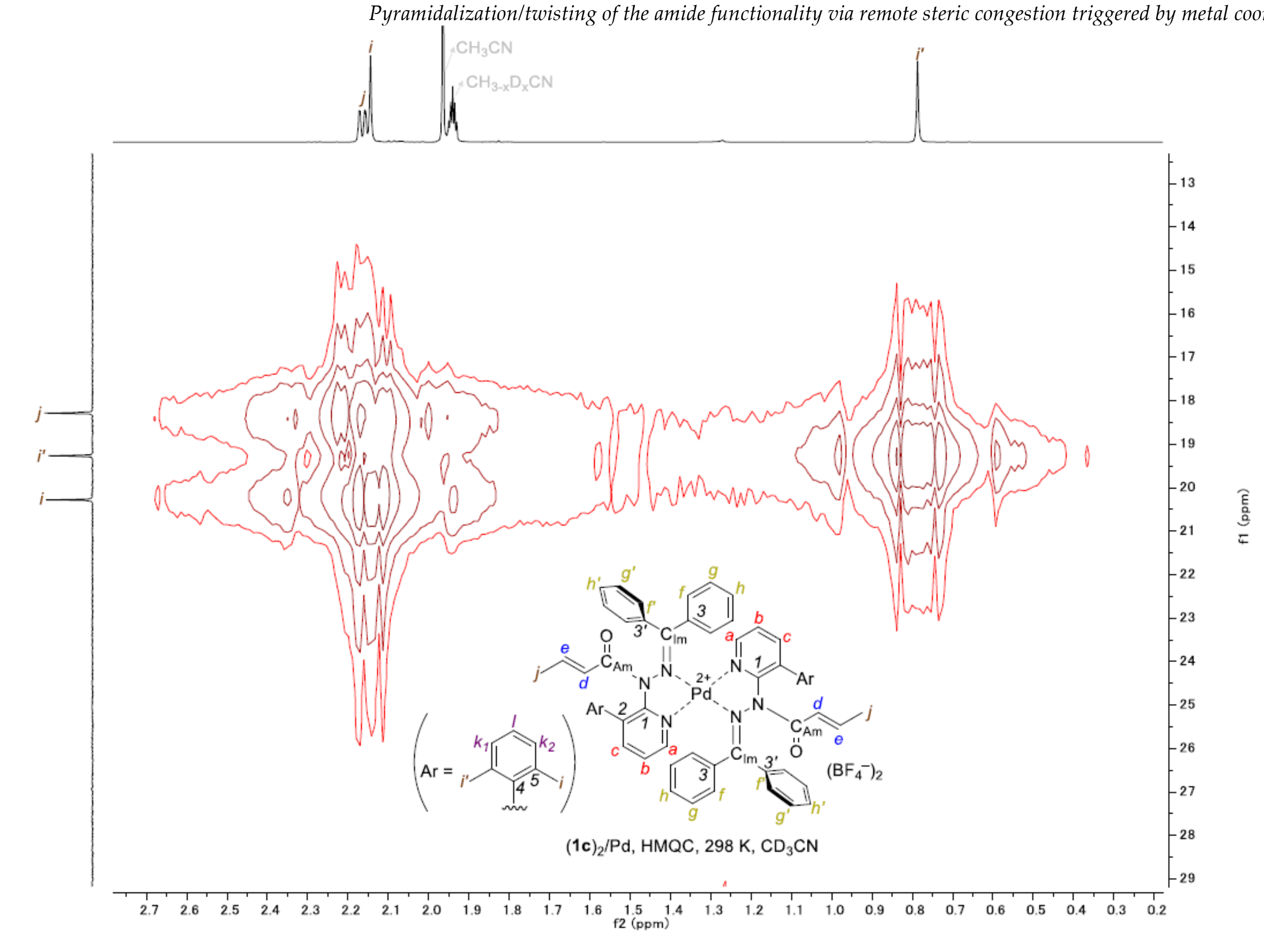
CH3CN
CH3-xDxCN
(1c)2/Pd, HMQC, 298 K, CD3CN
(BF4−)2
Ar =
f2 (ppm)
f1 (ppm)

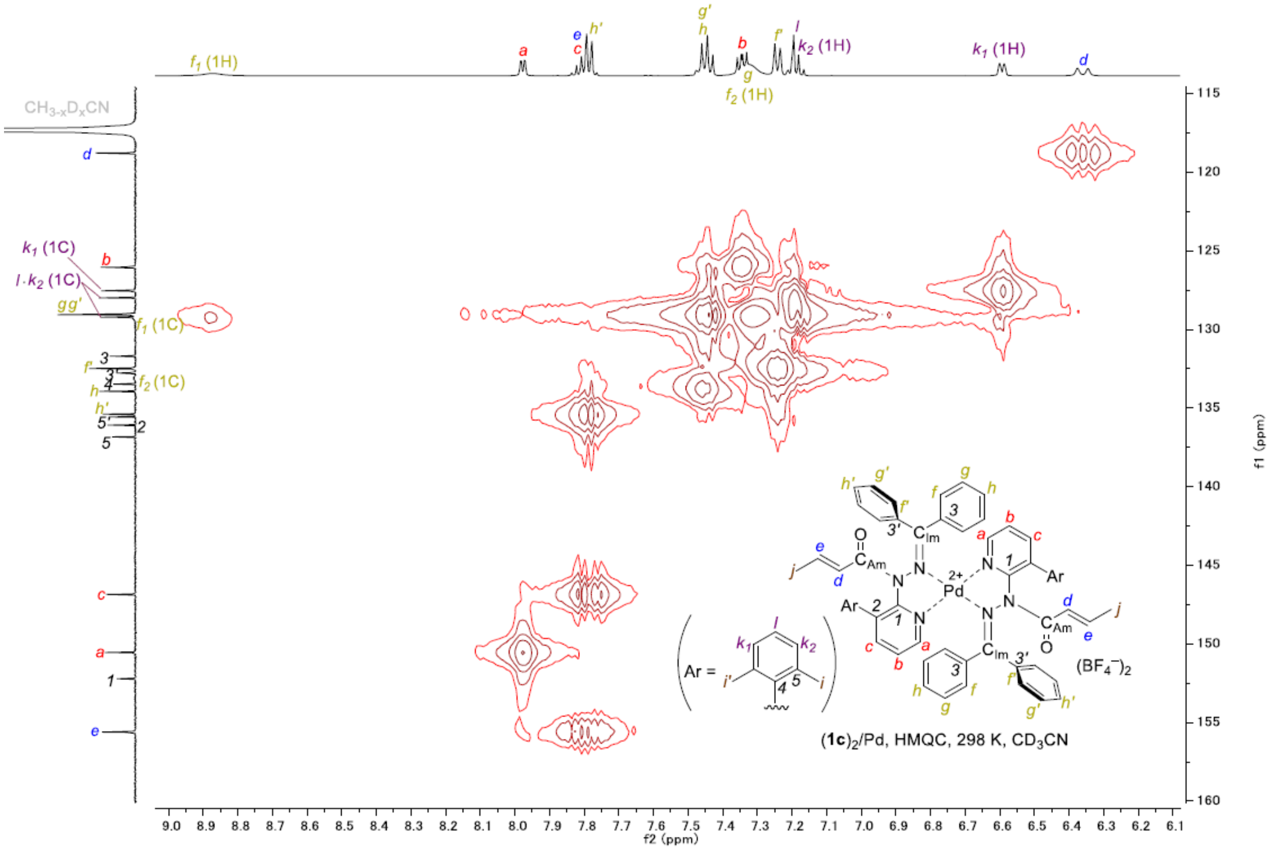
f1 (1H)
f2 (1H)
k1 (1H)
k2 (1H)
CH3-xDxCN
k1 (1C)
l-k2 (1C)
f1 (1C)
f2 (1C)
(1c)2/Pd, HMQC, 298 K, CD3CN
(BF4−)2
Ar =
f2 (ppm)
f1 (ppm)

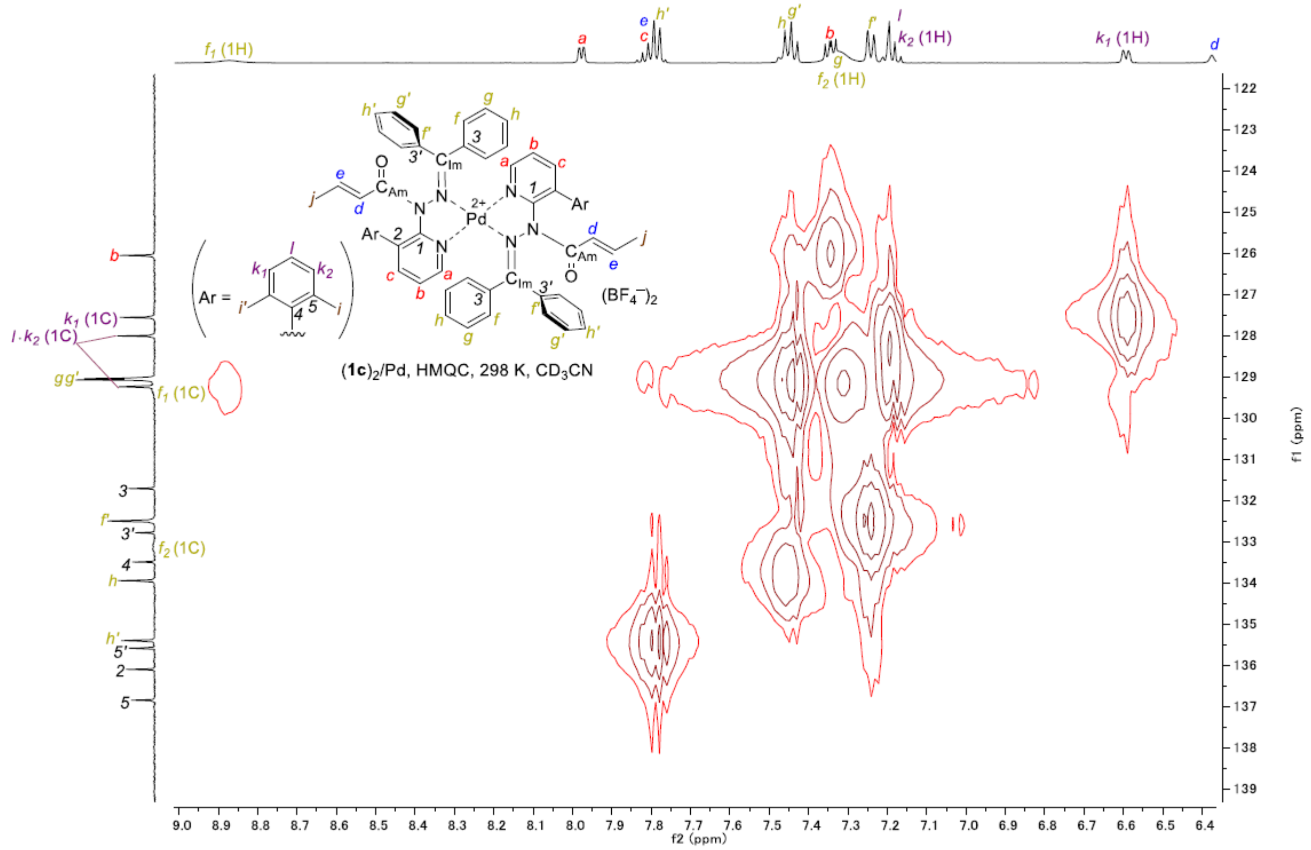
(1c)2/Pd, HMQC, 298 K, CD3CN
f2 (ppm)
f1 (ppm)

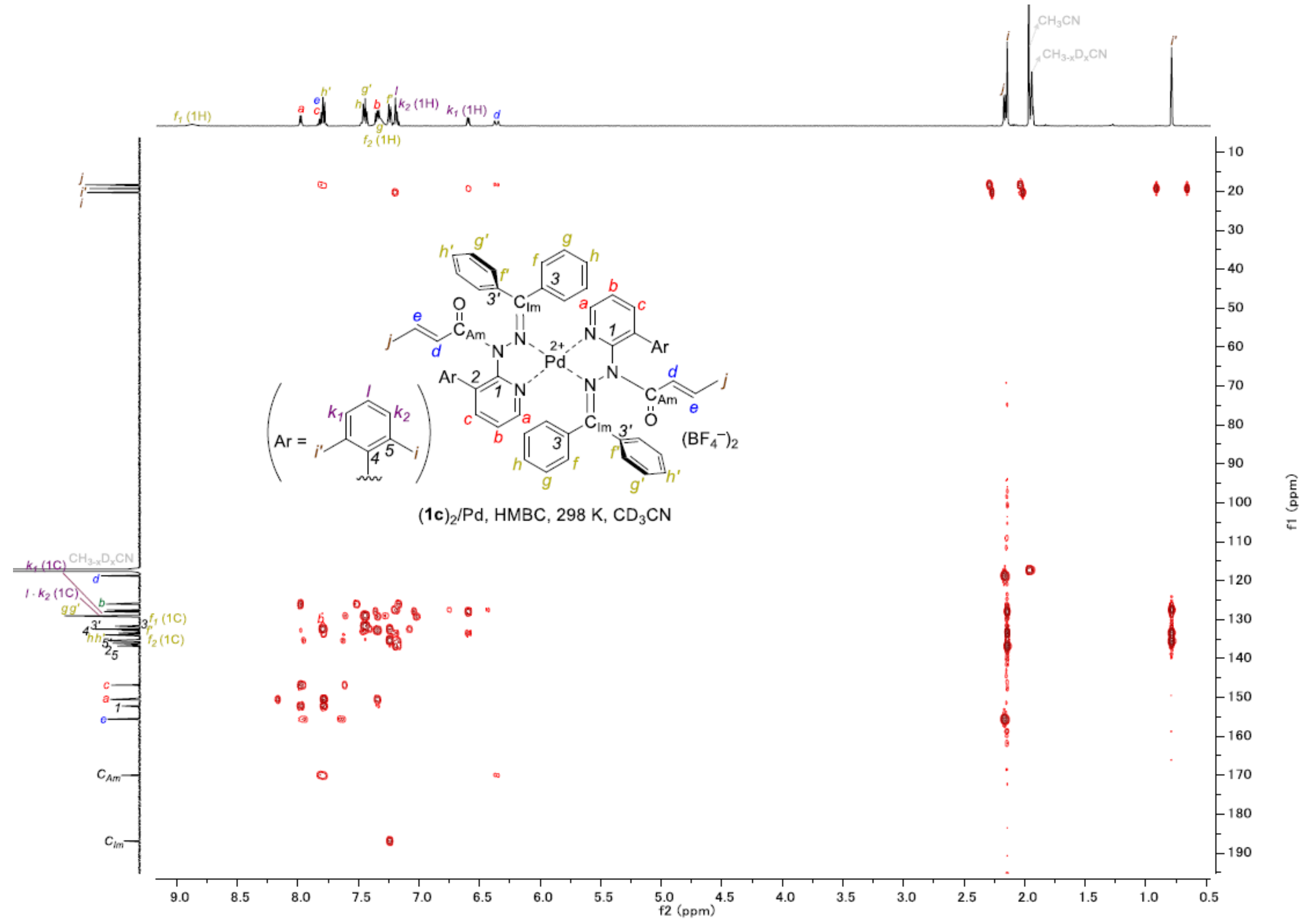
(1c)2/Pd, HMBC, 298 K, CD3CN
CH3CN
f2 (ppm)
f1 (ppm)

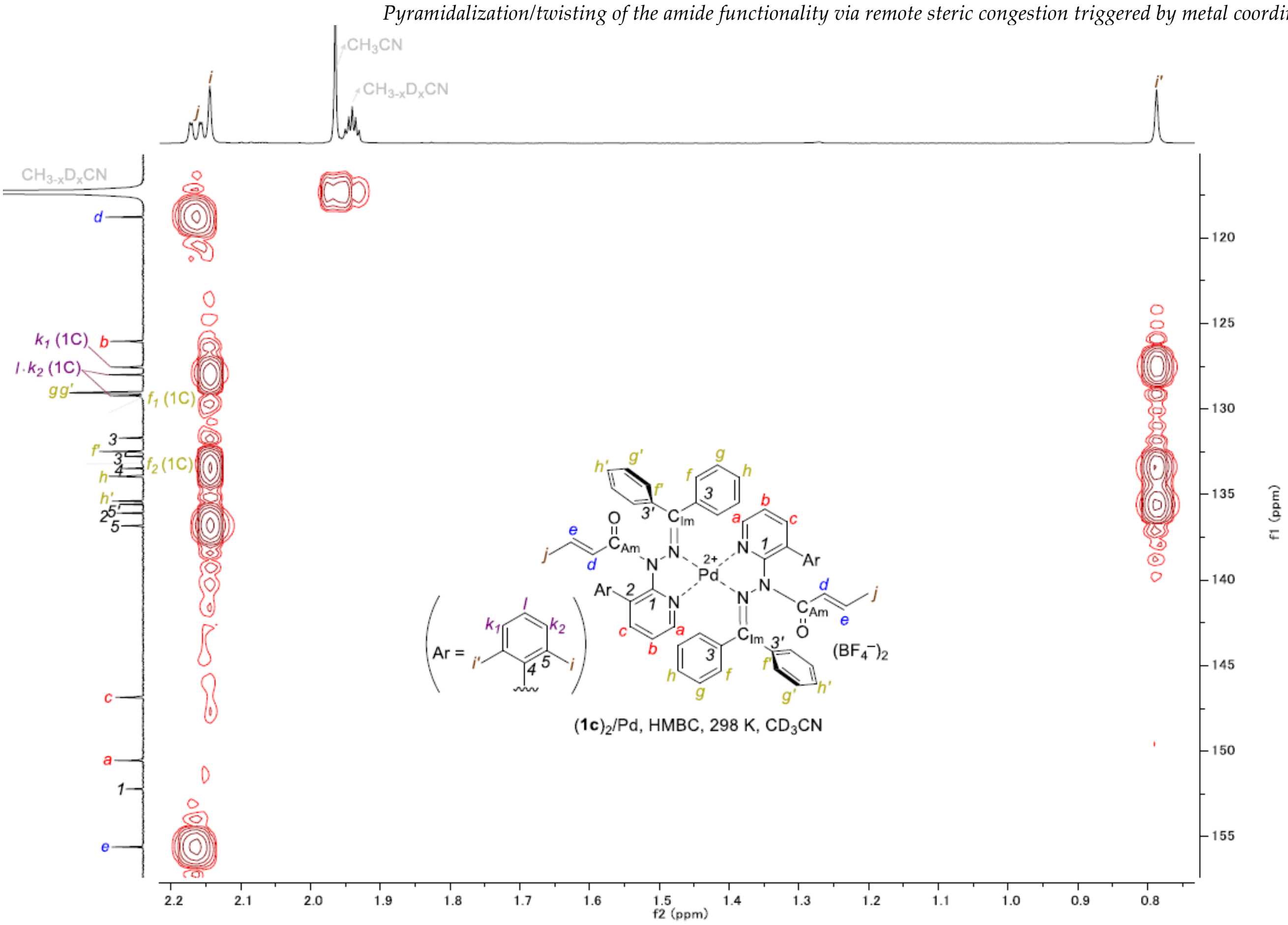
(1c)2/Pd, HMBC, 298 K, CD3CN
f2 (ppm)
f1 (ppm)

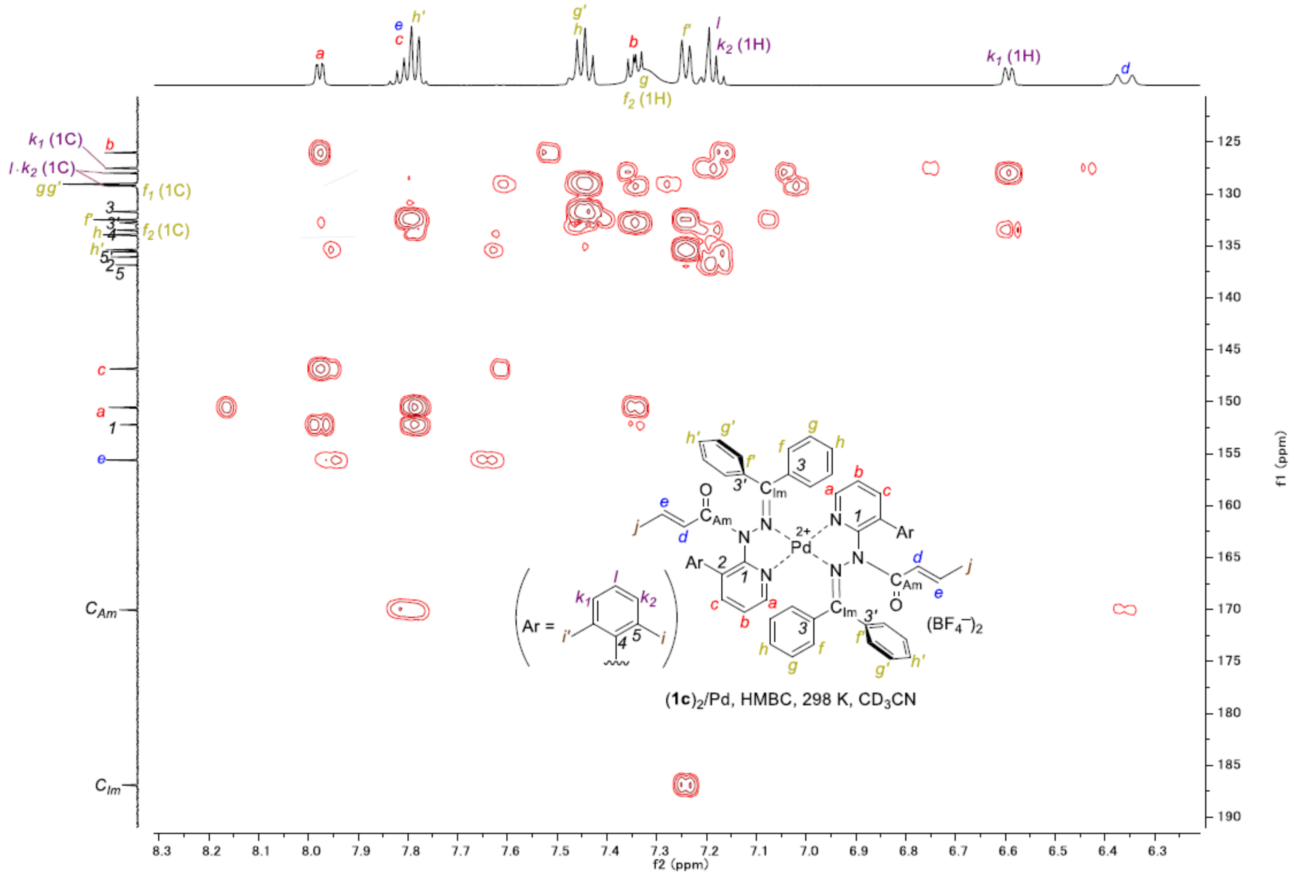
(1c)2/Pd, HMBC, 298 K, CD3CN
f2 (ppm)
f1 (ppm)

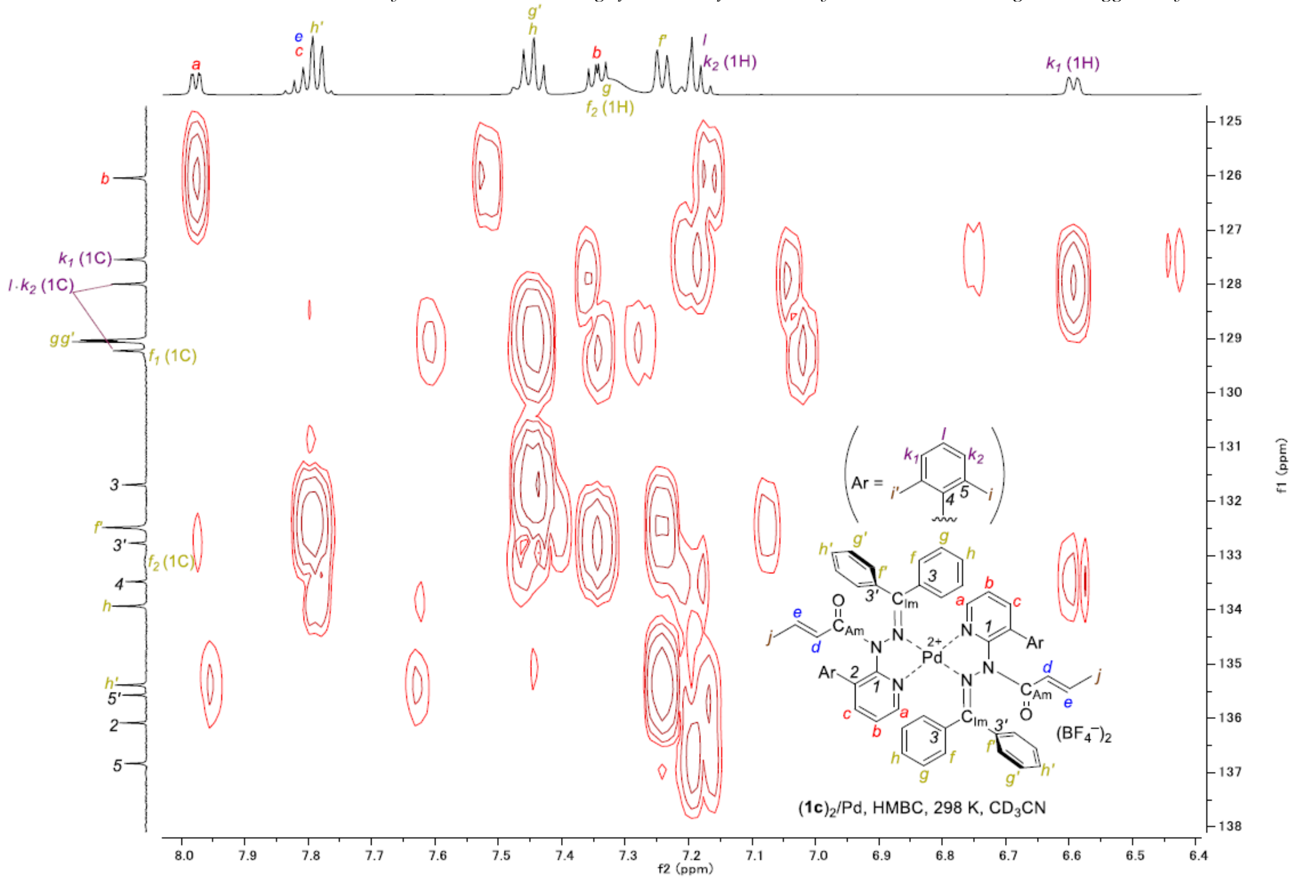
(1c)2/Pd, HMBC, 298 K, CD3CN
Ar =
(BF4−)2
k1 (1H)
k2 (1H)
f2 (1H)
k1 (1C)
l, k2 (1C)
gg′
f1 (1C)
f2 (1C)
f2 (ppm)
f1 (ppm)

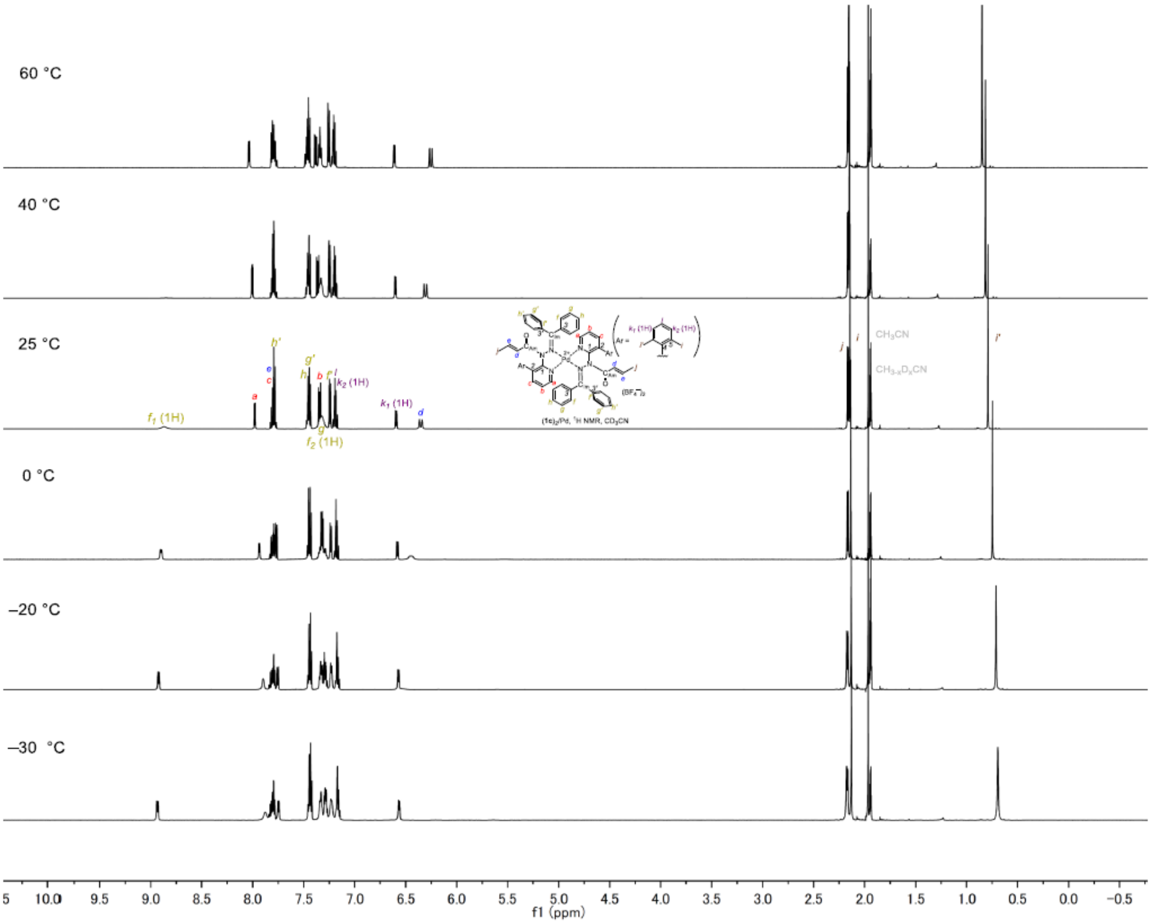
60 °C
40 °C
25 °C
0 °C
−20 °C
−30 °C
f1 (1H)
k2 (1H)
k1 (1H)
f2 (1H)
CH3CN
(1c)2/Pd, 1H NMR, CD3CN
f1 (ppm)

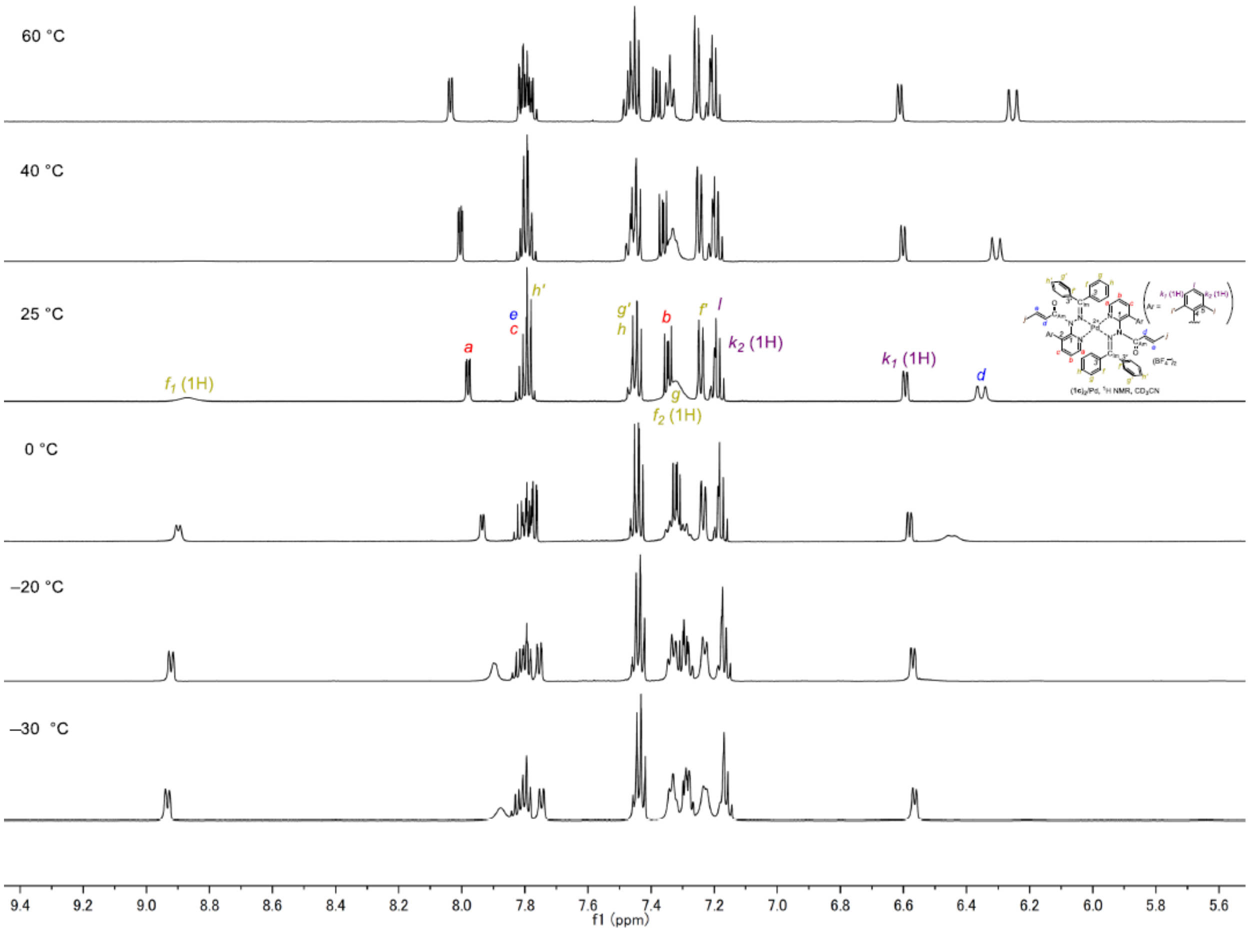
60 °C
40 °C
25 °C
0 °C
–20 °C
–30 °C
f1 (1H)
a
e
c
h'
g'
h
b
f'
l
k2 (1H)
g
f2 (1H)
k1 (1H)
d
(1c)2/Pd, 1H NMR, CD3CN
f1 (ppm)

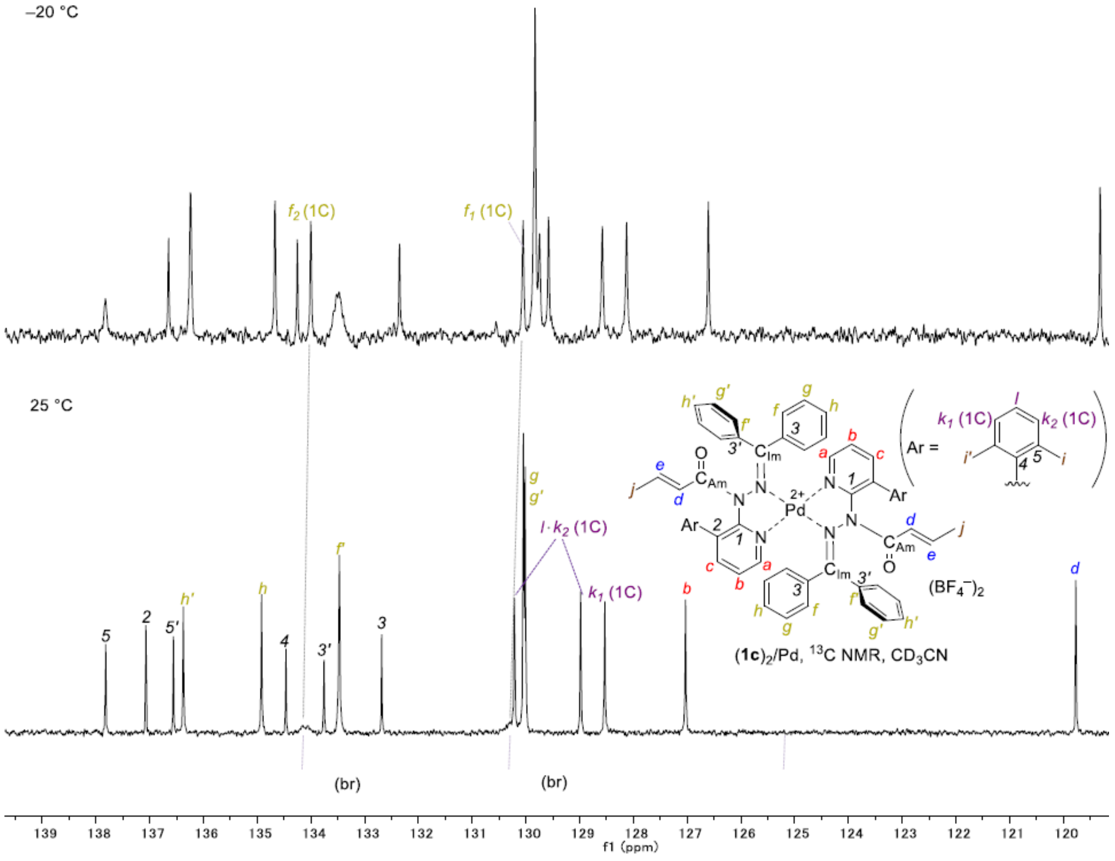
–20 °C
f2 (1C)
f1 (1C)
25 °C
5
2
5'
h'
h
4
3'
f'
3
g
g'
l·k2 (1C)
k1 (1C)
b
d
(br)
(br)
Ar =
k1 (1C)
l
k2 (1C)
i'
i
(BF4−)2
(1c)2/Pd, 13C NMR, CD3CN
f1 (ppm)

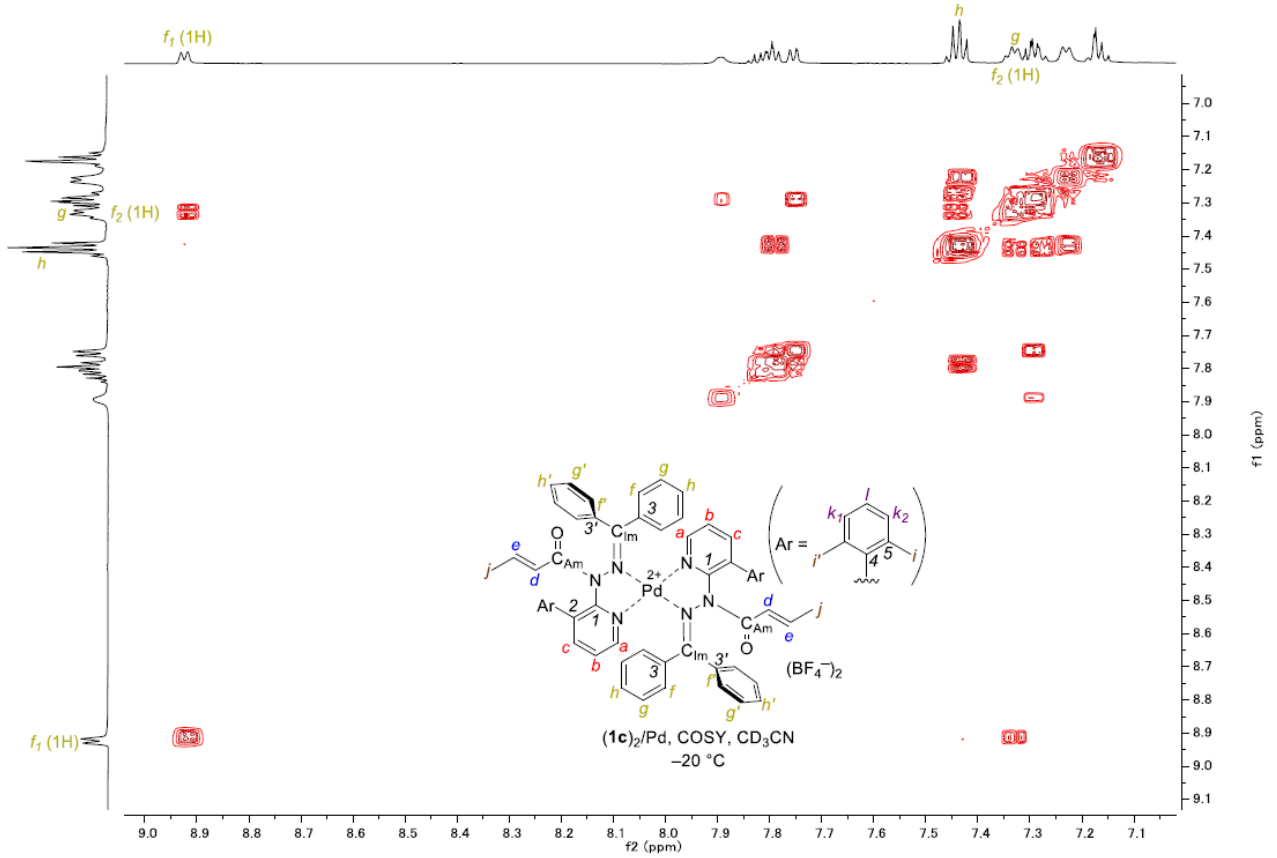
f1 (1H)
h
g
f2 (1H)
(1c)2/Pd, COSY, CD3CN
–20 °C
(BF4−)2
Ar =
f2 (ppm)
f1 (ppm)

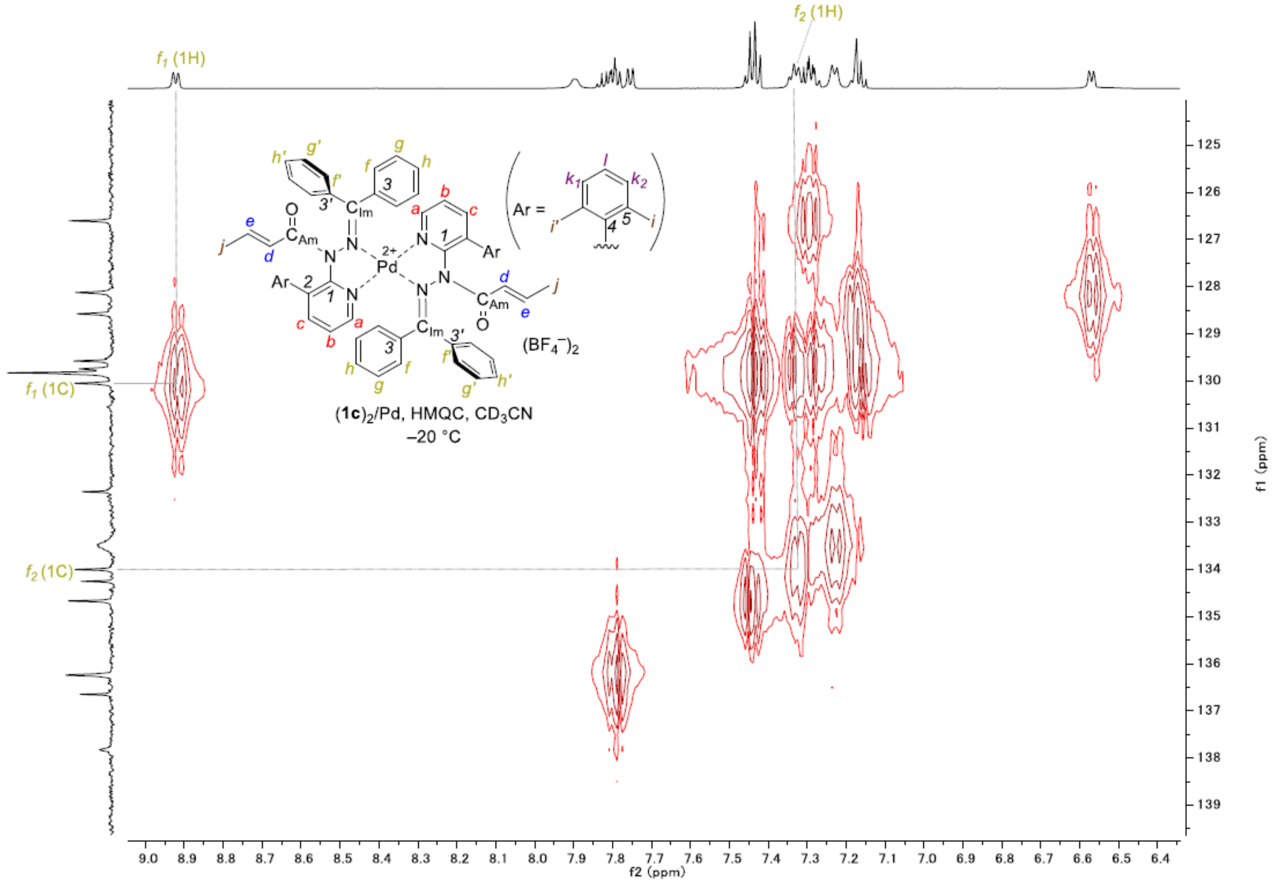
f1 (1H)
f2 (1H)
f1 (1C)
f2 (1C)
(1c)2/Pd, HMQC, CD3CN
–20 °C
(BF4−)2
Ar =
f2 (ppm)
f1 (ppm)

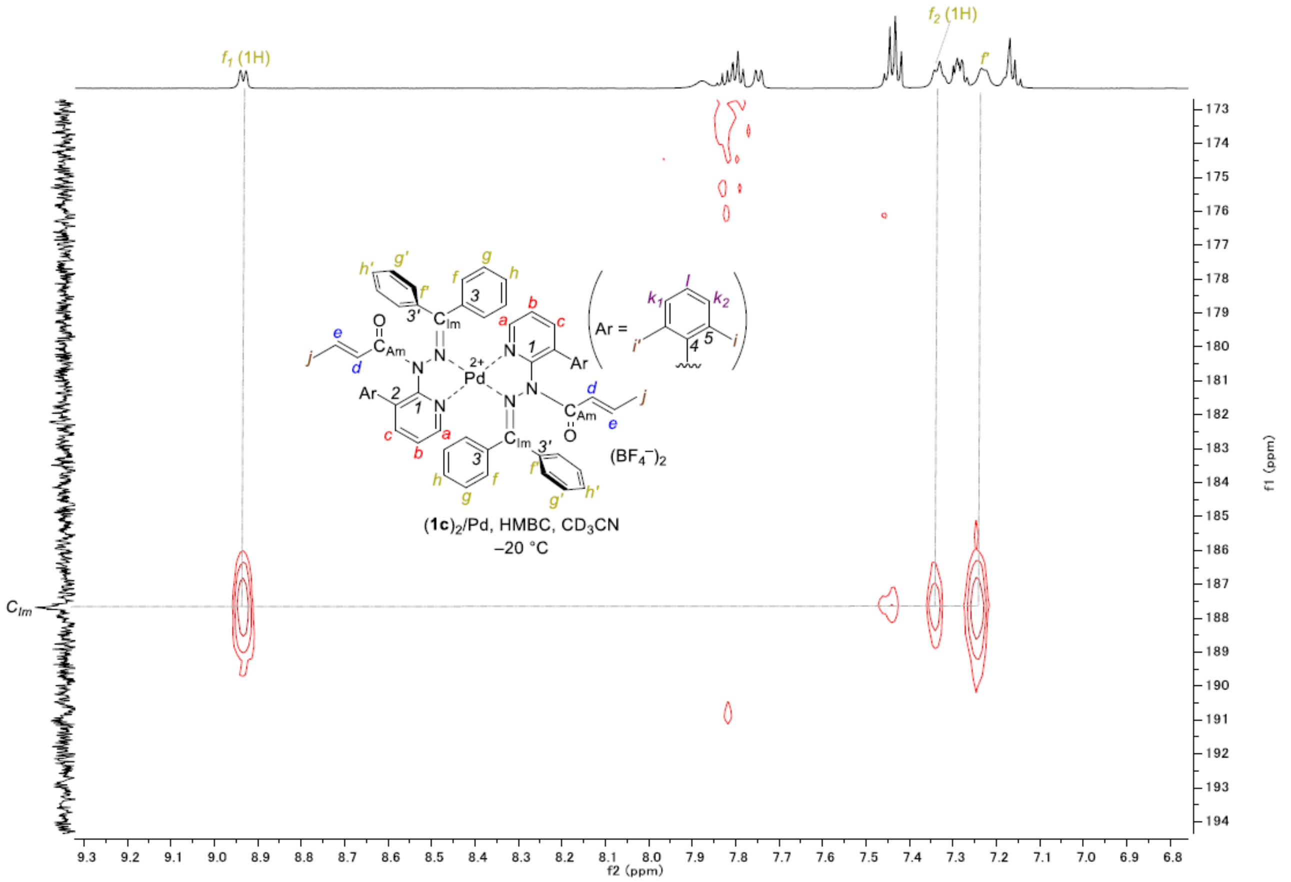
(1c)2/Pd, HMBC, CD3CN
–20 °C
f1 (1H)
f2 (1H)
CIm
Ar =
(BF4–)2
f2 (ppm)
f1 (ppm)

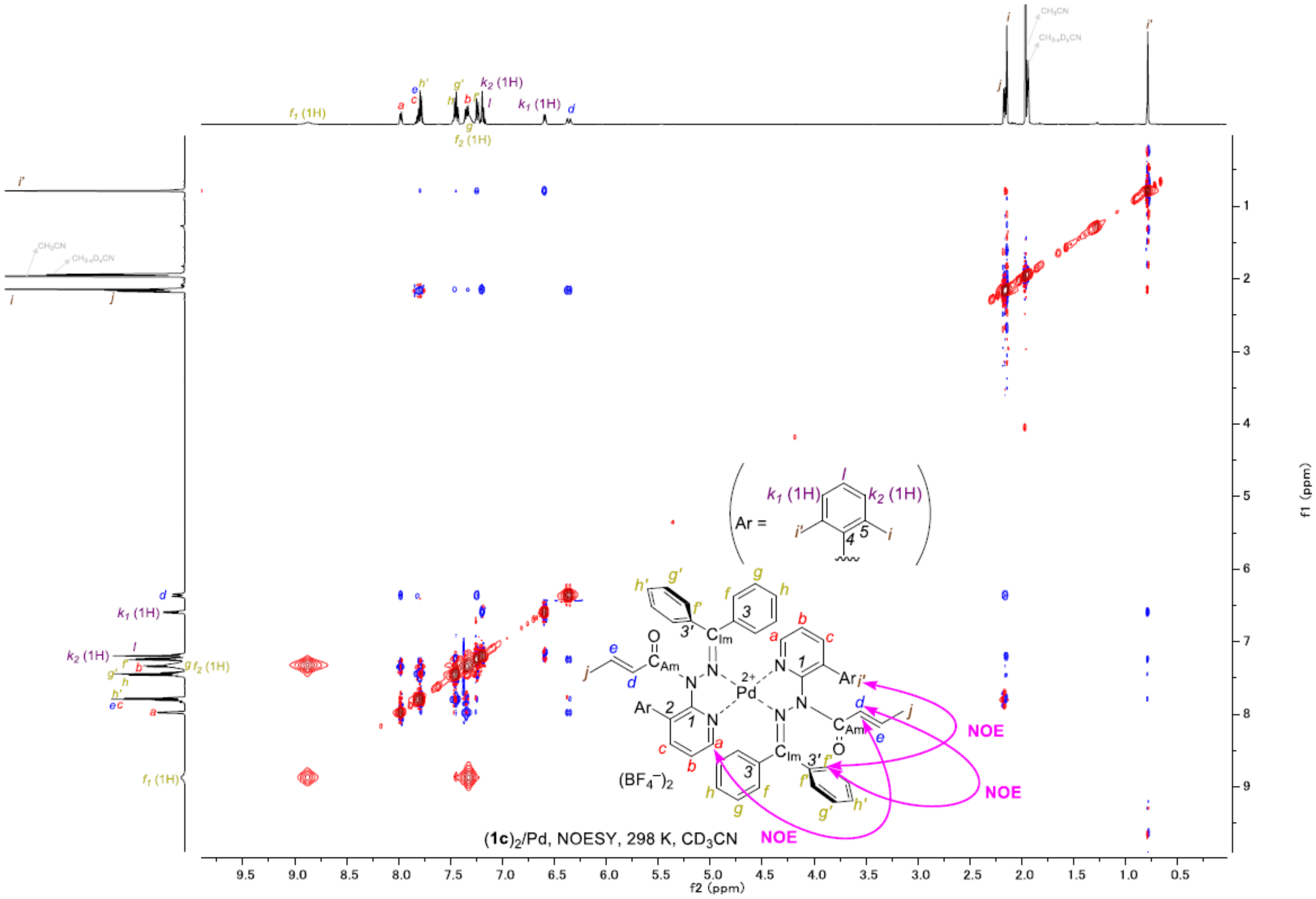
(1c)2/Pd, NOESY, 298 K, CD3CN
NOE
NOE
NOE
Ar =
(BF4–)2
f2 (ppm)
f1 (ppm)

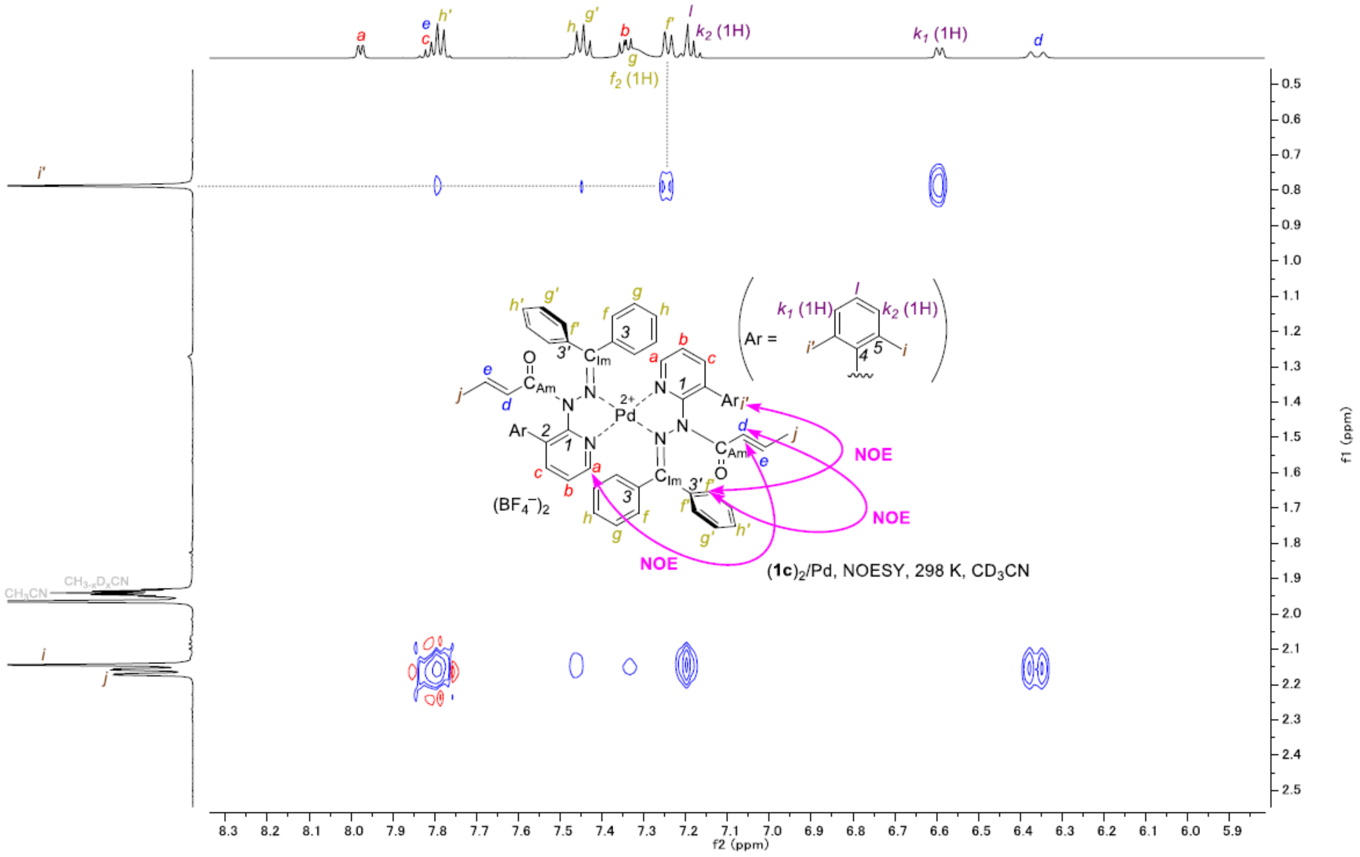
NOE
(1c)2/Pd, NOESY, 298 K, CD3CN
(BF4−)2
Ar =
k1 (1H)
k2 (1H)
f2 (1H)
CH3CN
CH3-xDxCN
f2 (ppm)
f1 (ppm)

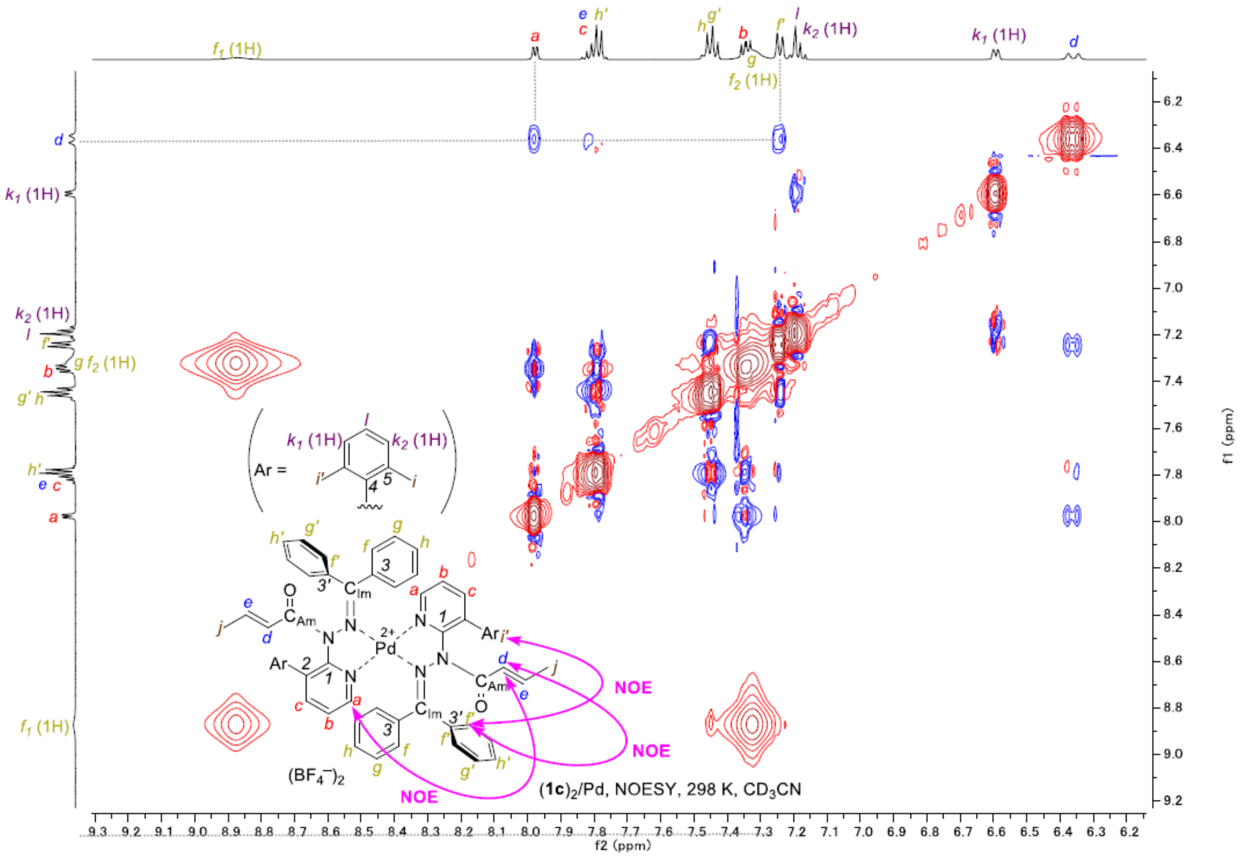
NOE
(1c)2/Pd, NOESY, 298 K, CD3CN
(BF4−)2
Ar =
f1 (1H)
k1 (1H)
k2 (1H)
f2 (1H)
f2 (ppm)
f1 (ppm)

3-3-3. Stacked ^{1}H and ^{13}C NMR of **1c** and (**1c**)$_2$/Pd

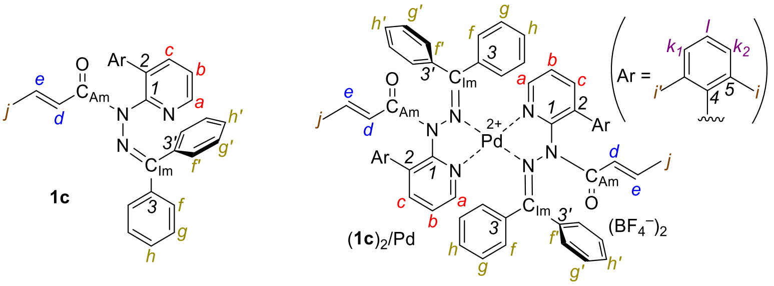

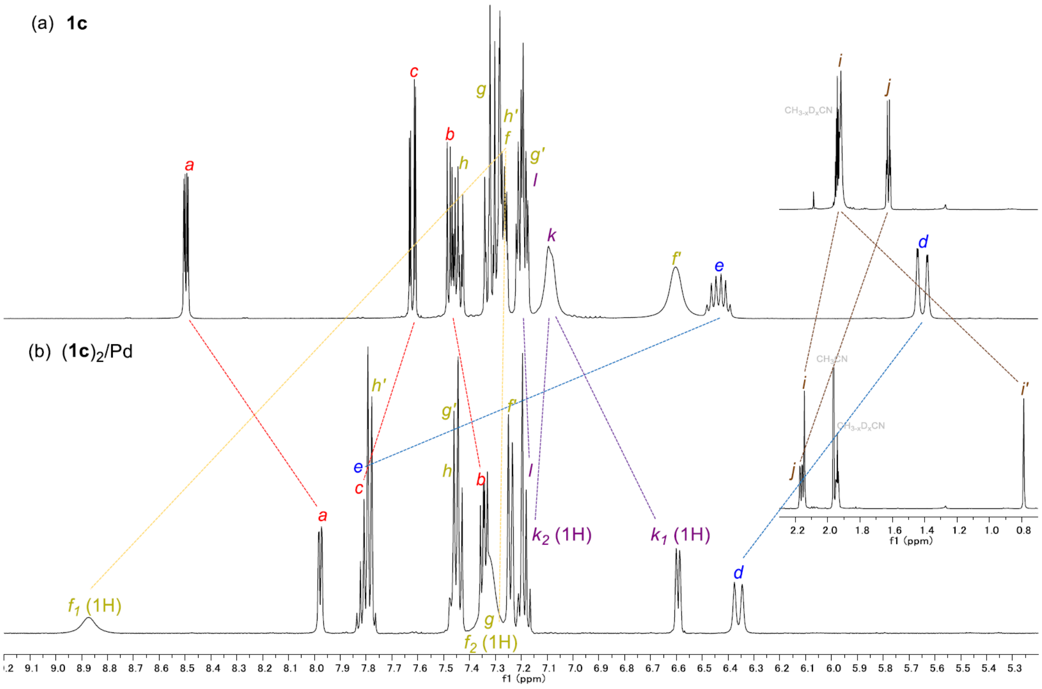

Figure S5. (a) ^{1}H NMR of **1c** in CD_3CN. (b) ^{1}H NMR of **1c**/[Pd(CH_3CN)$_4$](BF_4)$_2$ = 2/1 in CD_3CN. *f*▪*g*▪*h* and *f′*▪*g′*▪*h′* are not distinguished in (a).

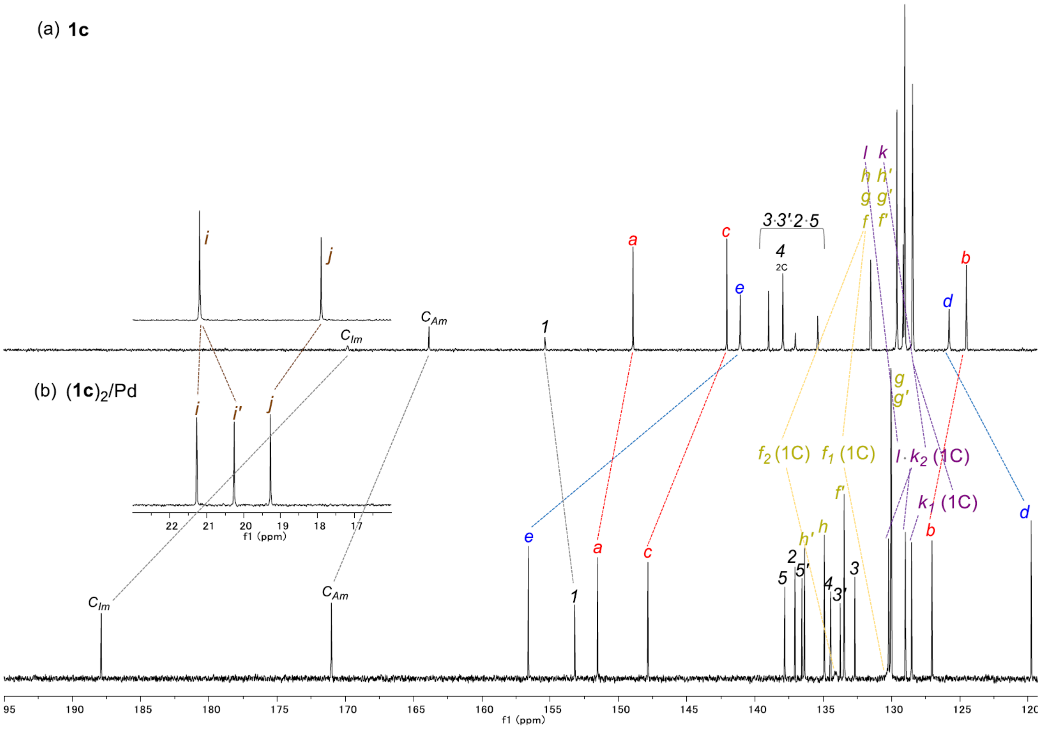

Figure S6. (a) ^{13}C NMR of **1c** in CD_3CN. (b) ^{13}C NMR of **1c**/[Pd(CH_3CN)$_4$](BF_4)$_2$ = 2/1 in CD_3CN. *f*▪*g*▪*h* 3 and *f′*▪*g′*▪*h′*▪3′ are not distinguished in (a)

*3-4-1. ^{1}H NMR of a 1:1 mixture of amide **1b** or **1c** with [Pd(CH$_3$CN)$_4$](BF$_4$)$_2$*

Although ^{1}H NMR of the mixture of **1a**:[Pd(CH$_3$CN)$_4$](BF$_4$)$_2$ = 1:1 in CD$_3$CN showed clean formation of **1a**/Pd (**1a**:Pd^{2+} = 1:1) (Figure S1b), **1b** and **1c** in NMR analysis under otherwise identical conditions gave two species, one of which is (**1**)$_2$/Pd (amide **1b** or **1c**: Pd^{2+} = 2:1) and another is assumed to be **1**/Pd (amide **1b** or **1c**: Pd^{2+} = 1:1) (Figure S7 and S8).

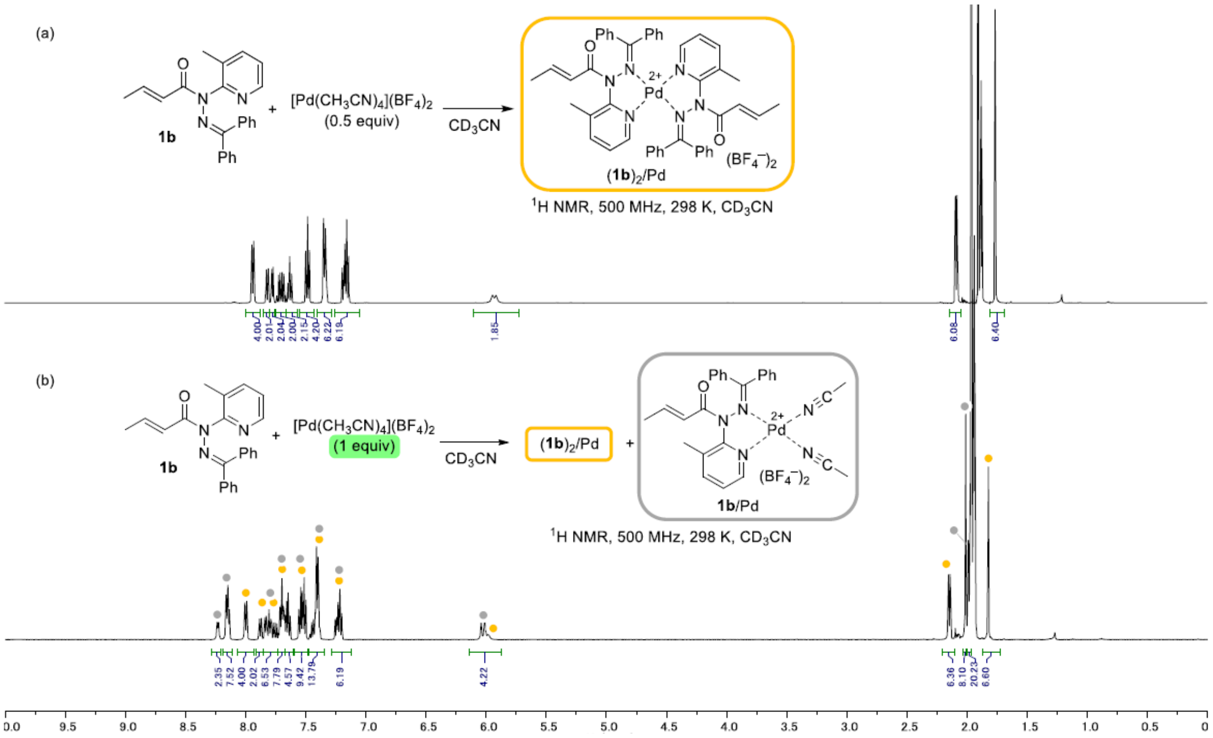

Figure S7. (a) ^{1}H NMR of **1c**/[Pd(CH$_3$CN)$_4$](BF$_4$)$_2$ = 2/1 in CD$_3$CN. (b) ^{1}H NMR of **1c**/[Pd(CH$_3$CN)$_4$](BF$_4$)$_2$ = 1/1 in CD$_3$CN.

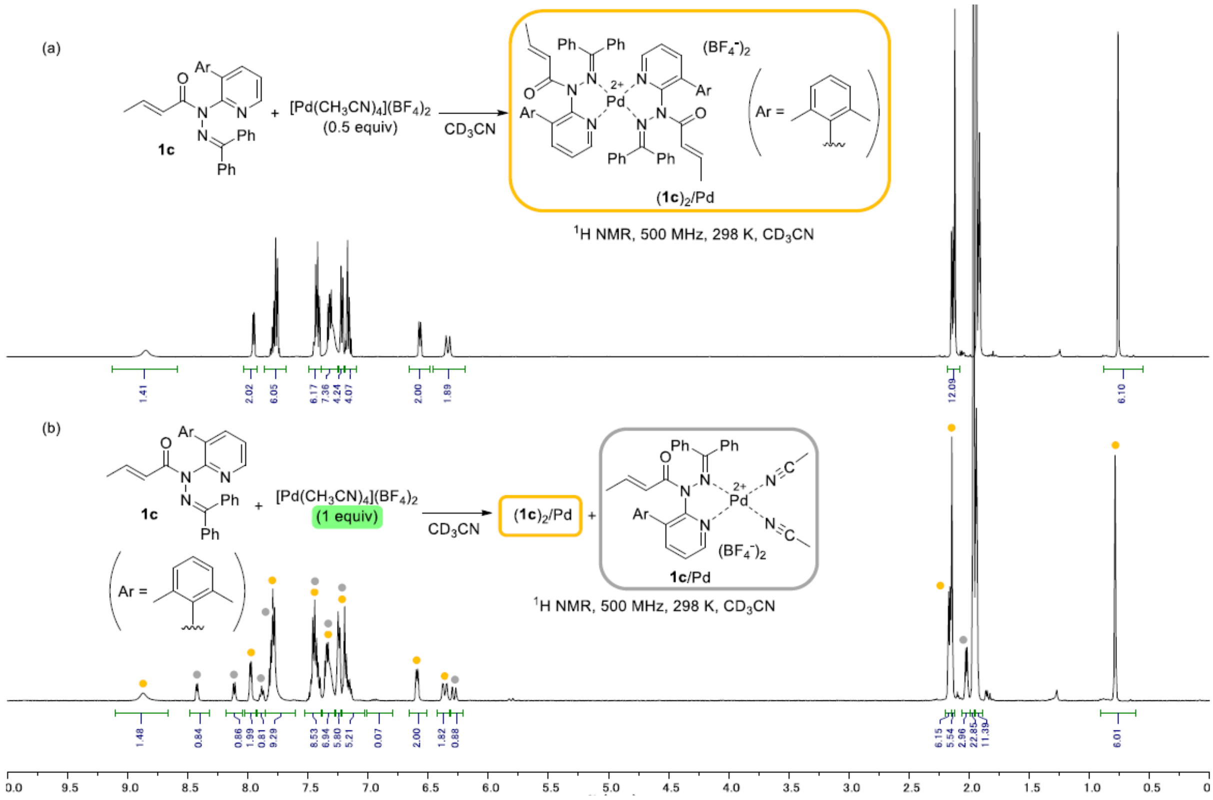

Figure S8. (a) ^{1}H NMR of **1c**/[Pd(CH$_3$CN)$_4$](BF$_4$)$_2$ = 2/1 in CD$_3$CN. (b) ^{1}H NMR of **1c**/[Pd(CH$_3$CN)$_4$](BF$_4$)$_2$ = 1/1 in CD$_3$CN.

*3-5-1. ¹H NMR of **1a**-metal complexes with variable stoichiometry*

The ¹H NMR of a mixture of (**1a**)$_2$/Pd with **1a** or **1a**/Pd showed the sum of the two individual spectra rather than an averaged spectrum (Figure S9). In contrast, ¹H NMR signals of a mixture of **1a** and Zn(OTf)$_2$ were averaged (Figure S10).

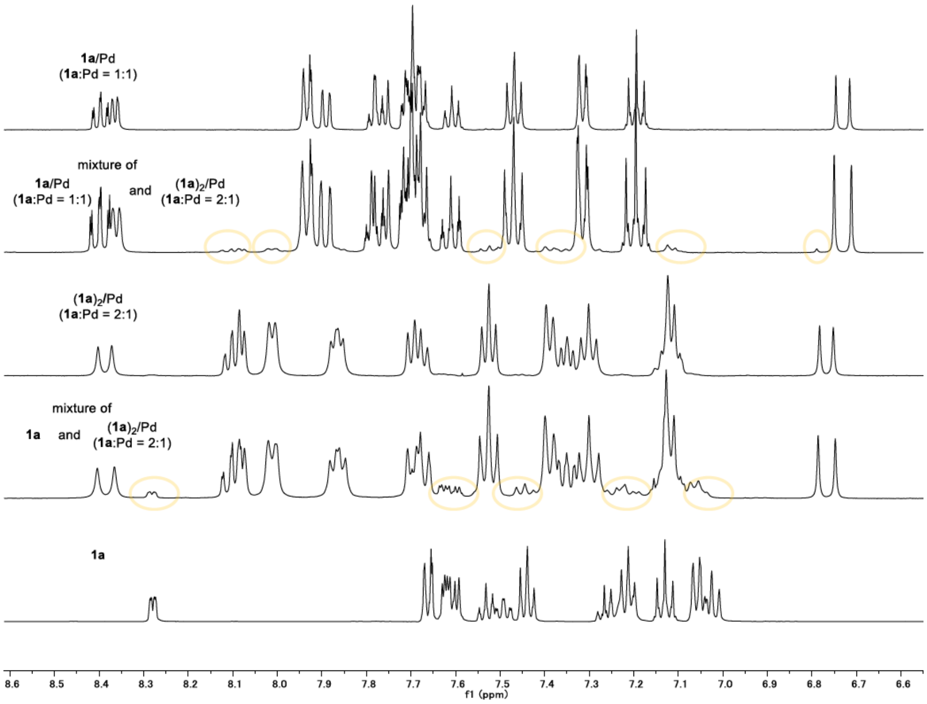

Figure S9. ¹H NMR of a mixture of (**1a**)$_2$Pd with **1a** or **1a**/Pd.

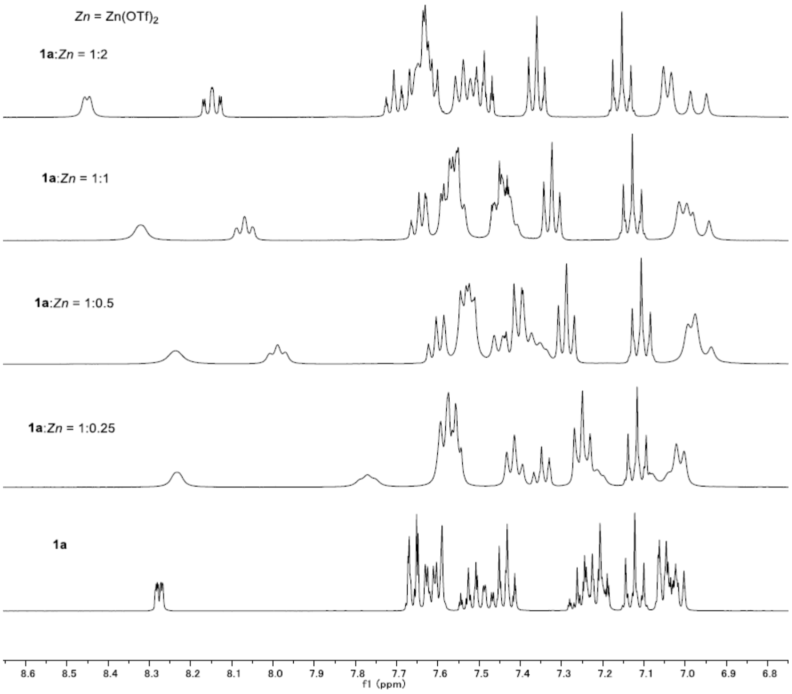

Figure S10. (a) Averaged ¹H NMR of a mixture of **1a** and Zn(OTf)$_2$

4. Crystal Structures of Amides 1a–c and Their Pd Complexes

4-1. Crystal structures of amides 1a–c

A crystal of **1a** was obtained by vapor diffusion of hexane into an acetone solution of **1a**. All measurements were made on a Rigaku XtaLAB P200 diffractometer using multi-layer mirror monochromated Cu-Kα radiation. All hydrogen atoms were placed in standard calculated positions, and were refined with an isotropically. Refined structure and crystallographic parameters are summarized in Figure S11 and Table S1. CCDC 1495002 contains the supplementary crystallographic data for **1a**.

(a) **(b)**

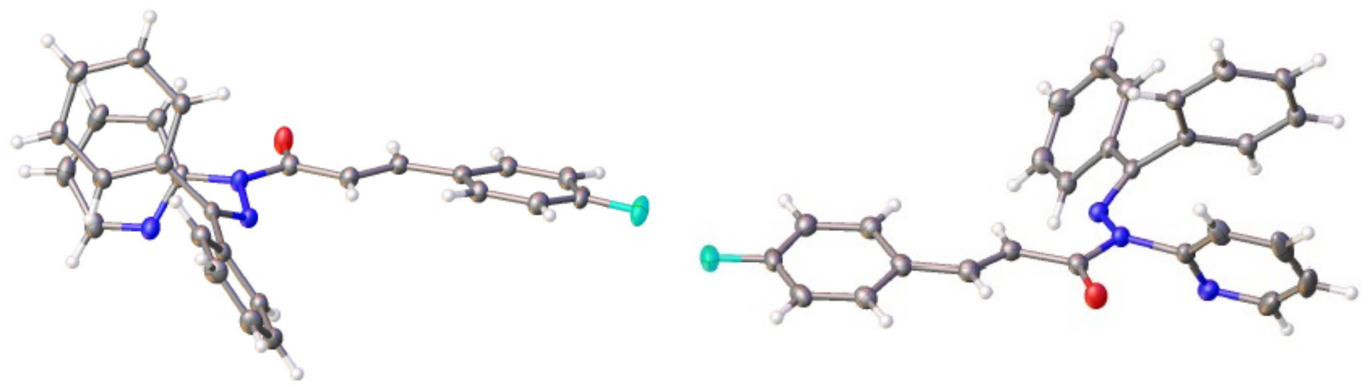

(a) χ_N = 19.6°, χ_C = 1.8°, τ = 1.8°
C_{Am}=O: 1.227(1) Å, N–C_{Am}(=O): 1.386(1) Å

(b) χ_N = 20.7°, χ_C = 2.2°, τ = 2.9°
C_{Am}=O: 1.226(1) Å, N–C_{Am}(=O): 1.384(1) Å

Figure S11. Structure of 1a. [ORTEP plot (50% ellipsoids)], Color code; gray: C, white: H, blue: N, red: O, skyblue: F
(Two structures were found per unit cell.)

Table S1. Selected crystal data of 1a

Empirical Formula	$C_{54}H_{40}F_2N_6O_2$
Formula Weight	842.95
Temperature/°C	–180.0
Crystal Color, Habit	colourless, prism
Crystal Dimensions	0.259 x 0.117 x 0.067 mm
Crystal System	triclinic
Cell constants	
a	12.2208(5) Å
b	12.9781(4) Å
c	15.6515(3) Å
V	2184.84(13) $Å^3$
Space Group	*P*–1 (#2)
Z value	2
D_{calc}	1.281 g/cm^3
F_{000}	880.00
No. of Reflections Measured	Total: 26191
	Unique: 7535 (R_{int} = 0.0825)
R_1 (I>2.00σ(I))	0.0358
R (All reflections)	0.0398
wR_2 (All reflections)	0.1020

A crystal of **1b** was obtained by vapor diffusion of *i*-Pr_2O into an CH_3CN solution of **1b**. All measurements were made on a Rigaku R-AXIS RAPID diffractometer using filtered Cu-Kα radiation. All hydrogen atoms were placed in standard calculated positions, and were refined with an isotropically. Refined structure and crystallographic parameters are summarized in Figure S12 and Table S2. CCDC 1495001 contains the supplementary crystallographic data for **1b**.

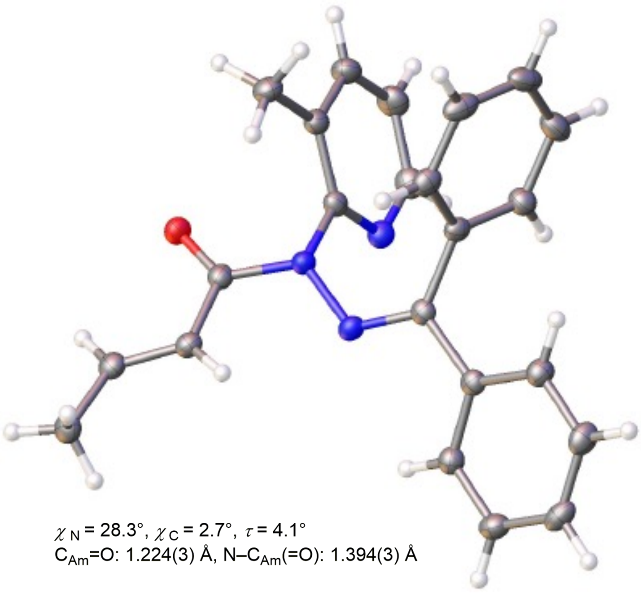

Figure S12. Structure of 1b. [ORTEP plot (50% ellipsoids)], Color code; gray: C, white: H, blue: N, red: O

Table S2. Selected crystal data of 1b

Empirical Formula	$C_{23}H_{21}N_3O$
Formula Weight	355.44
Temperature/°C	–180.0
Crystal Color, Habit	colorless, platelet
Crystal Dimensions	0.200 x 0.200 x 0.200 mm
Crystal System	monoclinic
Cell constants	
a	11.3565(2) Å
b	11.5389(2) Å
c	15.0221(3) Å
V	1878.76(10) $Å^3$
Space Group	$P2_1/n$ (#14)
Z value	4
D_{calc}	1.257 g/cm^3
F_{000}	752.00
No. of Reflections Measured	Total: 21297
	Unique: 3434 (R_{int} = 0.0330)
R_1 (I>2.00σ(I))	0.0575
R (All reflections)	0.0636
wR_2 (All reflections)	0.1613

A crystal of **1c** was obtained by vapor diffusion of *i*-Pr_2O into an CH_3CN solution of **1c**. All measurements were made on a Rigaku R-AXIS RAPID diffractometer using filtered Cu-Kα radiation. All hydrogen atoms were placed in standard calculated positions, and were refined with an isotropically. Refined structure and crystallographic parameters are summarized in Figure S13 and Table S3. CCDC 1495003 contains the supplementary crystallographic data for **1c**.

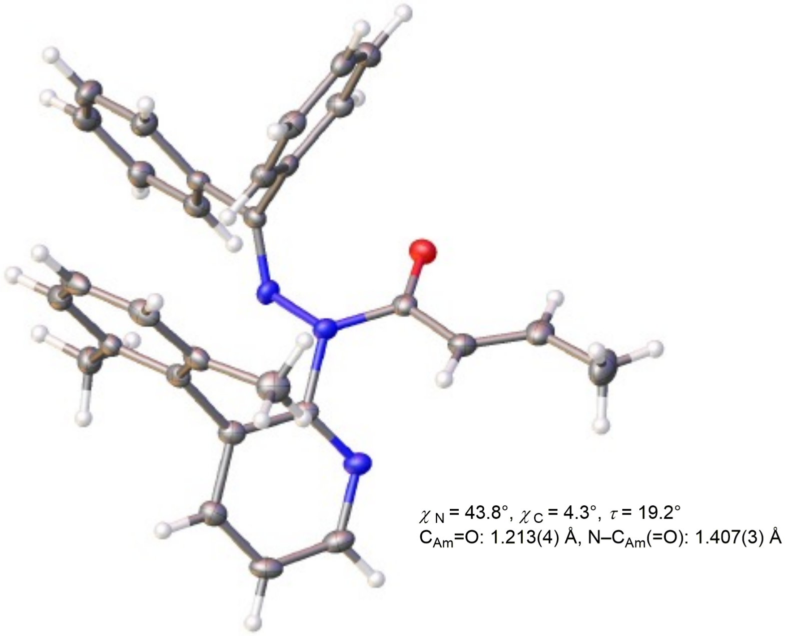

Figure S13. Structure of 1c. [ORTEP plot (50% ellipsoids)], Color code; gray: C, white: H, blue: N, red: O

Table S3. Selected crystal data of 1c

Empirical Formula	$C_{30}H_{27}N_3O$
Formula Weight	445.56
Temperature/°C	–180.0
Crystal Color, Habit	colorless, block
Crystal Dimensions	0.200 x 0.200 x 0.200 mm
Crystal System	monoclinic
Cell constants	
a	18.8592(4) Å
b	7.68843(16) Å
c	18.4442(3) Å
V	2438.13(17) Å^3
Space Group	$P2_1$/c (#14)
Z value	4
D_{calc}	1.214 g/cm^3
F_{000}	944.00
No. of Reflections Measured	Total: 25572
	Unique: 4459 (R_{int} = 0.0902)
R_1 (I>2.00σ(I))	0.0550
R (All reflections)	0.1009
wR_2 (All reflections)	0.1747

<u>*4-2. Crystal structures of metal complexes with amides **1a–c***</u>

A crystal of (**1a**)$_2$/Pd was obtained by vapor diffusion of hexane into an acetone solution of (**1a**)$_2$/Pd. All measurements were made on a Rigaku R-AXIS RAPID diffractometer using filtered Cu-Kα radiation. All hydrogen atoms were placed in standard calculated positions, and were refined with an isotropically. Refined structure and crystallographic parameters are summarized in Figure S14 and Table S4. CCDC 1495004 contains the supplementary crystallographic data for (**1a**)$_2$/Pd.

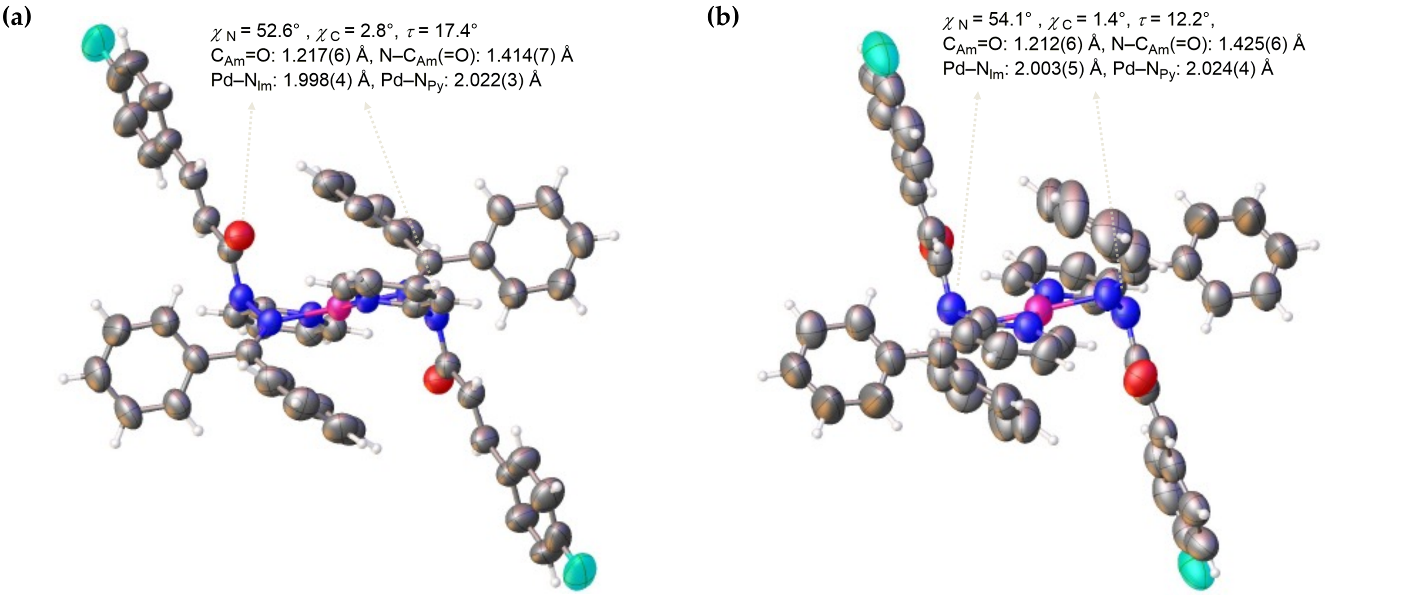

Figure S14. Structure of (1a)$_2$/Pd. [ORTEP plot (50% ellipsoids)], Color code; gray: C, white: H, blue: N, red: O, skyblue: F, yellow: S, pink: Pd
(Two structures were found per unit cell.)

Table S4. Selected crystal data of (1a)$_2$/Pd.

Empirical Formula	$C_{54}H_{40}F_2N_6O_2Pd$
Formula Weight	949.35
Temperature/°C	–180.0
Crystal Color, Habit	yellow, platelet
Crystal Dimensions	0.200 x 0.200 x 0.050 mm
Crystal System	triclinic
Cell constants	
a	10.7056(3) Å
b	16.7664(4) Å
c	18.6108(5) Å
V	3174.3(2) Å^3
Space Group	*P*–1 (#2)
Z value	2
D_{calc}	0.993 g/cm^3
F_{000}	972.00
No. of Reflections Measured	Total: 37622
	Unique: 11413 (R_{int} = 0.0884)
R_1 (I>2.00σ(I))	0.0659
R (All reflections)	0.0797
wR_2 (All reflections)	0.2041

A crystal of (**1b**)$_2$/Pd was obtained by vapor diffusion of *i*-Pr_2O into an CH_3CN solution of (**1b**)$_2$/Pd. All measurements were made on a Rigaku R-AXIS RAPID diffractometer using filtered Cu-Kα radiation. All hydrogen atoms were placed in standard calculated positions, and were refined with an isotropically. Refined structure and crystallographic parameters are summarized in Figure S15 and Table S5. CCDC 1494999 contains the supplementary crystallographic data for (**1b**)$_2$/Pd.

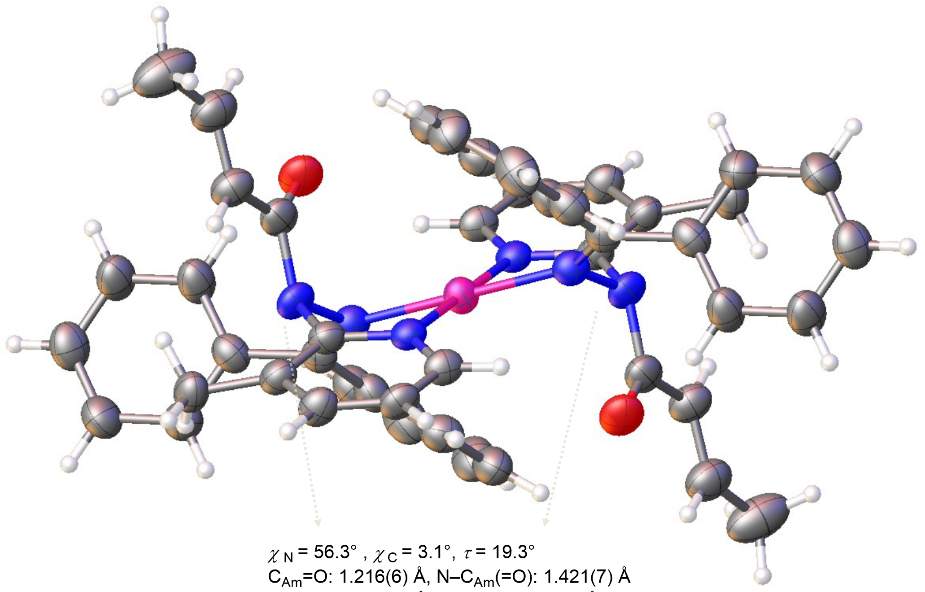

Figure S15. Structure of (1b)$_2$/Pd. [ORTEP plot (50% ellipsoids)], Color code; gray: C, white: H, blue: N, red: O, pink: Pd

Table S5. Selected crystal data of (1b)$_2$/Pd

Empirical Formula	$C_{46}H_{42}N_6O_2Pd$
Formula Weight	817.28
Temperature/°C	–180.0
Crystal Color, Habit	colorless, block
Crystal Dimensions	0.200 x 0.200 x 0.200 mm
Crystal System	triclinic
Cell constants	
a	8.88787(16) Å
b	10.65997(19)
c	12.1038(2) Å
V	1069.19(7) Å^3
Space Group	*P*–1 (#2)
Z value	1
D_{calc}	1.269 g/cm^3
F_{000}	422.00
No. of Reflections Measured	Total: 12558
	Unique: 3851 (R_{int} = 0.0638)
R_1 (I>2.00σ(I))	0.0541
R (All reflections)	0.0617
wR_2 (All reflections)	0.1453

A crystal of (**1c**)$_2$/Pd was obtained by vapor diffusion of *i*-Pr_2O into an CH_3CN solution of (**1c**)$_2$/Pd. All measurements were made on a Rigaku R-AXIS RAPID diffractometer using filtered Cu-Kα radiation. All hydrogen atoms were placed in standard calculated positions, and were refined with an isotropically. Refined structure and crystallographic parameters are summarized in Figure S16 and Table S6. CCDC 1495000 contains the supplementary crystallographic data for (**1c**)$_2$/Pd.

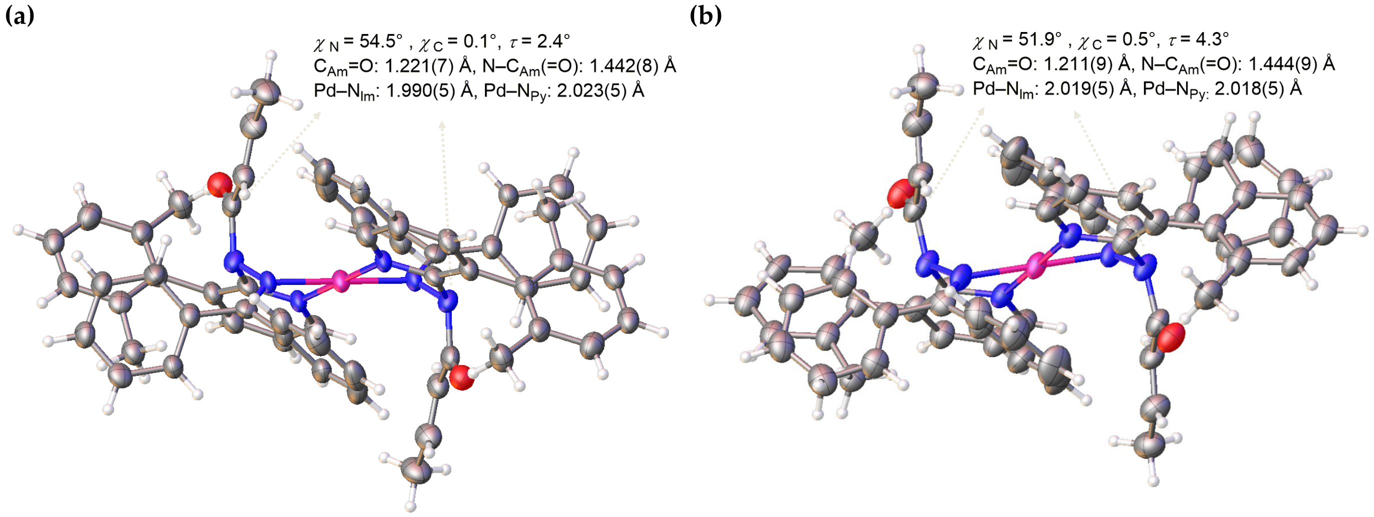

Figure S16. Structure of (1c)$_2$/Pd. [ORTEP plot (50% ellipsoids)], Color code; gray: C, white: H, blue: N, red: O, pink: Pd (Two structures were found per unit cell.)

Table S6. Selected crystal data of (1c)$_2$/Pd.

Empirical Formula	$C_{60}H_{55}B_2F_8N_6O_2Pd$
Formula Weight	1172.14
Temperature/°C	–180.0
Crystal Color, Habit	yellow, platelet
Crystal Dimensions	0.300 x 0.200 x 0.020 mm
Crystal System	triclinic
Cell constants	
a	13.2547(2) Å
b	14.2678(3) Å
c	15.5271(3) Å
V	2805.98(15) $Å^3$
Space Group	*P*–1 (#2)
Z value	2
D_{calc}	1.387 g/cm^3
F_{000}	1202.00
No. of Reflections Measured	Total: 33116
	Unique: 10068 (R_{int} = 0.0714)
R_1 (I>2.00σ(I))	0.0858
R (All reflections)	0.1110
wR_2 (All reflections)	0.2542

A crystal of **1c**/Pd was obtained by vapor diffusion of *i*-Pr_2O into an CH_3CN solution of **1c**/Pd. All measurements were made on a Rigaku R-AXIS RAPID diffractometer using filtered Cu-Kα radiation. All hydrogen atoms were placed in standard calculated positions, and were refined with an isotropically. Refined structure and crystallographic parameters are summarized in Figure S17 and Table S7. CCDC 1494998 contains the supplementary crystallographic data for **1c**/Pd.

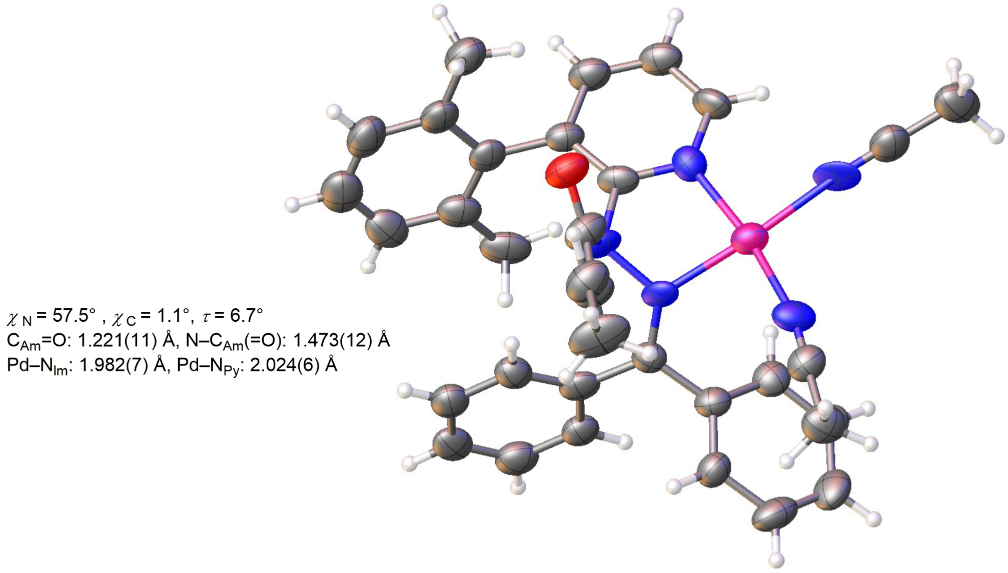

Figure S17. Structure of 1c/Pd. [ORTEP plot (50% ellipsoids)], Color code; gray: C, white: H, blue: N, red: O, pink: Pd

Table S7. Selected crystal data of 1c/Pd

Empirical Formula	$C_{34}H_{33}B_2F_8N_5OPd$
Formula Weight	807.67
Temperature/°C	–180.0
Crystal Color, Habit	yellow, platelet
Crystal Dimensions	0.200 x 0.200 x 0.050 mm
Crystal System	triclinic
Cell constants	
a	9.1646(3) Å
b	10.8129(4) Å
c	17.3580(8) Å
V	1703.87(11) $Å^3$
Space Group	*P*–1 (#2)
Z value	2
D_{calc}	1.574 g/cm^3
F_{000}	816.00
No. of Reflections Measured	Total: 20068
	Unique: 6102 (R_{int} = 0.1098)
R_1 (I>2.00σ(I))	0.0954
R (All reflections)	0.1223
wR_2 (All reflections)	0.2774

A crystal of (**1a**)$_2$/Zn was obtained by vapor diffusion of diisoporpyl ether into an CH_3CN solution of (**1a**)$_2$/Zn. All measurements were made on a Rigaku XtaLAB P200 diffractometer using multi-layer mirror monochromated Mo-Kα radiation. All hydrogen atoms were placed in standard calculated positions, and were refined with an isotropically. Refined structure and crystallographic parameters are summarized in Figure S18 and Table S8. CCDC 1495005 contains the supplementary crystallographic data for (**1a**)$_2$/Zn.

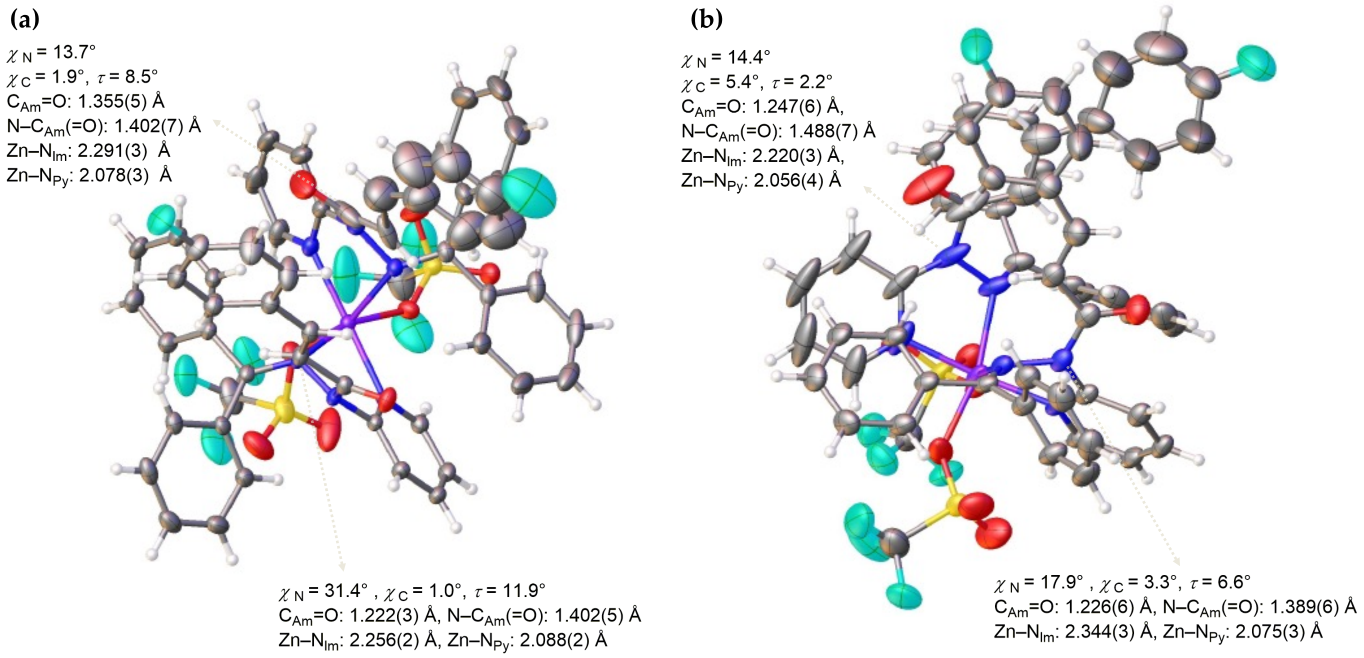

Figure S18. Structure of (1a)$_2$/Zn. [ORTEP plot (50% ellipsoids)], Color code; gray: C, white: H, blue: N, red: O, skyblue: F, yellow: S, purple: Zn
(Two structures were found per unit cell.)

Table S8. Selected crystal data of (1a)$_2$/Zn.

Empirical Formula	$C_{57}H_{41.5}F_8N_{6.5}O_8S_2Zn$
Formula Weight	1226.98
Temperature/°C	–180.0
Crystal Color, Habit	yellow, block
Crystal Dimensions	0.175 x 0.159 x 0.120 mm
Crystal System	triclinic
Cell constants	
a	15.7265(3) Å
b	20.4033(4) Å
c	21.0479(3) Å
V	6230.5(2) Å^3
Space Group	*P*–1 (#2)
Z value	4
D_{calc}	1.308 g/cm^3
F_{000}	2508.00
No. of Reflections Measured	Total: 122871
	Unique: 28530 (R_{int} = 0.0255)
R_1 (I>2.00σ(I))	0.0721
R (All reflections)	0.0818
wR_2 (All reflections)	0.2052

5. References

1 G. R. Fulmer, A. J. M. Miller, N. H. Sherden, H. E. Gottlieb, A. Nudelman, B. M. Stoltz, J. E. Bercaw, K. I. Goldberg, *Organometallics*, 2010, **29**, 2176–2179.
2 J. B. Arterburn, K. V. Rao, R. Ramdas, B. R. Dible, *Org. Lett.*, 2001, **3**, 1351–1354.
3 T. Q. Hung, T. T. Dang, J. Janke, A. Villinger, P. Langer, *Org. Biomol. Chem.*, 2015, **13**, 1375–1386.
4 X. Li, L. He, H. Chen, W. Wu, H. Jiang, *J. Org. Chem.*, 2013, **78**, 3636–3646.
5 G. Xie, P. Chellan, J. Mao, K. Chibale, G. S. Smith, *Adv. Synth. Catal.*, 2010, **352**, 1641–1647.

6. NMR Spectra of New Compounds (¹H, ¹³C, and ¹⁹F NMR spectra)

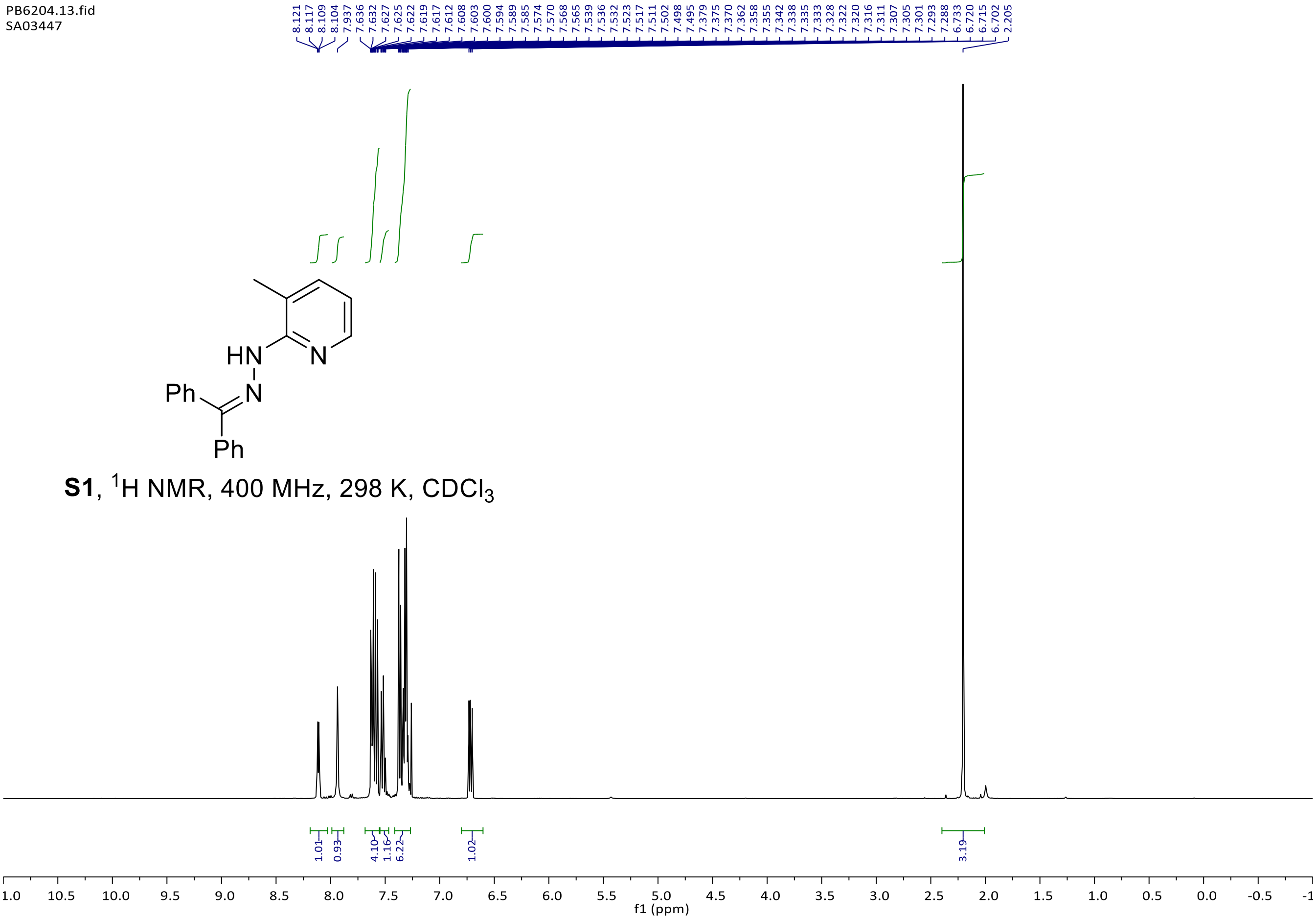

S1, ^{1}H NMR, 400 MHz, 298 K, $CDCl_3$

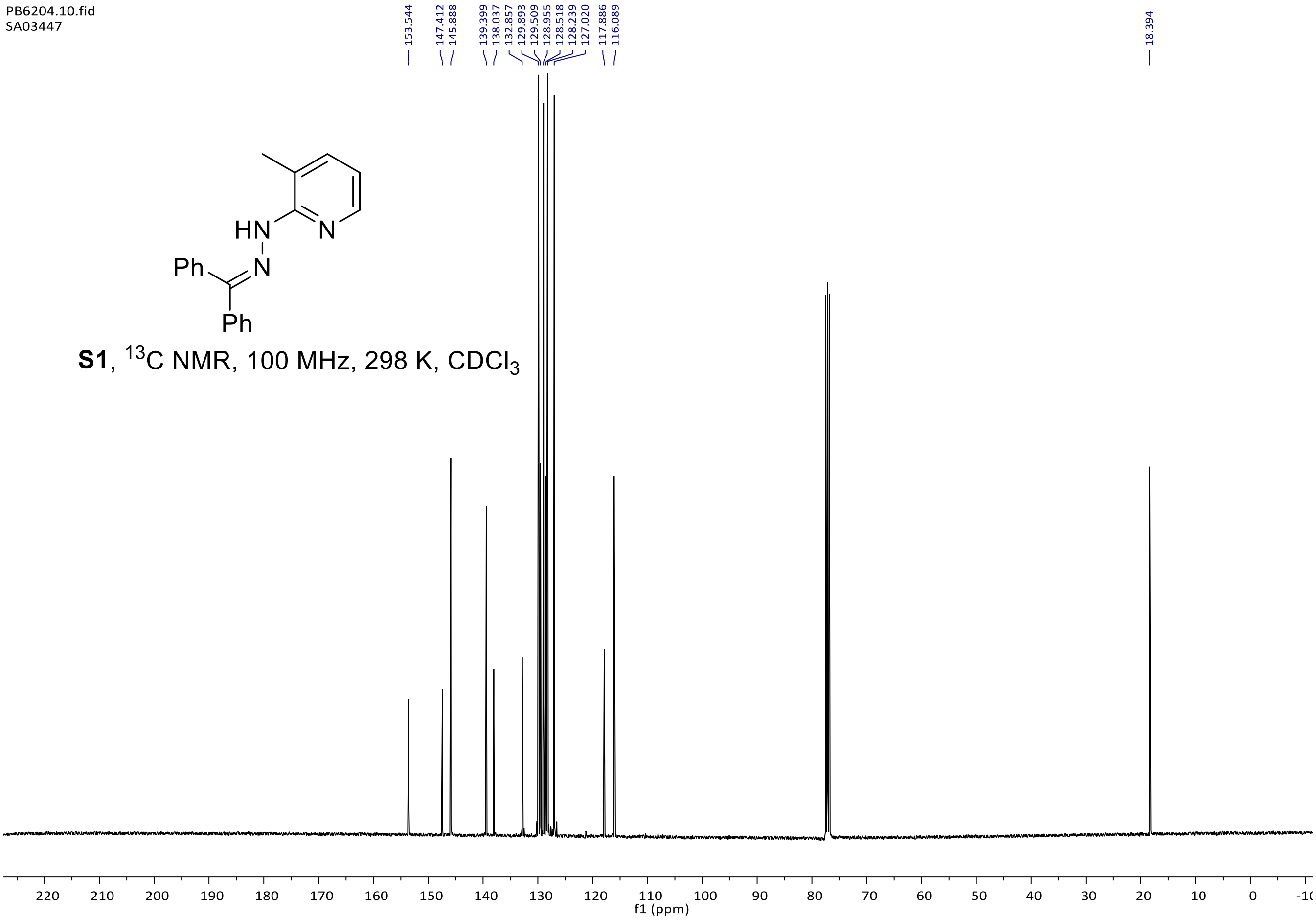

S1, ^{13}C NMR, 100 MHz, 298 K, $CDCl_3$

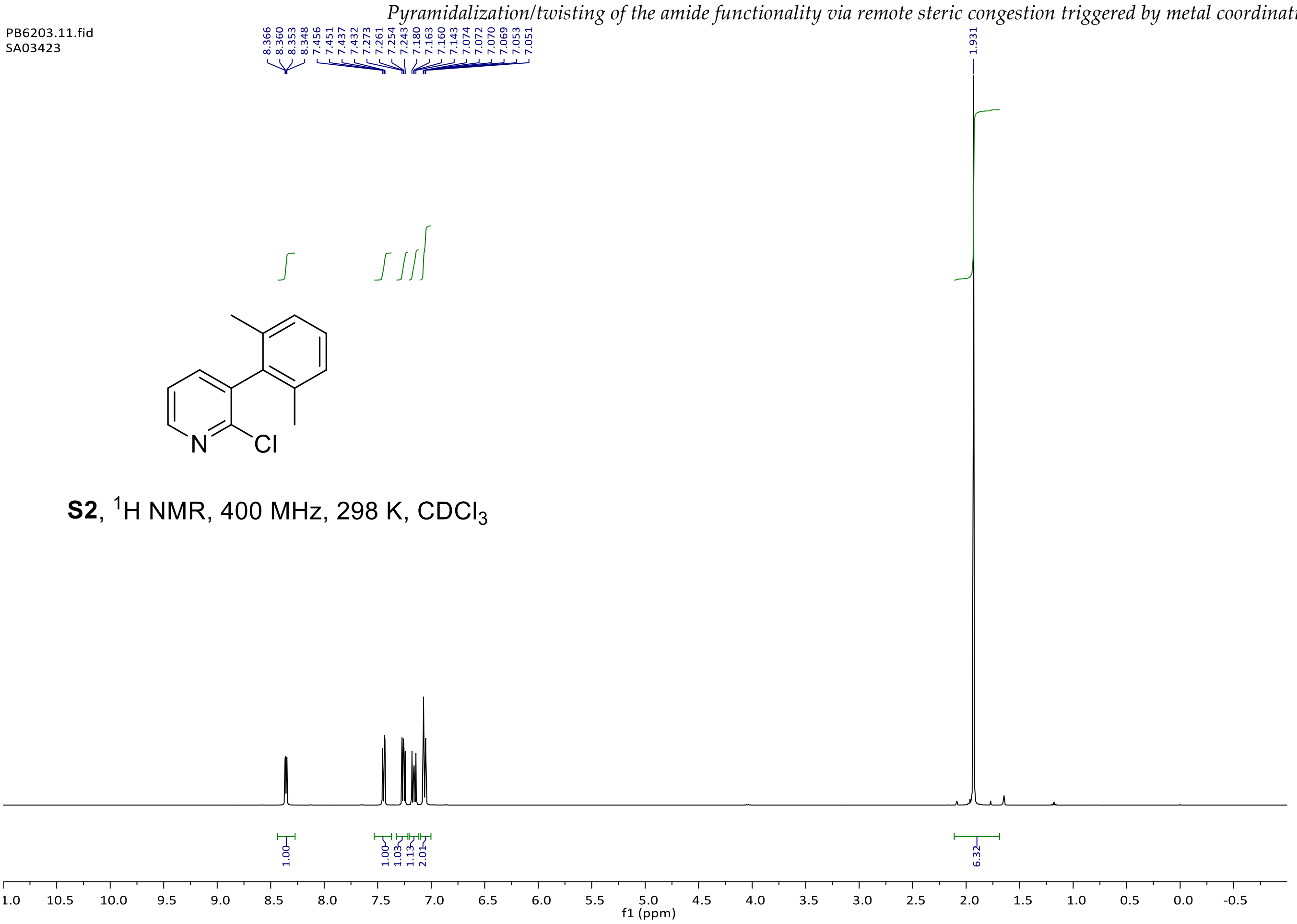
PB6203.11.fid
SA03423
8.366 8.360 8.353 8.348 7.456 7.451 7.437 7.432 7.273 7.261 7.254 7.243 7.180 7.163 7.160 7.143 7.074 7.072 7.070 7.069 7.053 7.051
1.931
N
Cl
S2, ^{1}H NMR, 400 MHz, 298 K, $CDCl_3$
1.00 1.00 1.03 1.13 2.01 6.32
f1 (ppm)

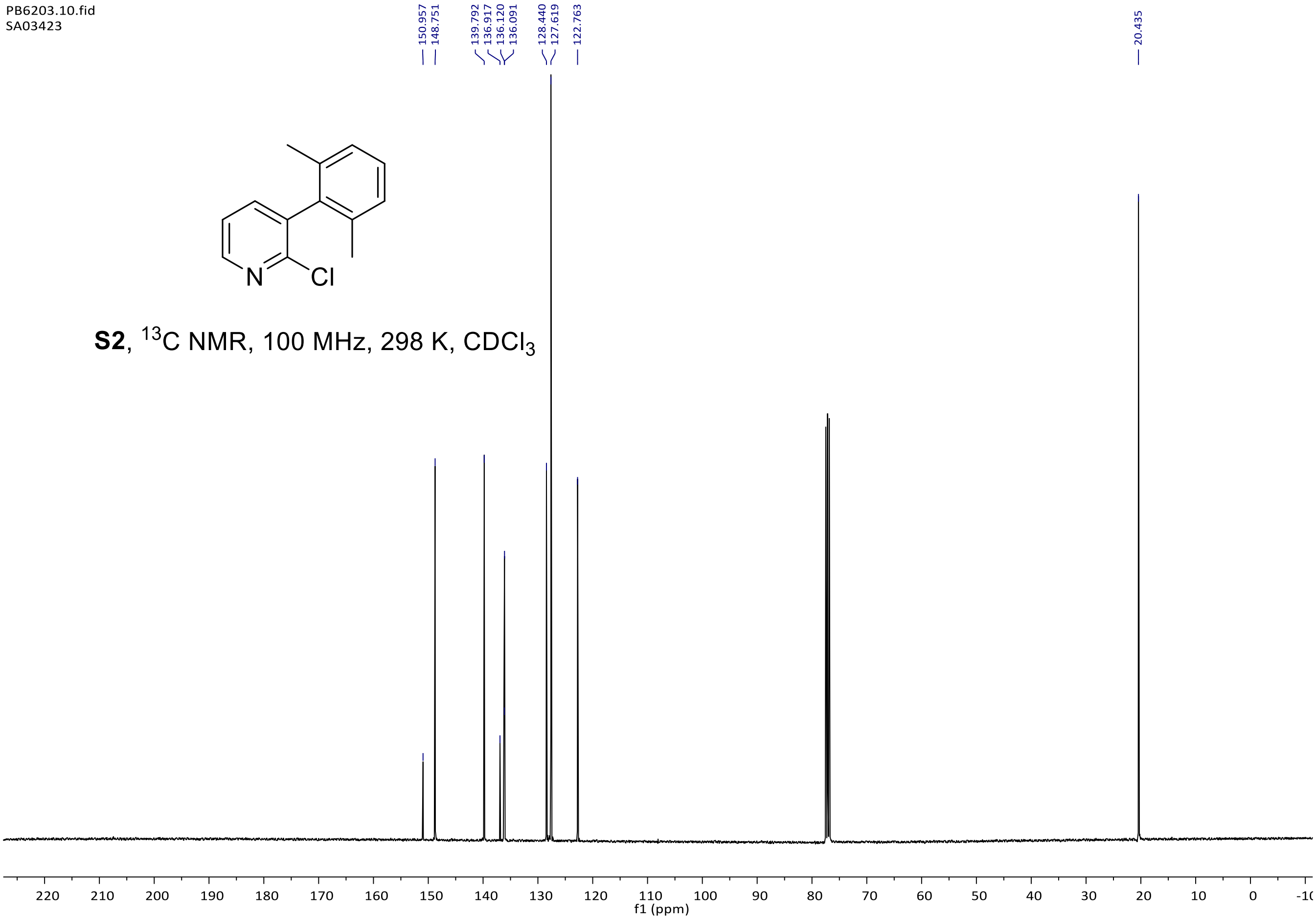
PB6203.10.fid
SA03423
150.957 148.751 139.792 136.917 136.120 136.091 128.440 127.619 122.763 20.435
N
Cl
S2, ^{13}C NMR, 100 MHz, 298 K, $CDCl_3$
f1 (ppm)

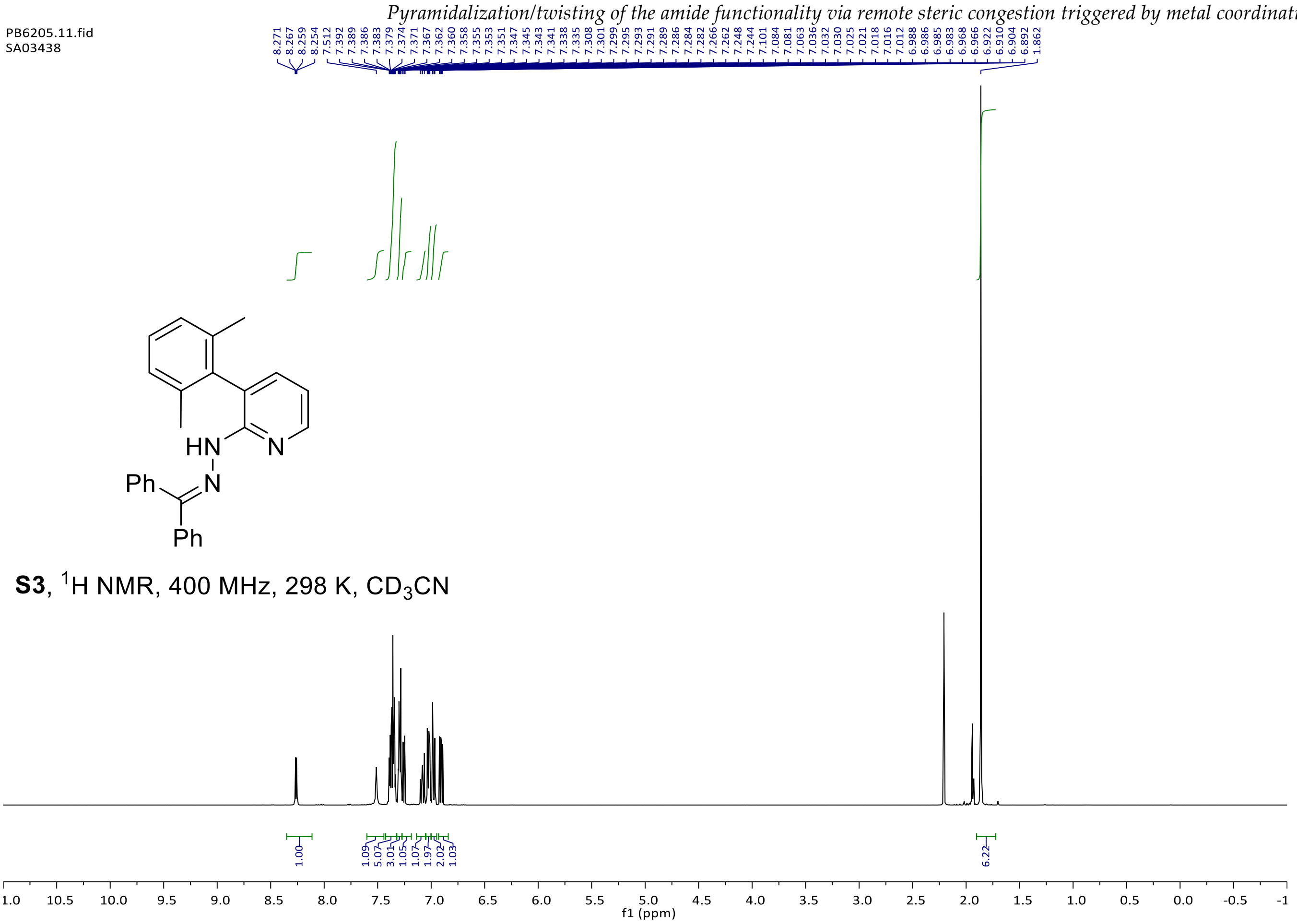

S3, ^{1}H NMR, 400 MHz, 298 K, CD_3CN

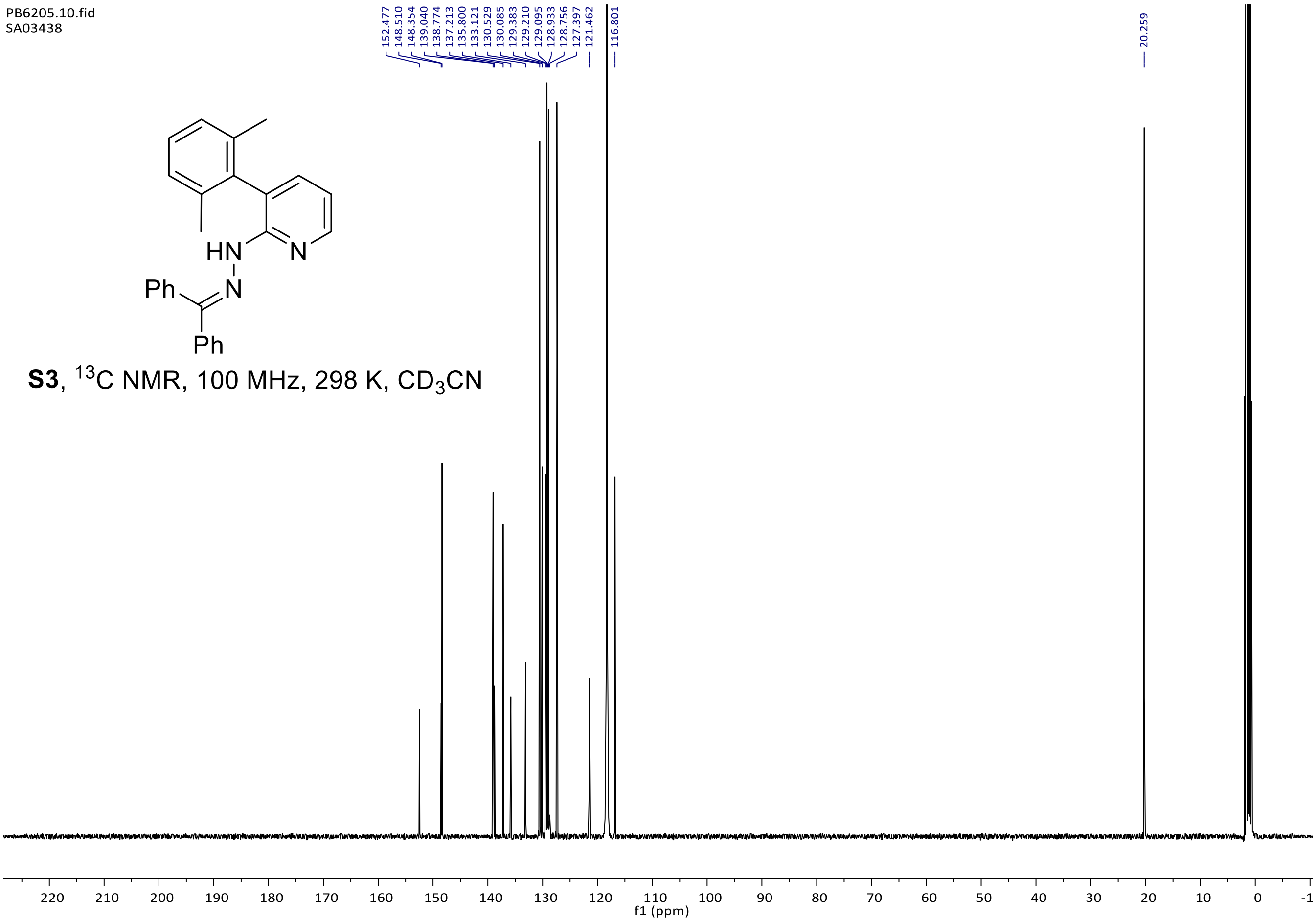

S3, ^{13}C NMR, 100 MHz, 298 K, CD_3CN

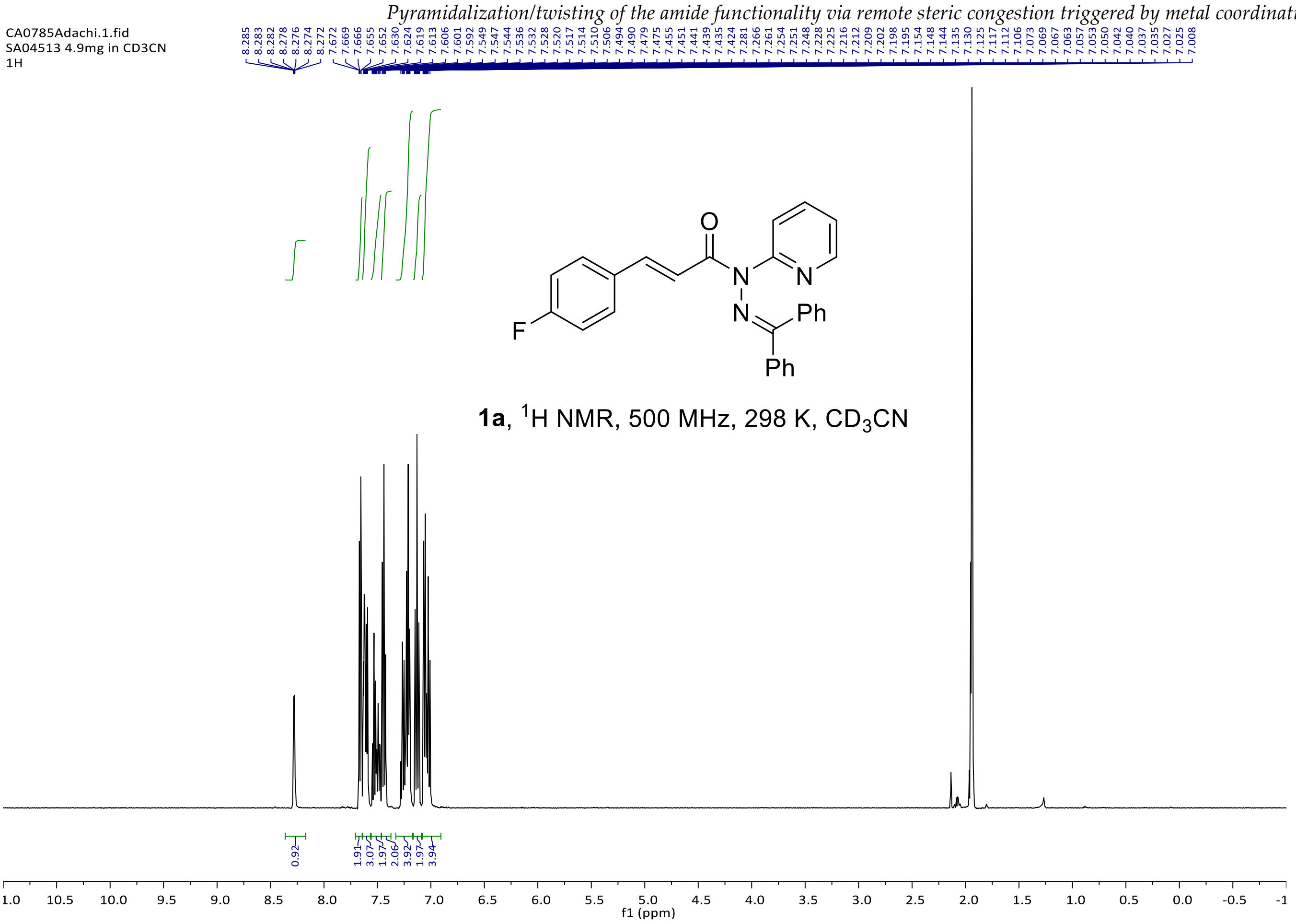
CA0785Adachi.1.fid
SA04513 4.9mg in CD3CN
1H
8.285 8.283 8.282 8.278 8.276 8.274 8.272 7.672 7.669 7.666 7.655 7.652 7.630 7.624 7.619 7.613 7.606 7.601 7.592 7.549 7.547 7.544 7.536 7.532 7.528 7.520 7.517 7.514 7.510 7.506 7.494 7.490 7.479 7.475 7.455 7.451 7.441 7.439 7.435 7.424 7.281 7.266 7.261 7.254 7.251 7.248 7.228 7.225 7.216 7.212 7.209 7.202 7.198 7.195 7.154 7.148 7.144 7.135 7.130 7.125 7.117 7.112 7.106 7.073 7.069 7.067 7.063 7.063 7.057 7.053 7.050 7.042 7.040 7.037 7.035 7.027 7.025 7.008
O
N
N
N
Ph
Ph
F
1a, ^{1}H NMR, 500 MHz, 298 K, CD_3CN
0.92
1.91 3.07 1.97 2.06 3.92 1.97 3.94
f1 (ppm)

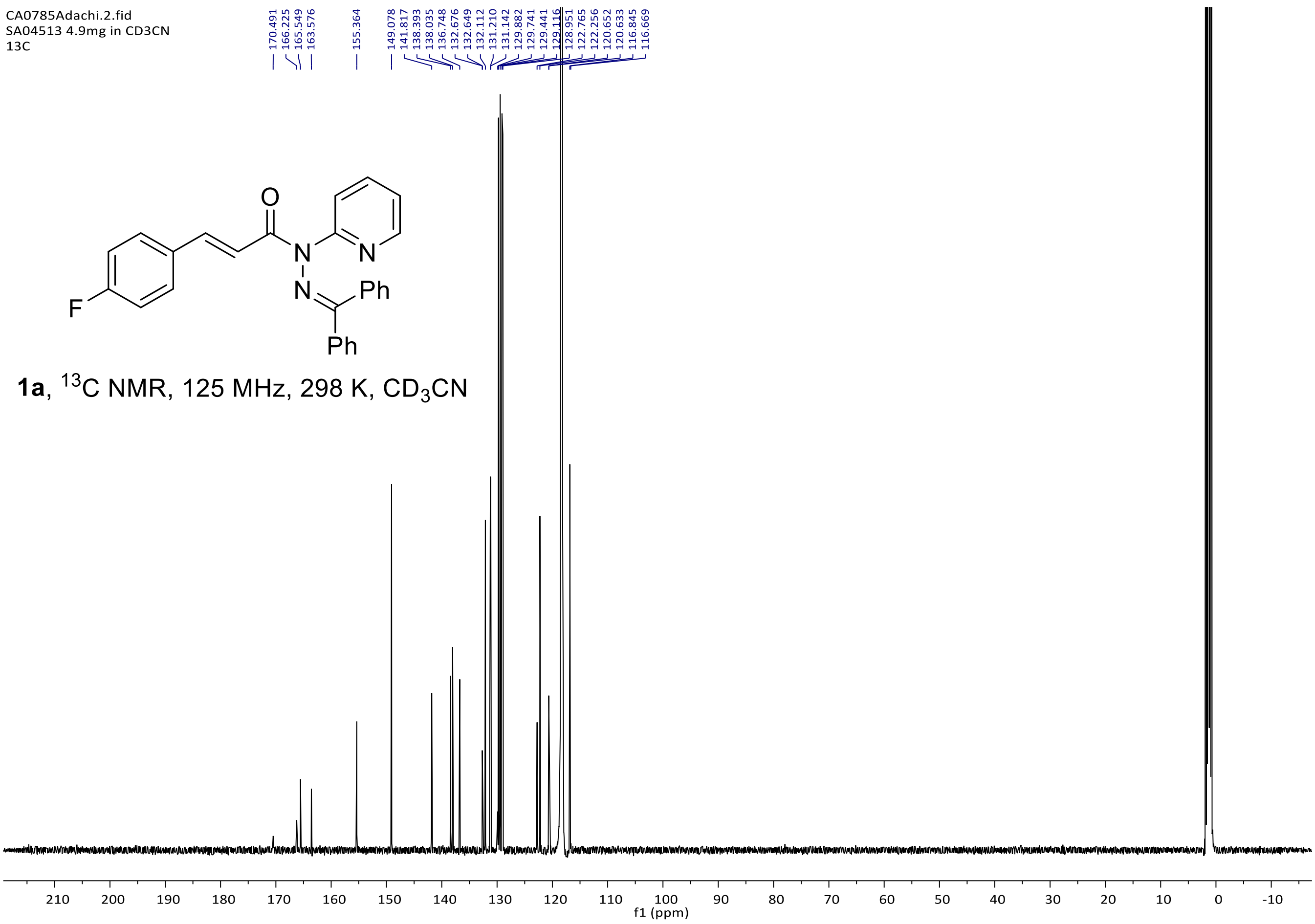
CA0785Adachi.2.fid
SA04513 4.9mg in CD3CN
13C
170.491 166.225 165.549 163.576 155.364 149.078 141.817 138.393 138.035 136.748 132.676 132.649 132.112 131.210 131.142 129.882 129.741 129.441 129.116 128.951 122.765 122.256 120.652 120.633 116.845 116.669
O
N
N
N
Ph
Ph
F
1a, ^{13}C NMR, 125 MHz, 298 K, CD_3CN
f1 (ppm)

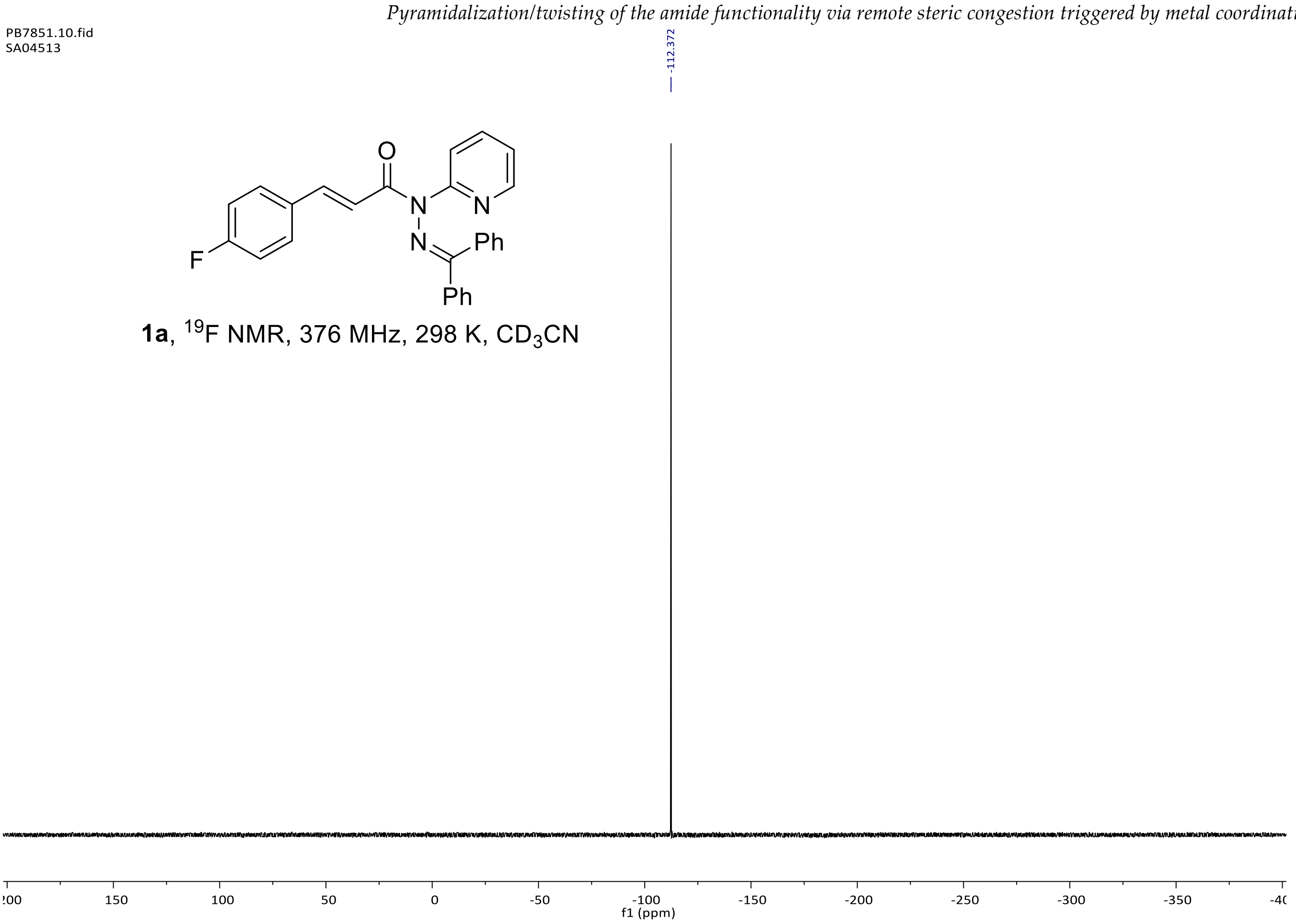
PB7851.10.fid
SA04513
-112.372
1a, ^{19}F NMR, 376 MHz, 298 K, CD_3CN
f1 (ppm)

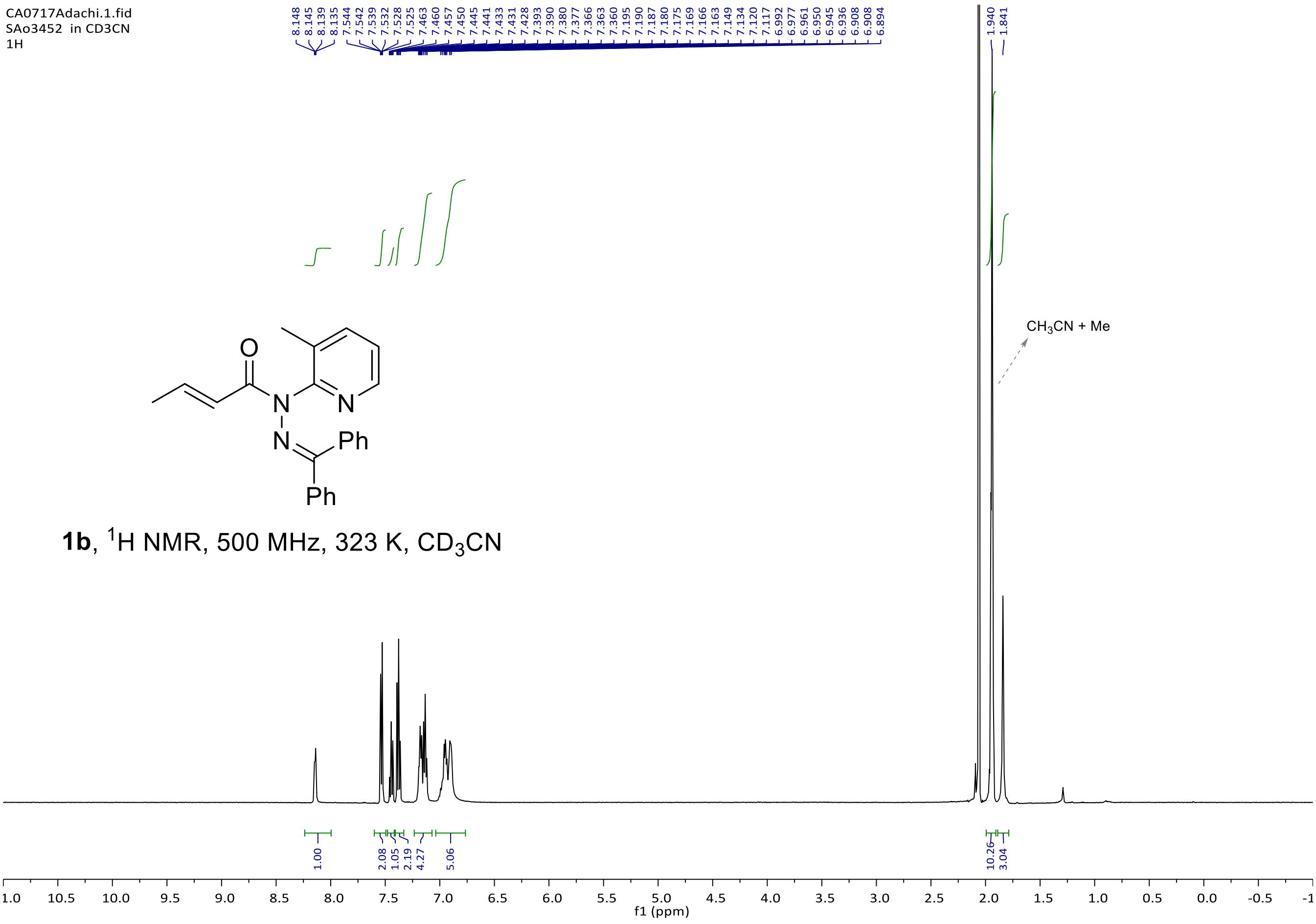
CA0717Adachi.1.fid
SAo3452 in CD3CN
1H
8.148 8.145 8.139 8.135 7.544 7.542 7.539 7.532 7.528 7.525 7.463 7.460 7.457 7.450 7.445 7.441 7.433 7.431 7.428 7.393 7.390 7.380 7.377 7.366 7.363 7.360 7.195 7.190 7.187 7.180 7.175 7.169 7.166 7.163 7.149 7.134 7.120 7.117 6.992 6.977 6.961 6.950 6.945 6.936 6.908 6.908 6.894
1.940 1.841
CH_3CN + Me
1b, ^{1}H NMR, 500 MHz, 323 K, CD_3CN
1.00 2.08 1.05 2.19 4.27 5.06 10.26 3.04
f1 (ppm)

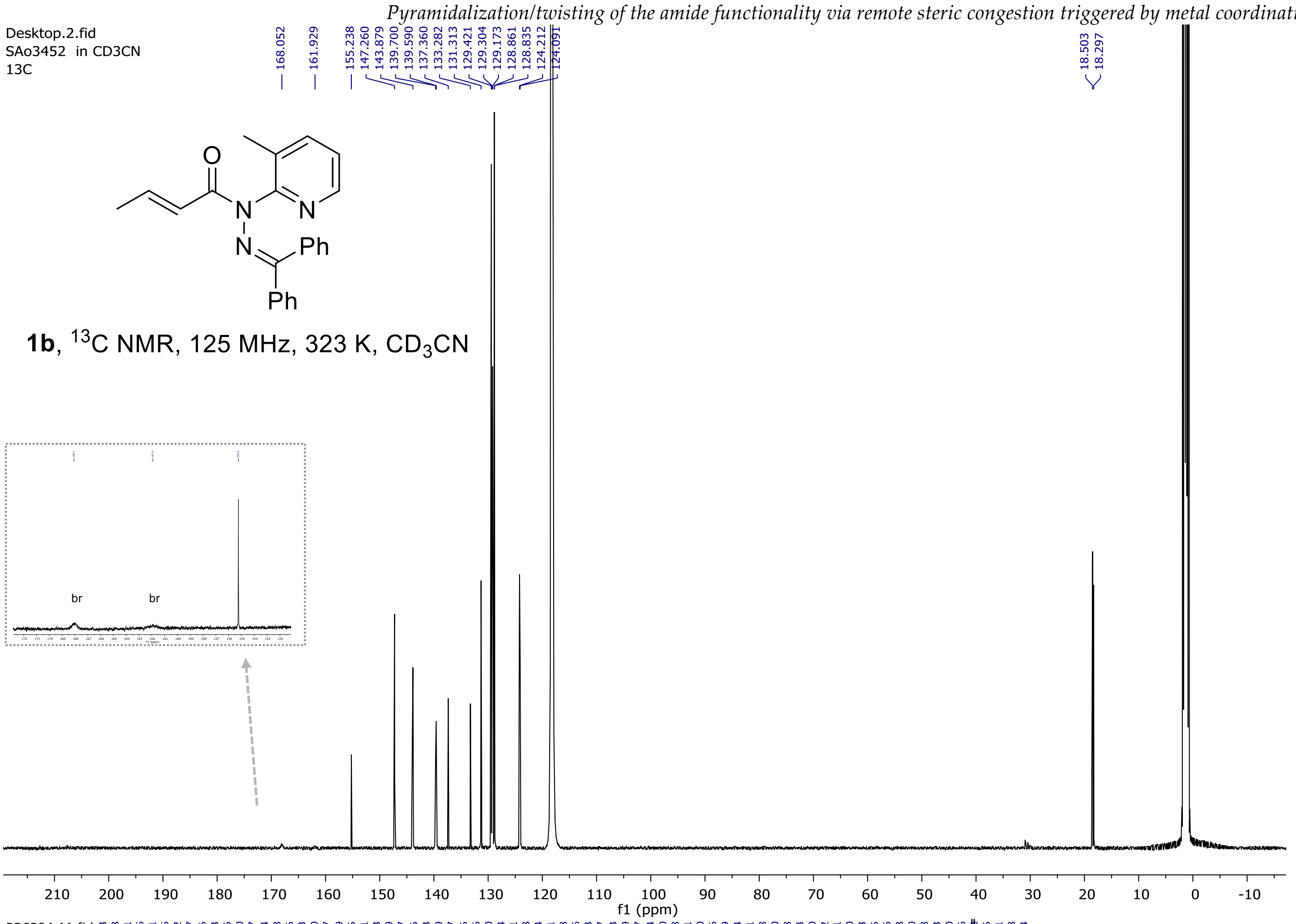

1b, ^{13}C NMR, 125 MHz, 323 K, CD_3CN

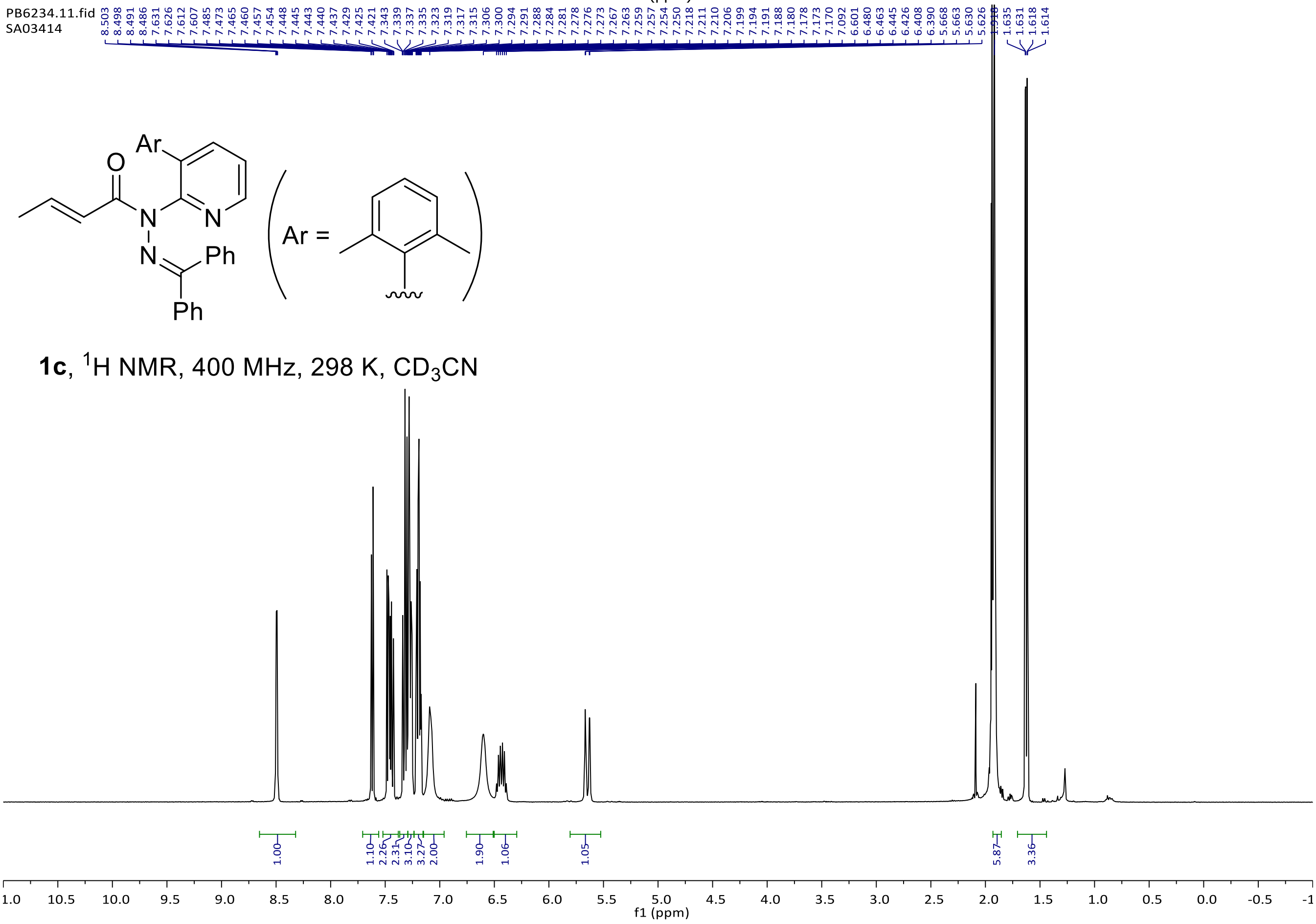

1c, ^{1}H NMR, 400 MHz, 298 K, CD_3CN

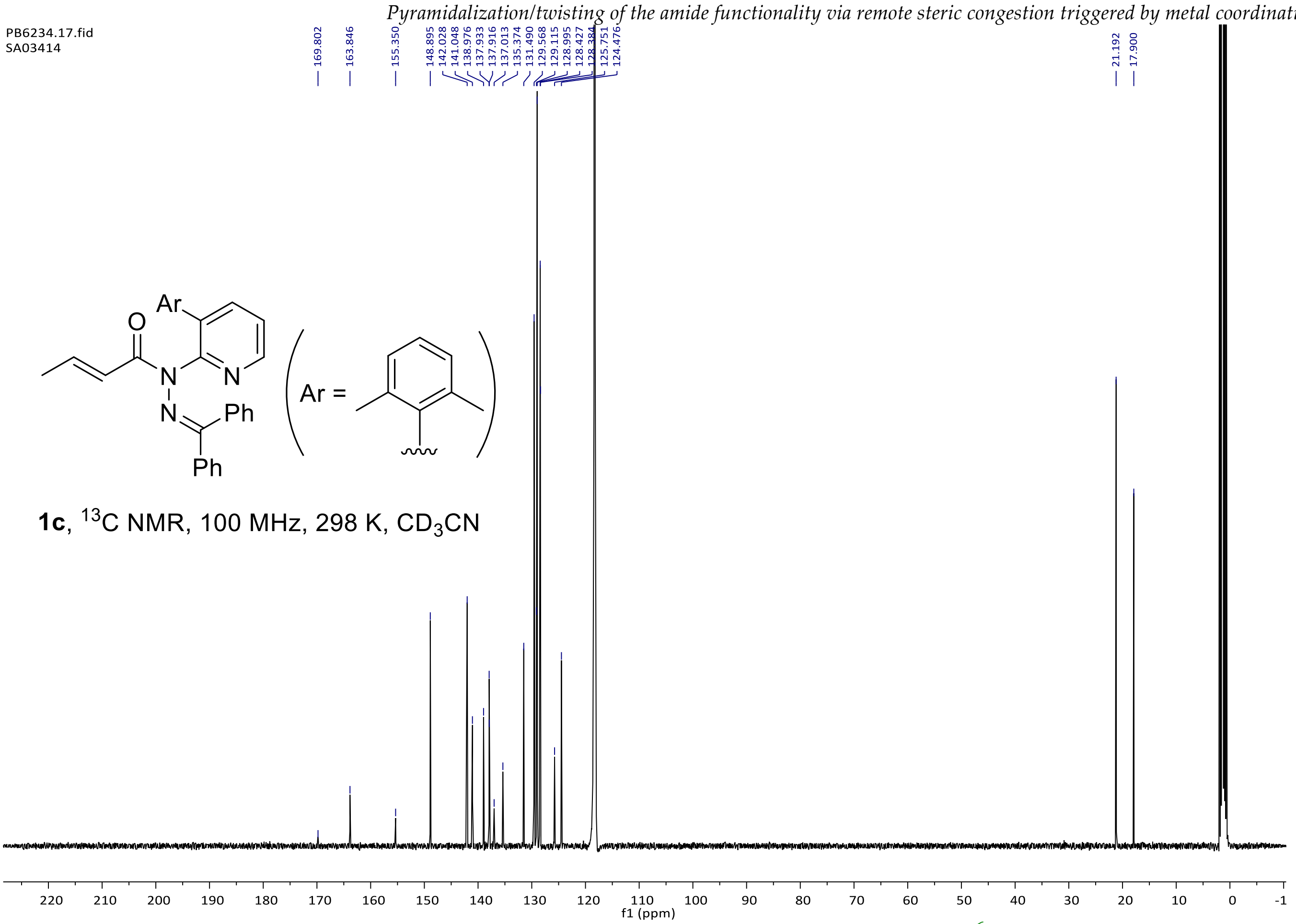
PB6234.17.fid
SA03414
169.802
163.846
155.350
148.895
142.028
141.048
138.976
137.933
137.916
137.013
135.374
131.490
129.568
129.115
128.995
128.427
125.751
124.476
21.192
17.900
Ar
O
N
N
N
Ph
Ph
Ar =
1c, 13C NMR, 100 MHz, 298 K, CD3CN
f1 (ppm)

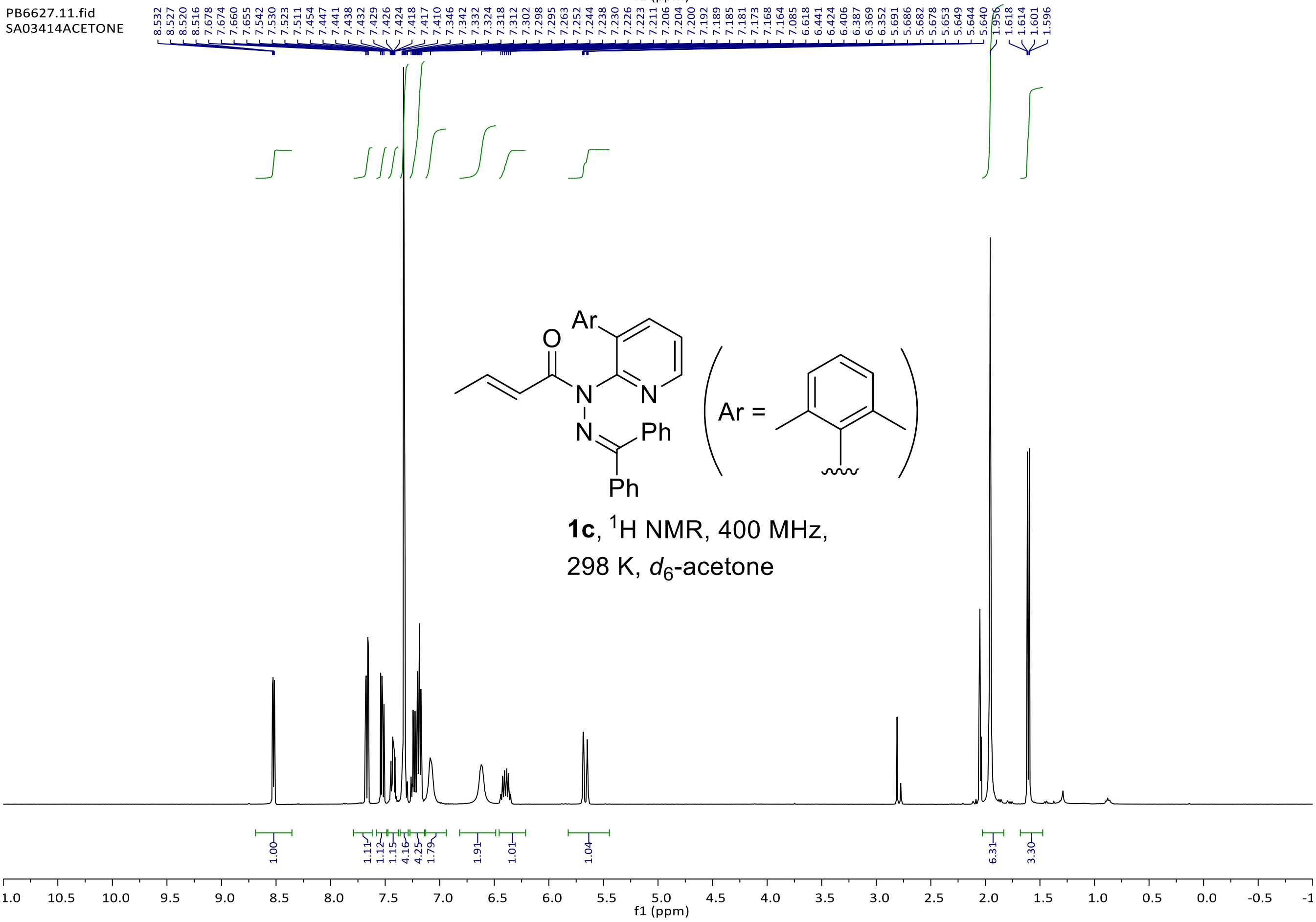
PB6627.11.fid
SA03414ACETONE
8.532
8.527
8.520
8.516
7.678
7.674
7.660
7.655
7.542
7.530
7.523
7.511
7.454
7.447
7.441
7.438
7.432
7.429
7.426
7.424
7.418
7.417
7.410
7.346
7.342
7.332
7.324
7.318
7.312
7.302
7.298
7.295
7.263
7.252
7.244
7.238
7.230
7.226
7.223
7.211
7.206
7.204
7.200
7.192
7.189
7.185
7.181
7.173
7.168
7.164
7.085
6.618
6.441
6.424
6.406
6.387
6.369
6.352
5.691
5.686
5.682
5.678
5.653
5.649
5.644
5.640
1.956
1.618
1.614
1.601
1.596
Ar
O
N
N
N
Ph
Ph
Ar =
1c, 1H NMR, 400 MHz, 298 K, d6-acetone
1.00
1.11
1.12
1.15
4.16
4.25
1.79
1.91
1.01
1.04
6.31
3.30
f1 (ppm)

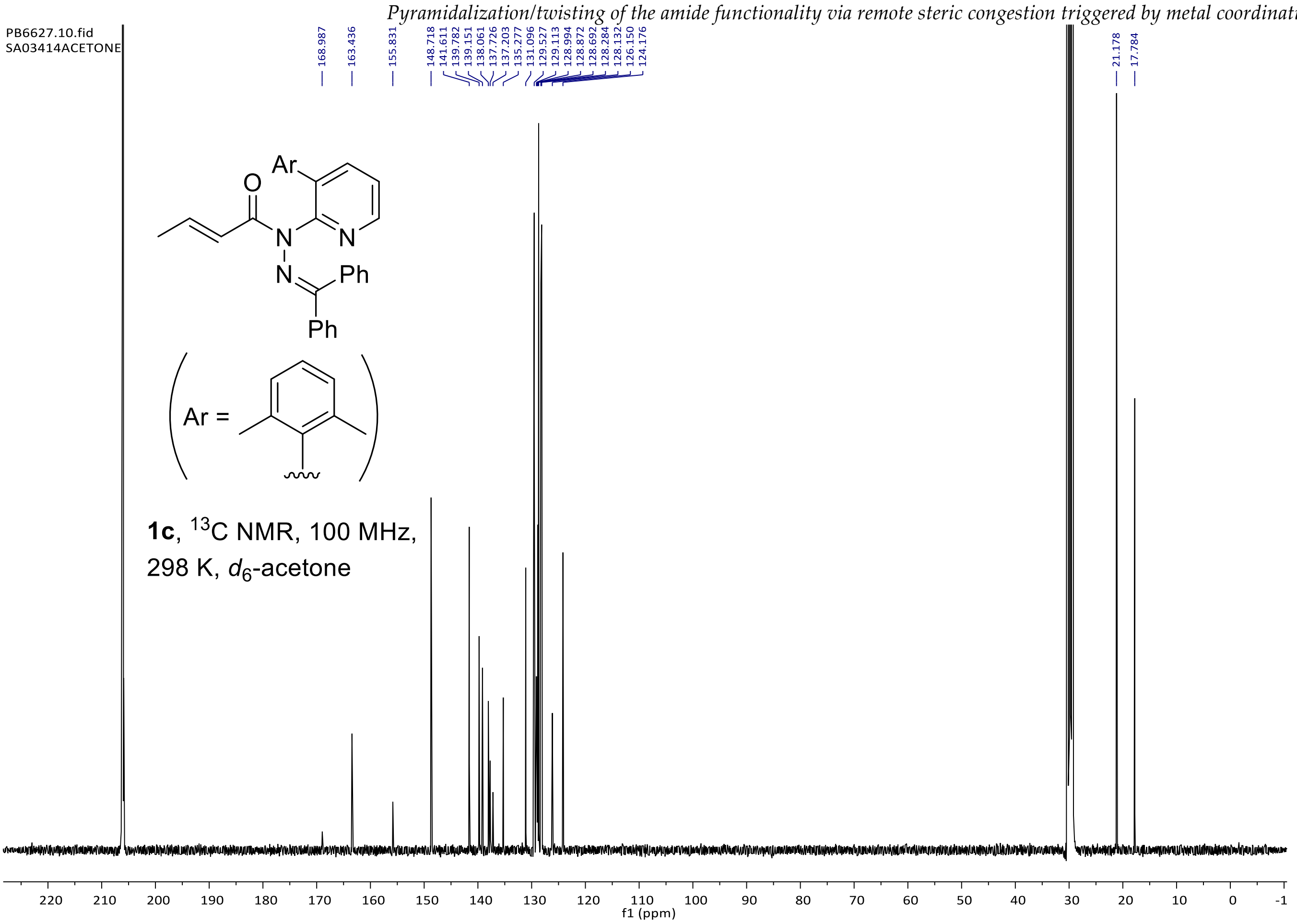
PB6627.10.fid
SA03414ACETONE
168.987
163.436
155.831
148.718
141.611
139.782
139.151
138.061
137.726
137.203
135.277
131.096
129.527
129.113
128.994
128.872
128.692
128.284
128.132
126.150
124.176
21.178
17.784
Ar
O
N
N
N
Ph
Ph
Ar =
1c, ¹³C NMR, 100 MHz,
298 K, d6-acetone
f1 (ppm)

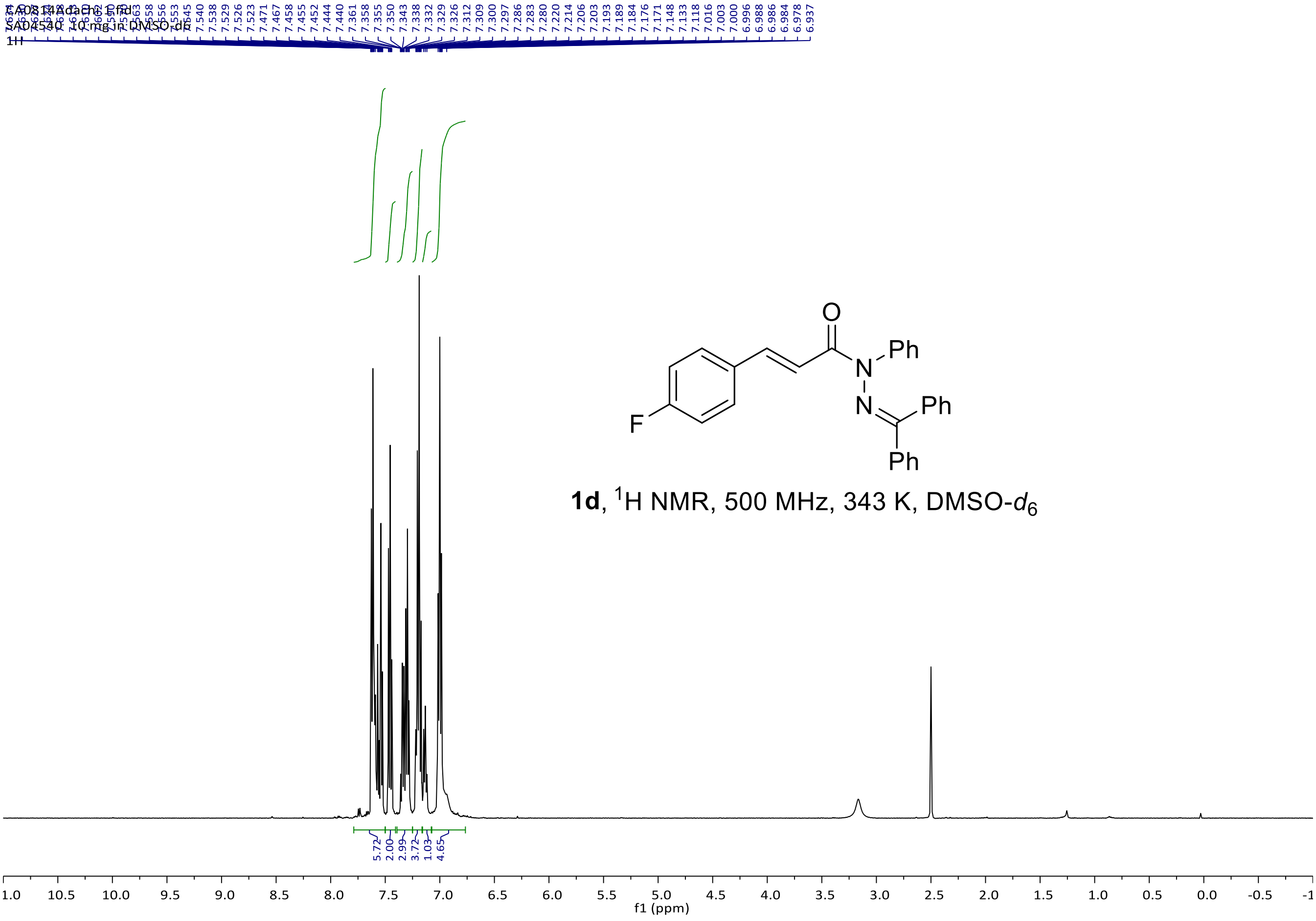
O
N
Ph
N
Ph
F
Ph
1d, ¹H NMR, 500 MHz, 343 K, DMSO-d6
5.72
2.00
2.99
3.72
1.03
4.65
f1 (ppm)

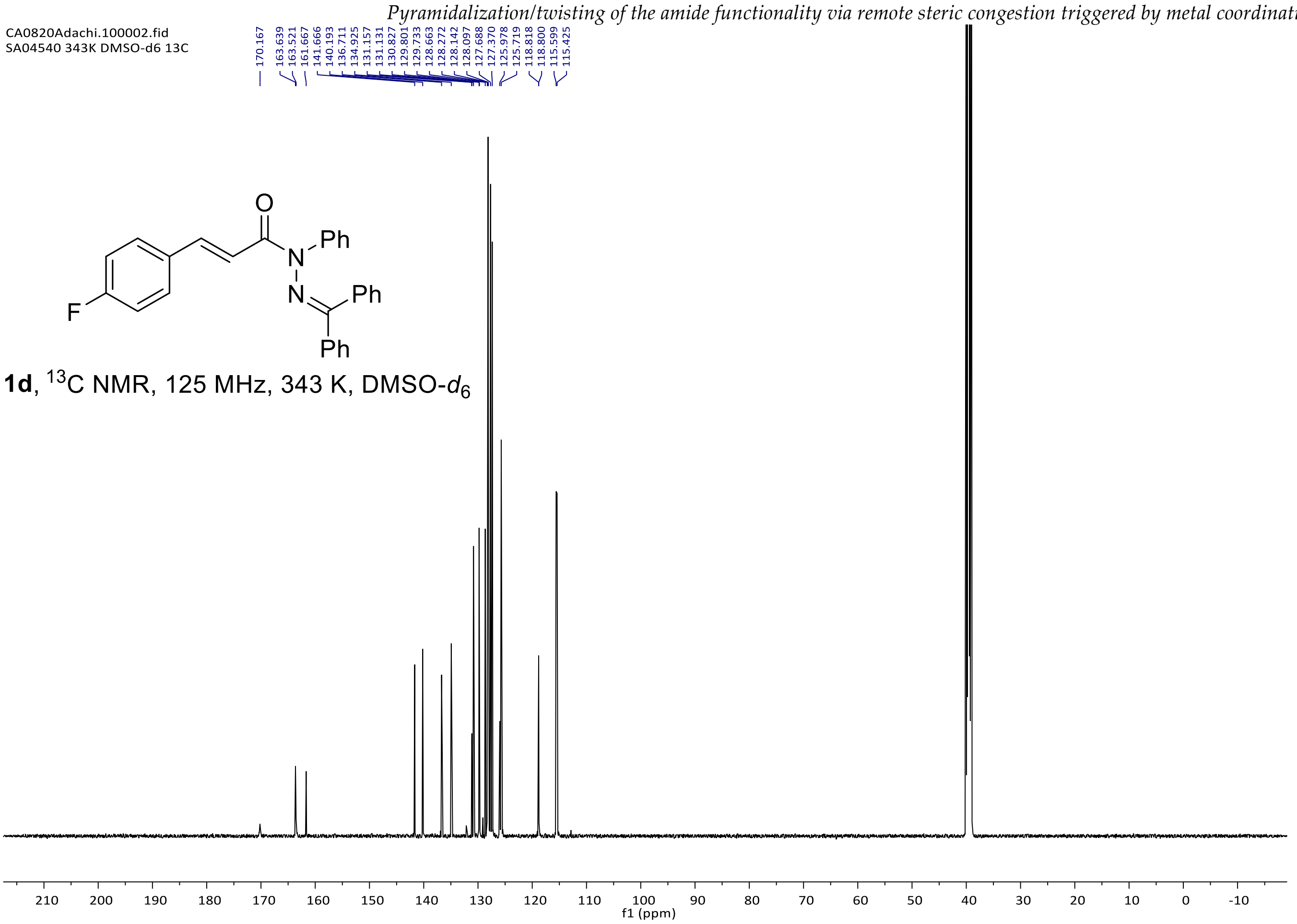

1d, ^{13}C NMR, 125 MHz, 343 K, DMSO-d_6

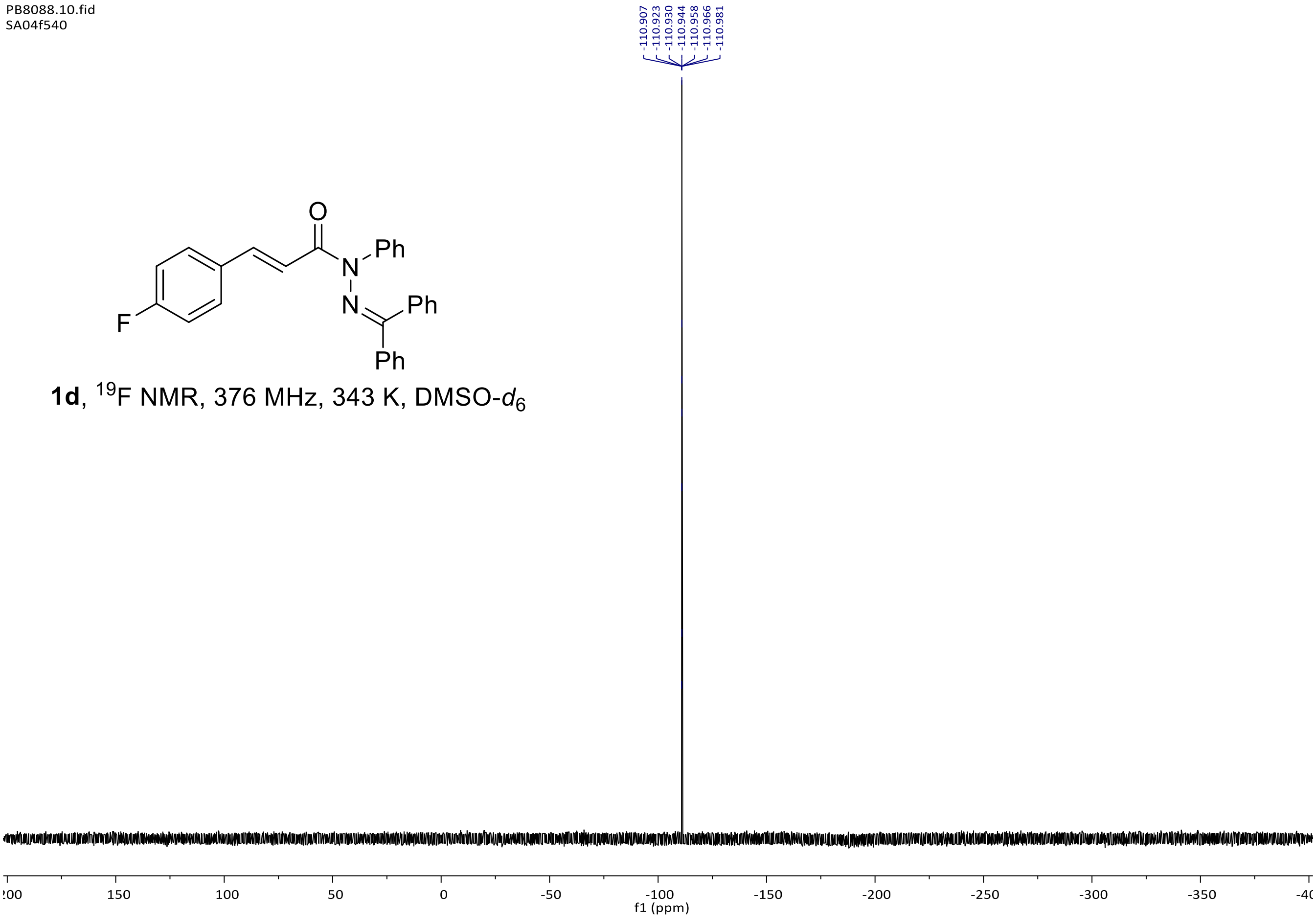

1d, ^{19}F NMR, 376 MHz, 343 K, DMSO-d_6

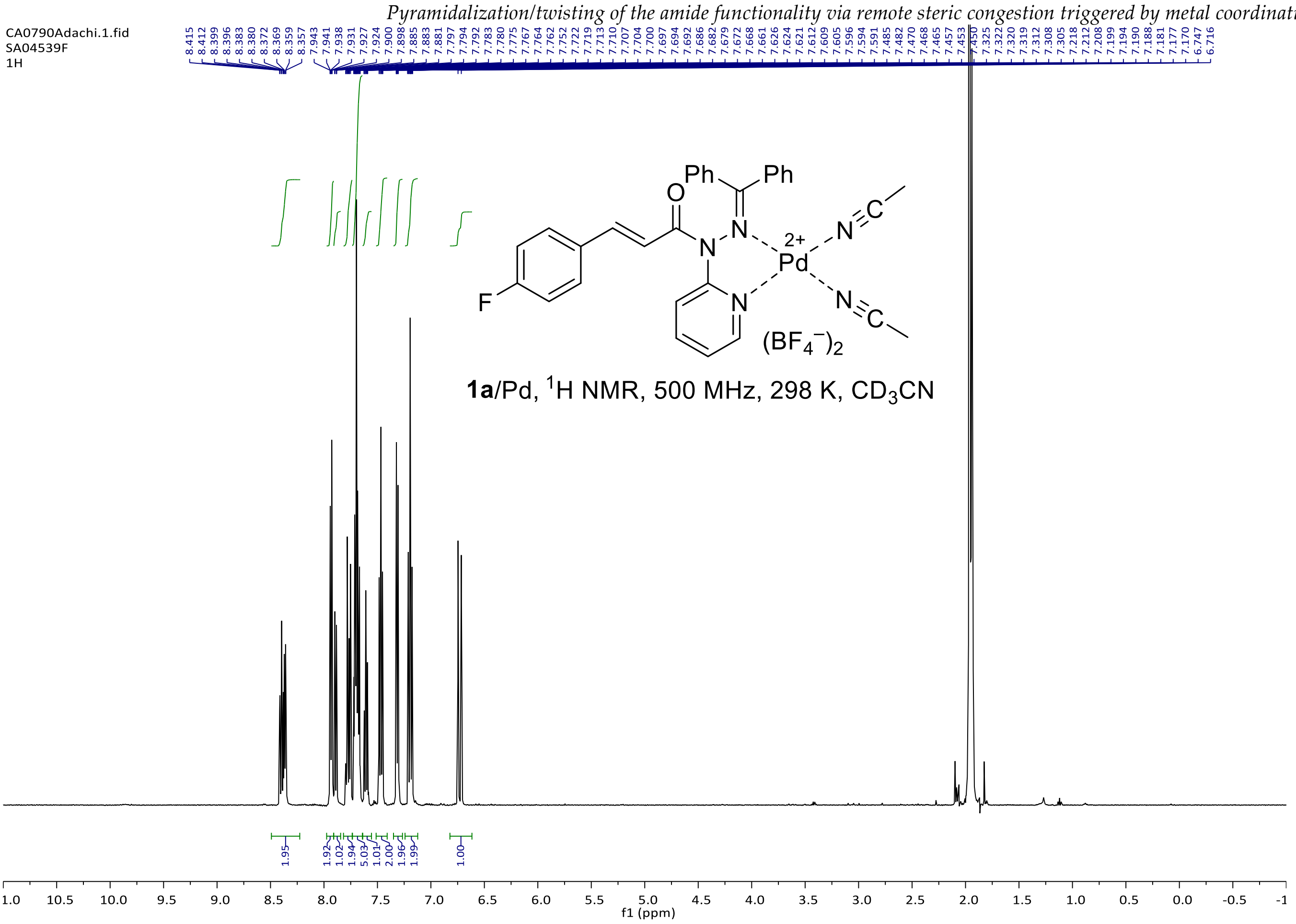
CA0790Adachi.1.fid
SA04539F
1H
1a/Pd, ^{1}H NMR, 500 MHz, 298 K, CD_3CN
Ph
Ph
O
N
N
2+
Pd
N
N
C
C
F
$(BF_4^-)_2$
1.95
1.92
1.02
1.94
5.03
1.01
2.00
1.96
1.99
1.00
f1 (ppm)

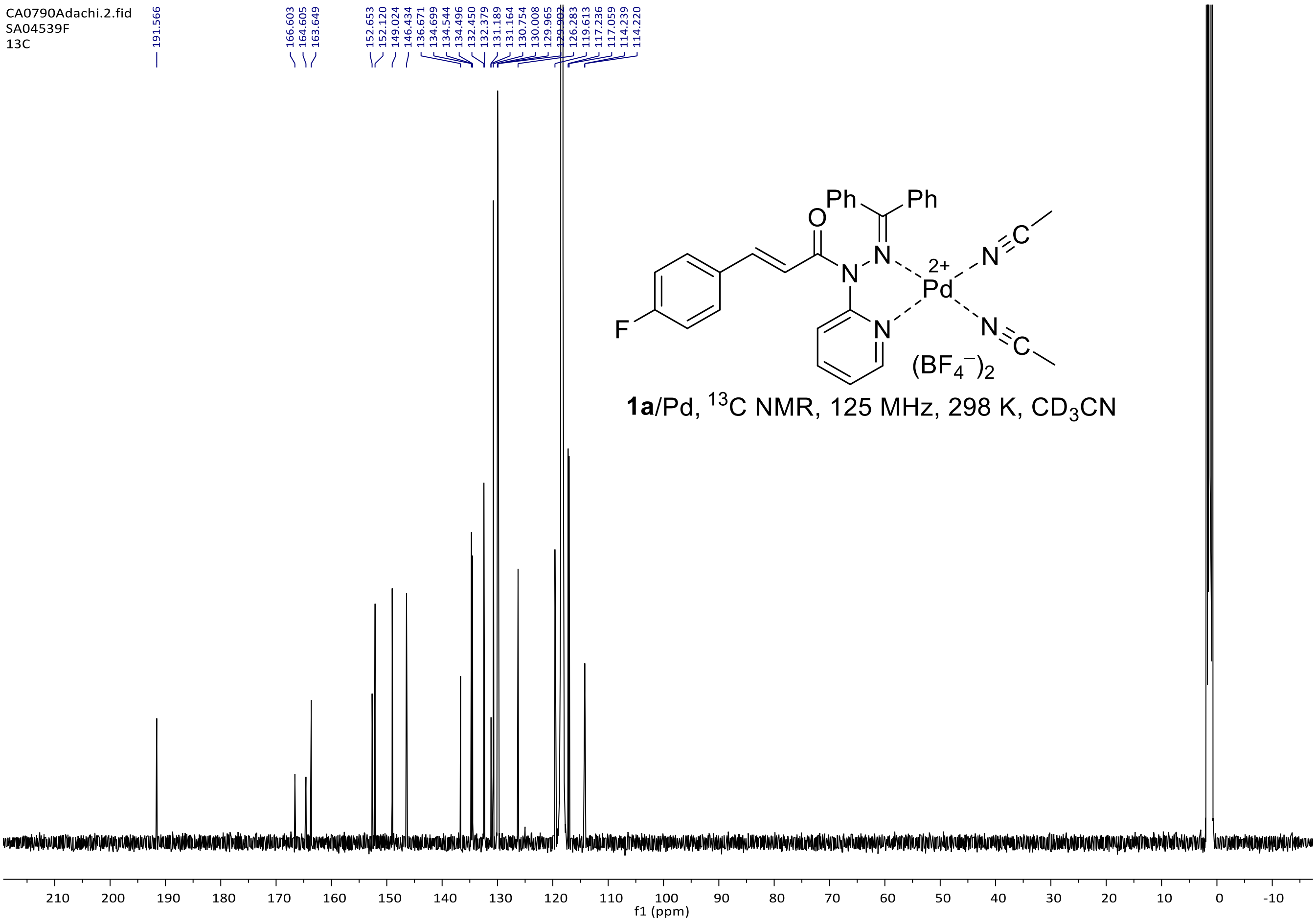
CA0790Adachi.2.fid
SA04539F
13C
191.566
166.603
164.605
163.649
152.653
152.120
149.024
146.434
136.671
134.699
134.544
134.496
132.450
132.379
131.189
131.164
130.754
130.008
129.965
126.283
119.613
117.236
117.059
114.239
114.220
1a/Pd, ^{13}C NMR, 125 MHz, 298 K, CD_3CN
Ph
Ph
O
N
N
2+
Pd
N
N
C
C
F
$(BF_4^-)_2$
f1 (ppm)

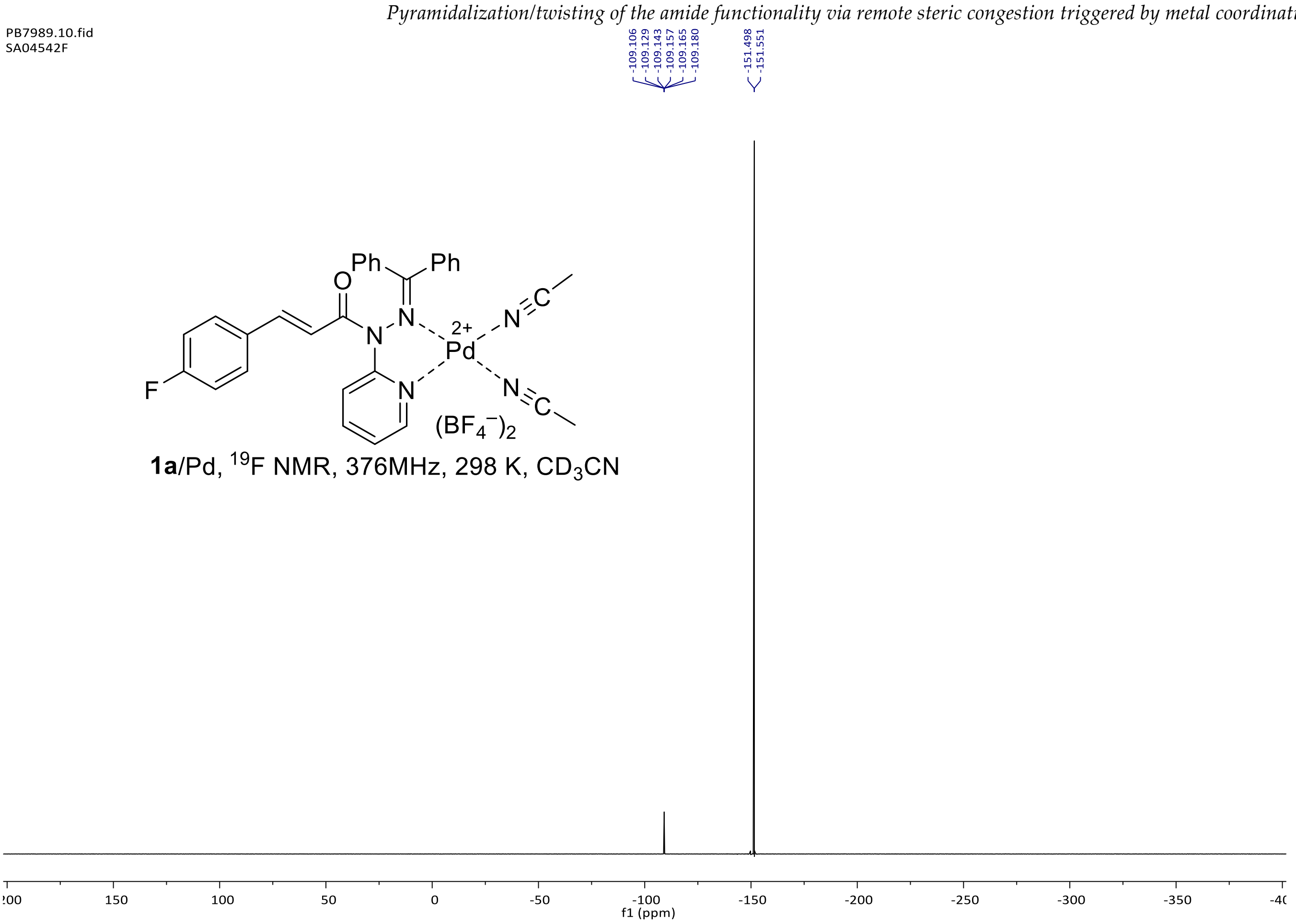
PB7989.10.fid
SA04542F
-109.106
-109.129
-109.143
-109.157
-109.165
-109.180
-151.498
-151.551
1a/Pd, 19F NMR, 376MHz, 298 K, CD3CN
f1 (ppm)

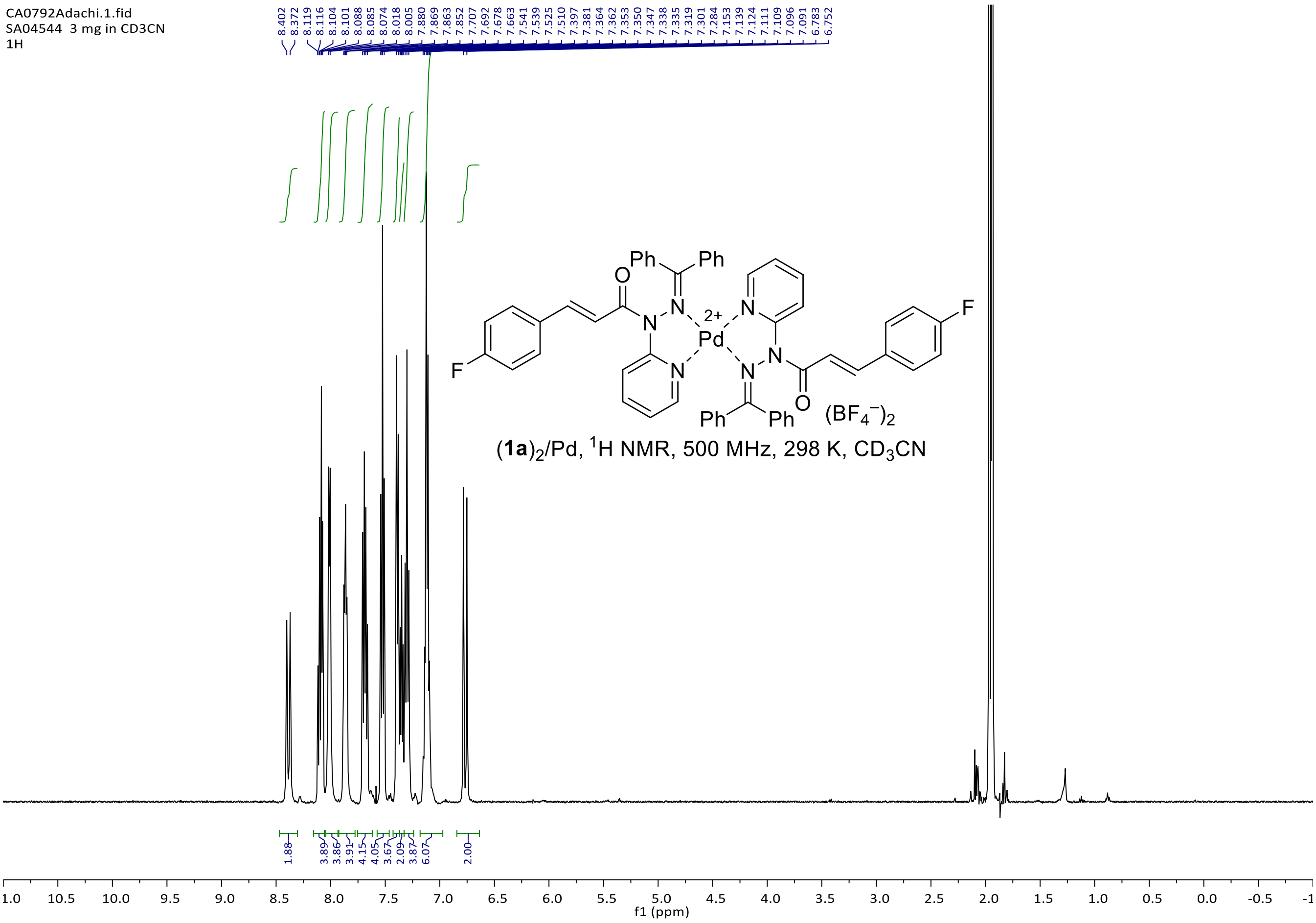
CA0792Adachi.1.fid
SA04544 3 mg in CD3CN
1H
(1a)2/Pd, 1H NMR, 500 MHz, 298 K, CD3CN
f1 (ppm)

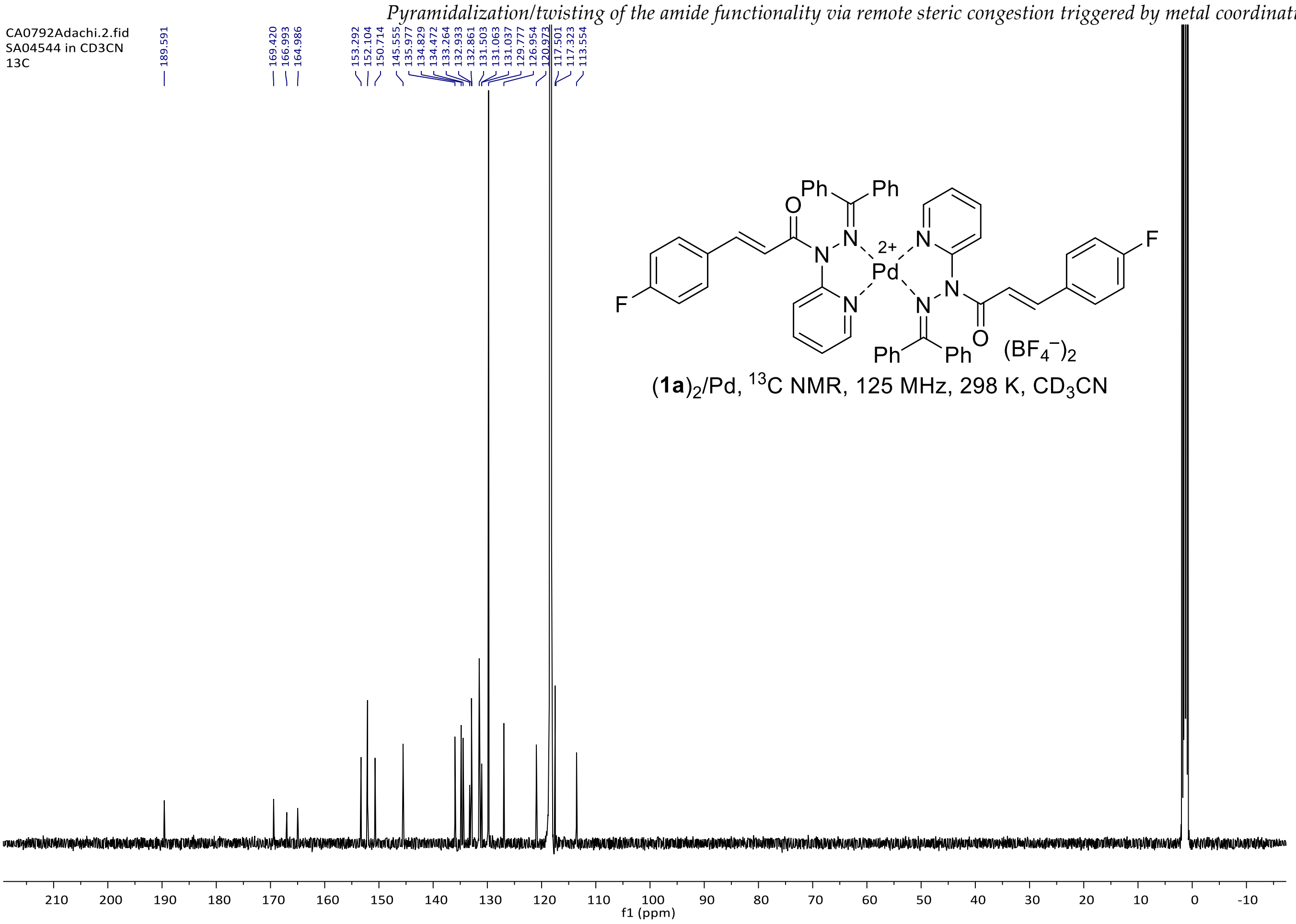
CA0792Adachi.2.fid
SA04544 in CD3CN
13C
189.591
169.420
166.993
164.986
153.292
152.104
150.714
145.555
135.977
134.829
134.472
133.264
132.933
132.861
131.503
131.063
131.037
129.777
126.954
120.973
117.501
117.323
113.554
(1a)2/Pd, 13C NMR, 125 MHz, 298 K, CD3CN
f1 (ppm)

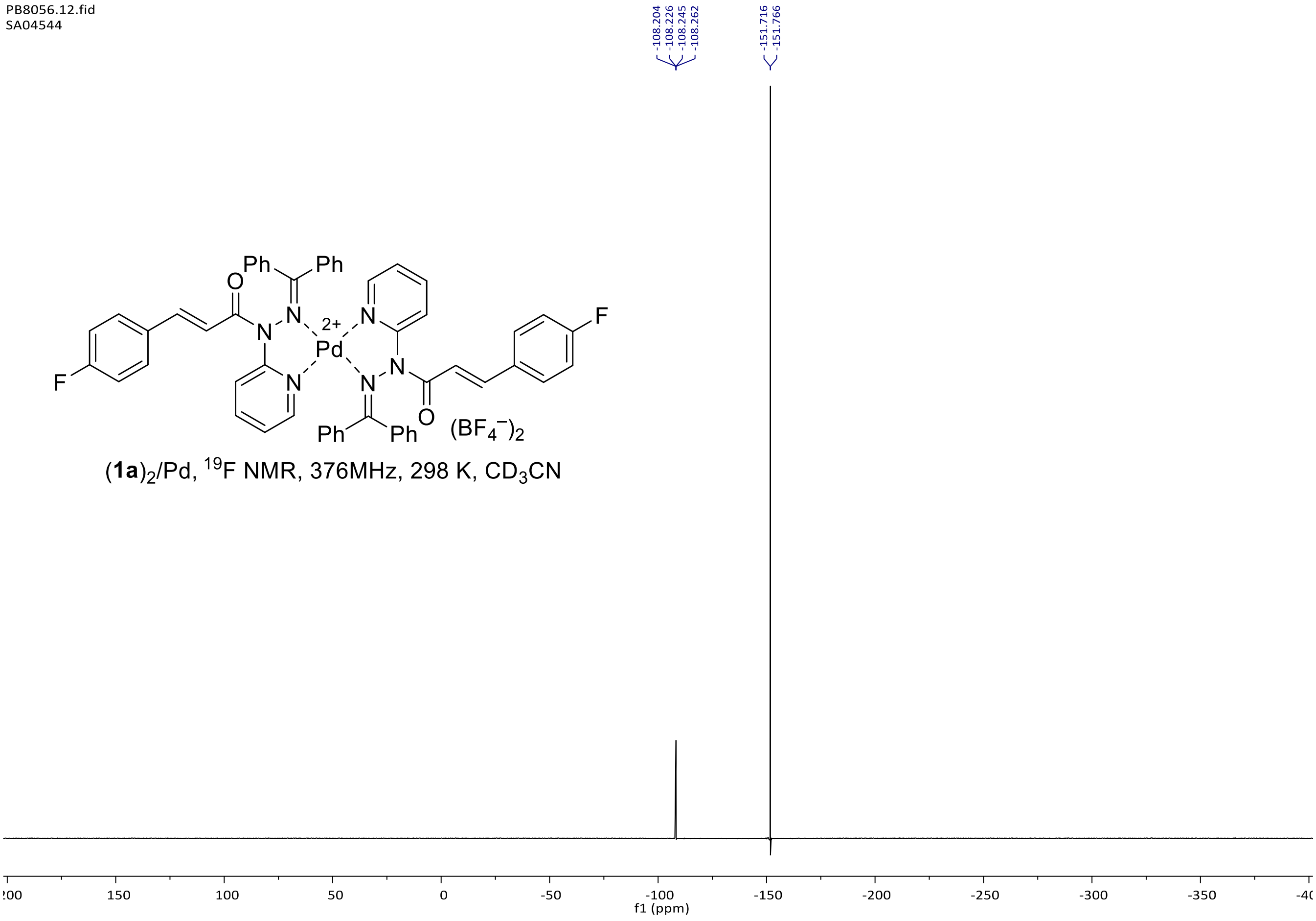
PB8056.12.fid
SA04544
-108.204
-108.226
-108.245
-108.262
-151.716
-151.766
(1a)2/Pd, 19F NMR, 376MHz, 298 K, CD3CN
f1 (ppm)

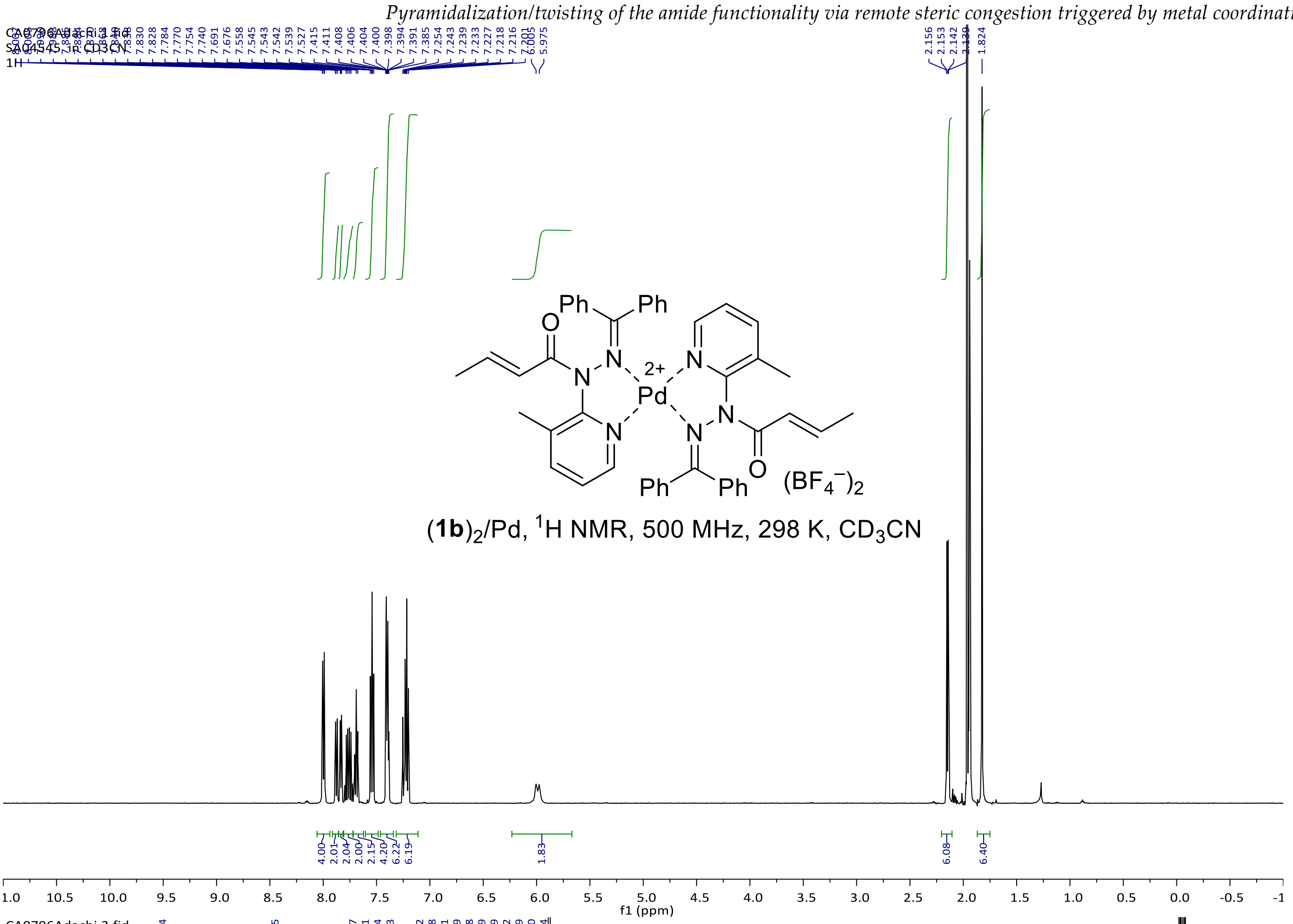
(1b)2/Pd, 1H NMR, 500 MHz, 298 K, CD3CN
(BF4−)2
f1 (ppm)

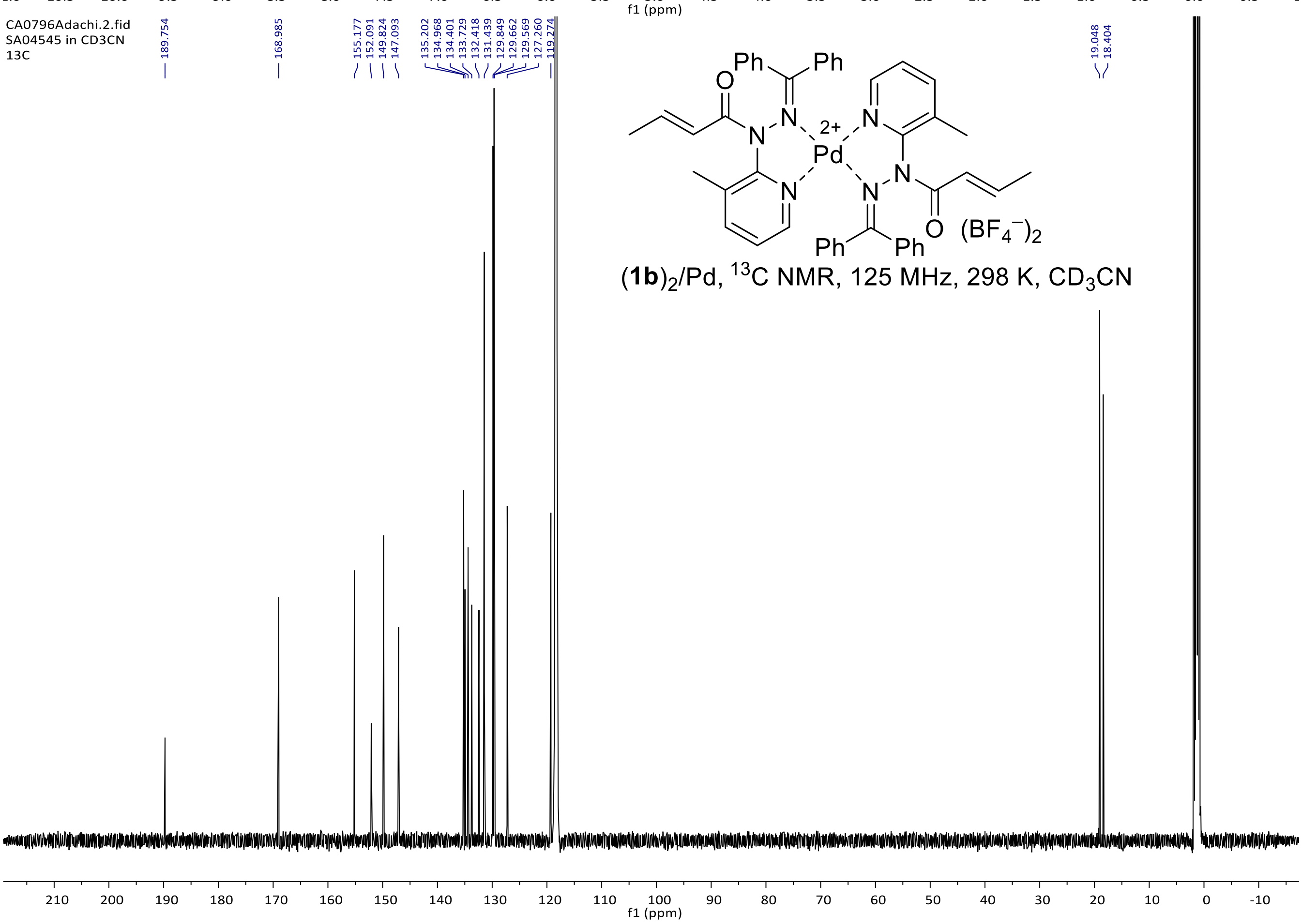
CA0796Adachi.2.fid
SA04545 in CD3CN
13C
189.754
168.985
155.177
152.091
149.824
147.093
135.202
134.968
134.401
133.729
132.418
131.439
129.849
129.662
129.569
127.260
119.274
19.048
18.404
(1b)2/Pd, 13C NMR, 125 MHz, 298 K, CD3CN
(BF4−)2
f1 (ppm)

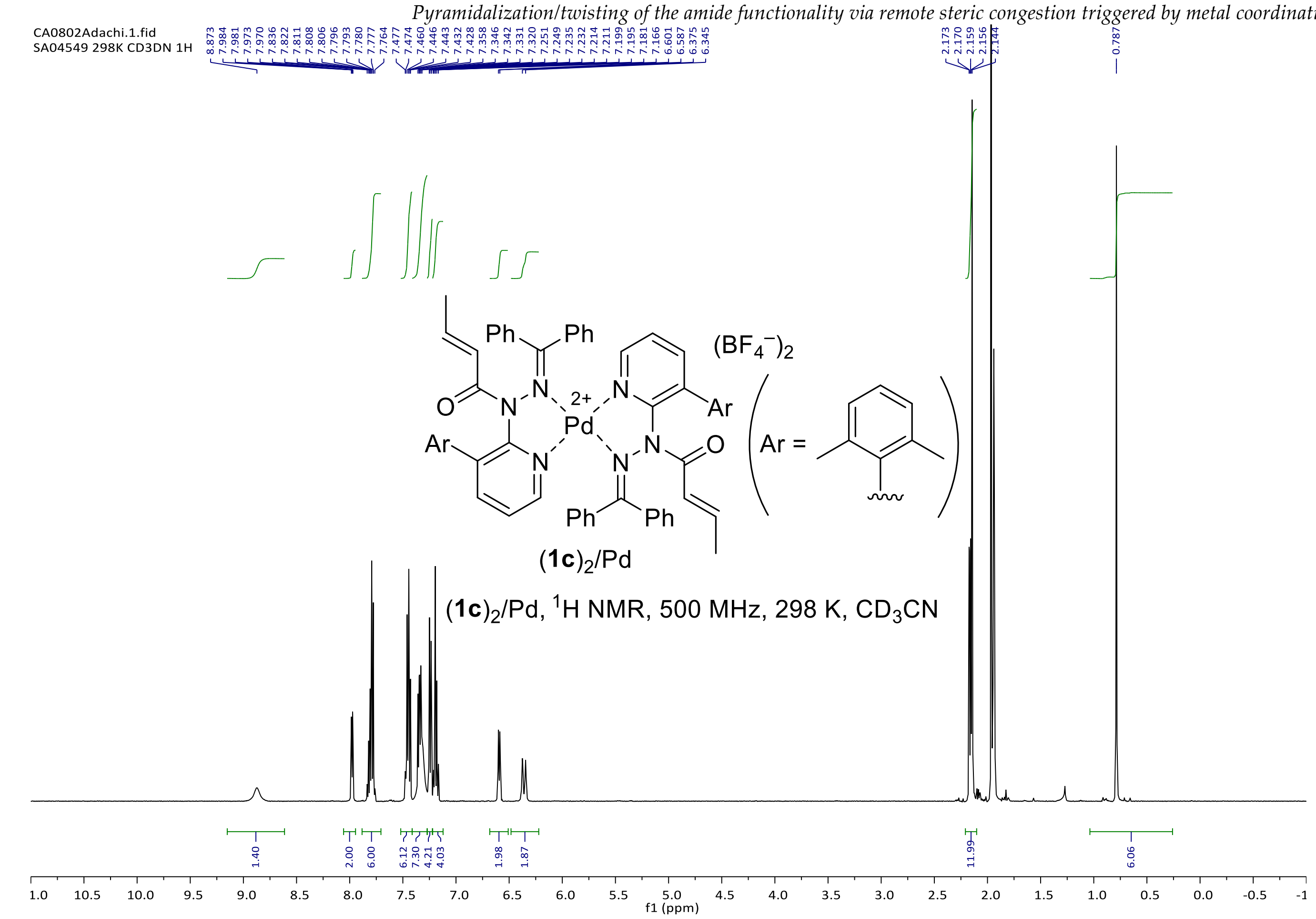
CA0802Adachi.1.fid
SA04549 298K CD3DN 1H
8.873 7.984 7.981 7.973 7.970 7.836 7.822 7.811 7.808 7.806 7.796 7.793 7.780 7.777 7.764 7.477 7.474 7.460 7.446 7.443 7.432 7.428 7.358 7.346 7.342 7.331 7.320 7.251 7.249 7.235 7.232 7.214 7.211 7.199 7.195 7.181 7.166 6.601 6.587 6.375 6.345
2.173 2.170 2.159 2.156 2.144
0.787
Ph
Ph
(BF4⁻)2
2+
O
N
N
Pd
N
Ar
Ar
N
N
N
O
Ar =
Ph
Ph
(1c)2/Pd
(1c)2/Pd, 1H NMR, 500 MHz, 298 K, CD3CN
1.40
2.00
6.00
6.12
7.30
4.21
4.03
1.98
1.87
11.99
6.06
f1 (ppm)

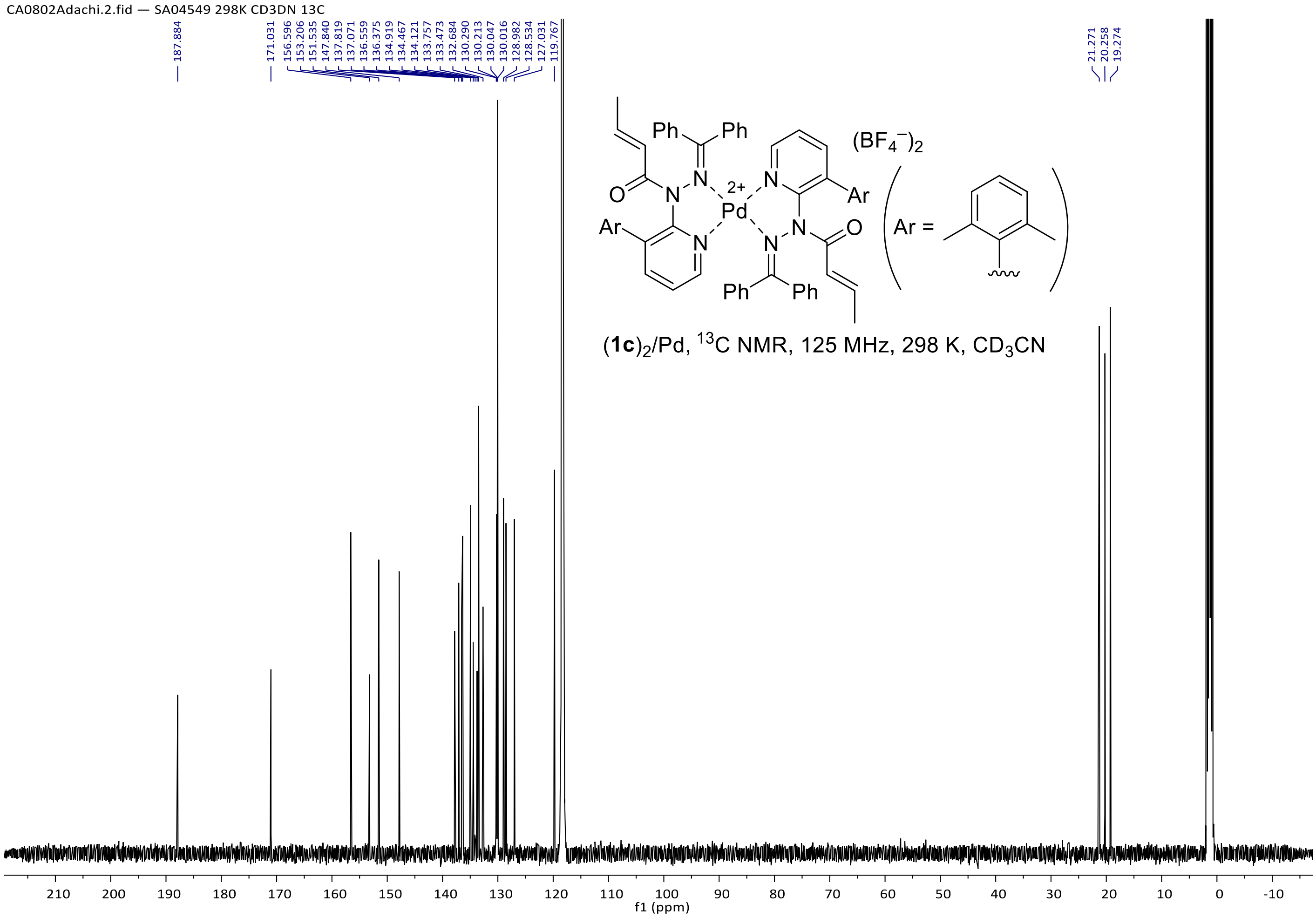
CA0802Adachi.2.fid — SA04549 298K CD3DN 13C
187.884
171.031 156.596 153.206 151.535 147.840 137.819 137.071 136.559 136.375 134.919 134.467 134.121 133.757 133.473 132.684 130.290 130.213 130.047 130.016 128.982 128.534 127.031 119.767
21.271 20.258 19.274
Ph
Ph
(BF4⁻)2
2+
O
N
N
Pd
N
Ar
Ar
N
N
N
O
Ar =
Ph
Ph
(1c)2/Pd, 13C NMR, 125 MHz, 298 K, CD3CN
f1 (ppm)

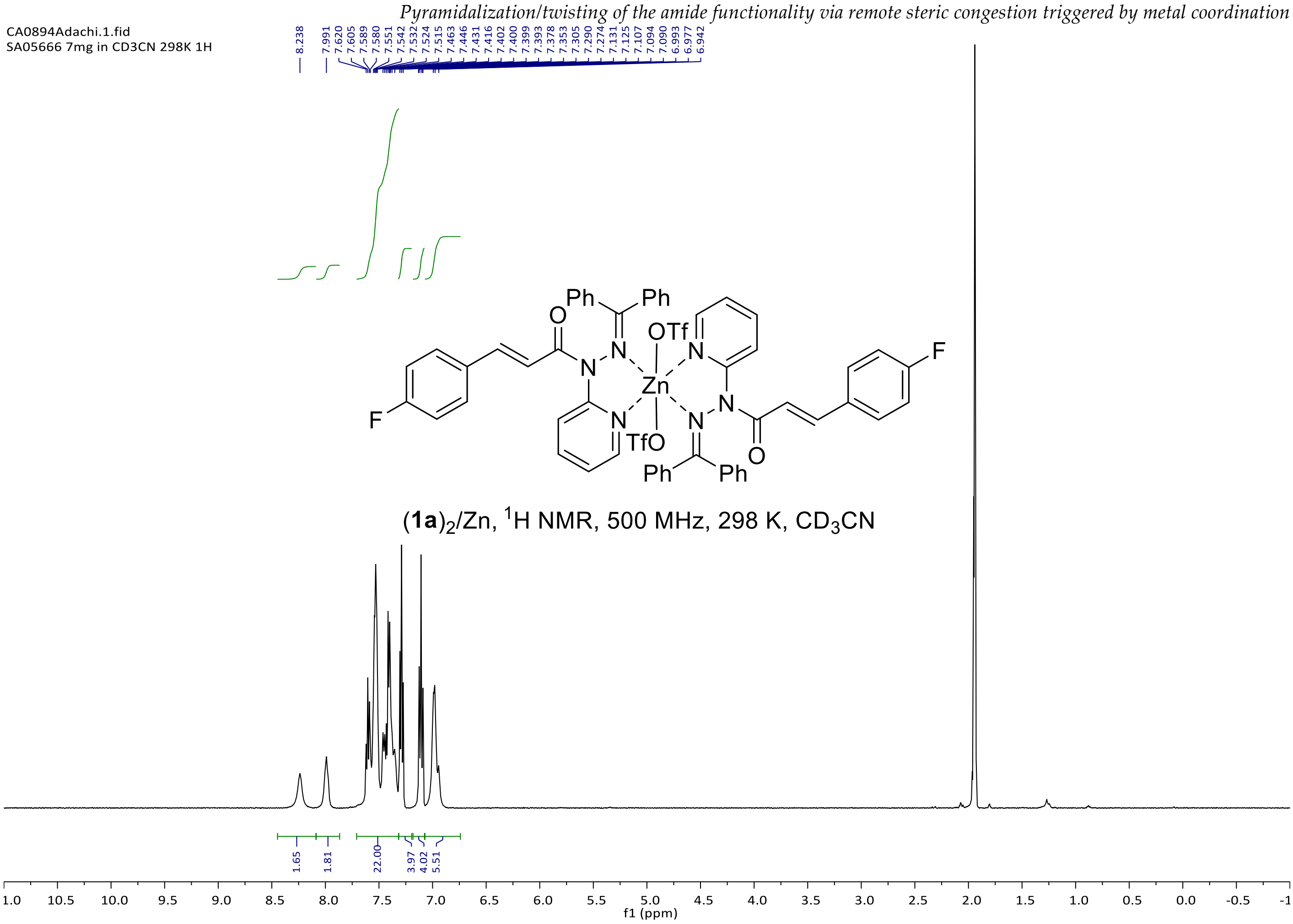

$(\mathbf{1a})_2$/Zn, ^{1}H NMR, 500 MHz, 298 K, CD_3CN

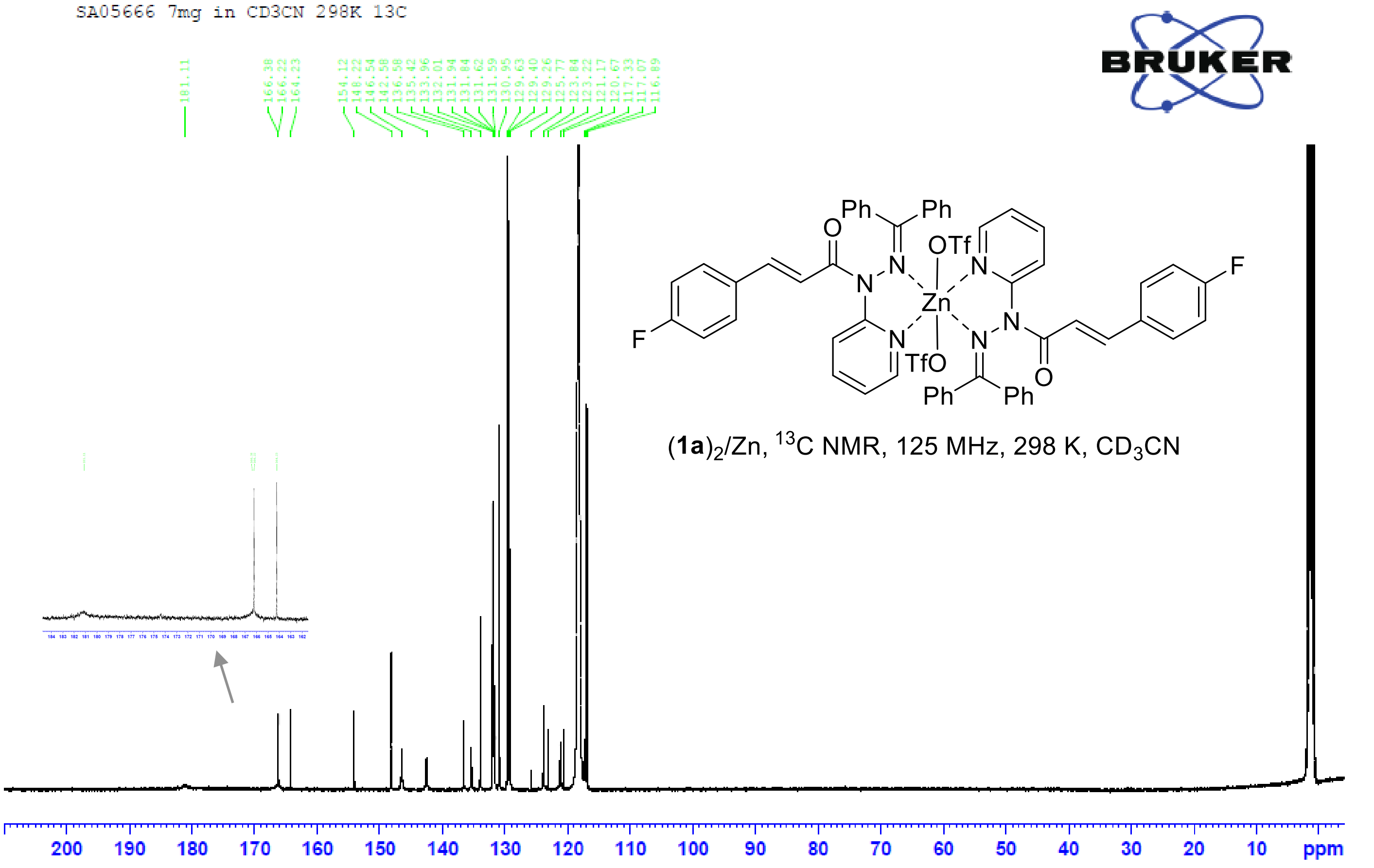

$(\mathbf{1a})_2$/Zn, ^{13}C NMR, 125 MHz, 298 K, CD_3CN

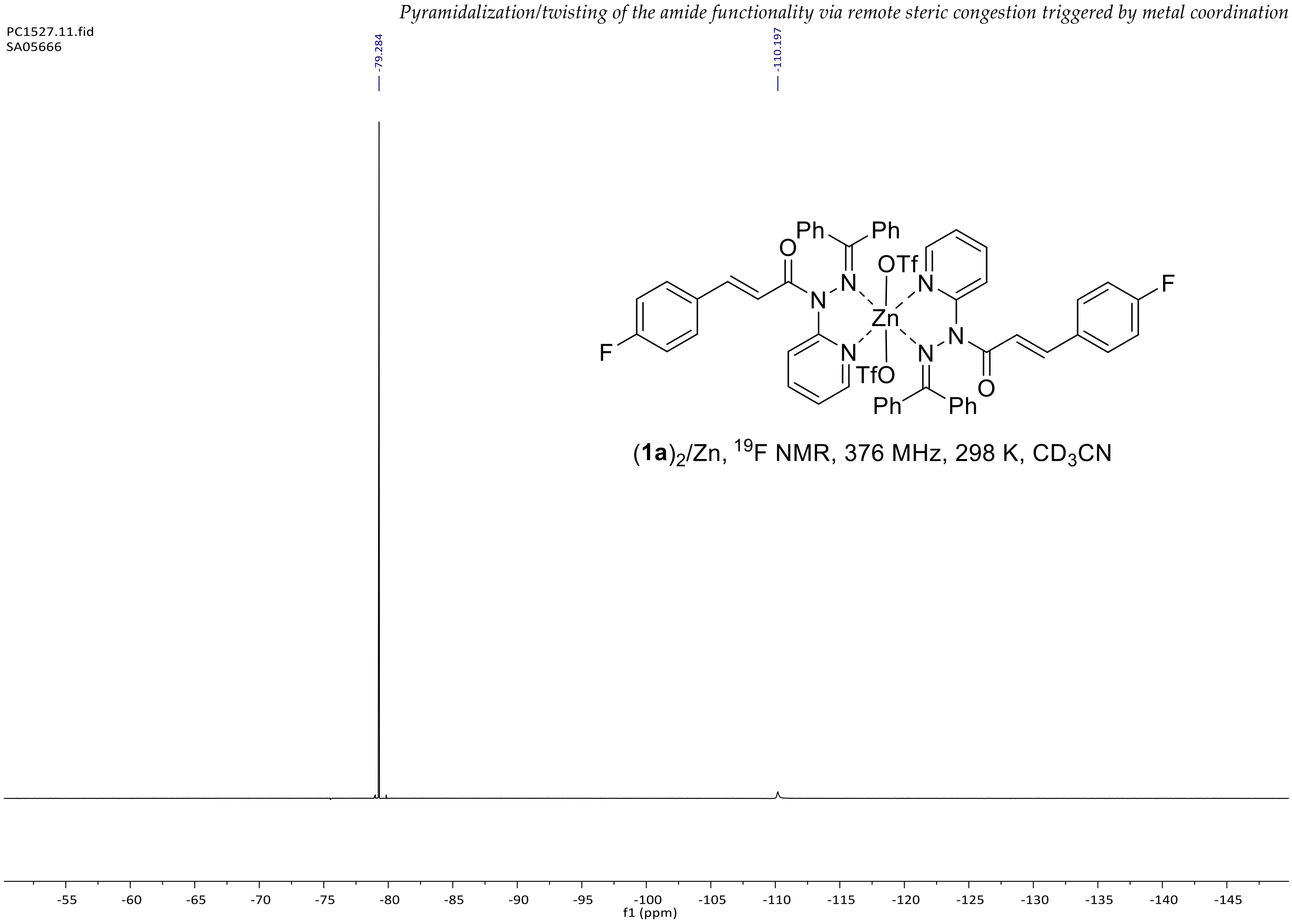
PC1527.11.fid
SA05666
-79.284
-110.197
Ph
Ph
O
OTf
N
N
Zn
F
TfO
(1a)2/Zn, 19F NMR, 376 MHz, 298 K, CD3CN
-55 -60 -65 -70 -75 -80 -85 -90 -95 -100 -105 -110 -115 -120 -125 -130 -135 -140 -145
f1 (ppm)

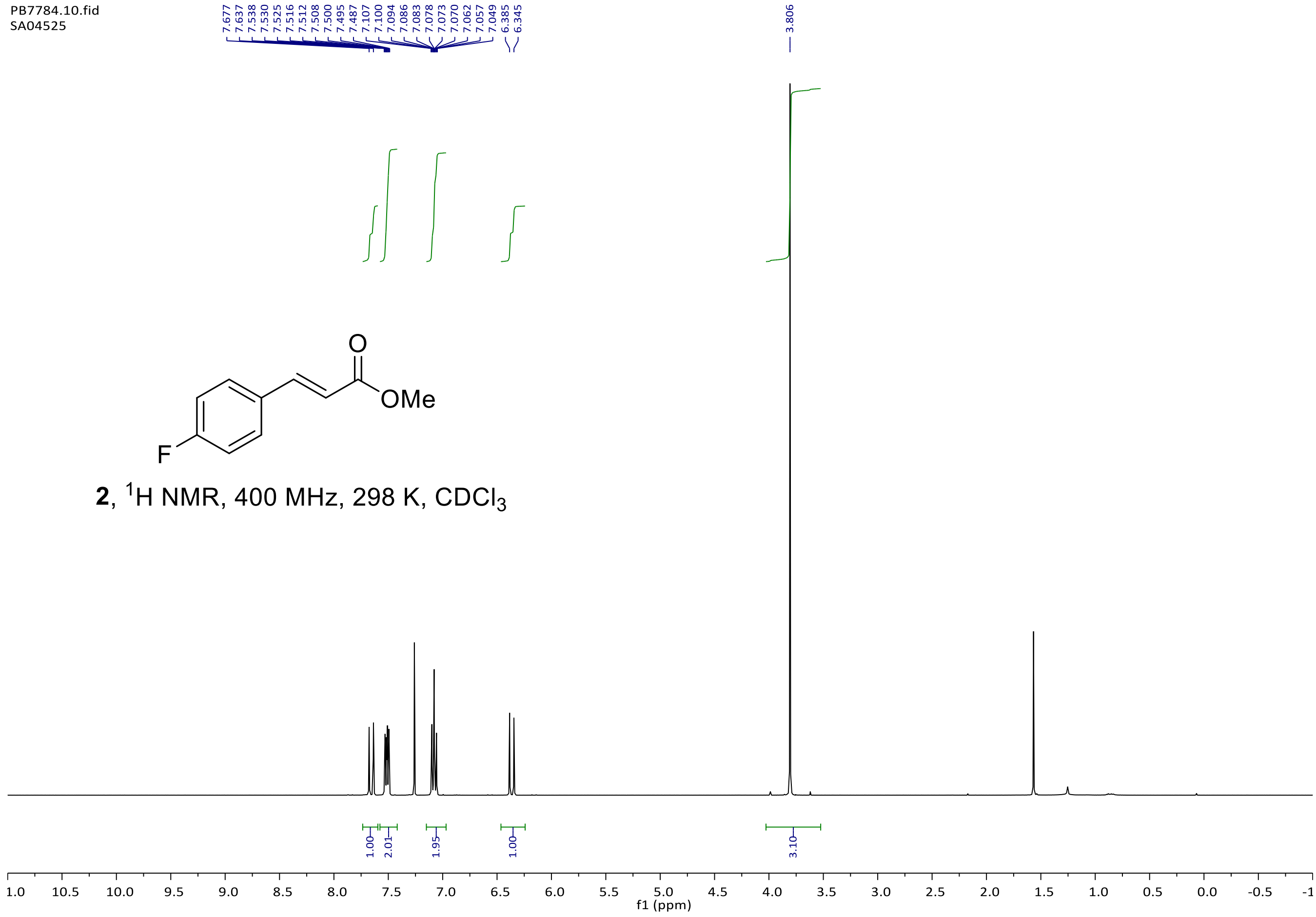
PB7784.10.fid
SA04525
7.677 7.637 7.538 7.530 7.525 7.516 7.512 7.508 7.500 7.495 7.487 7.107 7.100 7.094 7.086 7.083 7.078 7.073 7.070 7.062 7.057 7.049 6.385 6.345
3.806
O
OMe
F
2, 1H NMR, 400 MHz, 298 K, CDCl3
1.00 2.01 1.95 1.00 3.10
1.0 10.5 10.0 9.5 9.0 8.5 8.0 7.5 7.0 6.5 6.0 5.5 5.0 4.5 4.0 3.5 3.0 2.5 2.0 1.5 1.0 0.5 0.0 -0.5 -1
f1 (ppm)

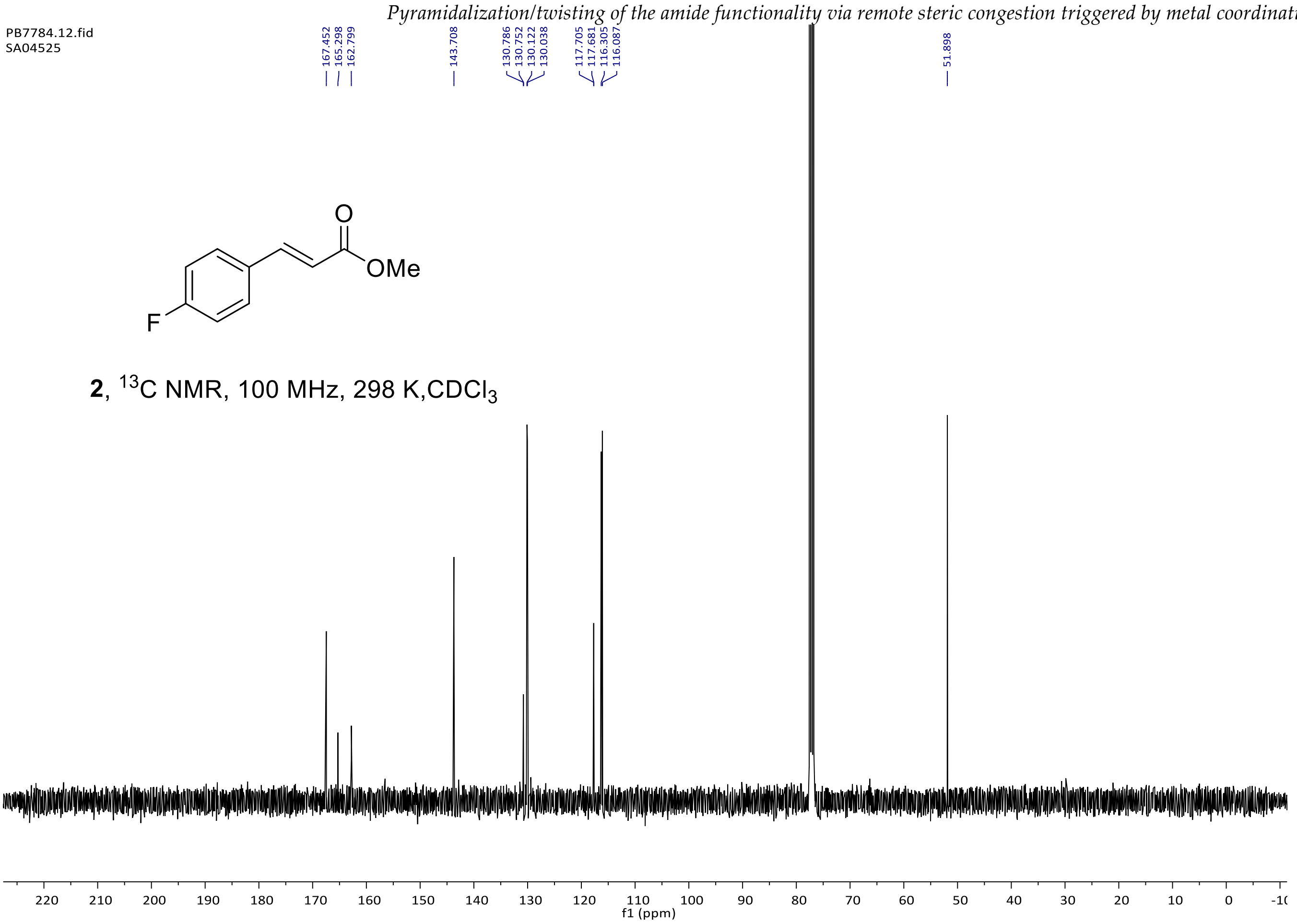
PB7784.12.fid
SA04525
167.452
165.298
162.799
143.708
130.786
130.752
130.122
130.038
117.705
117.681
116.305
116.087
51.898
O
OMe
F
2, 13C NMR, 100 MHz, 298 K,CDCl3
220 210 200 190 180 170 160 150 140 130 120 110 100 90 80 70 60 50 40 30 20 10 0
f1 (ppm)

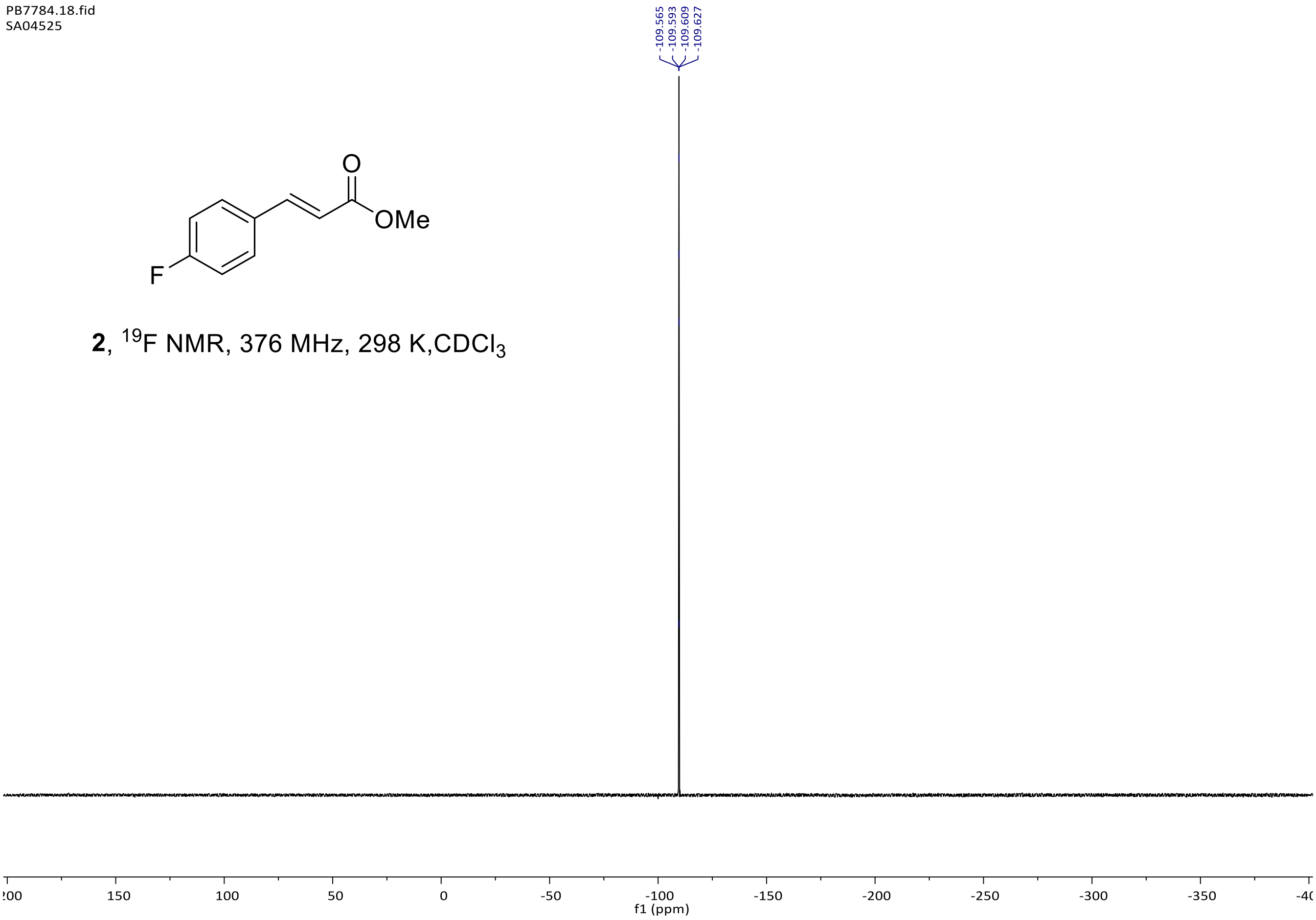
PB7784.18.fid
SA04525
-109.565
-109.593
-109.609
-109.627
O
OMe
F
2, 19F NMR, 376 MHz, 298 K,CDCl3
150 100 50 0 -50 -100 -150 -200 -250 -300 -350
f1 (ppm)
